# Cost-Effective Method to Perform SARS-CoV-2 Variant Surveillance: Detection of Alpha, Gamma, Lambda, Delta, Epsilon, and Zeta in Argentina

**DOI:** 10.3389/fmed.2021.755463

**Published:** 2021-12-10

**Authors:** Carolina Torres, Laura Mojsiejczuk, Dolores Acuña, Sofía Alexay, Ariel Amadio, Paula Aulicino, Humberto Debat, Fabián Fay, Franco Fernández, Adriana A. Giri, Stephanie Goya, Guido König, Horacio Lucero, Mercedes Nabaes Jodar, Luis Pianciola, Javier A. Sfalcin, Raúl M. Acevedo, Sofía Bengoa Luoni, Elisa M. Bolatti, Bettina Brusés, Marco Cacciabue, Pablo E. Casal, Agustina Cerri, Diego Chouhy, María José Dus Santos, María Florencia Eberhardt, Ailen Fernandez, Paula del Carmen Fernández, Darío Fernández Do Porto, Laura Formichelli, María Inés Gismondi, Matías Irazoqui, Melina Lorenzini Campos, Silvina Lusso, Nathalie Marquez, Marianne Muñoz, Javier Mussin, Mónica Natale, Griselda Oria, María Belén Pisano, Victoria Posner, Andrea Puebla, Viviana Re, Ezequiel Sosa, Gabriela V. Villanova, Jonathan Zaiat, Sebastián Zunino, María Elina Acevedo, Julián Acosta, Cristina Alvarez Lopez, María Laura Álvarez, Patricia Angeleri, Andrés Angelletti, Manuel Arca, Natalia A. Ayala, Gabriela Barbas, Ana Bertone, Agustina Bonnet, Ignacio Bourlot, Victoria Cabassi, Alejandro Castello, Gonzalo Castro, Ana Laura Cavatorta, Carolina Ceriani, Carlos Cimmino, Julián Cipelli, María Colmeiro, Andrés Cordero, Carolina Cristina, Sofia Di Bella, Guillermina Dolcini, Regina Ercole, Yesica Espasandin, Carlos Espul, Andrea Falaschi, Facundo Fernandez Moll, María Delia Foussal, Andrea Gatelli, Sandra Goñi, María Estela Jofré, José Jaramillo, Natalia Labarta, María Agustina Lacaze, Rocio Larreche, Viviana Leiva, Gustavo Levin, Erica Luczak, Marcelo Mandile, Gioia Marino, Carla Massone, Melina Mazzeo, Carla Medina, Belén Monaco, Luciana Montoto, Viviana Mugna, Alejandra Musto, Victoria Nadalich, María Victoria Nieto, Guillermo Ojeda, Andrea C. Piedrabuena, Carolina Pintos, Marcia Pozzati, Marilina Rahhal, Claudia Rechimont, Federico Remes Lenicov, Gabriela Rompato, Vanesa Seery, Leticia Siri, Julieta Spina, Cintia Streitenberger, Ariel Suárez, Jorgelina Suárez, Paula Sujansky, Juan Manuel Talia, Clara Theaux, Guillermo Thomas, Marina Ticeira, Estefanía Tittarelli, Rosana Toro, Osvaldo Uez, María Belén Zaffanella, Cecilia Ziehm, Martin Zubieta, Alicia S. Mistchenko, Laura Valinotto, Mariana Viegas

**Affiliations:** ^1^Facultad de Farmacia y Bioquímica, Instituto de Investigaciones en Bacteriología y Virología Molecular (IBaViM), Universidad de Buenos Aires, Buenos Aires, Argentina; ^2^Consejo Nacional de Investigaciones Científicas y Técnicas (CONICET), Buenos Aires, Argentina; ^3^Laboratorio de Virología, Hospital de Niños Dr. Ricardo Gutiérrez, Buenos Aires, Argentina; ^4^Instituto de Investigación de la Cadena Láctea (IDICAL) INTA-CONICET, Rafaela, Argentina; ^5^Laboratorio de Biología Celular y Retrovirus, Hospital de Pediatría “Prof. Juan P. Garrahan”, Buenos Aires, Argentina; ^6^Instituto de Patología Vegetal – Centro de Investigaciones Agropecuarias – Instituto Nacional de Tecnología Agropecuaria (IPAVE-CIAP-INTA), Córdoba, Argentina; ^7^CIBIC Laboratorio, Rosario, Argentina; ^8^Grupo Virología Humana, Instituto de Biología Molecular y Celular de Rosario CONICET, Rosario, Argentina; ^9^Instituto de Biotecnología/Instituto de Agrobiotecnología y Biología Molecular INTA-CONICET, Hurlingham, Argentina; ^10^Instituto de Medicina Regional, Universidad Nacional del Nordeste, Resistencia, Argentina; ^11^Laboratorio Central Ciudad de Neuquén, Ministerio de Salud, Neuquén, Argentina; ^12^Instituto de Botánica del Nordeste, Universidad Nacional del Nordeste-CONICET, Resistencia, Argentina; ^13^Instituto de Virología e Innovaciones Tecnológicas INTA-CONICET, Hurlingham, Argentina; ^14^Laboratorio de Diagnóstico-UNIDAD COVID- Universidad Nacional de Hurlingham, Hurlingham, Argentina; ^15^Instituto de Cálculo, Facultad de Ciencias Exactas y Naturales, Universidad de Buenos Aires, Buenos Aires, Argentina; ^16^Departamento de Ciencias Básicas, Universidad Nacional de Luján, Luján, Argentina; ^17^Unidad de Genómica del Instituto de Biotecnología/Instituto de Agrobiotecnología y Biología Molecular, INTA-CONICET, Hurlingham, Argentina; ^18^Instituto de Virología “Dr. J. M. Vanella”, Facultad de Ciencias Médicas, Universidad Nacional de Córdoba, Córdoba, Argentina; ^19^Laboratorio Mixto de Biotecnología Acuática, Facultad de Ciencias Bioquímicas y Farmacéuticas, Universidad Nacional de Rosario, Rosario, Argentina; ^20^Instituto de Química Biológica de la Facultad de Ciencias Exactas y Naturales (IQUIBICEN), CONICET, Ciudad Universitaria, Buenos Aires, Argentina; ^21^Laboratorio de Virología Molecular, Hospital Blas L. Dubarry, Mercedes, Argentina; ^22^Centro de Tecnología en Salud Pública, Facultad de Ciencias Bioquímicas y Farmacéuticas, Universidad Nacional de Rosario, Rosario, Argentina; ^23^Laboratorio del Hospital Zonal Dr. Ramón Carrillo, San Carlos de Bariloche, Argentina; ^24^Comité Operativo de Emergencia COVID, Ministerio de Salud de la Ciudad Autónoma de Buenos Aires, Buenos Aires, Argentina; ^25^Laboratorio de Salud Pública, Facultad de Ciencias Exactas, Universidad Nacional de La Plata, La Plata, Argentina; ^26^Laboratorio de Virología, HIEAyC “San Juan de Dios”, La Plata, Argentina; ^27^Laboratorio de Virología del Hospital JJ Urquiza, Concepción del Uruguay, Argentina; ^28^Laboratorio Central de Salud Pública del Chaco, Resistencia, Argentina; ^29^Secretaria de Prevención y Promoción, Ministerio de Salud de la Provincia de Córdoba, Córdoba, Argentina; ^30^Laboratorio de la Dirección de Epidemiología, Santa Rosa, Argentina; ^31^Laboratorio de Biología Molecular del Hospital Centenario, Gualeguaychú, Argentina; ^32^Unidad de Emergencia COVID-19, Plataforma de Servicios Biotecnológicos, Universidad Nacional de Quilmes, Bernal, Argentina; ^33^Laboratorio Central de la Provincia de Córdoba, Ministerio de Salud la Provincia de Córdoba, Córdoba, Argentina; ^34^Laboratorio de Virología, Facultad de Veterinaria, Universidad Nacional del Centro de la Provincia de Buenos Aires, Buenos Aires, Argentina; ^35^Instituto Nacional de Epidemiología “Dr. Jara”, Mar del Plata, Argentina; ^36^Centro de Investigaciones Básicas y Aplicadas, Universidad Nacional del Noroeste de la Provincia de Buenos Aires, Junín, Argentina; ^37^Dirección de Epidemiología y Red de Laboratorios del Ministerio de Salud de la Provincia de Mendoza, Mendoza, Argentina; ^38^Servicio de Inmunología, Hospital Dr. Julio C Perrando, Resistencia, Argentina; ^39^Laboratorio de Biología Molecular Bolívar, LABBO, Bolívar, Argentina; ^40^Programa Laboratorio de Salud Pública “Dr Dalmiro Pérez Laborda”, Ministerio de Salud de la Provincia de San Luis, San Luis, Argentina; ^41^Laboratorio de Salud Pública, Godoy Cruz, Argentina; ^42^Laboratorio del Hospital Interzonal General de Agudos “Evita”, Lanús, Argentina; ^43^Laboratorio Pediátrico Avelino Castelán, Resistencia, Argentina; ^44^Laboratorio de Biología Molecular Hospital Pedro de Elizalde, Buenos Aires, Argentina; ^45^Laboratorio Central, Santa Fe, Argentina; ^46^Servicio de Microbiología, Hospital 4 de Junio, Presidencia Roque Saenz Peña, Argentina; ^47^Laboratorio de Biología Molecular, Hospital Cosme Argerich, Buenos Aires, Argentina; ^48^Laboratorio de Hospital El Cruce Dr. Néstor C. Kirchner, Florencio Varela, Argentina; ^49^Instituto de Investigaciones Biomédicas en Retrovirus y Sida, CONICET-UBA, Buenos Aires, Argentina; ^50^Laboratorio de Biología Molecular, Hospital Dr. Héctor Cura, Olavarría, Argentina; ^51^Departamento de Biología y Genética Molecular; IACA Laboratorios, Bahía Blanca, Argentina; ^52^Laboratorio de Biología Molecular del Hospital General de Agudos Dr. Carlos G. Durand, Buenos Aires, Argentina; ^53^Ministerio de Ciencia, Tecnología e Innovación, Buenos Aires, Argentina; ^54^Comisión de Investigaciones Científicas de la Provincia de Buenos Aires, La Plata, Argentina

**Keywords:** SARS-CoV-2, variants, South America, surveillance, spike sequences, Gamma, Lambda, Delta

## Abstract

SARS-CoV-2 variants with concerning characteristics have emerged since the end of 2020. Surveillance of SARS-CoV-2 variants was performed on a total of 4,851 samples from the capital city and 10 provinces of Argentina, during 51 epidemiological weeks (EWs) that covered the end of the first wave and the ongoing second wave of the COVID-19 pandemic in the country (EW 44/2020 to EW 41/2021). The surveillance strategy was mainly based on Sanger sequencing of a Spike coding region that allows the identification of signature mutations associated with variants. In addition, whole-genome sequences were obtained from 637 samples. The main variants found were Gamma and Lambda, and to a lesser extent, Alpha, Zeta, and Epsilon, and more recently, Delta. Whereas, Gamma dominated in different regions of the country, both Gamma and Lambda prevailed in the most populated area, the metropolitan region of Buenos Aires. The lineages that circulated on the first wave were replaced by emergent variants in a term of a few weeks. At the end of the ongoing second wave, Delta began to be detected, replacing Gamma and Lambda. This scenario is consistent with the Latin American variant landscape, so far characterized by a concurrent increase in Delta circulation and a stabilization in the number of cases. The cost-effective surveillance protocol presented here allowed for a rapid response in a resource-limited setting, added information on the expansion of Lambda in South America, and contributed to the implementation of public health measures to control the disease spread in Argentina.

## Introduction

The emergence of SARS-CoV-2 variants with concerning characteristics to public health has attracted the attention of the scientific community and governments both regionally and globally since the end of 2020. The most relevant variants described so far include: Alpha (lineage B.1.1.7), first detected in the United Kingdom; Beta (lineage B.1.351), initially detected in South Africa; Gamma (lineage P.1), initially detected in Manaus, Brazil, and Japan; Delta (lineage B.1.627.2), initially detected in India; Lambda (lineage C.37), initially detected in Peru; Mu (lineage B.1.621), first detected in Colombia; Epsilon (lineages B.1.427 and B.1.429), initially detected in California, United States; and Zeta (lineage P.2), first detected in Rio de Janeiro, Brazil (1). Four of these variants (Alpha to Delta) have been defined as variants of concern (VOCs) given their increased transmissibility and other characteristics, while Lambda and Mu have been defined as variants of interest (VOIs). The VOCs have also been associated with an increased risk of hospitalization (2, 3) and, in the case of Beta, Gamma, and Delta, with a moderate to a substantial reduction in neutralizing activity of monoclonal antibodies, convalescent, and vaccine sera (4–6). Gamma and Lambda are particularly relevant for Argentina due to their major presence in the South American region during the time of this study.

Importantly, some of these variants share mutations in the Spike protein—several of them in the receptor-binding domain region—that potentially affect transmissibility, pathogenesis, and/or response to vaccination and immune-based therapies (7, 8).

PAIS is the interinstitutional federal consortium of SARS-CoV-2 genomics in Argentina. It was created by the Ministry of Science and Technology to monitor SARS-CoV-2 diversity and evolution in the country, including surveillance of SARS-CoV-2 variants of public health interest (http://pais.qb.fcen.uba.ar/).

The objective of this work was to implement a SARS-CoV-2 molecular surveillance strategy, in a context of limited resources, which allowed an assessment of the dynamic situation of circulation of viral variants, and at the same time, to perform genomic and evolutionary analyzes to study their origin and dispersion in our country.

## Materials and Methods

### Samples

**Figure 1:**
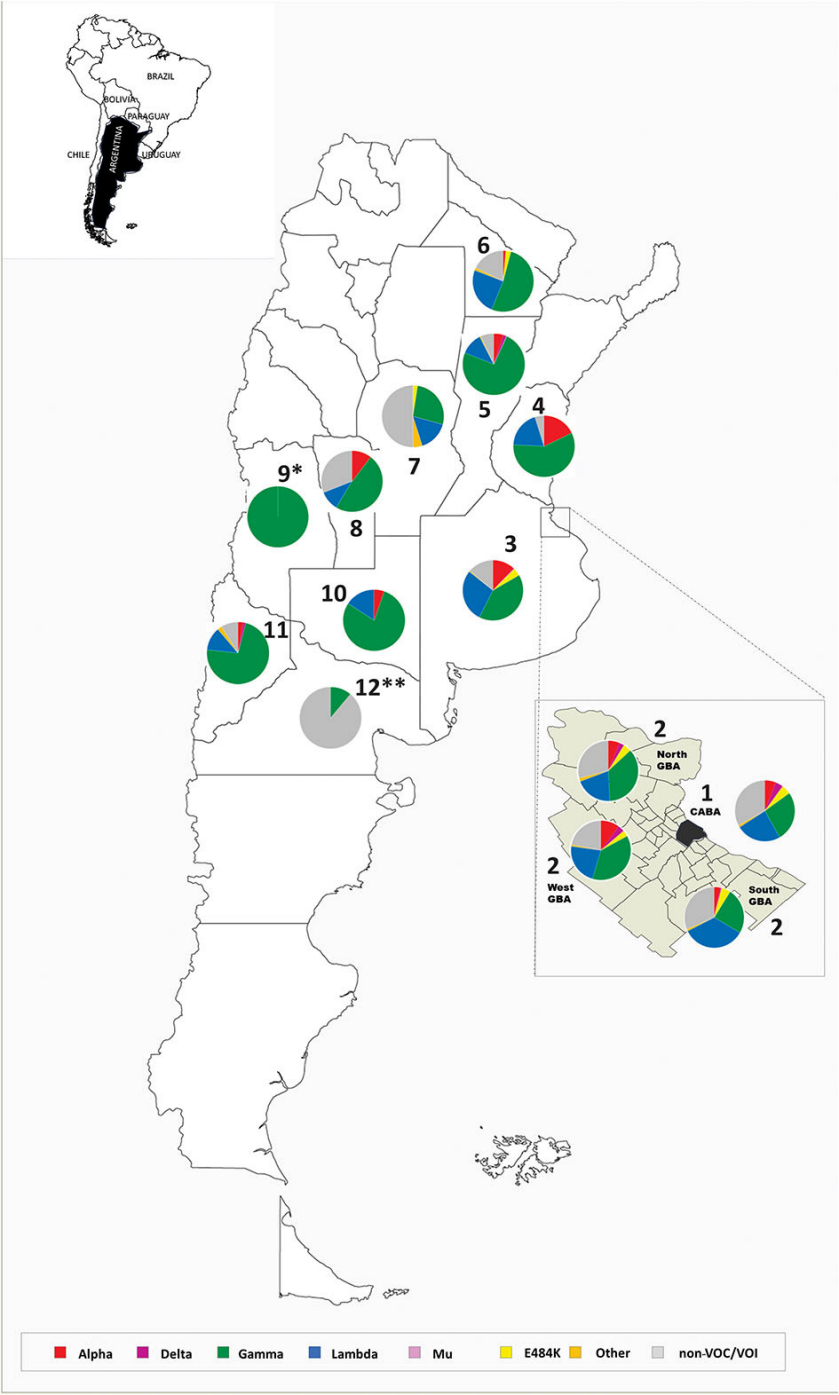
Location of cases analyzed in this work and pie charts representing the frequency of each variant detected in every region between EW44/2020 and EW41/2021. 1. Buenos Aires city, 2. Great Buenos Aires (North, West, South), 3. Province of Buenos Aires, 4. Province of Entre Ríos, 5. Province of Santa Fe, 6. Province of Chaco, 7. Province of Córdoba, 8. Province of San Luis, 9. Province of Mendoza, 10. Province of La Pampa, 11. Province of Neuquén, and 12. Province of Río Negro. *cases associated with an outbreak (*n* = 15) (Supplementary Table 1). **cases (*n* = 9) mainly associated with the first part of the period analyzed (Supplementary Table 1).

**Figure 2:**
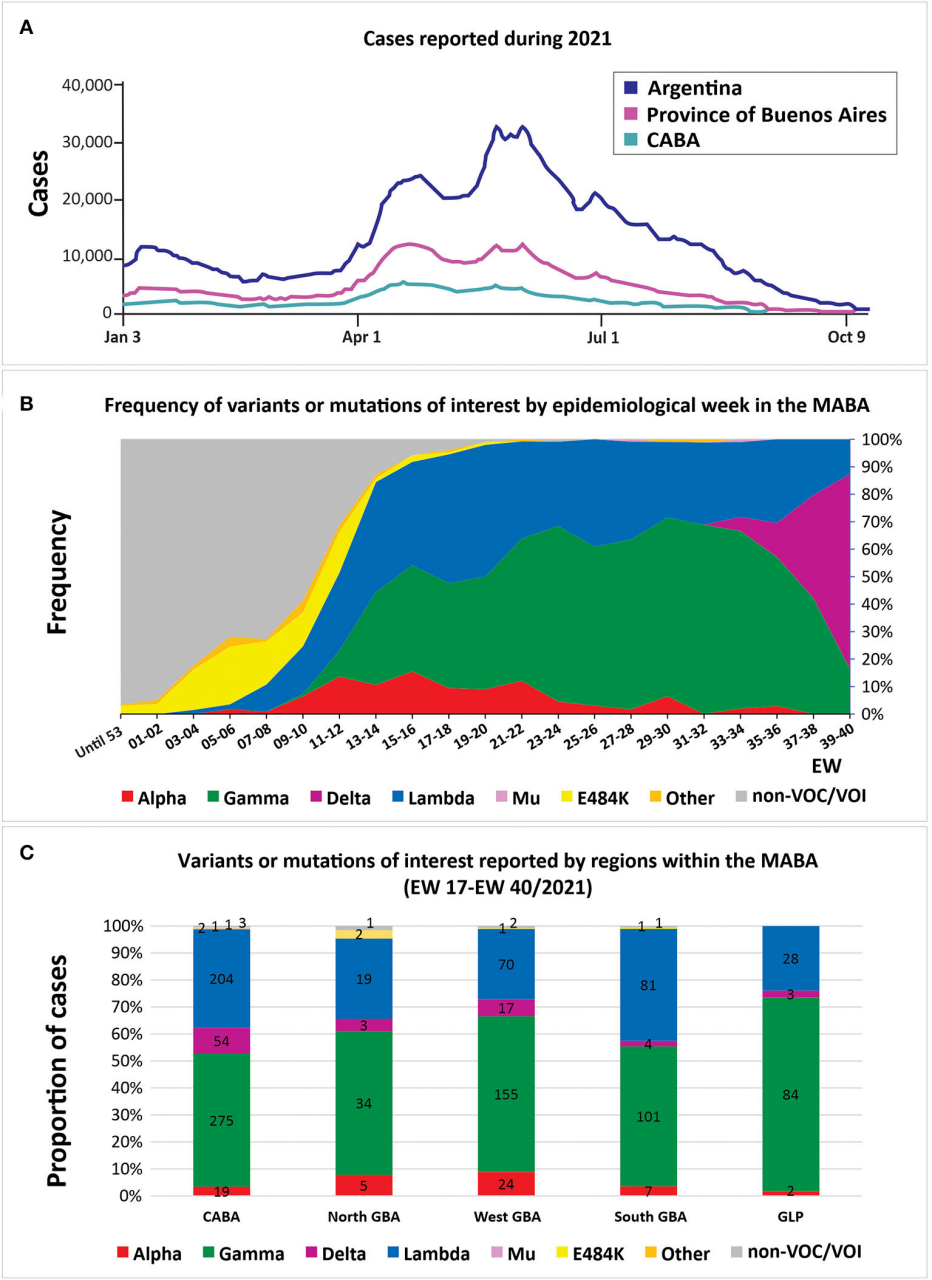
**(A)** Confirmed cases in Argentina, the city of Buenos Aires (CABA) and the province of Buenos Aires in the year 2021. **(B)** Frequency of SARS-CoV-2 variants and sequences with or without mutations of interest by epidemiological week (*n* = 2,625). Only cases from the metropolitan area of Buenos Aires (MABA) that did not present a history of travel or close contact with travelers are included; in cases with registered epidemiological links, only one case was considered representative. **(C)** The cumulative number of cases between EW 17/2021 and EW 40/2021 of SARS-CoV-2 variants and sequences with or without mutations of interest within the different regions of the MABA: CABA, Great Buenos Aires (GBA), and Great La Plata (GLP). Only cases that did not present a history of travel or close contact with travelers are included; in cases sharing an epidemiological link, only one was included. **(A,B)** are on the same time scale.

Between October 26th, 2020 and October 12th, 2021 (epidemiological week (EW) 44/2020 to EW41/2021), molecular surveillance was performed on a total of 4,851 samples from the capital city and 10 provinces of the country, including the four most populated districts (Figure 1, Supplementary Table 1). This period covers the end of the first wave and the second and largest wave of the COVID-19 pandemic in Argentina (Figure 2A). During this period, the frontiers were mostly open for Argentinean residents, but the foreigners had severe restrictions to enter the country as tourists.

For this work, samples analyzed included a randomly selected 2.5–50% fraction of the total positive cases weekly detected in different healthcare centers. The proportion of samples analyzed in each week was associated with the absolute number of samples that could be processed by the methodology implemented according to the number of cases reported at the beginning, at the peak, and toward the end of the second wave in the country. Regular sampling from four sentinel laboratories located in the metropolitan area of Buenos Aires (MABA) was performed along with sporadic sampling from other locations. Most of the samples corresponded to randomly selected cases with no epidemiological link among them or with international travel (*n* = 4,632), while a minor share corresponded to outbreak studies, special cases, or confirmatory studies of specific real time-PCR methodologies for the detection of variants (*n* = 219) (Supplementary Table 1).

### Sequencing

The surveillance strategy was based on Sanger sequencing of a 970 nt region of Spike encoding amino acids 428–750 (Supplementary Figure 1) (9). This region allows the identification of signature mutations associated with variants Alpha, Beta, Gamma, Delta, Lambda, and Mu. In addition, complete SARS-CoV-2 genome sequences were obtained from 637 samples using the Quick protocol (10) with Oxford Nanopore or Illumina platforms. Both strategies combined allowed to perform the analysis of 4,851 sequences (Supplementary Table 1).

### Statistical Analysis

The frequencies of detection of VOI, VOC, or mutations of interest and the 95% CIs were estimated with the Wilson/Brown method, implemented in the Graph Pad Prism v.9.2 program (San Diego, CA, United States, www.graphpad.com).

### Phylogenetic Analysis

Phylogenetic analysis was carried out for VOCs Alpha and Gamma to study their introduction and initial spread in Argentina. Datasets included the Argentine sequences from the first part of the second wave, their best five BLAST hits sequences (against the GISAID database on June 2nd, 2021), reference sequences of B.1.1.7 or P.1 lineages, and B.1.1.1 or B.1.1.28 sequences as outgroups, respectively. Alignments were built using MAFFT v7.486 (11) and maximum likelihood trees were built using IQ-TREE v.2.1 (12). The SH-like approximate likelihood ratio test (1,000 replicates) (13) and ultrafast bootstrap approximation (1,000 replicates) (14) were used as methods to evaluate the reliability of the groups and branches obtained. We gratefully acknowledge the authors from the originating laboratories responsible for obtaining the specimens and the submitting laboratories where genetic sequence data were generated and shared *via* the GISAID Initiative, on which part of this research is based (Supplementary Tables 4, 5).

### Ethics Statement

The study was revised and approved by the Medical Ethics and Research Committees of “Ricardo Gutiérrez” Children's Hospital, Buenos Aires, Argentina (DI-2020-165-GCABA-HGNRG). Informed consent was not obtained, as patient information was anonymized and de-identified before analysis.

## Results

### Alpha

**Alpha** was identified in 307 cases. This variant was detected in the city of Buenos Aires (CABA) and in the provinces of Buenos Aires, Córdoba, Chaco, Entre Ríos, Santa Fe, San Luis, La Pampa, and Neuquén. Its frequency in the MABA region (the CABA, Great Buenos Aires (GBA), Great La Plata (GLP), and surrounding areas) reached 15.5% (95% CI = 11.2–21.0) in EW 15-16/2021 (April 11th−24th) but decreased to 0% by EW 37-38/2021 (September 12th−25th, 2021) (Supplemetary Tables 1, 2, Figure 2).

**Figure 3:**
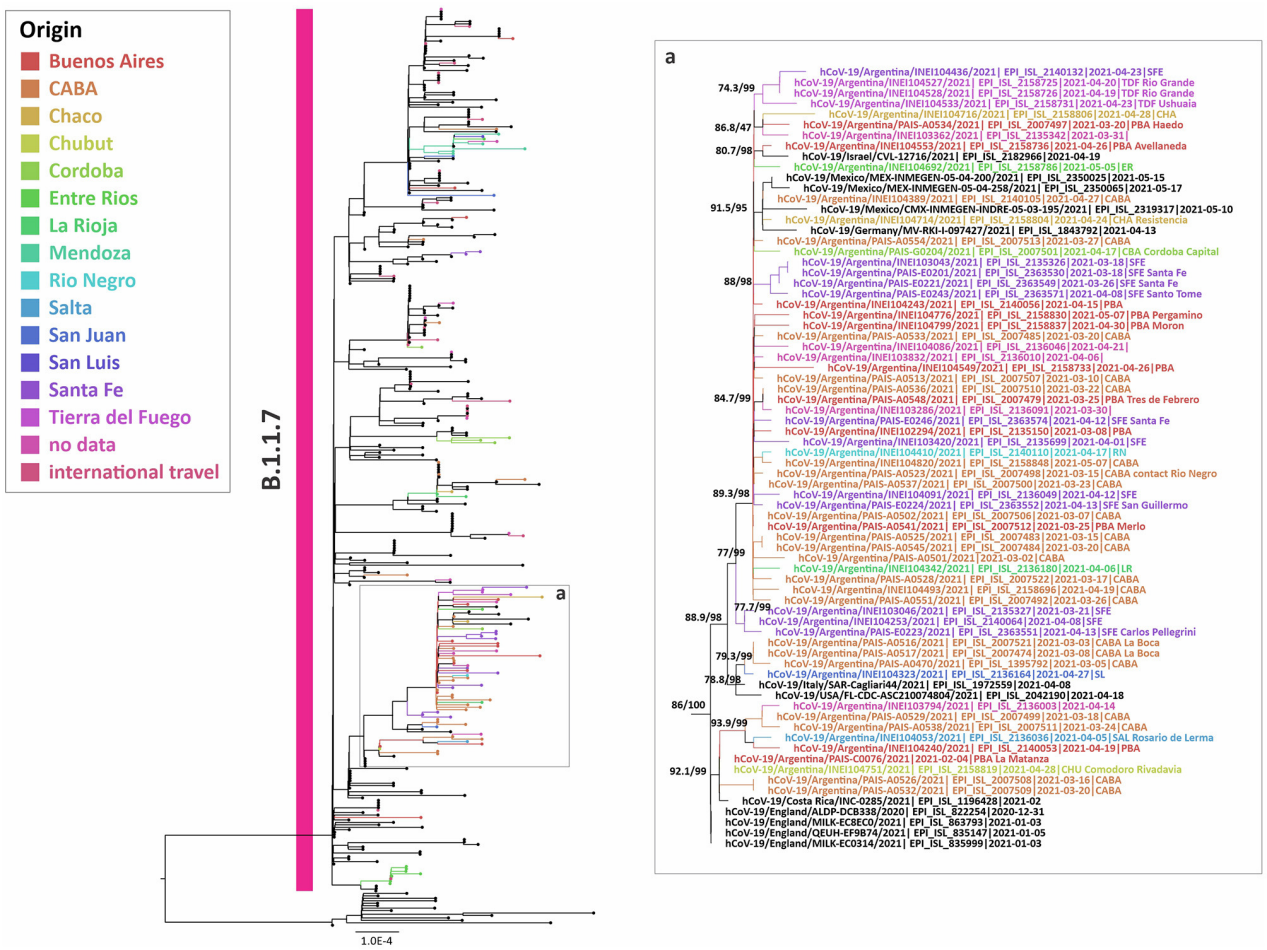
Phylogenetic tree of SARS-CoV-2 whole-genome sequences of Alpha (lineage B.1.1.7). B.1.1.1 sequences were used as outgroup. Only the largest group with Argentinean sequences is shown. The SH-like/UFB values for the relevant groups are indicated for some groups. UFB, ultrafast bootstrap. The right panel **(a)** represents an inset of the largest B.1.1.7 group from the tree.

To study the introduction of Alpha in our country, the dominant variant of the second wave throughout the northern hemisphere and other locations, a phylogenetic analysis of complete genome sequences was carried out. This analysis included 127 sequences from Argentina (43 from this work) and showed at least 35 independent introductions to the country, being the most related sequences from the USA, Central America, Europe, and the Middle East. Besides, five highly supported groups with at least three Argentinean sequences were observed (Figure 3). The largest group included 53 sequences from the CABA and nine provinces, from north to south of the country, suggesting a widespread local transmission and diversification.

### Gamma

**Figure 4:**
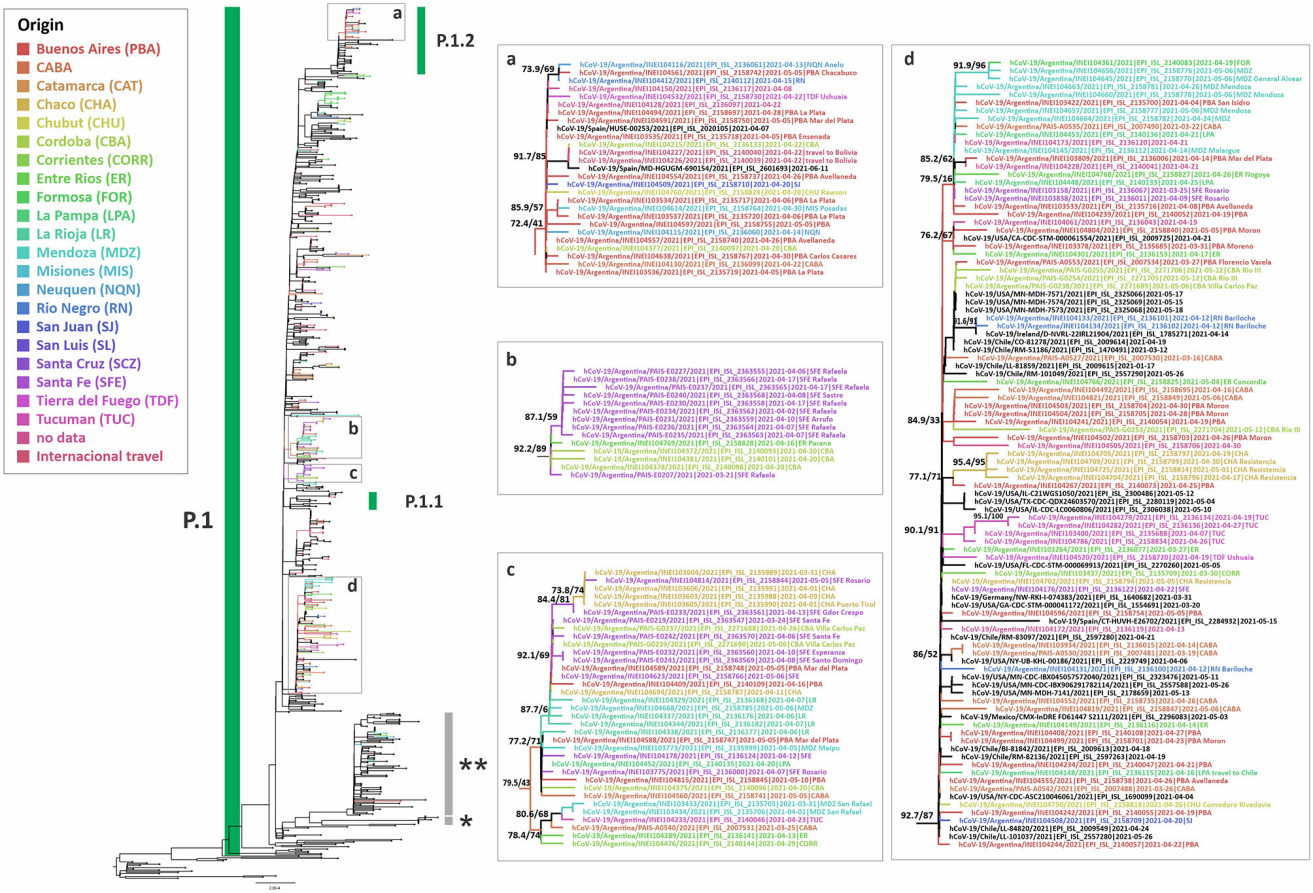
Phylogenetic tree of SARS-CoV-2 whole-genome sequences of Gamma (lineage P.1). B.1.1.28 sequences were used as outgroup. Only selected groups with Argentinean sequences are shown. The SH-like/UFB values for the relevant groups are indicated for some groups. *P.1-like-I and **P.1-like-II described by Gräf et al. (15). UFB, ultrafast bootstrap. The panels **(a–d)** represent insets of some groups from the tree.

**Gamma** was predominant in the second wave of the pandemic in Argentina, found in 2,123 out of 4,851 (43.8%) analyzed cases. This variant was detected in all the regions analyzed. Its frequency in the MABA remained at values over 30% since EW 13-14/2021, reaching a maximum of 68.9% (95% CI = 58.7–77.5) during EW 31-32/2021 (August 1st−14th, 2021). The study of the introduction of Gamma to Argentina through phylogenetic analysis with complete genomic sequences (including 238 from Argentina, 50 from this work) showed at least 50 introductions to the country, with the most related sequences from Brazil and the USA. Besides, 18 supported groups with at least three Argentinean sequences were observed (Figure 4). The largest one included 72 sequences from the CABA and 14 provinces, from north to south of the country, which suggests a wide local transmission and diversification of a common transmission chain together with the establishment of other more geographically limited chains (Figure 4d). Eight sequences from Argentina were located in a clade that showed a basal diversification within the P.1 lineage [previously named as P.1-like-II, (15)]. This clade was composed of sequences from South America only (Brazil, Chile, and Argentina) (Figure 4), which shows a diversification process at a regional level.

### Lambda

**Lambda** variant showed a continuous increase in MABA since EW 7-8/2021, reaching its maximum frequency of 48.0% (95% CI = 38.5–57.7) during EW 19-20/2021 (May 9th−22nd). After Gamma, this VOI was the most frequent one in the second wave of the country, until its recent displacement by the Delta variant.

### Delta

**Delta** variant has been detected in 103 randomly collected samples, with a frequency in the MABA that increased from 5.1% (95% CI % = 2.2–11.3) in EW 31-32/2021 to 70.9% (95% CI = 57.9–81.2%) in EW 39-40/2021 (from September 26th to October 9th, 2021). Its entry was also noticed in the provinces of Santa Fe and Neuquén, where Gamma was also the predominant variant throughout the second wave. In the last weeks analyzed, Delta was found with a frequency of 26.3% (95% CI = 11.8–48.8) in Santa Fe (EW 39-40/2021) and of 18.2% (95% CI % = 3.2–47.7) in Neuquén (EW 41/2021).

### Variant Distribution

The distribution of variants of epidemiological interest was heterogeneous within the MABA region. This could be evidenced when the VOIs/VOCs reached 95% of the total detections in the MABA (between EW 17/2021 and EW 40/2021), a time that marks the beginning of the characteristic pattern of the second wave in the country.

The North GBA presented a marked predominance of Gamma (34/64 cases, 53.1%), followed by Lambda (19/64 cases, 29.7%), Alpha (5/64, 7.9%), and Delta (3/64, 4.7%), similar to the West GBA that presented Gamma in 155/269 cases (57.6%), Lambda in 70/269 cases (21.5%), Alpha in 24/269 cases (8.9%), and Delta in 17/269 cases (6.3%). On the other hand, in the same period, the CABA presented a more homogeneous distribution of Gamma and Lambda, with 275/559 cases (49.2%) and 204/559 cases (36.5%), respectively, followed by Delta in 54/559 cases (9.7%)—detected since EW 35-36/2,021—and Alpha, in 19/559 cases (3.4%). It should be mentioned that the Mu variant was marginally detected in 2/559 cases (EW 27-28/2021 and EW 33-34/2021). Likewise, the South GBA presented Gamma in 101/195 cases (51.8%), followed by Lambda in 81/195 cases (41.5%), Alpha in 7/195 cases (3.6%), and Delta in 4/195 cases (2.1%). Finally, GLP showed a remarkable predominance of Gamma, with 84/117 (71.8%) (Figure 2C).

The analysis of sporadic sampling from various parts of the country showed Gamma as the predominant variant in most of the provinces studied, although it also revealed a heterogeneous distribution of other variants and mutations of interest (Supplementary Table 1).

### Mutations of Interests

The E484K mutation—not associated with VOC/VOI signatures—was found in 144 cases. Most of the samples were from the CABA and the GBA regions from individuals without travel history, suggesting local circulation, at least, since the last week of December 2020, when it was first detected by our surveillance program. Nevertheless, a reduction in its frequency was observed since EW 12/2021 in the MABA, being replaced by VOCs and VOIs of more recent emergence, such as Lambda (Figure 2B, Supplementary Table 2). So far, 53 of 144 cases were analyzed by full-length sequencing and all of them have been identified as Zeta.

Other mutations of interest, such as L452R—as the unique mutation of interest detected in the sequenced region—have been occasionally observed in several regions, mainly in the MABA (Supplementary Table 1), although it never exceeded 5% of detections (Figure 1). The complete genome analysis of 14 cases allowed the identification of the Epsilon variant in 12 (one from the lineage B.1.429 and 11 from the lineage B.1.427) and the lineage A.2.5 in two cases.

## Discussion

In this work, we show genomic evidence of the introduction and local transmission of the SARS-CoV-2 variants Gamma, Lambda, and Delta, and to a lesser extent, of Alpha, Epsilon, and Zeta in Argentina, the sporadic detection of Mu and the circulation of mutations of interests (such as L452R and E484K in Spike), in different geographical regions of the country.

The main lineages that circulated toward the end of the first wave were completely replaced by emerging variants at the regional (such as Gamma and Lambda) and global (such as Alpha and Delta) levels within a few weeks, as was previously observed in other countries of the region (16–18).

These results demonstrate the impact of the regional circulation of SARS-CoV-2 variants on the epidemiology of Argentina. Importantly, these data have contributed to the WHO declaration of Lambda as a global VOI (19). The neutralization capacity of sera, transmission, clinical behavior, and impact on vaccine effectiveness of this VOI is being studied. The first results have shown that the neutralization capacity of convalescent sera from the first wave in Argentina and sera from individuals vaccinated with Sputnik V was not compromised (6, 20). On the other hand, increased infectivity and resistance to neutralizing antibodies produced by individuals immunized with CoronaVac (SinoVac) and mRNA-1273 (Moderna) vaccines were observed (21–23).

Mutations at aa positions 484 and 452 of Spike protein have also been detected, with several samples confirmed as Zeta or Epsilon variants, respectively. These mutations have been associated with possible immune escape and modified affinity to the human receptor and may occur in various newly emerging lineages worldwide (22, 24, 25). Both positions are located within the receptor-binding motif. On one hand, the E484K mutation, constitutively present in Beta, Gamma, Mu, and Zeta variants, was associated with resistance to neutralization by monoclonal antibodies, convalescent, and vaccinated sera (4, 26–28). On the other hand, mutations at position 452 of the Spike protein, were associated with decreased neutralization by monoclonal antibodies, convalescent, and some vaccine sera (22, 24, 29). Another important mutation in Spike is P681R, recently associated with an enhanced viral fusion, which would result in a more efficient entry of the virus into the cell and a greater capacity for the virus to spread through cell-cell passages (30–32).

In recent months, the Delta variant has spread to multiple countries, causing new waves of infections around the world. Although South America is at the end of its second wave and is the region with the lowest prevalence of Delta in the period studied, this variant has already been detected in all the countries of the region and many of them, showing community transmission. The latest data from neighboring countries indicate that Delta is already dominant in Brazil, Chile, and Uruguay, and that community circulation has been verified in Paraguay. The latest reports indicate that Delta is found in ~87–92% of the cases in Chile during EW 38-39/2021 (18), in ~91.8% of the cases in Brazil during September 2021 (33), and 100% cases in Uruguay during October 2021 (34).

In this work, the proportion of Delta increased from 5% to more than 70% of MABA cases in eight epidemiological weeks, displacing the Gamma and Lambda variants, which were predominant in the second wave in the country. Similarly, an increase in Delta frequency detection, displacing Gamma, began to be observed in Santa Fe and Neuquén provinces. In this context, it is expected that eventually, Delta will be dominant in Argentina as in most countries.

These results showed a shared regional epidemiological situation between Argentina and neighboring countries, up to now characterized by a concurrent increase in Delta detection frequency, simultaneously with the report of a stable number of cases. However, the latest data would indicate that this trend could begin to reverse both in Chile and in some regions of Argentina, where cases have started to slowly increase, accompanied by the opening of massive social activities.

South American countries are facing the verge of a potential third wave, which could be restricted and/or favored by specific population groups that have not yet been vaccinated. The uncertainties in terms of the future epidemiological landscape in the region, the unknown lasting protection of the vaccines already applied, or the potential emergence of novel variants leading to antigenic drift, reinforce the importance of the molecular surveillance studies of viral circulation with a focus on the regional level.

In conclusion, the surveillance strategy implemented over 51 epidemiological weeks in Argentina, based on Spike and complete genome sequencing, allowed us to describe the introduction, establishment, dispersion, and evolution of SARS-CoV-2 variants of interest and concern in the second wave of the COVID-19 pandemic in Argentina and contributed to the description of variants of importance for South America. The main variants found were Gamma, Lambda, Delta, and to a lesser extent, Alpha, Zeta, and Epsilon. This implementation allowed a rapid response in a limited-resources scenario and contributed to the implementation of public health measures to control disease spread in the country.

## Data Availability Statement

The datasets presented in this study can be found in online repositories. The names of the repository/repositories and accession number(s) can be found in the article/Supplementary Material.

## Author Contributions

CTo, PAu, and GK analyzed data and co-wrote the article. LMoj and HD analyzed data and provided critical revision of the article. DA, SA, MN, and LV performed experiments, analyzed data, and provided critical revision of the article. SGoy designed analysis tools, performed experiments, and provided critical revision of the article. AAm, AGi, ACe, BB, DC, EB, FF, GV, GOr, HL, LF, LP, MD, JM, ML, AFe, PC, PF, MG, SL, NM, MMu, MNa, BP, APu, VP, VR, RA, SZ, GB, GC, MM, CP, AS, GT, ET, and CZ performed experiments and analyzed data. MCa and MI designed analysis tools and performed experiments. DF and JZ provided computing resources. ES provided computing resources and analyzed data. ACa, SB, CCa, ME, MAc, CA, MAl, AAn, APi, MA, ABe, ABo, IB, VC, ACa, CCe, CCi, GM, JC, MCo, ACo, CCr, SD, GD, GV, RE, YE, CE, AFa, FFM, AGa, SGon, MF, MJ, JA, JJ, NA, NL, ML, RL, VL, GL, EL, MMa, CMa, CMe, BM, LMon, VM, AMu, VN, MNi, GOj, MP, MR, CR, FR, GR, VS, LS, JSp, CS, JSu, JT, CTh, MT, RT, OU, MZa, and MZu collected samples and provided data. PAn and PS provided data. AMi provided and analyzed data and provided critical revision of the article. MV analyzed data, co-wrote the paper, and coordinated the project. All authors contributed to the article and approved the submitted version.

## Funding

This work was supported by the Proyecto IP COVID-19 N°08 (Ministerio de Ciencia, Tecnología e Innovación, Argentina) and the Focem COF 03/11 Covid-19 (Fondo para la Convergencia Estructural del MERCOSUR). Funding had no role in the study design, collection, analysis, or interpretation of data, in the writing or in the decision to submit the article for publication.

## Conflict of Interest

The authors declare that the research was conducted in the absence of any commercial or financial relationships that could be construed as a potential conflict of interest.

## Publisher's Note

All claims expressed in this article are solely those of the authors and do not necessarily represent those of their affiliated organizations, or those of the publisher, the editors and the reviewers. Any product that may be evaluated in this article, or claim that may be made by its manufacturer, is not guaranteed or endorsed by the publisher.
