# Supplementary material for: Cost-Effective Method to Perform SARS-CoV-2 Variant Surveillance: Detection of Alpha, Gamma, Lambda, Delta, Epsilon, and Zeta in Argentina
Source: Front Med (Lausanne). 2021 Dec 10;8:755463. doi: 10.3389/fmed.2021.755463 (PMC8703000; doi:10.3389/fmed.2021.755463)
Supplement: Supplementary file 1 [file Data_Sheet_1.pdf]

Supplementary Material

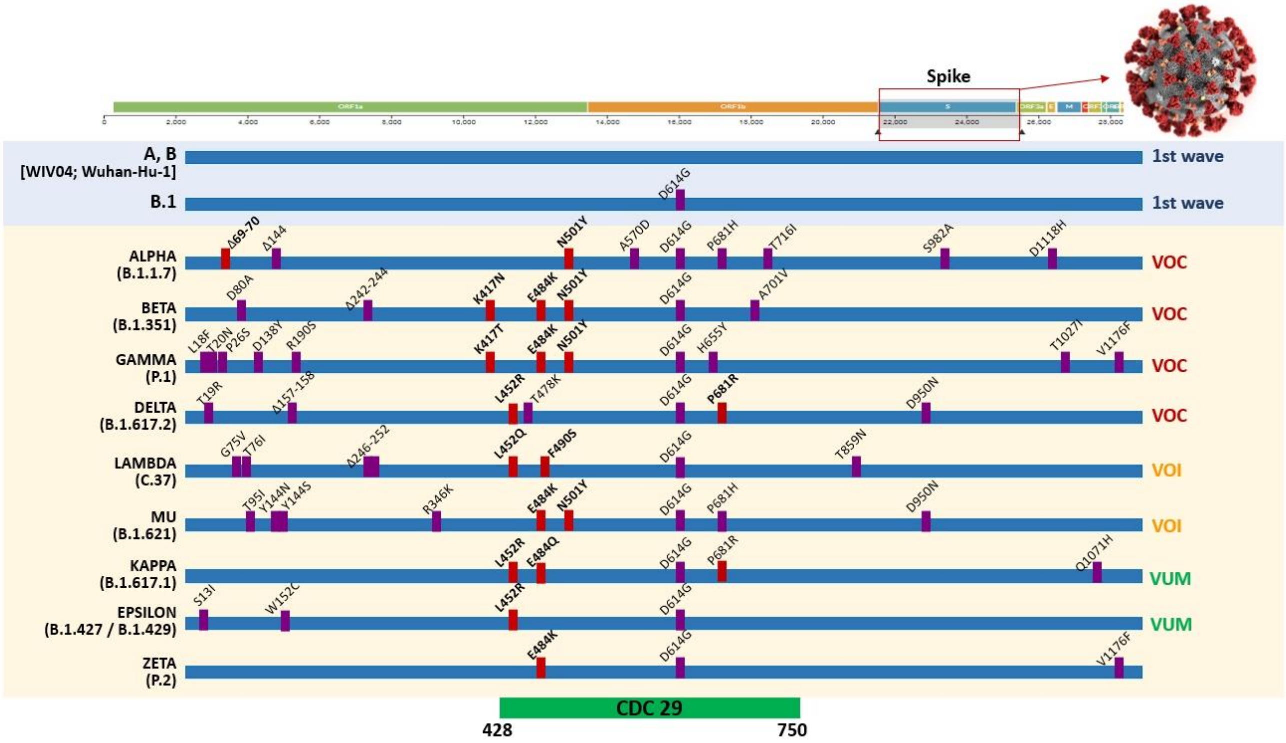

Figure S1. Representation of the amino acid sequence and changes in the Spike protein of SARS-CoV-2 for lineages A, B, B.1 (first wave) and variants of epidemiological interest, including variants of concern (VOC), variants of interest (VOI), and variants under monitoring (VUM). Changes indicated in red correspond to those with the greatest potential impact on viral biology or neutralization by antibodies. The location of CDC fragment 29 (codons 428 to 750) used for active surveillance of variants is indicated.

Supplementary Tables

Table S1. Cumulative cases analyzed in this work from different regions of Argentina and their classification as non-VOC/VOI, variants or mutations of epidemiological interest.

Sampling by Region	Period	Alpha	Gamma	Delta	Lambda	Mu	E484K	others	Non-VOC/VOI	Total
**Samples collected at random**		**286**	**2,042**	**103**	**1,014**	**2**	**142**	**47**	**996**	**4,632**
**MABA**	**2020-10-26 to 2021-10-09**	**152**	**823**	**81**	**668**	**2**	**113**	**27**	**759**	**2,625**
CABA	2020-11-01 to 2021-10-09	74	342	54	308	2	65	13	420	1,278
North GBA	2020-12-21 to 2021-09-22	7	41	3	23		5	2	33	114
West GBA	2020-11-28 to 2021-10-07	49	181	17	109		16	4	106	482
South GBA	2020-10-26 to 2021-10-09	17	130	4	183		27	5	167	533
GLP	2020-11-27 to 2021-10-06	3	127	3	41			2	31	207
Other	2021-01-13 to 2021-09-07	2	2		4			1	1	10
No data	2021-02-14								1	1
**Chaco**	2021-04-01 to 2021-10-08	**1**	**38**		**18**		**2**	**1**	**13**	**73**
**Entre Ríos**	2021-04-05 to 2021-09-09	**11**	**36**		**12**				**3**	**62**
**La Pampa**	2021-05-16 to 2021-05-22	**2**	**30**		**6**					**38**
**San Luis**	2021-04-12 to 2021-04-17	**3**	**14**		**3**				**9**	**29**
**Santa Fe**	2020-12-11 to 2021-10-12	**42**	**626**	**15**	**97**		**1**	**5**	**58**	**844**
**Córdoba**	2020-11-03 to 2021-08-27		**22**		**13**		**2**	**4**	**41**	**82**
**Neuquén**	2021-01-03 to 2021-10-12	**8**	**221**	**4**	**37**			**7**	**27**	**304**
**Buenos Aires (outside MABA)**	2020-11-27 to 2021-10-06	**67**	**231**	**3**	**160**		**24**	**3**	**78**	**566**
**Río Negro**	2020-12-21 to 2021-05-10		**1**						**8**	**9**
**Epidemiological link**		**4**	**24**	**5**	**1**					**34**
**MABA**	2021-02-24 to 2021-09-10	4	9	5	1					19
**Mendoza**	2021-03-22 to 2021-04-08		15							15
**Travel history**		**12**	**23**	**4**	**3**	**1**	**1**		**12**	**56**
**MABA**	2020-12-21 to 2021-10-05	4	6	1	1	1	1		4	18
**Santa Fe**	2021-08-09 to 2021-09-06		1	1						2
**Córdoba**	2021-01-25 to 2021-05-06	6	8		2				2	18
**Neuquén**	2021-01-03 to 2021-06-12	1	7						6	14
**Buenos Aires (outside MABA)**	2021-01-20 to 2021-09-02	1	1	1						3
**Mendoza**	2021-08-30			1						1
**Special selection***	**2021-03-26 to 2021-10-12**	**5**	**34**	**52**	**37**	**1**				**129**
**TOTAL**		**307**	**2,123**	**164**	**1,055**	**3**	**144**	**47**	**991**	**4,851**

* Correspond to cases selected to confirm the identification of variants by prior analysis of variant-specific RT-PCR or to surveillance cases in the vaccinated population. CABA: City of Buenos Aires. GBA: Great Buenos Aires. GLP: Great La Plata. MABA: Metropolitan Area of Buenos Aires. PBA: province of Buenos Aires.

Table S2. Frequency of cases classified as no VOC/VOI, variants or mutations of epidemiological interest by epidemiological week (EW) 2021 in the Metropolitan Area of Buenos Aires (MABA) [1].

**EW**	**Alpha**			**Gamma**			**Delta**			**Lambda**			**Mu**			**E484K[3]**			**Other[4]**			**Non-VOC/VOI**		
	**Freq (%)**	**95%CI[2]**		**Freq (%)**	**95%CI[2]**		**Freq (%)**	**95%CI[2]**		**Freq (%)**	**95%CI[2]**		**Freq (%)**	**95%CI[2]**		**Freq (%)**	**95%CI[2]**		**Freq (%)**	**95%CI[2]**		**Freq (%)**	**95%CI[2]**	
**until 53**																**3.0**	1.5	6.0	**0.4**	0.0	2.4	**96.6**	93.4	98.3
**01-02**																**3.7**	1.6	8.3	**1.5**	0.3	5.2	**94.9**	89.8	97.5
**03-04**										**1.5**	0.1	7.9				**14.7**	8.2	25.0	**1.5**	0.1	7.9	**82.4**	71.6	89.6
**05-06**	**1.8**	0.1	9.3							**1.8**	0.1	9.3				**21.1**	12.5	33.3	**3.5**	0.6	11.9	**71.9**	59.2	81.9
**07-08**	**0.8**	0.0	4.5							**9.9**	5.8	16.5				**15.7**	10.3	23.2	**0.8**	0.0	4.5	**72.7**	64.2	79.9
**09-10**	**6.4**	3.8	10.6	1.0	0.2	3.5				**17.2**	12.7	23.0				**12.3**	8.5	17.5	**4.4**	2.3	8.2	**58.6**	51.7	65.2
**11-12**	**13.6**	9.2	19.6	9.5	5.9	14.8				**28.4**	22.1	35.6				**14.8**	10.2	20.9	**2.4**	0.9	5.9	**31.4**	24.8	38.7
**13-14**	**10.6**	7.1	15.3	33.9	28.0	40.5				**39.9**	33.6	46.5				**1.4**	0.4	4.0	**1.4**	0.4	4.0	**12.8**	9.0	17.9
**15-16**	**15.5**	11.2	21.0	38.6	32.3	45.4				**37.7**	31.4	44.5				**2.4**	1.0	5.5				**5.8**	3.3	9.9
**17-18**	**9.4**	5.4	15.7	38.3	30.3	46.9				**46.9**	38.4	55.5				**0.8**	0.0	4.3	**0.8**	0.0	4.3	**3.9**	1.7	8.8
**19-20**	**9.0**	4.8	16.2	41.0	31.9	50.8				**48.0**	38.5	57.7				**1.0**	0.1	5.4				**1.0**	0.1	5.4
**21-22**	**12.0**	7.5	18.6	51.9	43.5	60.2				**35.3**	27.7	43.8							**0.8**	0.0	4.1			
**23-24**	**4.5**	1.9	10.1	64.0	54.7	72.3				**30.6**	22.8	39.7										**0.9**	0.0	4.9
**25-26**	**3.0**	0.8	8.5	58.0	48.2	67.2				**39.0**	30.0	48.8												
**27-28**	**1.7**	0.3	6.0	61.9	52.9	70.1				**35.6**	27.5	44.6	**0.8**	0.0	4.6									
**29-30**	**6.4**	3.1	12.7	**65.1**	55.8	73.4				**27.5**	20.0	36.6							**0.9**	0.0	5.0			
**31-32**				**68.9**	58.7	77.5				**30.0**	21.5	40.1							**1.1**	0.1	6.0			
**33-34**	**2.0**	0.4	7.1	**64.6**	54.8	73.4	**5.1**	2.2	11.3	**27.3**	19.5	36.8	**1.0**	0.1	5.5									
**35-36**	**2.9**	0.8	8.1	**54.3**	44.8	63.5	**12.4**	7.4	20.0	**30.5**	22.5	39.8												
**37-38**				**42.2**	30.9	54.4	**37.5**	26.7	49.7	**20.3**	12.3	31.7												
**39-40**				**16.4**	8.9	28.3	**70.9**	57.9	81.2	**12.7**	6.3	24.0												

[1] Only the cases from the MABA region that were randomly collected and did not present travel history or close contact with travelers are included; in cases with a known epidemiological link between samples, only one was included to estimate frequencies.[2] The 95% confidence intervals (95% CI) were estimated with the Wilson / Brown method, implemented in the Graph Pad Prism v.9.2 program (San Diego, California, United States, www.graphpad.com).
[3] Includes detections of the E484K mutation that do not belong to sequences with the characteristic combination of mutations of Gamma or Beta variants.
[4] Includes detections of the L452R mutation that do not belong to sequences with the characteristic combination of mutations of Delta or Kappa variants.

Table S3

We gratefully acknowledge the following Authors from the Originating laboratories responsible for obtaining the specimens, as well as the Submitting laboratories where the genome data were generated and shared via GISAID, on which this research is based.

All Submitters of data may be contacted directly via www.gisaid.org

Authors are sorted alphabetically.

Accession ID	Originating Laboratory	Submitting Laboratory	Authors
EPI_ISL_3278255, EPI_ISL_3278256, EPI_ISL_3278257, EPI_ISL_3278258, EPI_ISL_3278259, EPI_ISL_3278260, EPI_ISL_3278261, EPI_ISL_3278262, EPI_ISL_3278263, EPI_ISL_3278264, EPI_ISL_5656719, EPI_ISL_5656720, EPI_ISL_5656721, EPI_ISL_5656722, EPI_ISL_5656723, EPI_ISL_5656724, EPI_ISL_5656725, EPI_ISL_5656861, EPI_ISL_5656862, EPI_ISL_5656863, EPI_ISL_5656864, EPI_ISL_5656865, EPI_ISL_5656866, EPI_ISL_5656867, EPI_ISL_5656868, EPI_ISL_5656869, EPI_ISL_5656973, EPI_ISL_5656974, EPI_ISL_5656975, EPI_ISL_5657263, EPI_ISL_5657622, EPI_ISL_5657627, EPI_ISL_5657628, EPI_ISL_5657635, EPI_ISL_5657636			
see above	Centro Provincial de Referencia VIH/SIDA y Hepatitis Virales – Instituto Biológico Dr Tomás Perón	Área de Secuenciación del Laboratorio de Virología del Hospital de Niños Dr. Ricardo Gutierrez on behalf of 'Proyecto Argentino Interinstitucional de genomica de SARS-CoV-2' (PAIS Consortium)	Acuña; Aldana Gatti; Aldana Nardone; Alexay; Camila Real; Carolina Talavera; D; Daniela Sanchez; Felipe Szymanowski; Francisco Nogueira Laspuir; Goya; Karen Nicole Fresina; LE; Lucrecia Carlos; Lusso; M; MI; Magali Sanchez; Maria Valle; Marisa Corazza; María Eugenia Suarez; Matías Romanko; Nabaes Jodar; Natale; Rosario Dehaut; S; Samanta Ten Huver; Silvia González; Sol Pagliari; Susana Gimenez; Valeria Gutierrez; Valinotto; Vanina Dolcini; Viegas
EPI_ISL_1395463, EPI_ISL_2449251, EPI_ISL_2449252, EPI_ISL_2449253, EPI_ISL_2449264, EPI_ISL_2449282, EPI_ISL_2449283, EPI_ISL_2449284, EPI_ISL_2449285, EPI_ISL_2449296, EPI_ISL_2449297, EPI_ISL_2449298, EPI_ISL_2449299, EPI_ISL_2449334, EPI_ISL_2449353			
see above	Centro de Investigaciones Básicas y Aplicadas, UNNOBA	Área de Secuenciación del Laboratorio de Virología del Hospital de Niños Dr. Ricardo Gutierrez on behalf of 'Proyecto Argentino Interinstitucional de genomica de SARS-CoV-2' (PAIS Consortium)	A; Acuña; Alaniz; Alexay; Bagnis; Balbi; Barbero; Bioq. Lucía Romano; Bonadeo; Brandone; C; CH; Cassarini; Castro; Chimento; Cristina; D; Demarchi; Dr. Rodrigo Hernández del Pin; Dra. Alejandra Brandone y Dra. Natalí Bagnis; Dra. Carolina Cristina; Dra. Fiorella Spinelli; Dra. Gianina Demarchi; Dra. Ina Sevic; Dra. Laura Alaniz; Dra. Laura Palumbo; Dra. María Gracia Balbi; Dra. Virginia Pasquinelli; Español; Est. Lorenzo Morro; F; Fernández; G; Goya; Hernández del Pin; I; Icardi; L; LE; Lic. Agustina Chimento; Lic. Alejandra Fernández; Lic. Alejandro Moroni; Lic. Angela Barbero y Lic. Laureano Español; Lic. Antonella Icardi; Lic. Chiara Cassarini; Lic. Daiana Vitale; Lic. Micaela Castro; Lic. Nadia Bonadeo; Lic. Paolo Rosales; Lic. Sofia Perrone; Lic. Sofia Valla; Lusso; M; MG; MI; Menite; Moroni; Morro; N; Nabaes Jodar; Natale; P; Palumbo; Pasquinelli; Perrone; R; Romano; Rosales; S; Sevic; Spinelli; Tc. Gastón Villafañe; Tc. Natalia Menite; V; Valinotto; Valla; Viegas, M.; Villafañe; Vitale
EPI_ISL_3759752	Centro de Salud VARVARCO	Laboratorio Central Mg. Luis Alfredo Pianciola	C Pintos; C Rastellini; C Ziehm; J Ousset; L Pianciola; M Fernandez; M Mazzeo; M Viegas.
EPI_ISL_3235791, EPI_ISL_3235792, EPI_ISL_3235793, EPI_ISL_3235794, EPI_ISL_3235795, EPI_ISL_3235796, EPI_ISL_3235797, EPI_ISL_3235798, EPI_ISL_3235799, EPI_ISL_3235800, EPI_ISL_3235801, EPI_ISL_3235802, EPI_ISL_3235803, EPI_ISL_3235804, EPI_ISL_3235805, EPI_ISL_3235806, EPI_ISL_3235807, EPI_ISL_3235808, EPI_ISL_3235809, EPI_ISL_3235810, EPI_ISL_3235811, EPI_ISL_3235812, EPI_ISL_3235843, EPI_ISL_3235844, EPI_ISL_3235845, EPI_ISL_3235846, EPI_ISL_3235847, EPI_ISL_3235848, EPI_ISL_3235849, EPI_ISL_3235850, EPI_ISL_3235851, EPI_ISL_3235852, EPI_ISL_3235853, EPI_ISL_3235854, EPI_ISL_3235855, EPI_ISL_3235856, EPI_ISL_3235857, EPI_ISL_3235858, EPI_ISL_3235859, EPI_ISL_3235860, EPI_ISL_3235861, EPI_ISL_3235862, EPI_ISL_3235863, EPI_ISL_3235864, EPI_ISL_3235865, EPI_ISL_3235866, EPI_ISL_3235896, EPI_ISL_3235897, EPI_ISL_3235898, EPI_ISL_3235899, EPI_ISL_3235900, EPI_ISL_3235901, EPI_ISL_3235902, EPI_ISL_3235903, EPI_ISL_3235904, EPI_ISL_3235905, EPI_ISL_3235906, EPI_ISL_3235907, EPI_ISL_3235908, EPI_ISL_3235909, EPI_ISL_3235910, EPI_ISL_3235911, EPI_ISL_3235912, EPI_ISL_3235913, EPI_ISL_3235914, EPI_ISL_3235915, EPI_ISL_3235916, EPI_ISL_3235917, EPI_ISL_3235918, EPI_ISL_3235949, EPI_ISL_3235950, EPI_ISL_3235951, EPI_ISL_3235952, EPI_ISL_3235953, EPI_ISL_3235954, EPI_ISL_3235955, EPI_ISL_3235956, EPI_ISL_3235957, EPI_ISL_3235958, EPI_ISL_3235959, EPI_ISL_3235960, EPI_ISL_3235961, EPI_ISL_3235962, EPI_ISL_3235963, EPI_ISL_3235964, EPI_ISL_3235965, EPI_ISL_3235966, EPI_ISL_3235967, EPI_ISL_3235968, EPI_ISL_3235969, EPI_ISL_3235970, EPI_ISL_3235971, EPI_ISL_3236047, EPI_ISL_3236048, EPI_ISL_3236049, EPI_ISL_3236050, EPI_ISL_3236051, EPI_ISL_3236052, EPI_ISL_3236053, EPI_ISL_3236054, EPI_ISL_3236055, EPI_ISL_3236056, EPI_ISL_3236057, EPI_ISL_3236058, EPI_ISL_3236059, EPI_ISL_3236060, EPI_ISL_3236061, EPI_ISL_3236062, EPI_ISL_3236063, EPI_ISL_3236064, EPI_ISL_3236065, EPI_ISL_3236066, EPI_ISL_3236067, EPI_ISL_3259381, EPI_ISL_3259382, EPI_ISL_3259383, EPI_ISL_3259384, EPI_ISL_3259385, EPI_ISL_5335392, EPI_ISL_5335393, EPI_ISL_5335394, EPI_ISL_5335395, EPI_ISL_5335396, EPI_ISL_5335397, EPI_ISL_5335398, EPI_ISL_5335399, EPI_ISL_5335400, EPI_ISL_5335401, EPI_ISL_5335402, EPI_ISL_5335403, EPI_ISL_5335404, EPI_ISL_5335405, EPI_ISL_5335406, EPI_ISL_5335407, EPI_ISL_5335408, EPI_ISL_5335409, EPI_ISL_5335410, EPI_ISL_5335411, EPI_ISL_5335465, EPI_ISL_5335466, EPI_ISL_5335467, EPI_ISL_5335468, EPI_ISL_5335469, EPI_ISL_5335470, EPI_ISL_5335471, EPI_ISL_5335472, EPI_ISL_5335473, EPI_ISL_5335474, EPI_ISL_5335475, EPI_ISL_5335476, EPI_ISL_5335477, EPI_ISL_5335478, EPI_ISL_5335479, EPI_ISL_5335480, EPI_ISL_5335481, EPI_ISL_5335482, EPI_ISL_5335483, EPI_ISL_5335484, EPI_ISL_5335485, EPI_ISL_5335531, EPI_ISL_5335532, EPI_ISL_5335533, EPI_ISL_5335534, EPI_ISL_5335535, EPI_ISL_5335536, EPI_ISL_5335537, EPI_ISL_5335538, EPI_ISL_5335539, EPI_ISL_5335540, EPI_ISL_5335541, EPI_ISL_5335542, EPI_ISL_5335543, EPI_ISL_5335544, EPI_ISL_5335545, EPI_ISL_5335546, EPI_ISL_5335547, EPI_ISL_5335548, EPI_ISL_5335549, EPI_ISL_5335550, EPI_ISL_5335551, EPI_ISL_5335552, EPI_ISL_5335553, EPI_ISL_5335554, EPI_ISL_5335587, EPI_ISL_5335589, EPI_ISL_5335590, EPI_ISL_5335591, EPI_ISL_5335592, EPI_ISL_5335593, EPI_ISL_5335594, EPI_ISL_5335595, EPI_ISL_5335596, EPI_ISL_5335597, EPI_ISL_5335598, EPI_ISL_5335599, EPI_ISL_5335600, EPI_ISL_5335601, EPI_ISL_5335602, EPI_ISL_5335603, EPI_ISL_5335604, EPI_ISL_5335605, EPI_ISL_5335606, EPI_ISL_5335607, EPI_ISL_5335608, EPI_ISL_5335609, EPI_ISL_5335624, EPI_ISL_5335625, EPI_ISL_5335626, EPI_ISL_5335627, EPI_ISL_5335628, EPI_ISL_5335629, EPI_ISL_5335630, EPI_ISL_5335631, EPI_ISL_5335632, EPI_ISL_5335633, EPI_ISL_5335634, EPI_ISL_5335635, EPI_ISL_5335636, EPI_ISL_5335637, EPI_ISL_5335641, EPI_ISL_5335642, EPI_ISL_5335643, EPI_ISL_5335644, EPI_ISL_5335645, EPI_ISL_5335646, EPI_ISL_5335647, EPI_ISL_5335654, EPI_ISL_5335655, EPI_ISL_5335661, EPI_ISL_5335662, EPI_ISL_5348009, EPI_ISL_5348010			
see above	Centro de Tecnología en Salud Pública de la Universidad Nacional de Rosario	Laboratorio Mixto de Biotecnología Acuática (LMBA), on behalf of 'Proyecto Argentino Interinstitucional de genomica de SARS-CoV-2' (PAIS Consortium)	Adriana Giri; Agustina Cerri; Ana Cavatorta; Diego Chouhy; Elisa Bolatti; Gastón Viarengo; German Perez; Julian Acosta; Lucia Moriena; María Re; Pablo Casal; Silvia Arranz; Vanina Villanova; Victoria Posner
EPI_ISL_2449455, EPI_ISL_2449495, EPI_ISL_2449509, EPI_ISL_2449528, EPI_ISL_2449529, EPI_ISL_2449551, EPI_ISL_2449552			
see above	Centro diagnóstico de covid-19, CEMET	Área de Secuenciación del Laboratorio de Virología del Hospital de Niños Dr. Ricardo Gutierrez on behalf of 'Proyecto Argentino Interinstitucional de genomica de SARS-CoV-2' (PAIS Consortium)	Acuña; Alexay; D; Goya; LE; Lusso; M; MI; Marilina Rahhal; Martin Zubieta; Nabaes Jodar; Natale; S; Valinotto; Viegas, M.
EPI_ISL_3235733, EPI_ISL_3235734, EPI_ISL_3235735, EPI_ISL_3235736, EPI_ISL_3235737, EPI_ISL_3235738, EPI_ISL_3235739, EPI_ISL_3235740, EPI_ISL_3235741, EPI_ISL_3235742, EPI_ISL_3235743, EPI_ISL_3235744, EPI_ISL_3235745, EPI_ISL_3235746, EPI_ISL_3235747, EPI_ISL_3235748, EPI_ISL_3235749, EPI_ISL_3235750, EPI_ISL_3235751, EPI_ISL_3235752, EPI_ISL_3235753, EPI_ISL_3235754, EPI_ISL_3235755, EPI_ISL_3235756, EPI_ISL_3235757, EPI_ISL_3235758, EPI_ISL_3235759, EPI_ISL_3235760, EPI_ISL_3235761, EPI_ISL_3235762, EPI_ISL_3235763, EPI_ISL_3235764, EPI_ISL_3235765, EPI_ISL_3235766, EPI_ISL_3235767, EPI_ISL_3235768, EPI_ISL_3235769, EPI_ISL_3235770, EPI_ISL_3235771, EPI_ISL_3235772, EPI_ISL_3235773, EPI_ISL_3235774, EPI_ISL_3235775, EPI_ISL_3235776, EPI_ISL_3235777, EPI_ISL_3235778, EPI_ISL_3235779, EPI_ISL_3235780, EPI_ISL_3235781, EPI_ISL_3235782, EPI_ISL_3235783, EPI_ISL_3235784, EPI_ISL_3235785, EPI_ISL_3235786, EPI_ISL_3235787, EPI_ISL_3235788, EPI_ISL_3235789, EPI_ISL_3235790, EPI_ISL_3235813, EPI_ISL_3235814, EPI_ISL_3235815, EPI_ISL_3235816, EPI_ISL_3235817, EPI_ISL_3235818, EPI_ISL_3235819, EPI_ISL_3235820, EPI_ISL_3235821, EPI_ISL_3235822, EPI_ISL_3235823, EPI_ISL_3235824, EPI_ISL_3235825, EPI_ISL_3235826, EPI_ISL_3235827, EPI_ISL_3235828, EPI_ISL_3235829, EPI_ISL_3235830, EPI_ISL_3235831, EPI_ISL_3235832, EPI_ISL_3235833, EPI_ISL_3235834, EPI_ISL_3235835, EPI_ISL_3235836, EPI_ISL_3235837, EPI_ISL_3235838, EPI_ISL_3235839, EPI_ISL_3235840, EPI_ISL_3235841, EPI_ISL_3235842, EPI_ISL_3235867, EPI_ISL_3235868, EPI_ISL_3235869, EPI_ISL_3235870, EPI_ISL_3235871, EPI_ISL_3235872, EPI_ISL_3235873, EPI_ISL_3235874, EPI_ISL_3235875, EPI_ISL_3235876, EPI_ISL_3235877, EPI_ISL_3235878, EPI_ISL_3235879, EPI_ISL_3235880, EPI_ISL_3235881, EPI_ISL_3235882, EPI_ISL_3235883, EPI_ISL_3235884, EPI_ISL_3235885, EPI_ISL_3235886, EPI_ISL_3235887, EPI_ISL_3235888, EPI_ISL_3235889, EPI_ISL_3235890, EPI_ISL_3235891, EPI_ISL_3235892, EPI_ISL_3235893, EPI_ISL_3235894, EPI_ISL_3235895, EPI_ISL_3235919, EPI_ISL_3235920, EPI_ISL_3235921, EPI_ISL_3235922, EPI_ISL_3235923, EPI_ISL_3235924, EPI_ISL_3235925, EPI_ISL_3235926, EPI_ISL_3235927, EPI_ISL_3235928, EPI_ISL_3235929, EPI_ISL_3235930, EPI_ISL_3235931, EPI_ISL_3235932, EPI_ISL_3235933, EPI_ISL_3235934, EPI_ISL_3235935, EPI_ISL_3235936, EPI_ISL_3235937, EPI_ISL_3235938, EPI_ISL_3235939, EPI_ISL_3235940, EPI_ISL_3235941, EPI_ISL_3235942, EPI_ISL_3235943, EPI_ISL_3235944, EPI_ISL_3235945, EPI_ISL_3235946, EPI_ISL_3235947, EPI_ISL_3235948, EPI_ISL_3235972, EPI_ISL_3235973, EPI_ISL_3235974, EPI_ISL_3235975, EPI_ISL_3235976, EPI_ISL_3235977, EPI_ISL_3235978, EPI_ISL_3235979, EPI_ISL_3235980, EPI_ISL_3235981, EPI_ISL_3235982, EPI_ISL_3235983, EPI_ISL_3235984, EPI_ISL_3235985, EPI_ISL_3235986, EPI_ISL_3235987, EPI_ISL_3235988, EPI_ISL_3235989, EPI_ISL_3235990, EPI_ISL_3235991, EPI_ISL_3235992, EPI_ISL_3235993, EPI_ISL_3235994, EPI_ISL_3235995, EPI_ISL_3235996, EPI_ISL_3235997, EPI_ISL_3235998, EPI_ISL_3235999, EPI_ISL_3236000, EPI_ISL_3236001, EPI_ISL_3236002, EPI_ISL_3236003, EPI_ISL_3236004, EPI_ISL_3236005, EPI_ISL_3236006, EPI_ISL_3236007, EPI_ISL_3236008, EPI_ISL_3236009, EPI_ISL_3236010, EPI_ISL_3236011, EPI_ISL_3236012, EPI_ISL_3236013, EPI_ISL_3236014, EPI_ISL_3236015, EPI_ISL_3236016, EPI_ISL_3236017, EPI_ISL_3236018, EPI_ISL_3236019, EPI_ISL_3236020, EPI_ISL_3236021, EPI_ISL_3236022, EPI_ISL_3236023, EPI_ISL_3236024, EPI_ISL_3236025, EPI_ISL_3236026, EPI_ISL_3236027, EPI_ISL_3236028, EPI_ISL_3236029, EPI_ISL_3236030, EPI_ISL_3236031, EPI_ISL_3236032, EPI_ISL_3236033, EPI_ISL_3236034, EPI_ISL_3236035, EPI_ISL_3236036, EPI_ISL_3236037, EPI_ISL_3236038, EPI_ISL_3236039, EPI_ISL_3236040, EPI_ISL_3236041, EPI_ISL_3236042, EPI_ISL_3236043, EPI_ISL_3236044, EPI_ISL_3236045, EPI_ISL_3236046, EPI_ISL_3236068, EPI_ISL_3236069, EPI_ISL_3236070, EPI_ISL_3236071, EPI_ISL_3236072, EPI_ISL_3236073, EPI_ISL_3236074, EPI_ISL_3236075, EPI_ISL_3236076, EPI_ISL_3236077, EPI_ISL_3236078, EPI_ISL_3236079, EPI_ISL_3236080, EPI_ISL_3236081, EPI_ISL_3236082, EPI_ISL_3236083, EPI_ISL_3236084, EPI_ISL_3236085, EPI_ISL_3236086, EPI_ISL_3236087, EPI_ISL_3236088, EPI_ISL_3236089, EPI_ISL_3236090, EPI_ISL_3236091, EPI_ISL_3236092, EPI_ISL_3236093, EPI_ISL_3236094, EPI_ISL_3236095, EPI_ISL_3236096, EPI_ISL_3236097, EPI_ISL_3236098, EPI_ISL_3236099, EPI_ISL_3236100, EPI_ISL_3236101, EPI_ISL_3259386, EPI_ISL_5335412, EPI_ISL_5335413, EPI_ISL_5335414, EPI_ISL_5335415, EPI_ISL_5335416, EPI_ISL_5335417, EPI_ISL_5335418, EPI_ISL_5335419, EPI_ISL_5335420, EPI_ISL_5335421, EPI_ISL_5335422, EPI_ISL_5335423, EPI_ISL_5335424, EPI_ISL_5335425, EPI_ISL_5335426, EPI_ISL_5335427, EPI_ISL_5335428, EPI_ISL_5335429, EPI_ISL_5335430, EPI_ISL_5335431, EPI_ISL_5335432, EPI_ISL_5335433, EPI_ISL_5335434, EPI_ISL_5335435, EPI_ISL_5335436, EPI_ISL_5335437, EPI_ISL_5335438, EPI_ISL_5335439, EPI_ISL_5335440, EPI_ISL_5335441, EPI_ISL_5335442, EPI_ISL_5335443, EPI_ISL_5335444, EPI_ISL_5335445, EPI_ISL_5335446, EPI_ISL_5335447, EPI_ISL_5335448, EPI_ISL_5335449, EPI_ISL_5335450, EPI_ISL_5335451, EPI_ISL_5335452, EPI_ISL_5335453, EPI_ISL_5335454, EPI_ISL_5335455, EPI_ISL_5335456, EPI_ISL_5335457, EPI_ISL_5335458, EPI_ISL_5335459, EPI_ISL_5335460, EPI_ISL_5335461, EPI_ISL_5335462, EPI_ISL_5335463, EPI_ISL_5335464, EPI_ISL_5335486, EPI_ISL_5335487, EPI_ISL_5335492, EPI_ISL_5335493, EPI_ISL_5335494, EPI_ISL_5335495, EPI_ISL_5335496, EPI_ISL_5335497, EPI_ISL_5335498, EPI_ISL_5335499, EPI_ISL_5335500, EPI_ISL_5335501, EPI_ISL_5335502, EPI_ISL_5335503, EPI_ISL_5335504, EPI_ISL_5335505, EPI_ISL_5335506, EPI_ISL_5335507, EPI_ISL_5335508, EPI_ISL_5335509, EPI_ISL_5335510, EPI_ISL_5335511, EPI_ISL_5335512, EPI_ISL_5335513, EPI_ISL_5335514, EPI_ISL_5335515, EPI_ISL_5335516, EPI_ISL_5335517, EPI_ISL_5335518, EPI_ISL_5335519, EPI_ISL_5335520, EPI_ISL_5335521, EPI_ISL_5335522, EPI_ISL_5335523, EPI_ISL_5335524, EPI_ISL_5335525, EPI_ISL_5335526, EPI_ISL_5335527, EPI_ISL_5335528, EPI_ISL_5335529, EPI_ISL_5335530, EPI_ISL_5335555, EPI_ISL_5335556, EPI_ISL_5335557, EPI_ISL_5335558, EPI_ISL_5335559, EPI_ISL_5335560, EPI_ISL_5335561, EPI_ISL_5335562, EPI_ISL_5335563, EPI_ISL_5335564, EPI_ISL_5335565, EPI_ISL_5335566, EPI_ISL_5335567, EPI_ISL_5335568, EPI_ISL_5335569, EPI_ISL_5335570, EPI_ISL_5335571, EPI_ISL_5335572, EPI_ISL_5335573, EPI_ISL_5335574, EPI_ISL_5335575, EPI_ISL_5335576, EPI_ISL_5335577, EPI_ISL_5335578, EPI_ISL_5335579, EPI_ISL_5335580, EPI_ISL_5335581, EPI_ISL_5335582, EPI_ISL_5335583, EPI_ISL_5335584, EPI_ISL_5335585, EPI_ISL_5335586, EPI_ISL_5335610, EPI_ISL_5335611, EPI_ISL_5335612, EPI_ISL_5335613, EPI_ISL_5335614, EPI_ISL_5335615, EPI_ISL_5335616, EPI_ISL_5335617, EPI_ISL_5335618, EPI_ISL_5335619, EPI_ISL_5335620, EPI_ISL_5335621, EPI_ISL_5335622, EPI_ISL_5335623, EPI_ISL_5335638, EPI_ISL_5335639, EPI_ISL_5335648, EPI_ISL_5335649, EPI_ISL_5335650, EPI_ISL_5335651, EPI_ISL_5335652, EPI_ISL_5335653, EPI_ISL_5335656, EPI_ISL_5335657, EPI_ISL_5335658, EPI_ISL_5335659, EPI_ISL_5335660, EPI_ISL_5335663, EPI_ISL_5335664, EPI_ISL_5335665			
see above	Cibic Laboratorios	Cibic Laboratorios on behalf of 'Proyecto Argentino Interinstitucional de genomica de SARS-CoV-2' (PAIS Consortium)	Analía Seravalle; Clara Fay; Fabian Fay; Javier Sfalcin; Mariana Galizzi; Martina Fay
EPI_ISL_2348733	Clinica CMIC	Laboratorio Central Mg. Luis Alfredo Pianciola on behalf of 'Proyecto Argentino Interinstitucional de genomica de SARS-CoV-2' (PAIS Consortium)	C Pintos; C Rastellini; C Ziehm; J Ousset; L Pianciola; M Fernandez; M Mazzeo
EPI_ISL_2348737	Clinica Chapelco	Laboratorio Central Mg. Luis Alfredo Pianciola on behalf of 'Proyecto Argentino Interinstitucional de genomica de SARS-CoV-2' (PAIS Consortium)	C Pintos; C Rastellini; C Ziehm; J Ousset; L Pianciola; M Fernandez; M Mazzeo
EPI_ISL_3568631	Cs. Almafuerte	Laboratorio Central Mg. Luis Alfredo Pianciola	C Pintos; C Rastellini; C Ziehm; J Ousset; L Pianciola; M Fernandez; M Mazzeo; M Viegas.
EPI_ISL_2449456, EPI_ISL_2449457, EPI_ISL_2449458, EPI_ISL_2449472, EPI_ISL_2449473, EPI_ISL_2449474, EPI_ISL_2449496, EPI_ISL_2449497, EPI_ISL_2449498, EPI_ISL_2449512, EPI_ISL_2449553, EPI_ISL_2449554, EPI_ISL_2449581			
see above	Departamento de Biología y genética molecular, IACA Laboratorios.	Área de Secuenciación del Laboratorio de Virología del Hospital de Niños Dr. Ricardo Gutierrez on behalf of 'Proyecto Argentino Interinstitucional de genomica de SARS-CoV-2' (PAIS Consortium)	A; Acuña; Alexay; D; E; Goya; LE; Lusso; M; MI; Masciovecchio MV; Nabaes Jodar; Natale; S; Streitenberger ER; Suárez; Tittarelli; Valinotto; Viegas, M.
EPI_ISL_3277889, EPI_ISL_3277899, EPI_ISL_3277904, EPI_ISL_3278156, EPI_ISL_3278157, EPI_ISL_3278158, EPI_ISL_3278159, EPI_ISL_3278160, EPI_ISL_3278161, EPI_ISL_3278162, EPI_ISL_3278163, EPI_ISL_3278164, EPI_ISL_3278165, EPI_ISL_3278166, EPI_ISL_3278167, EPI_ISL_3278168, EPI_ISL_3278169, EPI_ISL_3278170, EPI_ISL_3278171, EPI_ISL_3278172, EPI_ISL_3278173			
see above	HIGA SAN ROQUE GONNET	Área de Secuenciación del Laboratorio de Virología del Hospital de Niños Dr. Ricardo Gutierrez on behalf of 'Proyecto Argentino Interinstitucional de genomica de SARS-CoV-2' (PAIS Consortium)	Acuña; Alexay; D; Evelyn De la Rubia; Goya; LE; Lusso; M; MI; Nabaes Jodar; Natale; Paula Carassi; Rosana Toro; S; Valinotto; Viegas
EPI_ISL_3759748	Hospital ALUMINE	Laboratorio Central Mg. Luis Alfredo Pianciola	C Pintos; C Rastellini; C Ziehm; J Ousset; L Pianciola; M Fernandez; M Mazzeo; M Viegas.
EPI_ISL_3759739, EPI_ISL_3759753	Hospital ANDACOLLO	Laboratorio Central Mg. Luis Alfredo Pianciola	C Pintos; C Rastellini; C Ziehm; J Ousset; L Pianciola; M Fernandez; M Mazzeo; M Viegas.
EPI_ISL_1394915, EPI_ISL_1394929, EPI_ISL_5657010, EPI_ISL_5657088, EPI_ISL_5657089, EPI_ISL_5657090, EPI_ISL_5657091, EPI_ISL_5657092, EPI_ISL_5657093, EPI_ISL_5657094, EPI_ISL_5657095, EPI_ISL_5657096, EPI_ISL_5657141, EPI_ISL_5657142, EPI_ISL_5657143, EPI_ISL_5657144, EPI_ISL_5657145, EPI_ISL_5657146, EPI_ISL_5657147, EPI_ISL_5657148, EPI_ISL_5657149, EPI_ISL_5657150, EPI_ISL_5657224, EPI_ISL_5657225, EPI_ISL_5657226, EPI_ISL_5657227, EPI_ISL_5657228, EPI_ISL_5657229, EPI_ISL_5657230, EPI_ISL_5657231, EPI_ISL_5657232, EPI_ISL_5657236, EPI_ISL_5657237, EPI_ISL_5657238, EPI_ISL_5657239, EPI_ISL_5657240, EPI_ISL_5657241, EPI_ISL_5657405, EPI_ISL_5657412, EPI_ISL_5657413, EPI_ISL_5657481, EPI_ISL_5657489, EPI_ISL_5657490, EPI_ISL_5657494, EPI_ISL_5657495, EPI_ISL_5657499, EPI_ISL_5657557, EPI_ISL_5657559, EPI_ISL_5657560, EPI_ISL_5657567, EPI_ISL_5657568, EPI_ISL_5657577, EPI_ISL_5657580, EPI_ISL_5657581, EPI_ISL_5657796, EPI_ISL_5657800, EPI_ISL_5657801, EPI_ISL_5657804, EPI_ISL_5657807, EPI_ISL_5657808, EPI_ISL_5657813, EPI_ISL_5657814, EPI_ISL_5657822, EPI_ISL_5657827, EPI_ISL_5657828, EPI_ISL_5657832, EPI_ISL_5657833			
see above	Hospital Alemán	Área de Secuenciación del Laboratorio de Virología del Hospital de Niños Dr. Ricardo	Acuña; Alexay; D; Eugenia Ibañez; Goya; LE; Lusso; M; MI; María Paula Della Latta; Nabaes Jodar; Natale; Natalia García Allende; Natalia García allende; S; Valinotto; Viegas; Viegas, M.

		Gutierrez on behalf of 'Proyecto Argentino Interinstitucional de genomica de SARS-CoV-2' (PAIS Consortium)	
EPI_ISL_3568629	Hospital Andacollo	Laboratorio Central Mg. Luis Alfredo Pianciola	C Pintos; C Rastellini; C Ziehm; J Ousset; L Pianciola; M Fernandez; M Mazzeo; M Viegas.
EPI_ISL_3759740	Hospital AÑELO	Laboratorio Central Mg. Luis Alfredo Pianciola	C Pintos; C Rastellini; C Ziehm; J Ousset; L Pianciola; M Fernandez; M Mazzeo; M Viegas.
EPI_ISL_3759742	Hospital BOUQUET ROLDAN	Laboratorio Central Mg. Luis Alfredo Pianciola	C Pintos; C Rastellini; C Ziehm; J Ousset; L Pianciola; M Fernandez; M Mazzeo; M Viegas.
EPI_ISL_3568633	Hospital Buta Ranquil	Laboratorio Central Mg. Luis Alfredo Pianciola	C Pintos; C Rastellini; C Ziehm; J Ousset; L Pianciola; M Fernandez; M Mazzeo; M Viegas.
EPI_ISL_3759749	Hospital CENTENARIO	Laboratorio Central Mg. Luis Alfredo Pianciola	C Pintos; C Rastellini; C Ziehm; J Ousset; L Pianciola; M Fernandez; M Mazzeo; M Viegas.
EPI_ISL_3759760	Hospital CHOS MALAL	Laboratorio Central Mg. Luis Alfredo Pianciola	C Pintos; C Rastellini; C Ziehm; J Ousset; L Pianciola; M Fernandez; M Mazzeo; M Viegas.
EPI_ISL_3759756	Hospital CUTRAL-CO / PLAZA HINCUL	Laboratorio Central Mg. Luis Alfredo Pianciola	C Pintos; C Rastellini; C Ziehm; J Ousset; L Pianciola; M Fernandez; M Mazzeo; M Viegas.
EPI_ISL_3568628	Hospital Cutral Co	Laboratorio Central Mg. Luis Alfredo Pianciola	C Pintos; C Rastellini; C Ziehm; J Ousset; L Pianciola; M Fernandez; M Mazzeo; M Viegas.
EPI_ISL_2348730	Hospital Dr. Horacio Heller	Laboratorio Central Mg. Luis Alfredo Pianciola on behalf of 'Proyecto Argentino Interinstitucional de genomica de SARS-CoV-2' (PAIS Consortium)	C Pintos; C Rastellini; C Ziehm; J Ousset; L Pianciola; M Fernandez; M Mazzeo
EPI_ISL_1395412, EPI_ISL_1395423, EPI_ISL_1395452, EPI_ISL_1395453, EPI_ISL_1395466, EPI_ISL_1395497, EPI_ISL_1395513, EPI_ISL_1395514, EPI_ISL_1395515, EPI_ISL_1395530, EPI_ISL_1395531, EPI_ISL_1395532, EPI_ISL_1395556, EPI_ISL_1395557, EPI_ISL_1395558, EPI_ISL_1395567, EPI_ISL_1395568, EPI_ISL_1395569, EPI_ISL_1395581, EPI_ISL_1395582, EPI_ISL_1395599, EPI_ISL_1395601, EPI_ISL_1395626, EPI_ISL_1395627, EPI_ISL_1395637, EPI_ISL_1395648, EPI_ISL_1395668, EPI_ISL_1395669, EPI_ISL_1395670, EPI_ISL_1395671, EPI_ISL_1395693, EPI_ISL_1395700, EPI_ISL_2449032, EPI_ISL_2449033, EPI_ISL_2449042, EPI_ISL_2449054, EPI_ISL_2449064, EPI_ISL_2449065, EPI_ISL_2449078, EPI_ISL_2449091, EPI_ISL_2449092, EPI_ISL_2449102, EPI_ISL_2449108, EPI_ISL_2449113, EPI_ISL_2449119, EPI_ISL_2449129, EPI_ISL_2449130, EPI_ISL_2449132, EPI_ISL_2449139, EPI_ISL_2449147, EPI_ISL_2449155, EPI_ISL_2449156, EPI_ISL_2449182, EPI_ISL_2449183, EPI_ISL_2449184, EPI_ISL_2449198, EPI_ISL_2449199, EPI_ISL_2449202, EPI_ISL_2449211, EPI_ISL_2449238, EPI_ISL_2449262, EPI_ISL_2449274, EPI_ISL_2449316, EPI_ISL_2449317, EPI_ISL_2449327, EPI_ISL_2449333, EPI_ISL_2449347, EPI_ISL_2449348, EPI_ISL_2449368, EPI_ISL_2449376, EPI_ISL_2449383, EPI_ISL_2449406, EPI_ISL_2449422, EPI_ISL_2449423, EPI_ISL_2449424, EPI_ISL_2449428, EPI_ISL_2449454, EPI_ISL_2449490, EPI_ISL_2449491, EPI_ISL_2449492, EPI_ISL_2449493, EPI_ISL_2449494, EPI_ISL_2449506, EPI_ISL_2449507, EPI_ISL_2449508, EPI_ISL_2449531, EPI_ISL_2449532, EPI_ISL_2449533, EPI_ISL_2449534, EPI_ISL_2449535, EPI_ISL_2449576, EPI_ISL_2449583, EPI_ISL_2449594, EPI_ISL_2449595, EPI_ISL_2449596, EPI_ISL_2449609, EPI_ISL_2449610, EPI_ISL_2449611, EPI_ISL_2449612, EPI_ISL_2449613, EPI_ISL_2449634, EPI_ISL_2449635, EPI_ISL_2449636, EPI_ISL_2449637, EPI_ISL_2449638, EPI_ISL_2449639, EPI_ISL_2449668, EPI_ISL_2449690, EPI_ISL_2449691, EPI_ISL_2449708, EPI_ISL_2449709, EPI_ISL_2449732, EPI_ISL_2449733, EPI_ISL_2449734, EPI_ISL_2449735, EPI_ISL_2449736, EPI_ISL_2449758, EPI_ISL_2449778, EPI_ISL_2449779, EPI_ISL_2449795, EPI_ISL_2449796, EPI_ISL_2449815, EPI_ISL_3277886, EPI_ISL_3277944, EPI_ISL_3277945, EPI_ISL_3277946, EPI_ISL_3277947, EPI_ISL_3277948, EPI_ISL_3277949, EPI_ISL_3277950, EPI_ISL_3277951, EPI_ISL_3277952, EPI_ISL_3277953, EPI_ISL_3277954, EPI_ISL_3277955, EPI_ISL_3277956, EPI_ISL_3278032, EPI_ISL_3278033, EPI_ISL_3278034, EPI_ISL_3278035, EPI_ISL_3278036, EPI_ISL_3278037, EPI_ISL_3278038, EPI_ISL_3278039, EPI_ISL_3278040, EPI_ISL_3278041, EPI_ISL_3278042, EPI_ISL_3278043, EPI_ISL_3278044, EPI_ISL_3278045, EPI_ISL_3278046, EPI_ISL_3278047, EPI_ISL_3278224, EPI_ISL_3278225, EPI_ISL_3278226, EPI_ISL_3278227, EPI_ISL_3278228, EPI_ISL_3278229, EPI_ISL_3278279, EPI_ISL_3278280, EPI_ISL_3278281, EPI_ISL_3278282			
see above	Hospital General de Agudos Dr. Cosme Argerich	Área de Secuenciación del Laboratorio de Virología del Hospital de Niños Dr. Ricardo Gutierrez on behalf of 'Proyecto Argentino Interinstitucional de genomica de SARS-CoV-2' (PAIS Consortium)	Acuña; Alexay; Andrea Fernández; D; Florencia Funez; Florencia Rodríguez; Goya; Jésica Galeano; Karina Polanski; LE; Lusso; M; MI; Marcia Pozzati; Nabaes Jodar; Natale; S; Valinotto; Viegas, M.
EPI_ISL_3759741, EPI_ISL_3759750	Hospital HELLER	Laboratorio Central Mg. Luis Alfredo Pianciola	C Pintos; C Rastellini; C Ziehm; J Ousset; L Pianciola; M Fernandez; M Mazzeo; M Viegas.
EPI_ISL_5658027, EPI_ISL_5658030	Hospital HIGA San Roque Gonnet	Área de Secuenciación del Laboratorio de Virología del Hospital de Niños Dr. Ricardo Gutierrez on behalf of 'Proyecto Argentino Interinstitucional de genomica de SARS-CoV-2' (PAIS Consortium)	Acuña; Alexay; D; Evelyn De la Rubia; Goya; LE; Lusso; M; MI; Nabaes Jodar; Natale; Paula Carassi; Rosana Toro; S; Valinotto; Viegas
EPI_ISL_3759758	Hospital JUNIN DE LOS ANDES	Laboratorio Central Mg. Luis Alfredo Pianciola	C Pintos; C Rastellini; C Ziehm; J Ousset; L Pianciola; M Fernandez; M Mazzeo; M Viegas.
EPI_ISL_3568632	Hospital Junin de los Andes	Laboratorio Central Mg. Luis Alfredo Pianciola	C Pintos; C Rastellini; C Ziehm; J Ousset; L Pianciola; M Fernandez; M Mazzeo; M Viegas.
EPI_ISL_3568630	Hospital Las Ovejas	Laboratorio Central Mg. Luis Alfredo Pianciola	C Pintos; C Rastellini; C Ziehm; J Ousset; L Pianciola; M Fernandez; M Mazzeo; M Viegas.
EPI_ISL_5656990, EPI_ISL_5656991, EPI_ISL_5656992, EPI_ISL_5656993, EPI_ISL_5656994, EPI_ISL_5656995, EPI_ISL_5656996, EPI_ISL_5656997, EPI_ISL_5656998, EPI_ISL_5657007, EPI_ISL_5657008, EPI_ISL_5657009			
see above	Hospital Municipal Ramon Santamarina	Área de Secuenciación del Laboratorio de Virología del Hospital de Niños Dr. Ricardo Gutierrez on behalf of 'Proyecto Argentino Interinstitucional de genomica de SARS-CoV-2' (PAIS Consortium)	Acuña; Alexay; Carolina Ceriani; D; Goya; Guillermina Dolcini; LE; Lusso; M; MI; Nabaes Jodar; Natale; S; Sandra Perez; Valinotto; Victoria Nieto Farias; Viegas
EPI_ISL_3759743	Hospital PICUN LEUFU	Laboratorio Central Mg. Luis Alfredo Pianciola	C Pintos; C Rastellini; C Ziehm; J Ousset; L Pianciola; M Fernandez; M Mazzeo; M Viegas.
EPI_ISL_3759751	Hospital PLOTTIER	Laboratorio Central Mg. Luis Alfredo Pianciola	C Pintos; C Rastellini; C Ziehm; J Ousset; L Pianciola; M Fernandez; M Mazzeo; M Viegas.
EPI_ISL_2348732	Hospital Provincial Neuquen Dr. Eduardo Castro Rendon	Laboratorio Central Mg. Luis Alfredo Pianciola on behalf of 'Proyecto Argentino Interinstitucional de genomica de SARS-CoV-2' (PAIS Consortium)	C Pintos; C Rastellini; C Ziehm; J Ousset; L Pianciola; M Fernandez; M Mazzeo
EPI_ISL_5335640, EPI_ISL_5335666, EPI_ISL_5348011	Hospital Provincial de Rosario	Laboratorio Mixto de Biotecnología Acuática (LMBA), on behalf of 'Proyecto Argentino Interinstitucional de genomica de SARS-CoV-2' (PAIS Consortium)	Adriana Giri; Agustina Cerri; Ana Cavatorta; Diego Chouhy; Elisa Bolatti; Gastón Viarengo; German Perez; Julian Acosta; Lucia Moriena; María Re; Pablo Casal; Silvia Arranz; Vanina Villanova; Victoria Posner
EPI_ISL_2348734, EPI_ISL_2348736	Hospital Regional Ramon Carillo	Laboratorio Central Mg. Luis Alfredo Pianciola on behalf of 'Proyecto Argentino Interinstitucional de genomica de SARS-CoV-2' (PAIS Consortium)	C Pintos; C Rastellini; C Ziehm; J Ousset; L Pianciola; M Fernandez; M Mazzeo
EPI_ISL_3759754	Hospital SAN PATRICIO DEL CHAÑAR	Laboratorio Central Mg. Luis Alfredo Pianciola	C Pintos; C Rastellini; C Ziehm; J Ousset; L Pianciola; M Fernandez; M Mazzeo; M Viegas.
EPI_ISL_3568634, EPI_ISL_3568637, EPI_ISL_3568638	Hospital San Martin de los Andes	Laboratorio Central Mg. Luis Alfredo Pianciola	C Pintos; C Rastellini; C Ziehm; J Ousset; L Pianciola; M Fernandez; M Mazzeo; M Viegas.
EPI_ISL_3759755	Hospital VILLA LA ANGOSTURA	Laboratorio Central Mg. Luis Alfredo Pianciola	C Pintos; C Rastellini; C Ziehm; J Ousset; L Pianciola; M Fernandez; M Mazzeo; M Viegas.
EPI_ISL_3759746, EPI_ISL_3759747	Hospital ZAPALA	Laboratorio Central Mg. Luis Alfredo Pianciola	C Pintos; C Rastellini; C Ziehm; J Ousset; L Pianciola; M Fernandez; M Mazzeo; M Viegas.
EPI_ISL_2348740	Hospital de Area Alumine	Laboratorio Central Mg. Luis Alfredo Pianciola on behalf of 'Proyecto Argentino Interinstitucional de genomica de SARS-CoV-2' (PAIS Consortium)	C Pintos; C Rastellini; C Ziehm; J Ousset; L Pianciola; M Fernandez; M Mazzeo
EPI_ISL_2348735	Hospital de Area Junin de los Andes	Laboratorio Central Mg. Luis Alfredo Pianciola on behalf of 'Proyecto Argentino Interinstitucional de genomica de SARS-CoV-2' (PAIS Consortium)	C Pintos; C Rastellini; C Ziehm; J Ousset; L Pianciola; M Fernandez; M Mazzeo
EPI_ISL_2348739, EPI_ISL_2348741	Hospital de Area Plottier	Laboratorio Central Mg. Luis Alfredo Pianciola on behalf of 'Proyecto Argentino Interinstitucional de genomica de SARS-CoV-2' (PAIS Consortium)	C Pintos; C Rastellini; C Ziehm; J Ousset; L Pianciola; M Fernandez; M Mazzeo
EPI_ISL_2348731	Hospital de Area Villa la Angostura Dr. Oscar Arraiz	Laboratorio Central Mg. Luis Alfredo Pianciola on behalf of 'Proyecto Argentino Interinstitucional de genomica de SARS-CoV-2' (PAIS Consortium)	C Pintos; C Rastellini; C Ziehm; J Ousset; L Pianciola; M Fernandez; M Mazzeo

EPI_ISL_1395330, EPI_ISL_1395543	Hospital de Clínicas "José de San Martín"	Área de Secuenciación del Laboratorio de Virología del Hospital de Niños Dr. Ricardo Gutierrez on behalf of 'Proyecto Argentino Interinstitucional de genomica de SARS-CoV-2' (PAIS Consortium)	Acuña; Alexay; Analia Patricia Toledano; D; Dra. Maria Lucia Gallo Vaulet; Goya; LE; Lusso; M; MI; Marcelo Rodríguez Fermepin; Nabaes Jodar; Natale; S; Valinotto; Viegas, M.
EPI_ISL_1395123, EPI_ISL_1395124, EPI_ISL_1395125, EPI_ISL_1395126, EPI_ISL_1395127, EPI_ISL_1395128, EPI_ISL_1395129, EPI_ISL_2449263, EPI_ISL_2449278, EPI_ISL_2449279, EPI_ISL_2449280, EPI_ISL_2449281, EPI_ISL_2449289, EPI_ISL_2449290, EPI_ISL_2449291, EPI_ISL_2449292, EPI_ISL_2449293, EPI_ISL_2449294, EPI_ISL_2449295, EPI_ISL_2449310, EPI_ISL_2449311, EPI_ISL_2449440, EPI_ISL_2449441, EPI_ISL_2449442, EPI_ISL_2449443, EPI_ISL_2449444, EPI_ISL_2449445, EPI_ISL_2449446, EPI_ISL_2449447, EPI_ISL_2449448, EPI_ISL_2449449, EPI_ISL_2449450, EPI_ISL_2449451, EPI_ISL_2449452, EPI_ISL_2449453, EPI_ISL_3278134, EPI_ISL_3278135, EPI_ISL_3278136, EPI_ISL_3278137, EPI_ISL_3278138, EPI_ISL_3278139, EPI_ISL_3278140, EPI_ISL_3278141, EPI_ISL_3278142, EPI_ISL_3278143, EPI_ISL_3278144, EPI_ISL_3278145, EPI_ISL_3278146, EPI_ISL_3278147, EPI_ISL_3278148, EPI_ISL_3278149, EPI_ISL_3278150, EPI_ISL_3278151, EPI_ISL_3278152, EPI_ISL_3278153, EPI_ISL_3278154, EPI_ISL_3278155, EPI_ISL_5656636, EPI_ISL_5656637, EPI_ISL_5656638, EPI_ISL_5656639, EPI_ISL_5656640, EPI_ISL_5656641, EPI_ISL_5656642, EPI_ISL_5656643, EPI_ISL_5656644, EPI_ISL_5656645, EPI_ISL_5656646, EPI_ISL_5656647, EPI_ISL_5656648, EPI_ISL_5656649, EPI_ISL_5656650, EPI_ISL_5656651, EPI_ISL_5656892, EPI_ISL_5656893, EPI_ISL_5656894, EPI_ISL_5656895, EPI_ISL_5656896, EPI_ISL_5656897, EPI_ISL_5656898, EPI_ISL_5656899, EPI_ISL_5656900, EPI_ISL_5656901, EPI_ISL_5656902, EPI_ISL_5656903, EPI_ISL_5656904, EPI_ISL_5656905, EPI_ISL_5656906, EPI_ISL_5656907, EPI_ISL_5656908, EPI_ISL_5656909, EPI_ISL_5656910, EPI_ISL_5656911, EPI_ISL_5656912, EPI_ISL_5657034, EPI_ISL_5657035, EPI_ISL_5657036, EPI_ISL_5657037, EPI_ISL_5657038, EPI_ISL_5657039, EPI_ISL_5657040, EPI_ISL_5657041, EPI_ISL_5657126, EPI_ISL_5657127, EPI_ISL_5657128, EPI_ISL_5657129, EPI_ISL_5657130, EPI_ISL_5657131, EPI_ISL_5657132, EPI_ISL_5657133, EPI_ISL_5657134, EPI_ISL_5657135, EPI_ISL_5657136, EPI_ISL_5657137, EPI_ISL_5657138, EPI_ISL_5657139, EPI_ISL_5657140, EPI_ISL_5657353			
see above	Instituto Nacional de Epidemiología "Dr. Jara"	Área de Secuenciación del Laboratorio de Virología del Hospital de Niños Dr. Ricardo Gutierrez on behalf of 'Proyecto Argentino Interinstitucional de genomica de SARS-CoV-2' (PAIS Consortium)	Acuña; Alexay; Carlos Jose Cimmino; D; Goya; Irene Pagano; LE; Lusso; M; MI; Nabaes Jodar; Natale; Osvaldo Uez; S; Valinotto; Viegas; Viegas, M.
EPI_ISL_1395281, EPI_ISL_1395282, EPI_ISL_1395283, EPI_ISL_1395284, EPI_ISL_1395318, EPI_ISL_1395340, EPI_ISL_1395341, EPI_ISL_1395342, EPI_ISL_1395343, EPI_ISL_1395344, EPI_ISL_1395345, EPI_ISL_1395346, EPI_ISL_1395347, EPI_ISL_1395348, EPI_ISL_1395349, EPI_ISL_1395373, EPI_ISL_1395374, EPI_ISL_1395375, EPI_ISL_1395376, EPI_ISL_1395377, EPI_ISL_2449165, EPI_ISL_2449166, EPI_ISL_2449167, EPI_ISL_2449168, EPI_ISL_2449169, EPI_ISL_2449170, EPI_ISL_2449171, EPI_ISL_2449172, EPI_ISL_2449173, EPI_ISL_2449174, EPI_ISL_2449175, EPI_ISL_2449176, EPI_ISL_2449177, EPI_ISL_2449178			
see above	Instituto de Investigaciones Biomédicas en Retrovirus y SIDA (INBIRS)	Área de Secuenciación del Laboratorio de Virología del Hospital de Niños Dr. Ricardo Gutierrez on behalf of 'Proyecto Argentino Interinstitucional de genomica de SARS-CoV-2' (PAIS Consortium)	Acuña; Alexay; D; F; Federico Remes Lenicov; Geffner; Goya; H; Horacio Salomón; J; LE; Lusso; M; MI; Nabaes Jodar; Natale; Remes Lenicov; S; Salomón; Seery; V; Valinotto; Vanesa Seery; Viegas, M.
EPI_ISL_5656667, EPI_ISL_5656668, EPI_ISL_5656669, EPI_ISL_5656670, EPI_ISL_5656671, EPI_ISL_5656672, EPI_ISL_5656673, EPI_ISL_5656674, EPI_ISL_5656675, EPI_ISL_5656763, EPI_ISL_5656764, EPI_ISL_5656765, EPI_ISL_5656766, EPI_ISL_5656767, EPI_ISL_5656768, EPI_ISL_5656769, EPI_ISL_5656873, EPI_ISL_5657109, EPI_ISL_5657110, EPI_ISL_5657111, EPI_ISL_5657112, EPI_ISL_5657113, EPI_ISL_5657114, EPI_ISL_5657115, EPI_ISL_5657116, EPI_ISL_5657117, EPI_ISL_5657267, EPI_ISL_5657273, EPI_ISL_5657278, EPI_ISL_5657282, EPI_ISL_5657289			
see above	Instituto de Investigaciones Biomédicas en Retrovirus y Sida, CONICET-UBA (INBIRS)	Área de Secuenciación del Laboratorio de Virología del Hospital de Niños Dr. Ricardo Gutierrez on behalf of 'Proyecto Argentino Interinstitucional de genomica de SARS-CoV-2' (PAIS Consortium)	Acuña; Adamczyk Alan; Alexay; Alves Camila; Arruvito Lourdes; Azzolina Rolon Sabrina; Belauzarán Laura; Benencio Paula; Berardino Bruno; Berini Carolina; Biglione Mirna; Blond Oliver; Budziñski Maia; Cabrerizo Gonzalo; Cairoli Victoria; Carobene Mauricio; Cassime Ricardo; Cassime Silvia; Ceballos Ana; Cevallos Cintia Gisela; Czernikier Alejandro; D; Delpino María Victoria; Di Diego García Facundo; Duarte Alejandra; Ducasa Nicolás; Elizalde María Mercedes; Elía Andrés; Erdocia Mariana; Fabiano; Fava Agustina; Felder Leandro; Flichman Diego; Fontecha María Belén; Friedrich Adrián David; Fuchs Wighman Federico; Gatti Ramiro Daniel; Geffner Jorge; Ghiglione Yanina Alejandra; Giannone Denise Anabella; Girotti Romina; Giusti Sebastián; Gonzalez Polo Virginia; Goya; Gómez Claudio; Hermida Alava Katherine; Holgado María Pia; LE; Laufer Natalia; Leicaj Luz; Longueira Yesica; Lopez Malizzia Alvaro; Lusso; M; MI; Manselle Cocco Montana; Mansilla María Agustina; Marin Franco Jose; Martina; Massillo Cintia; Mazzitelli Ignacio; Melucci Ganzarain Claudia; Meneghini María Agustina; Molina María Carolina; Montesano Fernando; Morando Nicolás; Nabaes Jodar; Natale; Ostrowski Matías; Paletta Ana Luz; Pampuro Sandra; Pando María; Pascuale Carla; Penas Federico; Piccardo Claudio; Pieralisi Azul; Pippo Mónica; Polo María Laura; Pérez Paula; Quiroga Cecilia; Quiroga Florencia; Ramirez Ezequiel; Remes Lenicov Federico; Rodriguez Jimena; S; Sabatté Juan; Salomón Horacio; Salvatori Melina; Sananes Inés; Santilli Cecilia; Sede Mariano; Seery Vanesa; Sierra Jessica Mariel; Sonzogni Silvina; Speroni Micaela; Trifone César; Trotta Aldana; Turk Gabriela; Valinotto; Varese Augusto; Vellón Luciano; Vera Aguilar Douglas; Vergara Maricef; Verneri Paula; Viegas; Waisman Ariel Witteveen Camila
EPI_ISL_5657469, EPI_ISL_5657477, EPI_ISL_5657478, EPI_ISL_5657791, EPI_ISL_5657792	LABORATORIO DE SALUD PUBLICA	Área de Secuenciación del Laboratorio de Virología del Hospital de Niños Dr. Ricardo Gutierrez on behalf of 'Proyecto Argentino Interinstitucional de genomica de SARS-CoV-2' (PAIS Consortium)	Acuña; Alexay; Belén Ortiz; Cristian Garay; D; Fernando Giuliani; Goya; LE; Luciana Martinez; Lusso; M; MI; Nabaes Jodar; Natale; S; Silvia Zerrer; Silvina Denita; Valinotto; Viegas; Viviana Leiva
EPI_ISL_5658034, EPI_ISL_5658038	Laboratorio del Hospital San Roque de Gonnet	Área de Secuenciación del Laboratorio de Virología del Hospital de Niños Dr. Ricardo Gutierrez on behalf of 'Proyecto Argentino Interinstitucional de genomica de SARS-CoV-2' (PAIS Consortium)	Acuña; Alexay; D; Evelyn De la Rubia; Goya; LE; Lusso; M; MI; Nabaes Jodar; Nadalich; Natale; Paula Carassi; Rosana Isabel Toro; S; Valinotto; Victoria; Viegas
EPI_ISL_2449016, EPI_ISL_2449692, EPI_ISL_2449693, EPI_ISL_2449737, EPI_ISL_2449738, EPI_ISL_2449759, EPI_ISL_2449816, EPI_ISL_2449817, EPI_ISL_2449823, EPI_ISL_2449824, EPI_ISL_2449825, EPI_ISL_2449827, EPI_ISL_2449828, EPI_ISL_2449831, EPI_ISL_2449832, EPI_ISL_2449833, EPI_ISL_2449836, EPI_ISL_2449841, EPI_ISL_2449844, EPI_ISL_2449859, EPI_ISL_2449860, EPI_ISL_2449861			
see above	Laboratorio Central "Mg. Luis Alfredo Pianciola"	Área de Secuenciación del Laboratorio de Virología del Hospital de Niños Dr. Ricardo Gutierrez on behalf of 'Proyecto Argentino Interinstitucional de genomica de SARS-CoV-2' (PAIS Consortium)	A; Acuña; Alexay; C; D; Fernández; Goya; LE; Lusso; M; MC; MI; Mazzeo; Nabaes Jodar; Natale; Pianciola, L.; Pinto; S; Valinotto; Viegas, M.; Ziehm
EPI_ISL_3908034, EPI_ISL_3908035, EPI_ISL_3908036, EPI_ISL_3908037, EPI_ISL_3908038, EPI_ISL_3908039, EPI_ISL_3908040, EPI_ISL_3908041, EPI_ISL_3908042, EPI_ISL_3908043, EPI_ISL_3908044, EPI_ISL_3908045, EPI_ISL_3908046, EPI_ISL_3908047, EPI_ISL_3908048, EPI_ISL_3908049, EPI_ISL_3908050, EPI_ISL_3908051, EPI_ISL_3908052, EPI_ISL_3908053, EPI_ISL_3908054, EPI_ISL_3908055, EPI_ISL_3908056, EPI_ISL_3908513, EPI_ISL_3908514, EPI_ISL_3908515, EPI_ISL_3908516, EPI_ISL_3908517, EPI_ISL_3908518, EPI_ISL_3908519, EPI_ISL_3908520, EPI_ISL_3908521, EPI_ISL_3908522, EPI_ISL_3908523, EPI_ISL_3908524, EPI_ISL_3908525, EPI_ISL_3908526, EPI_ISL_3908527, EPI_ISL_3908528, EPI_ISL_3908529, EPI_ISL_3908530, EPI_ISL_3908531, EPI_ISL_3908532, EPI_ISL_3945483, EPI_ISL_3945484, EPI_ISL_3945485, EPI_ISL_3945486, EPI_ISL_3945487, EPI_ISL_3945488, EPI_ISL_3945489, EPI_ISL_3945490, EPI_ISL_3945491, EPI_ISL_3945492, EPI_ISL_3945493, EPI_ISL_3945494, EPI_ISL_3945495, EPI_ISL_3945496, EPI_ISL_3945497, EPI_ISL_3945498, EPI_ISL_3945499, EPI_ISL_3945500, EPI_ISL_3945501, EPI_ISL_3945502, EPI_ISL_3945503, EPI_ISL_3945504, EPI_ISL_3945505, EPI_ISL_3945506, EPI_ISL_3945507, EPI_ISL_3945508, EPI_ISL_3945509, EPI_ISL_3945510, EPI_ISL_3945511, EPI_ISL_3945512, EPI_ISL_3945513, EPI_ISL_3945514, EPI_ISL_3945515, EPI_ISL_3945516, EPI_ISL_3945517, EPI_ISL_3945518, EPI_ISL_3945519, EPI_ISL_3945520, EPI_ISL_3945521, EPI_ISL_3945522, EPI_ISL_3945523, EPI_ISL_3945524, EPI_ISL_3945525, EPI_ISL_3945526			
see above	Laboratorio Central Mg. Luis Alfredo Pianciola	Laboratorio Central Mg. Luis Alfredo Pianciola	C Pintos; C Rastellini; C Ziehm; J Ousset; L Pianciola; L Pianciola.; M Fernandez; M Mazzeo; M Viegas.
EPI_ISL_3861188, EPI_ISL_3861190, EPI_ISL_3861192, EPI_ISL_3861194, EPI_ISL_3861196, EPI_ISL_3861198, EPI_ISL_3861202, EPI_ISL_3861207, EPI_ISL_3861208, EPI_ISL_3861210, EPI_ISL_3861212, EPI_ISL_3861213, EPI_ISL_3861215, EPI_ISL_3861217, EPI_ISL_3861219, EPI_ISL_3861220, EPI_ISL_3861222, EPI_ISL_3861224, EPI_ISL_3861225, EPI_ISL_3861227, EPI_ISL_3861229, EPI_ISL_3861233, EPI_ISL_3861237, EPI_ISL_3861241, EPI_ISL_3861245, EPI_ISL_3861247, EPI_ISL_3861337, EPI_ISL_3861342, EPI_ISL_3861347, EPI_ISL_3861351, EPI_ISL_3861355, EPI_ISL_3861359, EPI_ISL_3861364, EPI_ISL_3861367, EPI_ISL_3861371, EPI_ISL_3861375, EPI_ISL_3861379, EPI_ISL_3861383, EPI_ISL_3861385, EPI_ISL_3861387, EPI_ISL_3861389, EPI_ISL_3861390, EPI_ISL_3861392, EPI_ISL_3862480, EPI_ISL_3862487, EPI_ISL_3862491, EPI_ISL_3862495, EPI_ISL_3862497, EPI_ISL_3862499, EPI_ISL_3862501, EPI_ISL_3862503, EPI_ISL_3862504, EPI_ISL_3862506, EPI_ISL_3862508, EPI_ISL_3862510, EPI_ISL_3862514, EPI_ISL_3862518, EPI_ISL_3862523, EPI_ISL_3862527, EPI_ISL_3862532, EPI_ISL_4207071, EPI_ISL_4207072, EPI_ISL_4207073, EPI_ISL_4207074, EPI_ISL_4207075, EPI_ISL_4207076, EPI_ISL_4207077, EPI_ISL_4207078, EPI_ISL_4207079, EPI_ISL_4731515, EPI_ISL_4731516, EPI_ISL_4731517, EPI_ISL_4731518, EPI_ISL_4731519, EPI_ISL_4731520, EPI_ISL_4731521, EPI_ISL_5331023, EPI_ISL_5331024, EPI_ISL_5331025, EPI_ISL_5331026, EPI_ISL_5331027, EPI_ISL_5331028, EPI_ISL_5331029, EPI_ISL_5331030, EPI_ISL_5331031, EPI_ISL_5331032, EPI_ISL_5331033, EPI_ISL_5331034, EPI_ISL_5331035, EPI_ISL_5331036, EPI_ISL_5331037, EPI_ISL_5331301, EPI_ISL_5331308, EPI_ISL_5331315, EPI_ISL_5331324, EPI_ISL_5331332, EPI_ISL_5331339, EPI_ISL_5331345, EPI_ISL_5331351, EPI_ISL_5331354, EPI_ISL_5332687, EPI_ISL_5332688, EPI_ISL_5332689, EPI_ISL_5332690, EPI_ISL_5332691, EPI_ISL_5332692, EPI_ISL_5332693, EPI_ISL_5332694, EPI_ISL_5332695, EPI_ISL_5332696, EPI_ISL_5332697, EPI_ISL_5332698, EPI_ISL_5332699, EPI_ISL_5332700, EPI_ISL_5332701, EPI_ISL_5332702, EPI_ISL_5332703, EPI_ISL_5332704, EPI_ISL_5332705, EPI_ISL_5332706, EPI_ISL_5332707, EPI_ISL_5332708, EPI_ISL_5332709, EPI_ISL_5332710, EPI_ISL_5332711, EPI_ISL_5332712, EPI_ISL_5332971, EPI_ISL_5332979, EPI_ISL_5332988, EPI_ISL_5332993, EPI_ISL_5332994, EPI_ISL_5333001, EPI_ISL_5333002, EPI_ISL_5333003, EPI_ISL_5333004, EPI_ISL_5333005, EPI_ISL_5333006, EPI_ISL_5333007, EPI_ISL_5333008, EPI_ISL_5333009, EPI_ISL_5333010, EPI_ISL_5333011, EPI_ISL_5333012, EPI_ISL_5333013, EPI_ISL_5333014, EPI_ISL_5416042, EPI_ISL_5416043, EPI_ISL_5416044, EPI_ISL_5416045, EPI_ISL_5416046, EPI_ISL_5416047, EPI_ISL_5416048, EPI_ISL_5416049, EPI_ISL_5416050, EPI_ISL_5416051, EPI_ISL_5416052, EPI_ISL_5416053, EPI_ISL_5416054, EPI_ISL_5416055, EPI_ISL_5416056, EPI_ISL_5416057, EPI_ISL_5416058, EPI_ISL_5416059, EPI_ISL_5416060, EPI_ISL_5416061, EPI_ISL_5596932, EPI_ISL_5596933, EPI_ISL_5596934, EPI_ISL_5596935, EPI_ISL_5596936, EPI_ISL_5596937, EPI_ISL_5596938, EPI_ISL_5596939, EPI_ISL_5596940, EPI_ISL_5596941, EPI_ISL_5596942, EPI_ISL_5596943, EPI_ISL_5596944, EPI_ISL_5596945, EPI_ISL_5596946, EPI_ISL_5603598, EPI_ISL_5603599, EPI_ISL_5603607, EPI_ISL_5603608, EPI_ISL_5603613, EPI_ISL_5603620, EPI_ISL_5603621, EPI_ISL_5603629, EPI_ISL_5603630, EPI_ISL_5603636, EPI_ISL_5603637, EPI_ISL_5603643, EPI_ISL_5603644, EPI_ISL_5603649, EPI_ISL_5603655			
see above	Laboratorio Central Mg. Luis Alfredo Pianciola	Laboratorio Central Mg. Luis Alfredo Pianciola on behalf of 'Proyecto Argentino Interinstitucional de genomica de SARS-CoV-2' (PAIS Consortium)	C Pintos; C Rastellini; C Rastellini.; C Ziehm; J Ousset; L Pianciola; L Pianciola.; M Fernandez; M Mazzeo; M Viegas.
EPI_ISL_5657198	Laboratorio De Salud Pública	Área de Secuenciación del Laboratorio de Virología del Hospital de Niños Dr. Ricardo Gutierrez on behalf of 'Proyecto Argentino Interinstitucional de genomica de SARS-CoV-2' (PAIS Consortium)	Acuña; Alexay; Belén Ortiz; Cristian Garay; D; Fernando Giuliani; Goya; LE; Luciana Martinez; Lusso; M; MI; Nabaes Jodar; Natale; S; Silvia Zerrer; Silvina Denita; Valinotto; Viegas; Viviana Leiva
EPI_ISL_5658001, EPI_ISL_5658002	Laboratorio Vacunas Salud (VacSal) del Instituto de Biotecnología y Biología Molecular de la Facultad de Ciencias Exactas de la Universidad Nacional de La Plata-CONICET	Área de Secuenciación del Laboratorio de Virología del Hospital de Niños Dr. Ricardo Gutierrez on behalf of 'Proyecto Argentino Interinstitucional de genomica de SARS-CoV-2' (PAIS Consortium)	Acuña; Alejandra Giordano; Alexay; Aníbal Lodeiro; D; Daniela Bottero; Erika Rudi; Eugenia Zurita y Daniela Hozbor; Goya; Keila Belhart; LE; Lusso; M; MI; Magalí Gabrielli; Nabaes Jodar; Natale; Nicolás Ambrosis; Pablo Martin Aispuro; S; Valinotto; Viegas
EPI_ISL_2449631, EPI_ISL_2449663, EPI_ISL_2449664, EPI_ISL_2449710, EPI_ISL_2449711, EPI_ISL_2449739	Laboratorio de Biología Molecular Bolívar	Área de Secuenciación del Laboratorio de Virología del Hospital de Niños Dr. Ricardo Gutierrez on behalf of 'Proyecto Argentino Interinstitucional de genomica de SARS-CoV-2' (PAIS Consortium)	Acuña; Alexay; Cabrerizo; Caviglia; D; Di Claudio; D'Andrea; F; Goya; Jofré; LE; Larreche; Lescano; Lusso; M; MA; ME; MI; ML; MR; Martínez; Nabaes Jodar; Natale; R; S; Ticeira; Valinotto; Viegas, M.
EPI_ISL_3277885, EPI_ISL_3277901, EPI_ISL_3278174, EPI_ISL_3278175, EPI_ISL_3278176, EPI_ISL_3278177, EPI_ISL_3278178, EPI_ISL_3278179, EPI_ISL_3278180, EPI_ISL_3278181, EPI_ISL_3278182, EPI_ISL_3278183			
see above	Laboratorio de Biología Molecular Bolívar (LABBO)	Área de Secuenciación del Laboratorio de Virología del Hospital de Niños Dr. Ricardo Gutierrez on behalf of 'Proyecto Argentino Interinstitucional de genomica de SARS-CoV-2' (PAIS Consortium)	Acuña; Alexay; D; Felisa Lescano; Fiorella Di Claudio; Goya; LE; Lusso; M; MI; Marina Ticeira; María Estela Jofré; María Laura Caviglia; María Roció Larreche; María de los Ángeles D'Andrea; Micaela Martínez; Nabaes Jodar; Natale; Romina Cabrerizo; S; Valinotto; Viegas
EPI_ISL_1394885, EPI_ISL_1395177, EPI_ISL_2449079, EPI_ISL_2449093, EPI_ISL_2449109, EPI_ISL_2449122, EPI_ISL_2449123, EPI_ISL_2449140, EPI_ISL_2449141, EPI_ISL_2449142, EPI_ISL_2449151, EPI_ISL_2449185, EPI_ISL_2449186, EPI_ISL_3278048, EPI_ISL_3278049, EPI_ISL_3278050, EPI_ISL_3278051, EPI_ISL_3278052, EPI_ISL_3278053, EPI_ISL_3278054, EPI_ISL_3278055, EPI_ISL_3278056, EPI_ISL_3278057, EPI_ISL_3278058, EPI_ISL_3278059, EPI_ISL_3278060, EPI_ISL_3278061, EPI_ISL_3278062, EPI_ISL_3278063, EPI_ISL_3278064, EPI_ISL_3278065, EPI_ISL_3278066, EPI_ISL_3278067, EPI_ISL_3278068, EPI_ISL_3278069, EPI_ISL_3278070, EPI_ISL_3278071, EPI_ISL_3278072, EPI_ISL_3278073, EPI_ISL_3278074, EPI_ISL_3278075, EPI_ISL_3278076, EPI_ISL_3278077, EPI_ISL_3278078, EPI_ISL_3278079, EPI_ISL_3278080, EPI_ISL_3278081, EPI_ISL_3278082, EPI_ISL_3278083, EPI_ISL_3278084, EPI_ISL_3278085, EPI_ISL_3278086, EPI_ISL_3278087			
see above	Laboratorio de Biología Molecular Hospital Pedro de Elizalde	Área de Secuenciación del Laboratorio de Virología del Hospital de Niños Dr. Ricardo Gutierrez on behalf of 'Proyecto Argentino Interinstitucional de genomica de SARS-CoV-2' (PAIS Consortium)	Acuña; Aldana Claps; Alexay; Belen Carolina Diaz; D; Daiana Rosales; Emilce osaba; Eugenia Paez; Federico José Torres; Florencia Alegre; Franco Morandi; Goya; Gretel Wenk; Jaqueline Rocovich; Javier Indart; Julieta Gondolessi; Julián Chamorro; LE; Laura Bressan; Luciana Montoto Piazza; Lusso; M; MI; Maria Laura Sueiro; Marisa Lorena Gómez; María Eugenia Martín; María Florencia Sanchez; Nabaes Jodar; Natale; Natalia Zamora; Paulina Marchetti; S; Valinotto; Viegas, M.
EPI_ISL_5656726, EPI_ISL_5656727, EPI_ISL_5656728, EPI_ISL_5656729, EPI_ISL_5656730, EPI_ISL_5656731, EPI_ISL_5656732, EPI_ISL_5656733, EPI_ISL_5656734, EPI_ISL_5656735, EPI_ISL_5656736, EPI_ISL_5656737, EPI_ISL_5656738, EPI_ISL_5656739, EPI_ISL_5656740, EPI_ISL_5656741, EPI_ISL_5656778, EPI_ISL_5656779, EPI_ISL_5656780, EPI_ISL_5656781, EPI_ISL_5656782, EPI_ISL_5656783, EPI_ISL_5656784, EPI_ISL_5656785, EPI_ISL_5656814, EPI_ISL_5656815, EPI_ISL_5656816, EPI_ISL_5656817, EPI_ISL_5656870, EPI_ISL_5656871, EPI_ISL_5656872, EPI_ISL_5657060, EPI_ISL_5657061, EPI_ISL_5657062, EPI_ISL_5657063, EPI_ISL_5657104, EPI_ISL_5657105, EPI_ISL_5657106, EPI_ISL_5657107, EPI_ISL_5657108, EPI_ISL_5657168, EPI_ISL_5657169, EPI_ISL_5657170, EPI_ISL_5657171, EPI_ISL_5657172, EPI_ISL_5657194, EPI_ISL_5657195, EPI_ISL_5657196, EPI_ISL_5657197, EPI_ISL_5657242, EPI_ISL_5657243, EPI_ISL_5657244, EPI_ISL_5657245, EPI_ISL_5657246, EPI_ISL_5657247, EPI_ISL_5657248, EPI_ISL_5657249, EPI_ISL_5657250, EPI_ISL_5657251, EPI_ISL_5657252, EPI_ISL_5657253, EPI_ISL_5657254, EPI_ISL_5657255, EPI_ISL_5657256, EPI_ISL_5657257, EPI_ISL_5657258,			

EPI_ISL_5657259, EPI_ISL_5657354, EPI_ISL_5657374, EPI_ISL_5657378, EPI_ISL_5657379, EPI_ISL_5657384, EPI_ISL_5657391, EPI_ISL_5657392, EPI_ISL_5657395, EPI_ISL_5657396, EPI_ISL_5657680, EPI_ISL_5657683, EPI_ISL_5657687, EPI_ISL_5657694, EPI_ISL_5657695, EPI_ISL_5657697, EPI_ISL_5657698, EPI_ISL_5657705, EPI_ISL_5657713, EPI_ISL_5657714, EPI_ISL_5657722, EPI_ISL_5657732, EPI_ISL_5657733, EPI_ISL_5657735, EPI_ISL_5657736, EPI_ISL_5657841, EPI_ISL_5657847, EPI_ISL_5657848, EPI_ISL_5657854, EPI_ISL_5657858, EPI_ISL_5657859, EPI_ISL_5657863, EPI_ISL_5657864, EPI_ISL_5657870, EPI_ISL_5657876, EPI_ISL_5657877, EPI_ISL_5657880, EPI_ISL_5657890, EPI_ISL_5657891, EPI_ISL_5657895, EPI_ISL_5657904, EPI_ISL_5657905, EPI_ISL_5657908, EPI_ISL_5657909, EPI_ISL_5657917			
see above	Laboratorio de Biología Molecular UFU y Epidemiología Hospital Pedro de Elizalde	Área de Secuenciación del Laboratorio de Virología del Hospital de Niños Dr. Ricardo Gutierrez on behalf of 'Proyecto Argentino Interinstitucional de genomica de SARS-CoV-2' (PAIS Consortium)	Acuña; Aldana Claps; Alejo Fernandez; Alexay; Belén Carolina Diaz; D; Daiana Rosales; Eugenia Paez; Eugenia Sevilla; Federico Lorenzo; Florencia Alegre; Franco Morandi; Goya; Gretel Wenk; Jaqueline Ramirez; Jaqueline Rocovich; Javier Indart; Jimena Castedo; Julieta Gondolessi; Julián Chamorro; LE; Laura Miño; Luciana Montoto Piazza; Lusso; M; MI; Maria Jose Rial; Maria Laura Sueiro; Nabaes Jodar; Natale; Natalia Zamora; Paulina Marchetti; Ricardo Del Olmo; Rocío Romero; Ruth Galarza; S; Valinotto; Viegas; Vivian Bokser
EPI_ISL_5656686, EPI_ISL_5656687, EPI_ISL_5656688, EPI_ISL_5656689, EPI_ISL_5656690, EPI_ISL_5656791, EPI_ISL_5656792, EPI_ISL_5656803, EPI_ISL_5656804, EPI_ISL_5656805, EPI_ISL_5656806, EPI_ISL_5656818, EPI_ISL_5656876, EPI_ISL_5656877, EPI_ISL_5656916, EPI_ISL_5656917, EPI_ISL_5656918, EPI_ISL_5656919, EPI_ISL_5656920, EPI_ISL_5656987, EPI_ISL_5656988, EPI_ISL_5656989, EPI_ISL_5657068, EPI_ISL_5657069, EPI_ISL_5657070, EPI_ISL_5657071, EPI_ISL_5657072, EPI_ISL_5657119, EPI_ISL_5657120, EPI_ISL_5657121, EPI_ISL_5657122, EPI_ISL_5657123, EPI_ISL_5657124, EPI_ISL_5657125, EPI_ISL_5657151, EPI_ISL_5657152, EPI_ISL_5657153, EPI_ISL_5657154, EPI_ISL_5657155, EPI_ISL_5657156, EPI_ISL_5657157, EPI_ISL_5657158, EPI_ISL_5657218, EPI_ISL_5657219, EPI_ISL_5657220, EPI_ISL_5657221, EPI_ISL_5657222, EPI_ISL_5657223, EPI_ISL_5657362, EPI_ISL_5657363, EPI_ISL_5657373, EPI_ISL_5657465, EPI_ISL_5657505, EPI_ISL_5657506, EPI_ISL_5657588, EPI_ISL_5657594, EPI_ISL_5657595, EPI_ISL_5657600, EPI_ISL_5657608, EPI_ISL_5657609, EPI_ISL_5657617, EPI_ISL_5657618, EPI_ISL_5658009, EPI_ISL_5658010, EPI_ISL_5658012, EPI_ISL_5658020, EPI_ISL_5658021			
see above	Laboratorio de Biología Molecular, Hospital Cosme Argerich	Área de Secuenciación del Laboratorio de Virología del Hospital de Niños Dr. Ricardo Gutierrez on behalf of 'Proyecto Argentino Interinstitucional de genomica de SARS-CoV-2' (PAIS Consortium)	Acuña; Alexay; Andrea Fernández; D; Florencia Funez; Florencia Rodríguez; Goya; Jésica Galeano; Karina Polanski; LE; Lusso; M; MI; Marcia Pozzati; Nabaes Jodar; Natale; S; Valinotto; Viegas
EPI_ISL_2449632, EPI_ISL_2449633, EPI_ISL_2449665, EPI_ISL_2449666, EPI_ISL_2449667, EPI_ISL_2449712, EPI_ISL_2449740, EPI_ISL_2449760, EPI_ISL_2449761, EPI_ISL_2449780, EPI_ISL_2449781, EPI_ISL_2449782, EPI_ISL_2449783, EPI_ISL_2449784, EPI_ISL_2449799, EPI_ISL_2449800, EPI_ISL_2449801, EPI_ISL_2449802, EPI_ISL_2449820			
see above	Laboratorio de Biología Molecular. Hospital Dr. Héctor Cura	Área de Secuenciación del Laboratorio de Virología del Hospital de Niños Dr. Ricardo Gutierrez on behalf of 'Proyecto Argentino Interinstitucional de genomica de SARS-CoV-2' (PAIS Consortium)	Acuña; Alexay; D; Ghiano; Goya; J; LE; Lusso; M; MB; MI; N; Nabaes Jodar; Natale; R; S; Spina; Turrina; Valinotto; Viegas, M.; Zaffanella
EPI_ISL_3277887, EPI_ISL_3277888, EPI_ISL_3277893, EPI_ISL_3277894, EPI_ISL_3277895, EPI_ISL_3277896, EPI_ISL_3277897, EPI_ISL_3277898, EPI_ISL_3277902, EPI_ISL_3277903, EPI_ISL_3277957, EPI_ISL_3277958, EPI_ISL_3277959, EPI_ISL_3277960, EPI_ISL_3277961, EPI_ISL_3277962, EPI_ISL_3277963, EPI_ISL_3277964, EPI_ISL_3277965, EPI_ISL_3277966, EPI_ISL_3277967, EPI_ISL_3277968, EPI_ISL_3277969, EPI_ISL_3277970, EPI_ISL_3277971, EPI_ISL_3277972, EPI_ISL_3278088, EPI_ISL_3278089, EPI_ISL_3278090, EPI_ISL_3278091, EPI_ISL_3278092, EPI_ISL_3278093, EPI_ISL_3278094, EPI_ISL_3278095, EPI_ISL_3278096, EPI_ISL_3278206, EPI_ISL_3278207, EPI_ISL_3278208, EPI_ISL_3278209, EPI_ISL_3278210, EPI_ISL_3278211, EPI_ISL_3278212, EPI_ISL_3278213, EPI_ISL_3278214, EPI_ISL_3278215, EPI_ISL_3278216, EPI_ISL_3278217, EPI_ISL_3278218, EPI_ISL_3278219, EPI_ISL_3278220, EPI_ISL_3278221, EPI_ISL_3278222, EPI_ISL_3278223, EPI_ISL_3278243, EPI_ISL_3278244, EPI_ISL_3278245, EPI_ISL_3278246, EPI_ISL_3278247, EPI_ISL_3278248, EPI_ISL_3278249, EPI_ISL_3278250, EPI_ISL_3278251, EPI_ISL_3278252, EPI_ISL_3278253, EPI_ISL_3278254, EPI_ISL_5656676, EPI_ISL_5656677, EPI_ISL_5656678, EPI_ISL_5656679, EPI_ISL_5656680, EPI_ISL_5656681, EPI_ISL_5656682, EPI_ISL_5656683, EPI_ISL_5656684, EPI_ISL_5656685, EPI_ISL_5656709, EPI_ISL_5656710, EPI_ISL_5656711, EPI_ISL_5656712, EPI_ISL_5656713, EPI_ISL_5656714, EPI_ISL_5656715, EPI_ISL_5656716, EPI_ISL_5656717, EPI_ISL_5656718, EPI_ISL_5656770, EPI_ISL_5656771, EPI_ISL_5656772, EPI_ISL_5656773, EPI_ISL_5656774, EPI_ISL_5656775, EPI_ISL_5656776, EPI_ISL_5656777, EPI_ISL_5656878, EPI_ISL_5656879, EPI_ISL_5656880, EPI_ISL_5656881, EPI_ISL_5656882, EPI_ISL_5656883, EPI_ISL_5656888, EPI_ISL_5656889, EPI_ISL_5656890, EPI_ISL_5656891, EPI_ISL_5656921, EPI_ISL_5656922, EPI_ISL_5656923, EPI_ISL_5656924, EPI_ISL_5656925, EPI_ISL_5656926, EPI_ISL_5656927, EPI_ISL_5656928, EPI_ISL_5656929, EPI_ISL_5656976, EPI_ISL_5656977, EPI_ISL_5656978, EPI_ISL_5656979, EPI_ISL_5656980, EPI_ISL_5656981, EPI_ISL_5656982, EPI_ISL_5656983, EPI_ISL_5656984, EPI_ISL_5656985, EPI_ISL_5656986, EPI_ISL_5657024, EPI_ISL_5657025, EPI_ISL_5657026, EPI_ISL_5657027, EPI_ISL_5657028, EPI_ISL_5657029, EPI_ISL_5657030, EPI_ISL_5657031, EPI_ISL_5657032, EPI_ISL_5657033, EPI_ISL_5657097, EPI_ISL_5657098, EPI_ISL_5657099, EPI_ISL_5657100, EPI_ISL_5657101, EPI_ISL_5657102, EPI_ISL_5657103, EPI_ISL_5657159, EPI_ISL_5657160, EPI_ISL_5657161, EPI_ISL_5657162, EPI_ISL_5657163, EPI_ISL_5657164, EPI_ISL_5657165, EPI_ISL_5657166, EPI_ISL_5657167, EPI_ISL_5657193, EPI_ISL_5657307, EPI_ISL_5657308, EPI_ISL_5657314, EPI_ISL_5657322, EPI_ISL_5657323, EPI_ISL_5657330, EPI_ISL_5657339, EPI_ISL_5657340, EPI_ISL_5657344, EPI_ISL_5657348, EPI_ISL_5657420, EPI_ISL_5657421, EPI_ISL_5657427, EPI_ISL_5657433, EPI_ISL_5657434, EPI_ISL_5657437, EPI_ISL_5657444, EPI_ISL_5657445, EPI_ISL_5657452, EPI_ISL_5657453, EPI_ISL_5657456, EPI_ISL_5657512, EPI_ISL_5657513, EPI_ISL_5657522, EPI_ISL_5657529, EPI_ISL_5657530, EPI_ISL_5657534, EPI_ISL_5657535, EPI_ISL_5657538, EPI_ISL_5657540, EPI_ISL_5657541, EPI_ISL_5657642, EPI_ISL_5657648, EPI_ISL_5657649, EPI_ISL_5657657, EPI_ISL_5657659, EPI_ISL_5657669, EPI_ISL_5657675, EPI_ISL_5657676, EPI_ISL_5657926, EPI_ISL_5657933, EPI_ISL_5657934, EPI_ISL_5657939, EPI_ISL_5657940, EPI_ISL_5657943, EPI_ISL_5657951, EPI_ISL_5657952, EPI_ISL_5657959, EPI_ISL_5657960, EPI_ISL_5657963, EPI_ISL_5657968, EPI_ISL_5657975, EPI_ISL_5657976, EPI_ISL_5657985			
see above	Laboratorio de Biología molecular del Hospital General de Agudos Dr. Carlos G. Durand	Área de Secuenciación del Laboratorio de Virología del Hospital de Niños Dr. Ricardo Gutierrez on behalf of 'Proyecto Argentino Interinstitucional de genomica de SARS-CoV-2' (PAIS Consortium)	Acuña; Adriana Rozo; Ailin Domínguez; Albano Costa; Alexay; Anisa Marchissio; Belen Warszatska; Camila Elsegood; Cecilia Irrazábal; Clara Theaux; Constanza Marina; D; Estela Dima; Eugenia Langan; Flavia Rivarola; Goya; Guillermo Notaristéfano; LE; Laura Jurado; Laura Álvarez; Lucas Dahinten; Lusso; M; MI; Maricel Leivas; Martín Rodríguez Saá; Maryury Colina; María Belén Rodríguez Cardozo; María Fernanda Yaunguzian; Miriam Bruno; Nabaes Jodar; Natale; Natalia Chiussi; Natasha Frisone; Noelia Anzorena; Noelia Narduzzi; Nuria Cañellas Walter Loayza; Rocío Carrón; Romina Gatica; S; Silvia Matillas; Valinotto; Viegas; Viegas, M.; Yesica Castro; Yesica Castro. Promoción y Protección de la Salud: Miriam Bruno
EPI_ISL_1394925, EPI_ISL_1394926, EPI_ISL_1394927, EPI_ISL_1394928, EPI_ISL_1394930, EPI_ISL_1394931, EPI_ISL_1394933, EPI_ISL_1394934, EPI_ISL_1394935, EPI_ISL_1395055, EPI_ISL_1395073, EPI_ISL_1395074, EPI_ISL_1395075, EPI_ISL_1395076, EPI_ISL_1395077, EPI_ISL_1395092, EPI_ISL_1395093, EPI_ISL_1395094, EPI_ISL_1395095, EPI_ISL_1395096, EPI_ISL_1395097, EPI_ISL_1395098, EPI_ISL_1395099, EPI_ISL_1395100, EPI_ISL_1395101, EPI_ISL_1395115, EPI_ISL_1395174, EPI_ISL_1395175, EPI_ISL_1395176			
see above	Laboratorio de Diagnóstico de la UNAHUR	Área de Secuenciación del Laboratorio de Virología del Hospital de Niños Dr. Ricardo Gutierrez on behalf of 'Proyecto Argentino Interinstitucional de genomica de SARS-CoV-2' (PAIS Consortium)	Acuña; Adriana Fernandez Souto; Aldana Trotta; Alexay; Angel German Arias; Angélica María Ramirez Londoño; Blanc Daiana Sofia; Boero Carolina Jazmín; Brenda Gimeno; Camila Frydman; Cristina Belén Serrano; D; Daniela Vega; David Ybarra; Didier Garnham Mercedes; Germán Albornoz; Goya; Juan Manuel Velazquez; LE; Luis Castillo; Lusso; M; MI; Marcela Pilloff; Maria Natalia Calienni; Maria del C Lorenzo; Marina Mozgovoj; María José Dus Santos; Nabaes Jodar; Natale; Pablo Raies; S; Sabrina Amalfi; Valeria Marsal; Valinotto; Vanina Saraullo; Viegas, M.
EPI_ISL_3455896	Laboratorio de Diagnóstico-UNIDAD COVID- Universidad Nacional de Hurlingham	Departamento de Ciencias Básicas – Universidad Nacional de Luján y Unidad de Genómica – Instituto Nacional de Tecnología Agropecuaria on behalf of 'Proyecto Argentino Interinstitucional de genomica de SARS-CoV-2' (PAIS Consortium)	Andrea Puebla.; Anna Belén Calloni; Belén Ubaldo; Carla Antonella Massone; Elizabeth Joyce Maleplate; Flavia Noelia Minutto; Gabriela Elena Zunino; Guido König; José Jaramillo Ortiz; Leandro García; Marco Cacciabue; Marianne Muñoz; María Belén Mónaco; María Inés Gismondi; Paula del Carmen Fernandez; Sebastián Gabriel Zunino; Sofia Bengoa Luoni
EPI_ISL_1597827, EPI_ISL_1597828, EPI_ISL_1597829, EPI_ISL_1597830, EPI_ISL_1597831, EPI_ISL_1597832, EPI_ISL_1597833, EPI_ISL_1597834, EPI_ISL_1597835, EPI_ISL_1597836, EPI_ISL_1597837, EPI_ISL_1597838, EPI_ISL_1597839, EPI_ISL_1597840, EPI_ISL_1597841, EPI_ISL_1597842, EPI_ISL_1597843, EPI_ISL_1597844, EPI_ISL_1597845, EPI_ISL_1597846, EPI_ISL_1597847, EPI_ISL_1597848			
see above	Laboratorio de Diagnóstico-UNIDAD COVID- Universidad Nacional de Hurlingham	Laboratorio de Diagnóstico-UNIDAD COVID- Universidad Nacional de Hurlingham y Unidad de Genómica – Instituto Nacional de Tecnología Agropecuaria on behalf of 'Proyecto Argentino Interinstitucional de genomica de SARS-CoV-2' (PAIS Consortium)	Adriana Fernández Souto; Andrea Puebla.; Angel Arias; Angélica M Ramirez; Camila Frydman; Carolina Jazmín Boero; Cristina Belén Serrano; Daiana Sofia Blanc; Daniela Vega; David Ybarra; Didier Garnham Mercedes; Germán Albornoz; Guido König; Juan Manuel Velazquez; Luis Castillo; Marcela Pilloff; Marco Cacciabue; Marianne Muñoz; Marina Mozgovoj; Marisa Lorenzo; María José Dus Santos; Natalia Calienni; Pablo Raies; Paula del Carmen Fernandez; Sabrina Amalfi; Sofia Bengoa Luoni; Valeria Marsal; Vanina Saraullo
EPI_ISL_2454389, EPI_ISL_2454390, EPI_ISL_2454391, EPI_ISL_2454392, EPI_ISL_2454393, EPI_ISL_2454394, EPI_ISL_2454395, EPI_ISL_2454396, EPI_ISL_2454397, EPI_ISL_2454398, EPI_ISL_2454399, EPI_ISL_2454400, EPI_ISL_2454401, EPI_ISL_2454402, EPI_ISL_2454403, EPI_ISL_2454404, EPI_ISL_2454405, EPI_ISL_2454406, EPI_ISL_2454407, EPI_ISL_2454408, EPI_ISL_2454437, EPI_ISL_2454438, EPI_ISL_2454439, EPI_ISL_2454440, EPI_ISL_2454441, EPI_ISL_2454442, EPI_ISL_2454443, EPI_ISL_2454444, EPI_ISL_2454445, EPI_ISL_2454446, EPI_ISL_2454447, EPI_ISL_2454448, EPI_ISL_2454449, EPI_ISL_2454450, EPI_ISL_2454451, EPI_ISL_2454452, EPI_ISL_2454453, EPI_ISL_2454454, EPI_ISL_2454455, EPI_ISL_2454456, EPI_ISL_2454457, EPI_ISL_2454458, EPI_ISL_2454459, EPI_ISL_2454460, EPI_ISL_2454461, EPI_ISL_2454472, EPI_ISL_2454473, EPI_ISL_2454474, EPI_ISL_2454475, EPI_ISL_2454476, EPI_ISL_2454477, EPI_ISL_2454478, EPI_ISL_2454479, EPI_ISL_2454480, EPI_ISL_2454481, EPI_ISL_2454482, EPI_ISL_2454483, EPI_ISL_2454484, EPI_ISL_2454485, EPI_ISL_2454486, EPI_ISL_2454487, EPI_ISL_2454488, EPI_ISL_2454489, EPI_ISL_2454490, EPI_ISL_2454491, EPI_ISL_2454492, EPI_ISL_2454493, EPI_ISL_2454494, EPI_ISL_2454495, EPI_ISL_2454496, EPI_ISL_2454497, EPI_ISL_2454498, EPI_ISL_2454499, EPI_ISL_2454500, EPI_ISL_2454501, EPI_ISL_2454502, EPI_ISL_2454503, EPI_ISL_2454504, EPI_ISL_2454505, EPI_ISL_2454506, EPI_ISL_2454507, EPI_ISL_2454508, EPI_ISL_2454509, EPI_ISL_2454510, EPI_ISL_3455862, EPI_ISL_3455863, EPI_ISL_3455864, EPI_ISL_3455880, EPI_ISL_3455881, EPI_ISL_3455895, EPI_ISL_3455897, EPI_ISL_3455898, EPI_ISL_3455899, EPI_ISL_3455900, EPI_ISL_3455901, EPI_ISL_3455902, EPI_ISL_3455903, EPI_ISL_3455904, EPI_ISL_3455905, EPI_ISL_3455906, EPI_ISL_3455907, EPI_ISL_3455908, EPI_ISL_3455909, EPI_ISL_3455910, EPI_ISL_3455911, EPI_ISL_3455912, EPI_ISL_3455913, EPI_ISL_3455914, EPI_ISL_3455915, EPI_ISL_3455916, EPI_ISL_3455917, EPI_ISL_3455918, EPI_ISL_3455919, EPI_ISL_3455920, EPI_ISL_3455921, EPI_ISL_3455922, EPI_ISL_3455923, EPI_ISL_3455924, EPI_ISL_3455925, EPI_ISL_3455926, EPI_ISL_3455927, EPI_ISL_3455928, EPI_ISL_3455929, EPI_ISL_3455930, EPI_ISL_3455931, EPI_ISL_3455932, EPI_ISL_3455933, EPI_ISL_3455934, EPI_ISL_3455935, EPI_ISL_3455936, EPI_ISL_3455937, EPI_ISL_3455938, EPI_ISL_3455939, EPI_ISL_3455940, EPI_ISL_3455941, EPI_ISL_3455942, EPI_ISL_3455943, EPI_ISL_3455944, EPI_ISL_3455945, EPI_ISL_3455946, EPI_ISL_3455947, EPI_ISL_3455948, EPI_ISL_3455949, EPI_ISL_3455950, EPI_ISL_3455951, EPI_ISL_3455952, EPI_ISL_3455953, EPI_ISL_3455954, EPI_ISL_3455955, EPI_ISL_3455956, EPI_ISL_3455957, EPI_ISL_3455958, EPI_ISL_3455959, EPI_ISL_3455960, EPI_ISL_3455961, EPI_ISL_3455962, EPI_ISL_3455963, EPI_ISL_3455964, EPI_ISL_3455965, EPI_ISL_3455966, EPI_ISL_3455967, EPI_ISL_3455968, EPI_ISL_3455969, EPI_ISL_3455970, EPI_ISL_3455971, EPI_ISL_3455977, EPI_ISL_3456014, EPI_ISL_3456015, EPI_ISL_3456016, EPI_ISL_3456017, EPI_ISL_3456018, EPI_ISL_3456019, EPI_ISL_3478826, EPI_ISL_3478828			
see above	Laboratorio de Diagnóstico-UNIDAD COVID- Universidad Nacional de Hurlingham	Laboratorio de Diagnóstico-UNIDAD COVID- Universidad Nacional de Hurlingham y Unidad de Genómica – Instituto Nacional de Tecnología Agropecuaria on behalf of 'Proyecto Argentino Interinstitucional de genomica de SARS-CoV-2' (PAIS Consortium)	Adriana Fernández Souto; Andrea Puebla.; Angel Arias; Angélica M Ramirez; Camila Frydman; Carolina Jazmín Boero; Cristina Belén Serrano; Daiana Sofia Blanc; Daniela Vega; David Ybarra; Didier Garnham Mercedes; Germán Albornoz; Guido König; Juan Manuel Velazquez; Luis Castillo; Marcela Pilloff; Marco Cacciabue; Marianne Muñoz; Marina Mozgovoj; Marisa Lorenzo; María José Dus Santos; Natalia Calienni; Pablo Raies; Paula del Carmen Fernandez; Sabrina Amalfi; Sofia Bengoa Luoni; Valeria Marsal; Vanina Saraullo
EPI_ISL_1395598, EPI_ISL_1395600, EPI_ISL_2449114, EPI_ISL_2449124, EPI_ISL_2449125, EPI_ISL_2449131, EPI_ISL_2449143, EPI_ISL_2449164, EPI_ISL_2449187, EPI_ISL_2449188, EPI_ISL_2449201, EPI_ISL_2449266, EPI_ISL_2449674, EPI_ISL_2449741, EPI_ISL_2449762, EPI_ISL_2449763, EPI_ISL_2449764, EPI_ISL_2449765, EPI_ISL_2449766, EPI_ISL_2449785, EPI_ISL_2449803, EPI_ISL_2449847, EPI_ISL_2449848, EPI_ISL_2449849			
see above	Laboratorio de Hospital El Cruce Dr. Néstor C. Kirchner	Área de Secuenciación del Laboratorio de Virología del Hospital de Niños Dr. Ricardo Gutierrez on behalf of 'Proyecto Argentino Interinstitucional de genomica de SARS-CoV-2' (PAIS Consortium)	Acuña; Alexay; D; Goya; LE; Lusso; M; MI; Marilina Rahhal; Martin Zubieta; Nabaes Jodar; Natale; S; Valinotto; Viegas, M.
EPI_ISL_2449007, EPI_ISL_2449008, EPI_ISL_2449009, EPI_ISL_2449010, EPI_ISL_2449011, EPI_ISL_2449012, EPI_ISL_2449013, EPI_ISL_2449014, EPI_ISL_2449015, EPI_ISL_2449329, EPI_ISL_2449330, EPI_ISL_2449331, EPI_ISL_2449332, EPI_ISL_2449349, EPI_ISL_2449350, EPI_ISL_2449351, EPI_ISL_2449352, EPI_ISL_2449373, EPI_ISL_2449374, EPI_ISL_2449375, EPI_ISL_2449386, EPI_ISL_2449387, EPI_ISL_2449388, EPI_ISL_2449389, EPI_ISL_2449390, EPI_ISL_2449391, EPI_ISL_2449392, EPI_ISL_2449393, EPI_ISL_2449409			
see above	Laboratorio de Salud Pública "Dr Dalmiro Pérez Laborda"	Área de Secuenciación del Laboratorio de Virología del Hospital de Niños Dr. Ricardo Gutierrez on behalf of 'Proyecto Argentino Interinstitucional de genomica de SARS-CoV-2' (PAIS Consortium)	ACH; Acuña; Aguilera; Alexay; BE; Bohn; Cabral Bombardieri; Campos; Carrizo; D; E; EM; Esteves; F; G; Goya; J; JM; JP; Jofré; L; LE; Lacaze; Lusso; M; MA; MI; Mastrodonato; Molina; Nabaes Jodar; Natale; Olivera; Perez Diaz; Peñalva; Quijano; Rivero; Rosales; S; Talia; Valinotto; Viegas, M.
EPI_ISL_1394932, EPI_ISL_1394952, EPI_ISL_1394953, EPI_ISL_1395070, EPI_ISL_1395078, EPI_ISL_1395079, EPI_ISL_1395102, EPI_ISL_1395103, EPI_ISL_1395116, EPI_ISL_1395130, EPI_ISL_1395131, EPI_ISL_1395145, EPI_ISL_1395146, EPI_ISL_1395147, EPI_ISL_1395148, EPI_ISL_1395178, EPI_ISL_1395185, EPI_ISL_1395186, EPI_ISL_1395199, EPI_ISL_1395200, EPI_ISL_1395201, EPI_ISL_1395202, EPI_ISL_2449525, EPI_ISL_2449526, EPI_ISL_2449527, EPI_ISL_2449548, EPI_ISL_2449549, EPI_ISL_2449550, EPI_ISL_2449564, EPI_ISL_2449570, EPI_ISL_2449577, EPI_ISL_2449578, EPI_ISL_2449579, EPI_ISL_2449580, EPI_ISL_2449584, EPI_ISL_2449585, EPI_ISL_2449586, EPI_ISL_2449597, EPI_ISL_2449598, EPI_ISL_2449599, EPI_ISL_2449614, EPI_ISL_2449615, EPI_ISL_2449616, EPI_ISL_2449617, EPI_ISL_2449618, EPI_ISL_2449619, EPI_ISL_2449620, EPI_ISL_2449640, EPI_ISL_2449641, EPI_ISL_2449642, EPI_ISL_2449643, EPI_ISL_2449644, EPI_ISL_2449670, EPI_ISL_2449671, EPI_ISL_2449672, EPI_ISL_2449673, EPI_ISL_2449689, EPI_ISL_2454378, EPI_ISL_3277900, EPI_ISL_3278230, EPI_ISL_3278231, EPI_ISL_3278232, EPI_ISL_3278233, EPI_ISL_3278265, EPI_ISL_3278266, EPI_ISL_3278267, EPI_ISL_3278268, EPI_ISL_3278269, EPI_ISL_3278270, EPI_ISL_3278271, EPI_ISL_3278272, EPI_ISL_3278273, EPI_ISL_3278274, EPI_ISL_3278275, EPI_ISL_3278276, EPI_ISL_3278277, EPI_ISL_3278278			
see above	Laboratorio de Virologia - HIEAyC "San Juan de Dios"	Área de Secuenciación del Laboratorio de Virología del Hospital de Niños Dr. Ricardo Gutierrez on behalf of 'Proyecto Argentino Interinstitucional de genomica de SARS-CoV-2' (PAIS Consortium)	Acuña; Alexay; Andrea Gatelli; D; Francisco Echeverria; Goya; Karina Gil; LE; Lusso; M; MI; Maria Colmeiro; Martina Ferioli; Nabaes Jodar; Natale; Ramiro Agüero; Regina Ercole; S; Silvia Galvez; Valinotto; Viegas, M.
EPI_ISL_5656742, EPI_ISL_5656743, EPI_ISL_5656744, EPI_ISL_5656745, EPI_ISL_5656746, EPI_ISL_5656747, EPI_ISL_5656748, EPI_ISL_5656749, EPI_ISL_5656750, EPI_ISL_5656751, EPI_ISL_5656752, EPI_ISL_5656753, EPI_ISL_5656754, EPI_ISL_5656755, EPI_ISL_5657078, EPI_ISL_5657079, EPI_ISL_5657080, EPI_ISL_5657081, EPI_ISL_5657082, EPI_ISL_5657083, EPI_ISL_5657084, EPI_ISL_5657085, EPI_ISL_5657086, EPI_ISL_5657087, EPI_ISL_5657199, EPI_ISL_5657200, EPI_ISL_5657201, EPI_ISL_5657202, EPI_ISL_5657203, EPI_ISL_5657204, EPI_ISL_5657205, EPI_ISL_5657206, EPI_ISL_5657207, EPI_ISL_5657208, EPI_ISL_5657209, EPI_ISL_5657741, EPI_ISL_5657746, EPI_ISL_5657747, EPI_ISL_5657755, EPI_ISL_5657756, EPI_ISL_5657760, EPI_ISL_5657766			

see above	Laboratorio de Virologia, HIEAyC "San Juan de Dios"	Área de Secuenciación del Laboratorio de Virología del Hospital de Niños Dr. Ricardo Gutierrez on behalf of 'Proyecto Argentino Interinstitucional de genomica de SARS-CoV-2' (PAIS Consortium)	Acuña; Alexay; Angeletti Andres; Colmeiro Maria; D; Di Bella Sofia; Ercole Regina; Ferioli Martina; Gatelli Andrea; Goya; LE; Lusso; M; MI; Malaissi Luciano; Martinez Agustina; Nabaes Jodar; Natale; S; Valinotto; Viegas
EPI_ISL_1597783, EPI_ISL_1597784, EPI_ISL_1597785, EPI_ISL_1597786, EPI_ISL_1597787, EPI_ISL_1597788, EPI_ISL_1597789, EPI_ISL_1597790, EPI_ISL_1597791, EPI_ISL_1597792, EPI_ISL_1597793, EPI_ISL_1597794, EPI_ISL_1597795, EPI_ISL_1597796, EPI_ISL_1597797, EPI_ISL_1597798, EPI_ISL_1597799, EPI_ISL_1597800, EPI_ISL_1597801, EPI_ISL_1597802, EPI_ISL_1597803, EPI_ISL_1597804, EPI_ISL_1597805, EPI_ISL_1597806, EPI_ISL_1597807, EPI_ISL_1597808, EPI_ISL_1597809, EPI_ISL_1597810, EPI_ISL_1597811, EPI_ISL_1597812, EPI_ISL_1597813, EPI_ISL_1597814, EPI_ISL_1597815, EPI_ISL_1597816, EPI_ISL_1597817, EPI_ISL_1597818, EPI_ISL_1597819, EPI_ISL_1597820, EPI_ISL_1597821, EPI_ISL_1597822, EPI_ISL_1597823, EPI_ISL_1597824, EPI_ISL_1597825, EPI_ISL_1597826, EPI_ISL_2454380, EPI_ISL_2454381, EPI_ISL_2454382, EPI_ISL_2454383, EPI_ISL_2454384, EPI_ISL_2454385, EPI_ISL_2454386, EPI_ISL_2454387, EPI_ISL_2454388, EPI_ISL_2454409, EPI_ISL_2454410, EPI_ISL_2454411, EPI_ISL_2454412, EPI_ISL_2454413, EPI_ISL_2454414, EPI_ISL_2454415, EPI_ISL_2454416, EPI_ISL_2454417, EPI_ISL_2454418, EPI_ISL_2454419, EPI_ISL_2454420, EPI_ISL_2454421, EPI_ISL_2454422, EPI_ISL_2454423, EPI_ISL_2454424, EPI_ISL_2454425, EPI_ISL_2454426, EPI_ISL_2454427, EPI_ISL_2454428, EPI_ISL_2454429, EPI_ISL_2454430, EPI_ISL_2454431, EPI_ISL_2454432, EPI_ISL_2454433, EPI_ISL_2454434, EPI_ISL_2454435, EPI_ISL_2454436, EPI_ISL_2454462, EPI_ISL_2454463, EPI_ISL_2454464, EPI_ISL_2454465, EPI_ISL_2454466, EPI_ISL_2454467, EPI_ISL_2454468, EPI_ISL_2454469, EPI_ISL_2454470, EPI_ISL_2454471, EPI_ISL_3455865, EPI_ISL_3455866, EPI_ISL_3455867, EPI_ISL_3455868, EPI_ISL_3455869, EPI_ISL_3455870, EPI_ISL_3455871, EPI_ISL_3455872, EPI_ISL_3455873, EPI_ISL_3455874, EPI_ISL_3455875, EPI_ISL_3455876, EPI_ISL_3455877, EPI_ISL_3455878, EPI_ISL_3455879, EPI_ISL_3455882, EPI_ISL_3455883, EPI_ISL_3455884, EPI_ISL_3455885, EPI_ISL_3455886, EPI_ISL_3455887, EPI_ISL_3455888, EPI_ISL_3455889, EPI_ISL_3455890, EPI_ISL_3455891, EPI_ISL_3455892, EPI_ISL_3455893, EPI_ISL_3455894, EPI_ISL_3455972, EPI_ISL_3455973, EPI_ISL_3455974, EPI_ISL_3455975, EPI_ISL_3455976, EPI_ISL_3455978, EPI_ISL_3455979, EPI_ISL_3455980, EPI_ISL_3455981, EPI_ISL_3455982, EPI_ISL_3455983, EPI_ISL_3455984, EPI_ISL_3455985, EPI_ISL_3455986, EPI_ISL_3455987, EPI_ISL_3455988, EPI_ISL_3455989, EPI_ISL_3455990, EPI_ISL_3455991, EPI_ISL_3455992, EPI_ISL_3455993, EPI_ISL_3455994, EPI_ISL_3455995, EPI_ISL_3455996, EPI_ISL_3455997, EPI_ISL_3455998, EPI_ISL_3455999, EPI_ISL_3456000, EPI_ISL_3456001, EPI_ISL_3456002, EPI_ISL_3456003, EPI_ISL_3456004, EPI_ISL_3456005, EPI_ISL_3456006, EPI_ISL_3456007, EPI_ISL_3456008, EPI_ISL_3456009, EPI_ISL_3456010, EPI_ISL_3456011, EPI_ISL_3456012, EPI_ISL_3456013, EPI_ISL_3456020, EPI_ISL_3456021, EPI_ISL_3456022, EPI_ISL_3456023, EPI_ISL_3456024, EPI_ISL_3456025, EPI_ISL_3456026, EPI_ISL_3456027, EPI_ISL_3456028, EPI_ISL_3478827			
see above	Laboratorio de Virología Molecular – Hospital Blas L. Dubarry	Departamento de Ciencias Básicas – Universidad Nacional de Luján y Unidad de Genómica – Instituto Nacional de Tecnología Agropecuaria on behalf of 'Proyecto Argentino Interinstitucional de genomica de SARS-CoV-2' (PAIS Consortium)	Andrea Puebla.; Anna Belén Calloni; Belén Ubaldo; Carla Antonella Massone; Elizabeth Joyce Maleplate; Flavia Noelia Minutto; Gabriela Elena Zunino; Guido König; José Jaramillo Ortiz; Leandro García; Marco Cacciabue; Marianne Muñoz; María Belén Mónaco; María Inés Gismondi; Paula del Carmen Fernandez; Sebastián Gabriel Zunino; Sofia Bengoa Luoni
EPI_ISL_2469471	Laboratorio de Virología Molecular – Hospital Blas L. Dubarry	National Institute of Agronomic Technologies	Andrea Puebla.; Anna Belén Calloni; Belén Ubaldo; Carla Antonella Massone; Elizabeth Joyce Maleplate; Flavia Noelia Minutto; Gabriela Elena Zunino; Guido König; José Jaramillo Ortiz; Leandro García; Marco Cacciabue; Marianne Muñoz; María Belén Mónaco; María Inés Gismondi; Paula del Carmen Fernandez; Sebastián Gabriel Zunino; Sofia Bengoa Luoni
EPI_ISL_1394913, EPI_ISL_1395251	Laboratorio de Virología Molecular – Hospital Blas L. Dubarry y Departamento de Ciencias Básicas – Universidad Nacional de Luján	Área de Secuenciación del Laboratorio de Virología del Hospital de Niños Dr. Ricardo Gutierrez on behalf of 'Proyecto Argentino Interinstitucional de genomica de SARS-CoV-2' (PAIS Consortium)	Acuña; Alexay; Anna Belén Calloni; Belén Ubaldo; Carla Antonella Massone; D; Elizabeth Joyce Maleplate; Flavia Noelia Minutto; Gabriela Elena Zunino; Goya; José Jaramillo Ortiz; LE; Leandro García; Lusso; M; MI; María Belén Mónaco; María Inés Gismondi; Nabaes Jodar; Natale; S; Sebastián Gabriel Zunino; Valinotto; Viegas, M.
EPI_ISL_2449344, EPI_ISL_2449345, EPI_ISL_2449363, EPI_ISL_2449364, EPI_ISL_2449365, EPI_ISL_2449403, EPI_ISL_2449404, EPI_ISL_2449405, EPI_ISL_5656999, EPI_ISL_5657000, EPI_ISL_5657001, EPI_ISL_5657002, EPI_ISL_5657003, EPI_ISL_5657004, EPI_ISL_5657005, EPI_ISL_5657006			
see above	Laboratorio de Virología de la Facultad de Veterinaria de la Universidad Nacional del Centro de la provincia de Buenos Aires (UNCPBA)	Área de Secuenciación del Laboratorio de Virología del Hospital de Niños Dr. Ricardo Gutierrez on behalf of 'Proyecto Argentino Interinstitucional de genomica de SARS-CoV-2' (PAIS Consortium)	Acuña; Alexay; C; Carolina Ceriani; Ceriani; D; Dolcini; G; Goya; Guillermina Dolcini; LE; Lusso; M; MI; Nabaes Jodar; Natale; Nieto Farias; Perez; S; Sandra Perez; V; Valinotto; Victoria Nieto Farias; Viegas; Viegas, M.
EPI_ISL_2449027, EPI_ISL_2449028, EPI_ISL_2449029, EPI_ISL_2449030, EPI_ISL_2449031, EPI_ISL_2449038, EPI_ISL_2449039, EPI_ISL_2449040, EPI_ISL_2449041, EPI_ISL_2449049, EPI_ISL_2449050, EPI_ISL_2449051, EPI_ISL_2449052, EPI_ISL_2449053, EPI_ISL_2449059, EPI_ISL_2449060, EPI_ISL_2449061, EPI_ISL_2449062, EPI_ISL_2449063, EPI_ISL_2449073, EPI_ISL_2449074, EPI_ISL_2449075, EPI_ISL_2449076, EPI_ISL_2449077, EPI_ISL_5657210, EPI_ISL_5657211, EPI_ISL_5657212, EPI_ISL_5657213, EPI_ISL_5657214, EPI_ISL_5657215, EPI_ISL_5657216, EPI_ISL_5657217			
see above	Laboratorio de Virología del Hospital JJ Urquiza	Área de Secuenciación del Laboratorio de Virología del Hospital de Niños Dr. Ricardo Gutierrez on behalf of 'Proyecto Argentino Interinstitucional de genomica de SARS-CoV-2' (PAIS Consortium)	; A; Acuña; Agustina Bonnet; Alejandra Lezica; Alexay; Anabella Gimenez; Arca; B; Betiana Paucar; Bonnet; Carrocino; Cerutti; D; F; Facundo Punzi; Fernando Gadea; Fick; Florencia Garelli; Florencia Rodríguez; Florencia Rojas; Francisco Arca; Gadea; Garelli; Gimenez; Giraldes; Goya; J; Jessica Carrocino; L; LE; Lezica; Luis Fick; Lusso; M; MI; Magalí Rolleri; Manuel Arca; Marlene Giraldes; Nabaes Jodar; Natale; Paucar; Punzi; Rodríguez; Rojas; Rolleri; S; Sabrina Cerutti; Valinotto; Viegas
EPI_ISL_1394870, EPI_ISL_1394871, EPI_ISL_1394872, EPI_ISL_1394873, EPI_ISL_1394874, EPI_ISL_1394875, EPI_ISL_1394876, EPI_ISL_1394877, EPI_ISL_1394878, EPI_ISL_1394879, EPI_ISL_1394880, EPI_ISL_1394881, EPI_ISL_1394882, EPI_ISL_1394883, EPI_ISL_1394884, EPI_ISL_1394886, EPI_ISL_1394887, EPI_ISL_1394888, EPI_ISL_1394889, EPI_ISL_1394890, EPI_ISL_1394891, EPI_ISL_1394892, EPI_ISL_1394893, EPI_ISL_1394894, EPI_ISL_1394895, EPI_ISL_1394896, EPI_ISL_1394897, EPI_ISL_1394898, EPI_ISL_1394899, EPI_ISL_1394900, EPI_ISL_1394901, EPI_ISL_1394902, EPI_ISL_1394903, EPI_ISL_1394904, EPI_ISL_1394905, EPI_ISL_1394906, EPI_ISL_1394907, EPI_ISL_1394908, EPI_ISL_1394911, EPI_ISL_1394912, EPI_ISL_1394941, EPI_ISL_1394942, EPI_ISL_1394943, EPI_ISL_1394944, EPI_ISL_1394947, EPI_ISL_1394950, EPI_ISL_1394951, EPI_ISL_1394954, EPI_ISL_1394955, EPI_ISL_1394956, EPI_ISL_1394957, EPI_ISL_1394958, EPI_ISL_1394959, EPI_ISL_1394960, EPI_ISL_1394961, EPI_ISL_1394962, EPI_ISL_1394963, EPI_ISL_1394964, EPI_ISL_1394965, EPI_ISL_1394966, EPI_ISL_1394967, EPI_ISL_1394968, EPI_ISL_1394969, EPI_ISL_1394970, EPI_ISL_1394971, EPI_ISL_1394973, EPI_ISL_1394974, EPI_ISL_1394975, EPI_ISL_1394976, EPI_ISL_1394977, EPI_ISL_1394978, EPI_ISL_1394979, EPI_ISL_1394980, EPI_ISL_1394981, EPI_ISL_1394982, EPI_ISL_1394983, EPI_ISL_1394984, EPI_ISL_1394986, EPI_ISL_1394987, EPI_ISL_1394988, EPI_ISL_1394989, EPI_ISL_1394990, EPI_ISL_1394991, EPI_ISL_1394992, EPI_ISL_1394993, EPI_ISL_1394994, EPI_ISL_1394995, EPI_ISL_1394996, EPI_ISL_1394997, EPI_ISL_1395001, EPI_ISL_1395002, EPI_ISL_1395003, EPI_ISL_1395004, EPI_ISL_1395005, EPI_ISL_1395006, EPI_ISL_1395007, EPI_ISL_1395008, EPI_ISL_1395009, EPI_ISL_1395010, EPI_ISL_1395011, EPI_ISL_1395012, EPI_ISL_1395013, EPI_ISL_1395014, EPI_ISL_1395015, EPI_ISL_1395016, EPI_ISL_1395021, EPI_ISL_1395022, EPI_ISL_1395023, EPI_ISL_1395024, EPI_ISL_1395025, EPI_ISL_1395026, EPI_ISL_1395027, EPI_ISL_1395028, EPI_ISL_1395029, EPI_ISL_1395030, EPI_ISL_1395031, EPI_ISL_1395032, EPI_ISL_1395033, EPI_ISL_1395034, EPI_ISL_1395035, EPI_ISL_1395042, EPI_ISL_1395043, EPI_ISL_1395044, EPI_ISL_1395045, EPI_ISL_1395046, EPI_ISL_1395047, EPI_ISL_1395048, EPI_ISL_1395049, EPI_ISL_1395050, EPI_ISL_1395051, EPI_ISL_1395056, EPI_ISL_1395057, EPI_ISL_1395058, EPI_ISL_1395059, EPI_ISL_1395060, EPI_ISL_1395061, EPI_ISL_1395062, EPI_ISL_1395063, EPI_ISL_1395064, EPI_ISL_1395065, EPI_ISL_1395066, EPI_ISL_1395071, EPI_ISL_1395080, EPI_ISL_1395081, EPI_ISL_1395082, EPI_ISL_1395083, EPI_ISL_1395084, EPI_ISL_1395085, EPI_ISL_1395086, EPI_ISL_1395087, EPI_ISL_1395088, EPI_ISL_1395089, EPI_ISL_1395090, EPI_ISL_1395091, EPI_ISL_1395104, EPI_ISL_1395105, EPI_ISL_1395106, EPI_ISL_1395107, EPI_ISL_1395108, EPI_ISL_1395109, EPI_ISL_1395110, EPI_ISL_1395111, EPI_ISL_1395112, EPI_ISL_1395113, EPI_ISL_1395114, EPI_ISL_1395117, EPI_ISL_1395118, EPI_ISL_1395119, EPI_ISL_1395120, EPI_ISL_1395121, EPI_ISL_1395122, EPI_ISL_1395132, EPI_ISL_1395133, EPI_ISL_1395134, EPI_ISL_1395135, EPI_ISL_1395136, EPI_ISL_1395137, EPI_ISL_1395138, EPI_ISL_1395139, EPI_ISL_1395149, EPI_ISL_1395150, EPI_ISL_1395151, EPI_ISL_1395152, EPI_ISL_1395153, EPI_ISL_1395154, EPI_ISL_1395155, EPI_ISL_1395156, EPI_ISL_1395157, EPI_ISL_1395158, EPI_ISL_1395159, EPI_ISL_1395167, EPI_ISL_1395168, EPI_ISL_1395169, EPI_ISL_1395170, EPI_ISL_1395171, EPI_ISL_1395172, EPI_ISL_1395173, EPI_ISL_1395179, EPI_ISL_1395180, EPI_ISL_1395181, EPI_ISL_1395182, EPI_ISL_1395183, EPI_ISL_1395184, EPI_ISL_1395187, EPI_ISL_1395188, EPI_ISL_1395189, EPI_ISL_1395190, EPI_ISL_1395191, EPI_ISL_1395192, EPI_ISL_1395193, EPI_ISL_1395198, EPI_ISL_1395203, EPI_ISL_1395204, EPI_ISL_1395205, EPI_ISL_1395206, EPI_ISL_1395207, EPI_ISL_1395208, EPI_ISL_1395209, EPI_ISL_1395210, EPI_ISL_1395211, EPI_ISL_1395212, EPI_ISL_1395213, EPI_ISL_1395218, EPI_ISL_1395219, EPI_ISL_1395220, EPI_ISL_1395221, EPI_ISL_1395222, EPI_ISL_1395223, EPI_ISL_1395224, EPI_ISL_1395225, EPI_ISL_1395226, EPI_ISL_1395227, EPI_ISL_1395228, EPI_ISL_1395229, EPI_ISL_1395230, EPI_ISL_1395231, EPI_ISL_1395232, EPI_ISL_1395233, EPI_ISL_1395234, EPI_ISL_1395235, EPI_ISL_1395236, EPI_ISL_1395237, EPI_ISL_1395238, EPI_ISL_1395239, EPI_ISL_1395240, EPI_ISL_1395241, EPI_ISL_1395242, EPI_ISL_1395243, EPI_ISL_1395244, EPI_ISL_1395245, EPI_ISL_1395246, EPI_ISL_1395247, EPI_ISL_1395248, EPI_ISL_1395249, EPI_ISL_1395250, EPI_ISL_1395252, EPI_ISL_1395253, EPI_ISL_1395254, EPI_ISL_1395255, EPI_ISL_1395256, EPI_ISL_1395257, EPI_ISL_1395258, EPI_ISL_1395259, EPI_ISL_1395260, EPI_ISL_1395261, EPI_ISL_1395262, EPI_ISL_1395263, EPI_ISL_1395264, EPI_ISL_1395265, EPI_ISL_1395266, EPI_ISL_1395267, EPI_ISL_1395268, EPI_ISL_1395269, EPI_ISL_1395270, EPI_ISL_1395271, EPI_ISL_1395272, EPI_ISL_1395273, EPI_ISL_1395274, EPI_ISL_1395275, EPI_ISL_1395276, EPI_ISL_1395277, EPI_ISL_1395278, EPI_ISL_1395279, EPI_ISL_1395280, EPI_ISL_1395285, EPI_ISL_1395286, EPI_ISL_1395287, EPI_ISL_1395288, EPI_ISL_1395289, EPI_ISL_1395290, EPI_ISL_1395291, EPI_ISL_1395292, EPI_ISL_1395293, EPI_ISL_1395294, EPI_ISL_1395295, EPI_ISL_1395296, EPI_ISL_1395297, EPI_ISL_1395298, EPI_ISL_1395299, EPI_ISL_1395300, EPI_ISL_1395301, EPI_ISL_1395302, EPI_ISL_1395303, EPI_ISL_1395304, EPI_ISL_1395305, EPI_ISL_1395306, EPI_ISL_1395307, EPI_ISL_1395308, EPI_ISL_1395309, EPI_ISL_1395310, EPI_ISL_1395311, EPI_ISL_1395312, EPI_ISL_1395313, EPI_ISL_1395314, EPI_ISL_1395315, EPI_ISL_1395316, EPI_ISL_1395317, EPI_ISL_1395319, EPI_ISL_1395320, EPI_ISL_1395321, EPI_ISL_1395322, EPI_ISL_1395323, EPI_ISL_1395324, EPI_ISL_1395325, EPI_ISL_1395326, EPI_ISL_1395327, EPI_ISL_1395328, EPI_ISL_1395329, EPI_ISL_1395331, EPI_ISL_1395332, EPI_ISL_1395333, EPI_ISL_1395334, EPI_ISL_1395335, EPI_ISL_1395336, EPI_ISL_1395337, EPI_ISL_1395338, EPI_ISL_1395339, EPI_ISL_1395350, EPI_ISL_1395351, EPI_ISL_1395352, EPI_ISL_1395353, EPI_ISL_1395354, EPI_ISL_1395355, EPI_ISL_1395362, EPI_ISL_1395363, EPI_ISL_1395364, EPI_ISL_1395365, EPI_ISL_1395369, EPI_ISL_1395370, EPI_ISL_1395371, EPI_ISL_1395372, EPI_ISL_1395382, EPI_ISL_1395383, EPI_ISL_1395384, EPI_ISL_1395390, EPI_ISL_1395391, EPI_ISL_1395393, EPI_ISL_1395398, EPI_ISL_1395399, EPI_ISL_1395400, EPI_ISL_1395401, EPI_ISL_1395402, EPI_ISL_1395403, EPI_ISL_1395404, EPI_ISL_1395408, EPI_ISL_1395409, EPI_ISL_1395410, EPI_ISL_1395411, EPI_ISL_1395419, EPI_ISL_1395420, EPI_ISL_1395421, EPI_ISL_1395422, EPI_ISL_1395433, EPI_ISL_1395434, EPI_ISL_1395435, EPI_ISL_1395436, EPI_ISL_1395437, EPI_ISL_1395438, EPI_ISL_1395439, EPI_ISL_1395440, EPI_ISL_1395447, EPI_ISL_1395448, EPI_ISL_1395449, EPI_ISL_1395450, EPI_ISL_1395451, EPI_ISL_1395460, EPI_ISL_1395461, EPI_ISL_1395462, EPI_ISL_1395464, EPI_ISL_1395465, EPI_ISL_1395473, EPI_ISL_1395474, EPI_ISL_1395475, EPI_ISL_1395476, EPI_ISL_1395477, EPI_ISL_1395478, EPI_ISL_1395479, EPI_ISL_1395480, EPI_ISL_1395481, EPI_ISL_1395482, EPI_ISL_1395483, EPI_ISL_1395491, EPI_ISL_1395492, EPI_ISL_1395493, EPI_ISL_1395494, EPI_ISL_1395495, EPI_ISL_1395496, EPI_ISL_1395504, EPI_ISL_1395505, EPI_ISL_1395506, EPI_ISL_1395507, EPI_ISL_1395508, EPI_ISL_1395509, EPI_ISL_1395510, EPI_ISL_1395511, EPI_ISL_1395512, EPI_ISL_1395522, EPI_ISL_1395523, EPI_ISL_1395524, EPI_ISL_1395525, EPI_ISL_1395526, EPI_ISL_1395527, EPI_ISL_1395528, EPI_ISL_1395529, EPI_ISL_1395533, EPI_ISL_1395534, EPI_ISL_1395535, EPI_ISL_1395536, EPI_ISL_1395537, EPI_ISL_1395538, EPI_ISL_1395539, EPI_ISL_1395540, EPI_ISL_1395541, EPI_ISL_1395542, EPI_ISL_1395544, EPI_ISL_1395545, EPI_ISL_1395546, EPI_ISL_1395547, EPI_ISL_1395548, EPI_ISL_1395549, EPI_ISL_1395550, EPI_ISL_1395551, EPI_ISL_1395552, EPI_ISL_1395553, EPI_ISL_1395554, EPI_ISL_1395555, EPI_ISL_1395559, EPI_ISL_1395560, EPI_ISL_1395561, EPI_ISL_1395562, EPI_ISL_1395563, EPI_ISL_1395565, EPI_ISL_1395566, EPI_ISL_1395570, EPI_ISL_1395571, EPI_ISL_1395572, EPI_ISL_1395573, EPI_ISL_1395574, EPI_ISL_1395575, EPI_ISL_1395576, EPI_ISL_1395577, EPI_ISL_1395578, EPI_ISL_1395579, EPI_ISL_1395580, EPI_ISL_1395583, EPI_ISL_1395584, EPI_ISL_1395585, EPI_ISL_1395586, EPI_ISL_1395587, EPI_ISL_1395588, EPI_ISL_1395589, EPI_ISL_1395590, EPI_ISL_1395591, EPI_ISL_1395592, EPI_ISL_1395593, EPI_ISL_1395594, EPI_ISL_1395595, EPI_ISL_1395596, EPI_ISL_1395597, EPI_ISL_1395605, EPI_ISL_1395606, EPI_ISL_1395607, EPI_ISL_1395608, EPI_ISL_1395609, EPI_ISL_1395610, EPI_ISL_1395611, EPI_ISL_1395612, EPI_ISL_1395613, EPI_ISL_1395614, EPI_ISL_1395615, EPI_ISL_1395621, EPI_ISL_1395622, EPI_ISL_1395623, EPI_ISL_1395624, EPI_ISL_1395625, EPI_ISL_1395633, EPI_ISL_1395634, EPI_ISL_1395635, EPI_ISL_1395636, EPI_ISL_1395639, EPI_ISL_1395640, EPI_ISL_1395641, EPI_ISL_1395642, EPI_ISL_1395643, EPI_ISL_1395644, EPI_ISL_1395645, EPI_ISL_1395646, EPI_ISL_1395647, EPI_ISL_1395652, EPI_ISL_1395653, EPI_ISL_1395654, EPI_ISL_1395655, EPI_ISL_1395656, EPI_ISL_1395657, EPI_ISL_1395658, EPI_ISL_1395659, EPI_ISL_1395660, EPI_ISL_1395664, EPI_ISL_1395665, EPI_ISL_1395666, EPI_ISL_1395667, EPI_ISL_1395678, EPI_ISL_1395679, EPI_ISL_1395680, EPI_ISL_1395681, EPI_ISL_1395682, EPI_ISL_1395683, EPI_ISL_1395684, EPI_ISL_1395685, EPI_ISL_1395686, EPI_ISL_1395687, EPI_ISL_1395688, EPI_ISL_1395689, EPI_ISL_1395690, EPI_ISL_1395691, EPI_ISL_1395692, EPI_ISL_1395694, EPI_ISL_1395695, EPI_ISL_1395696, EPI_ISL_1395697, EPI_ISL_1395698, EPI_ISL_1395699, EPI_ISL_1395701, EPI_ISL_1395702, EPI_ISL_1395703, EPI_ISL_1395704, EPI_ISL_1395705, EPI_ISL_2449017, EPI_ISL_2449018, EPI_ISL_2449019, EPI_ISL_2449020, EPI_ISL_2449021, EPI_ISL_2449022, EPI_ISL_2449023, EPI_ISL_2449024, EPI_ISL_2449025, EPI_ISL_2449026, EPI_ISL_2449034, EPI_ISL_2449035, EPI_ISL_2449036, EPI_ISL_2449037, EPI_ISL_2449043, EPI_ISL_2449044, EPI_ISL_2449045, EPI_ISL_2449046, EPI_ISL_2449047, EPI_ISL_2449048, EPI_ISL_2449055, EPI_ISL_2449056, EPI_ISL_2449057, EPI_ISL_2449058, EPI_ISL_2449066, EPI_ISL_2449067, EPI_ISL_2449068, EPI_ISL_2449069, EPI_ISL_2449070, EPI_ISL_2449071, EPI_ISL_2449072, EPI_ISL_2449080, EPI_ISL_2449081, EPI_ISL_2449082, EPI_ISL_2449083, EPI_ISL_2449084, EPI_ISL_2449085, EPI_ISL_2449086, EPI_ISL_2449087, EPI_ISL_2449088, EPI_ISL_2449089, EPI_ISL_2449090, EPI_ISL_2449094, EPI_ISL_2449095, EPI_ISL_2449096, EPI_ISL_2449097, EPI_ISL_2449098, EPI_ISL_2449099, EPI_ISL_2449100, EPI_ISL_2449101, EPI_ISL_2449103, EPI_ISL_2449104, EPI_ISL_2449105, EPI_ISL_2449106, EPI_ISL_2449107, EPI_ISL_2449110, EPI_ISL_2449111, EPI_ISL_2449112, EPI_ISL_2449115, EPI_ISL_2449116, EPI_ISL_2449117, EPI_ISL_2449118, EPI_ISL_2449120, EPI_ISL_2449121, EPI_ISL_2449126, EPI_ISL_2449127, EPI_ISL_2449128, EPI_ISL_2449133, EPI_ISL_2449134, EPI_ISL_2449135, EPI_ISL_2449136, EPI_ISL_2449137, EPI_ISL_2449138, EPI_ISL_2449144, EPI_ISL_2449145, EPI_ISL_2449146, EPI_ISL_2449152, EPI_ISL_2449153, EPI_ISL_2449154, EPI_ISL_2449158, EPI_ISL_2449159, EPI_ISL_2449160, EPI_ISL_2449161, EPI_ISL_2449162, EPI_ISL_2449179, EPI_ISL_2449180, EPI_ISL_2449181, EPI_ISL_2449189, EPI_ISL_2449190, EPI_ISL_2449191, EPI_ISL_2449192, EPI_ISL_2449193, EPI_ISL_2449194, EPI_ISL_2449195, EPI_ISL_2449196, EPI_ISL_2449197, EPI_ISL_2449200, EPI_ISL_2449203, EPI_ISL_2449204, EPI_ISL_2449205, EPI_ISL_2449206, EPI_ISL_2449207, EPI_ISL_2449208, EPI_ISL_2449209, EPI_ISL_2449210, EPI_ISL_2449225, EPI_ISL_2449229, EPI_ISL_2449230, EPI_ISL_2449231, EPI_ISL_2449232, EPI_ISL_2449233, EPI_ISL_2449234, EPI_ISL_2449235, EPI_ISL_2449236, EPI_ISL_2449237, EPI_ISL_2449245, EPI_ISL_2449246, EPI_ISL_2449247, EPI_ISL_2449248, EPI_ISL_2449249, EPI_ISL_2449250, EPI_ISL_2449255, EPI_ISL_2449256, EPI_ISL_2449257, EPI_ISL_2449258, EPI_ISL_2449259, EPI_ISL_2449260, EPI_ISL_2449261, EPI_ISL_2449267, EPI_ISL_2449268, EPI_ISL_2449269, EPI_ISL_2449270, EPI_ISL_2449271, EPI_ISL_2449286, EPI_ISL_2449287, EPI_ISL_2449302, EPI_ISL_2449303, EPI_ISL_2449304, EPI_ISL_2449305, EPI_ISL_2449306, EPI_ISL_2449312, EPI_ISL_2449318, EPI_ISL_2449319, EPI_ISL_2449321, EPI_ISL_2449322, EPI_ISL_2449323, EPI_ISL_2449324, EPI_ISL_2449325, EPI_ISL_2449328, EPI_ISL_2449336, EPI_ISL_2449337, EPI_ISL_2449338, EPI_ISL_2449339, EPI_ISL_2449340, EPI_ISL_2449341, EPI_ISL_2449342, EPI_ISL_2449343, EPI_ISL_2449356, EPI_ISL_2449357, EPI_ISL_2449358, EPI_ISL_2449359, EPI_ISL_2449360, EPI_ISL_2449361, EPI_ISL_2449362, EPI_ISL_2449369, EPI_ISL_2449370, EPI_ISL_2449371, EPI_ISL_2449372, EPI_ISL_2449377, EPI_ISL_2449378, EPI_ISL_2449379, EPI_ISL_2449380, EPI_ISL_2449381, EPI_ISL_2449384, EPI_ISL_2449385, EPI_ISL_2449394, EPI_ISL_2449395, EPI_ISL_2449396, EPI_ISL_2449397, EPI_ISL_2449398, EPI_ISL_2449399, EPI_ISL_2449400, EPI_ISL_2449401, EPI_ISL_2449402, EPI_ISL_2449407, EPI_ISL_2449408, EPI_ISL_2449410, EPI_ISL_2449411, EPI_ISL_2449412, EPI_ISL_2449413, EPI_ISL_2449414, EPI_ISL_2449415, EPI_ISL_2449416, EPI_ISL_2449417, EPI_ISL_2449418, EPI_ISL_2449419, EPI_ISL_2449425, EPI_ISL_2449426, EPI_ISL_2449427, EPI_ISL_2449429, EPI_ISL_2449430, EPI_ISL_2449431, EPI_ISL_2449432, EPI_ISL_2449433, EPI_ISL_2449434, EPI_ISL_2449435, EPI_ISL_2449436, EPI_ISL_2449437, EPI_ISL_2449438, EPI_ISL_2449439, EPI_ISL_2449460, EPI_ISL_2449461, EPI_ISL_2449462, EPI_ISL_2449463, EPI_ISL_2449464, EPI_ISL_2449465, EPI_ISL_2449466, EPI_ISL_2449467, EPI_ISL_2449468, EPI_ISL_2449469, EPI_ISL_2449470, EPI_ISL_2449471, EPI_ISL_2449476, EPI_ISL_2449477, EPI_ISL_2449478, EPI_ISL_2449479, EPI_ISL_2449480, EPI_ISL_2449481, EPI_ISL_2449482, EPI_ISL_2449483, EPI_ISL_2449484, EPI_ISL_2449485, EPI_ISL_2449486, EPI_ISL_2449487, EPI_ISL_2449488, EPI_ISL_2449489, EPI_ISL_2449501, EPI_ISL_2449502, EPI_ISL_2449503, EPI_ISL_2449504, EPI_ISL_2449505, EPI_ISL_2449511, EPI_ISL_2449513, EPI_ISL_2449514, EPI_ISL_2449515, EPI_ISL_2449516, EPI_ISL_2449517, EPI_ISL_2449518, EPI_ISL_2449519, EPI_ISL_2449520, EPI_ISL_2449521, EPI_ISL_2449522, EPI_ISL_2449523, EPI_ISL_2449524, EPI_ISL_2449530, EPI_ISL_2449536, EPI_ISL_2449537, EPI_ISL_2449538, EPI_ISL_2449539, EPI_ISL_2449540, EPI_ISL_2449541, EPI_ISL_2449542, EPI_ISL_2449543, EPI_ISL_2449544, EPI_ISL_2449545, EPI_ISL_2449546, EPI_ISL_2449547, EPI_ISL_2449556, EPI_ISL_2449559, EPI_ISL_2449560, EPI_ISL_2449561, EPI_ISL_2449562, EPI_ISL_2449563, EPI_ISL_2449565, EPI_ISL_2449566, EPI_ISL_2449567, EPI_ISL_2449568, EPI_ISL_2449569, EPI_ISL_2449571, EPI_ISL_2449572, EPI_ISL_2449573, EPI_ISL_2449574, EPI_ISL_2449575, EPI_ISL_2449582, EPI_ISL_2449587, EPI_ISL_2449588, EPI_ISL_2449589, EPI_ISL_2449590, EPI_ISL_2449591, EPI_ISL_2449592, EPI_ISL_2449593, EPI_ISL_2449600, EPI_ISL_2449601, EPI_ISL_2449602, EPI_ISL_2449603, EPI_ISL_2449604, EPI_ISL_2449605, EPI_ISL_2449606, EPI_ISL_2449607, EPI_ISL_2449608, EPI_ISL_2449621, EPI_ISL_2449622, EPI_ISL_2449623, EPI_ISL_2449624, EPI_ISL_2449625, EPI_ISL_2449626, EPI_ISL_2449627, EPI_ISL_2449628, EPI_ISL_2449629, EPI_ISL_2449630, EPI_ISL_2449645, EPI_ISL_2449646, EPI_ISL_2449647, EPI_ISL_2449648, EPI_ISL_2449649, EPI_ISL_2449650, EPI_ISL_2449651, EPI_ISL_2449652, EPI_ISL_2449653, EPI_ISL_2449654, EPI_ISL_2449655, EPI_ISL_2449656, EPI_ISL_2449657, EPI_ISL_2449658, EPI_ISL_2449659, EPI_ISL_2449660, EPI_ISL_2449661, EPI_ISL_2449662, EPI_ISL_2449669, EPI_ISL_2449675, EPI_ISL_2449678, EPI_ISL_2449679, EPI_ISL_2449680, EPI_ISL_2449681, EPI_ISL_2449682, EPI_ISL_2449683, EPI_ISL_2449684, EPI_ISL_2449685, EPI_ISL_2449686, EPI_ISL_2449687, EPI_ISL_2449688, EPI_ISL_2449694, EPI_ISL_2449699, EPI_ISL_2449700, EPI_ISL_2449701, EPI_ISL_2449702, EPI_ISL_2449703, EPI_ISL_2449704, EPI_ISL_2449705, EPI_ISL_2449706, EPI_ISL_2449707, EPI_ISL_2449717, EPI_ISL_2449718, EPI_ISL_2449719, EPI_ISL_2449720, EPI_ISL_2449721, EPI_ISL_2449722, EPI_ISL_2449723, EPI_ISL_2449724, EPI_ISL_2449725, EPI_ISL_2449726, EPI_ISL_2449727, EPI_ISL_2449728, EPI_ISL_2449729, EPI_ISL_2449730, EPI_ISL_2449731, EPI_ISL_2449743, EPI_ISL_2449744, EPI_ISL_2449745, EPI_ISL_2449746, EPI_ISL_2449747, EPI_ISL_2449748, EPI_ISL_2449749, EPI_ISL_2449750, EPI_ISL_2449751, EPI_ISL_2449752, EPI_ISL_2449753, EPI_ISL_2449754, EPI_ISL_2449755, EPI_ISL_2449756, EPI_ISL_2449757, EPI_ISL_2449767, EPI_ISL_2449768, EPI_ISL_2449769, EPI_ISL_2449770, EPI_ISL_2449771, EPI_ISL_2449772, EPI_ISL_2449773, EPI_ISL_2449774, EPI_ISL_2449775, EPI_ISL_2449776, EPI_ISL_2449777, EPI_ISL_2449786, EPI_ISL_2449787, EPI_ISL_2449788, EPI_ISL_2449789, EPI_ISL_2449790, EPI_ISL_2449791, EPI_ISL_2449792, EPI_ISL_2449793, EPI_ISL_2449794, EPI_ISL_2449804, EPI_ISL_2449805, EPI_ISL_2449806, EPI_ISL_2449807, EPI_ISL_2449808, EPI_ISL_2449809, EPI_ISL_2449810, EPI_ISL_2449811, EPI_ISL_2449812, EPI_ISL_2449813, EPI_ISL_2449814, EPI_ISL_2449822, EPI_ISL_2449829, EPI_ISL_2449830, EPI_ISL_2449835, EPI_ISL_2449837, EPI_ISL_2454372, EPI_ISL_2454373, EPI_ISL_2454374, EPI_ISL_2454375, EPI_ISL_2454376, EPI_ISL_2454377, EPI_ISL_2454379, EPI_ISL_3277884, EPI_ISL_3277890, EPI_ISL_3277891, EPI_ISL_3277892, EPI_ISL_3277905, EPI_ISL_3277906, EPI_ISL_3277907, EPI_ISL_3277908, EPI_ISL_3277909, EPI_ISL_3277910, EPI_ISL_3277911, EPI_ISL_3277912, EPI_ISL_3277913, EPI_ISL_3277914, EPI_ISL_3277915, EPI_ISL_3277916, EPI_ISL_3277917, EPI_ISL_3277918, EPI_ISL_3277919, EPI_ISL_3277920, EPI_ISL_3277921, EPI_ISL_3277922, EPI_ISL_3277923, EPI_ISL_3277924, EPI_ISL_3277925, EPI_ISL_3277926, EPI_ISL_3277927, EPI_ISL_3277928, EPI_ISL_3277929, EPI_ISL_3277930, EPI_ISL_3277931, EPI_ISL_3277932, EPI_ISL_3277933, EPI_ISL_3277934, EPI_ISL_3277935, EPI_ISL_3277936, EPI_ISL_3277937, EPI_ISL_3277938, EPI_ISL_3277939, EPI_ISL_3277940, EPI_ISL_3277941, EPI_ISL_3277942, EPI_ISL_3277943, EPI_ISL_3277991, EPI_ISL_3277992, EPI_ISL_3277993, EPI_ISL_3277994, EPI_ISL_3277995, EPI_ISL_3277996, EPI_ISL_3277997, EPI_ISL_3277998, EPI_ISL_3277999, EPI_ISL_3278000, EPI_ISL_3278001, EPI_ISL_3278002, EPI_ISL_3278003, EPI_ISL_3278004, EPI_ISL_3278005, EPI_ISL_3278006, EPI_ISL_3278007, EPI_ISL_3278008, EPI_ISL_3278009, EPI_ISL_3278010, EPI_ISL_3278011, EPI_ISL_3278012, EPI_ISL_3278013, EPI_ISL_3278014, EPI_ISL_3278015, EPI_ISL_3278016, EPI_ISL_3278017, EPI_ISL_3278018, EPI_ISL_3278019, EPI_ISL_3278020, EPI_ISL_3278021, EPI_ISL_3278022, EPI_ISL_3278023, EPI_ISL_3278024, EPI_ISL_3278025, EPI_ISL_3278026, EPI_ISL_3278027, EPI_ISL_3278028, EPI_ISL_3278029, EPI_ISL_3278030, EPI_ISL_3278031, EPI_ISL_3278184, EPI_ISL_3278185, EPI_ISL_3278186, EPI_ISL_3278187, EPI_ISL_3278188, EPI_ISL_3278189, EPI_ISL_3278190, EPI_ISL_3278191, EPI_ISL_3278192, EPI_ISL_3278193, EPI_ISL_3278194, EPI_ISL_3278195, EPI_ISL_3278196, EPI_ISL_3278197, EPI_ISL_3278198, EPI_ISL_3278199, EPI_ISL_3278200, EPI_ISL_3278201, EPI_ISL_3278202, EPI_ISL_3278203, EPI_ISL_3278204, EPI_ISL_3278205, EPI_ISL_3278234, EPI_ISL_3278235, EPI_ISL_3278236, EPI_ISL_3278237, EPI_ISL_3278238, EPI_ISL_3278239, EPI_ISL_3278240, EPI_ISL_3278241, EPI_ISL_3278242, EPI_ISL_3278283, EPI_ISL_5656652, EPI_ISL_5656653, EPI_ISL_5656654, EPI_ISL_5656655, EPI_ISL_5656656, EPI_ISL_5656657, EPI_ISL_5656658, EPI_ISL_5656659, EPI_ISL_5656660, EPI_ISL_5656661, EPI_ISL_5656662, EPI_ISL_5656663, EPI_ISL_5656664, EPI_ISL_5656665, EPI_ISL_5656666, EPI_ISL_5656691, EPI_ISL_5656692, EPI_ISL_5656693, EPI_ISL_5656694, EPI_ISL_5656695, EPI_ISL_5656696, EPI_ISL_5656697, EPI_ISL_5656698, EPI_ISL_5656699, EPI_ISL_5656700, EPI_ISL_5656701, EPI_ISL_5656702, EPI_ISL_5656703, EPI_ISL_5656704, EPI_ISL_5656705, EPI_ISL_5656706, EPI_ISL_5656707, EPI_ISL_5656708, EPI_ISL_5656756, EPI_ISL_5656757, EPI_ISL_5656758, EPI_ISL_5656759, EPI_ISL_5656786, EPI_ISL_5656787, EPI_ISL_5656788, EPI_ISL_5656789, EPI_ISL_5656790, EPI_ISL_5656793, EPI_ISL_5656794, EPI_ISL_5656795, EPI_ISL_5656796, EPI_ISL_5656797, EPI_ISL_5656798, EPI_ISL_5656799, EPI_ISL_5656800, EPI_ISL_5656801, EPI_ISL_5656802, EPI_ISL_5656819, EPI_ISL_5656820, EPI_ISL_5656821, EPI_ISL_5656822, EPI_ISL_5656823, EPI_ISL_5656824, EPI_ISL_5656825, EPI_ISL_5656826, EPI_ISL_5656827, EPI_ISL_5656828, EPI_ISL_5656829, EPI_ISL_5656830, EPI_ISL_5656831, EPI_ISL_5656832, EPI_ISL_5656833, EPI_ISL_5656834, EPI_ISL_5656835, EPI_ISL_5656836, EPI_ISL_5656837, EPI_ISL_5656838, EPI_ISL_5656839, EPI_ISL_5656840, EPI_ISL_5656841, EPI_ISL_5656842, EPI_ISL_5656843, EPI_ISL_5656844, EPI_ISL_5656845, EPI_ISL_5656846, EPI_ISL_5656847, EPI_ISL_5656848, EPI_ISL_5656849, EPI_ISL_5656850, EPI_ISL_5656851, EPI_ISL_5656852, EPI_ISL_5656853, EPI_ISL_5656854, EPI_ISL_5656855, EPI_ISL_5656856, EPI_ISL_5656857, EPI_ISL_5656858, EPI_ISL_5656859, EPI_ISL_5656860, EPI_ISL_5656874, EPI_ISL_5656875, EPI_ISL_5656930, EPI_ISL_5656931, EPI_ISL_5656932, EPI_ISL_5656933, EPI_ISL_5656934, EPI_ISL_5656935, EPI_ISL_5656936, EPI_ISL_5656937, EPI_ISL_5656938, EPI_ISL_5656939, EPI_ISL_5656940, EPI_ISL_5656941, EPI_ISL_5656942, EPI_ISL_5656943, EPI_ISL_5656944, EPI_ISL_5656945, EPI_ISL_5656946, EPI_ISL_5656947, EPI_ISL_5656948, EPI_ISL_5656949, EPI_ISL_5656950, EPI_ISL_5656951, EPI_ISL_5656952, EPI_ISL_5656953, EPI_ISL_5656954, EPI_ISL_5656955, EPI_ISL_5656956, EPI_ISL_5656957, EPI_ISL_5656958, EPI_ISL_5656959, EPI_ISL_5656960, EPI_ISL_5656961, EPI_ISL_5656962, EPI_ISL_5656963, EPI_ISL_5656964, EPI_ISL_5656965, EPI_ISL_5656966, EPI_ISL_5656967, EPI_ISL_5656968, EPI_ISL_5656969, EPI_ISL_5656970, EPI_ISL_5656971, EPI_ISL_5656972, EPI_ISL_5657011, EPI_ISL_5657014, EPI_ISL_5657015, EPI_ISL_5657016, EPI_ISL_5657017, EPI_ISL_5657018, EPI_ISL_5657019, EPI_ISL_5657020, EPI_ISL_5657021, EPI_ISL_5657022, EPI_ISL_5657023, EPI_ISL_5657064, EPI_ISL_5657065, EPI_ISL_5657066, EPI_ISL_5657067, EPI_ISL_5657118, EPI_ISL_5657173, EPI_ISL_5657174, EPI_ISL_5657175, EPI_ISL_5657176, EPI_ISL_5657177, EPI_ISL_5657178, EPI_ISL_5657179, EPI_ISL_5657180, EPI_ISL_5657181, EPI_ISL_5657182, EPI_ISL_5657183, EPI_ISL_5657184, EPI_ISL_5657185, EPI_ISL_5657186, EPI_ISL_5657187, EPI_ISL_5657188, EPI_ISL_5657189, EPI_ISL_5657191, EPI_ISL_5657192, EPI_ISL_5657296, EPI_ISL_5657300, EPI_ISL_5657349, EPI_ISL_5657466, EPI_ISL_5657548, EPI_ISL_5657556			
see above	Laboratorio de Virología del Hospital de Niños Dr. Ricardo Gutierrez	Área de Secuenciación del Laboratorio de Virología del Hospital de Niños Dr. Ricardo Gutierrez on behalf of 'Proyecto Argentino Interinstitucional de genomica de SARS-CoV-2' (PAIS Consortium)	A; Acevedo; Acuña; Alexay; Alvarez Lopez; Barreda Frank; C; D; E; G; Goya; Grandis; Jacques; LE; Labarta; Lusso; M; ME; MI; Medina; Mistchenko; N; Nabaes Jodar; Natale; O; S; Streitenberger; Thomas; Valinotto; Viegas, M.; Villegas

EPI_ISL_2449315, EPI_ISL_2449326, EPI_ISL_2449346, EPI_ISL_2449366, EPI_ISL_2449382, EPI_ISL_2449420, EPI_ISL_2449421, EPI_ISL_2449459, EPI_ISL_2449475, EPI_ISL_2449499, EPI_ISL_2449510, EPI_ISL_2449555, EPI_ISL_5657042, EPI_ISL_5657043, EPI_ISL_5657044, EPI_ISL_5657045, EPI_ISL_5657046, EPI_ISL_5657047, EPI_ISL_5657048, EPI_ISL_5657049, EPI_ISL_5657050, EPI_ISL_5657051, EPI_ISL_5657052, EPI_ISL_5657054, EPI_ISL_5657055, EPI_ISL_5657056, EPI_ISL_5657057, EPI_ISL_5657058			
see above	Laboratorio de biología molecular del Hospital Centenario	Área de Secuenciación del Laboratorio de Virología del Hospital de Niños Dr. Ricardo Gutierrez on behalf of 'Proyecto Argentino Interinstitucional de genomica de SARS-CoV-2' (PAIS Consortium)	Acuña; Alexay; Bourlot; D; Débora Kesselman; G; Goya; Gustavo Levin; I; Ignacio Bourlot; Kesselman; L; LE; Leticia Siri; Levin; Lusso; M; MI; Melchiori; Nabaes Jodar; Natale; P; Paulina Melchiori; S; Siri; Valinotto; Viegas; Viegas, M.
EPI_ISL_3278097, EPI_ISL_3278098, EPI_ISL_3278099, EPI_ISL_3278100, EPI_ISL_3278101, EPI_ISL_3278102, EPI_ISL_3278103, EPI_ISL_3278104, EPI_ISL_3278105, EPI_ISL_3278106, EPI_ISL_3278107, EPI_ISL_3278108, EPI_ISL_3278109, EPI_ISL_3278110, EPI_ISL_3278111, EPI_ISL_3278112, EPI_ISL_3278113, EPI_ISL_3278114, EPI_ISL_3278115, EPI_ISL_3278116, EPI_ISL_3278117, EPI_ISL_3278118, EPI_ISL_3278119, EPI_ISL_3278120, EPI_ISL_3278121, EPI_ISL_3278122, EPI_ISL_3278123, EPI_ISL_3278124, EPI_ISL_3278125, EPI_ISL_3278126, EPI_ISL_3278127, EPI_ISL_3278128, EPI_ISL_3278129, EPI_ISL_3278130, EPI_ISL_3278131, EPI_ISL_3278132, EPI_ISL_3278133			
see above	Laboratorio de la Dirección de Epidemiología	Área de Secuenciación del Laboratorio de Virología del Hospital de Niños Dr. Ricardo Gutierrez on behalf of 'Proyecto Argentino Interinstitucional de genomica de SARS-CoV-2' (PAIS Consortium)	Acuña; Alexay; Claudia Rechimont; D; Goya; José Carlos Usero; LE; Lusso; M; MI; María de los Ángeles Irastorza; Matías Villasana; Nabaes Jodar; Natale; S; Valinotto; Viegas
EPI_ISL_1394922, EPI_ISL_1394923, EPI_ISL_1394924, EPI_ISL_1395140, EPI_ISL_1395141, EPI_ISL_1395142, EPI_ISL_1395143, EPI_ISL_1395144, EPI_ISL_1395160, EPI_ISL_1395161, EPI_ISL_1395162, EPI_ISL_1395163, EPI_ISL_1395164, EPI_ISL_1395165, EPI_ISL_1395166, EPI_ISL_2449212, EPI_ISL_2449213, EPI_ISL_2449214, EPI_ISL_2449215, EPI_ISL_2449216, EPI_ISL_2449217, EPI_ISL_2449218, EPI_ISL_2449219, EPI_ISL_2449220, EPI_ISL_2449221, EPI_ISL_2449222, EPI_ISL_2449223, EPI_ISL_2449224, EPI_ISL_2449239, EPI_ISL_2449240, EPI_ISL_2449241, EPI_ISL_2449242, EPI_ISL_2449243, EPI_ISL_2449244, EPI_ISL_2449275, EPI_ISL_2449276, EPI_ISL_2449277, EPI_ISL_2449288, EPI_ISL_2449307, EPI_ISL_2449308, EPI_ISL_2449309			
see above	Laboratorio de salud pública, Facultad de Ciencias Exactas, UNLP	Área de Secuenciación del Laboratorio de Virología del Hospital de Niños Dr. Ricardo Gutierrez on behalf of 'Proyecto Argentino Interinstitucional de genomica de SARS-CoV-2' (PAIS Consortium)	Acuña; Alexay; Andrés Angelletti; Andrés Cordero; Carina Tersigni; D; Goya; LE; Laura Delaplace; Lusso; M; MI; Nabaes Jodar; Natale; Rosana Isabel Toro; S; Valinotto; Victoria Nadalich; Viegas, M.; Víctoria Cabassi
EPI_ISL_1394909, EPI_ISL_1394948, EPI_ISL_1394972, EPI_ISL_1394985, EPI_ISL_1394998, EPI_ISL_1394999, EPI_ISL_1395000, EPI_ISL_1395017, EPI_ISL_1395018, EPI_ISL_1395019, EPI_ISL_1395020, EPI_ISL_1395036, EPI_ISL_1395037, EPI_ISL_1395038, EPI_ISL_1395039, EPI_ISL_1395040, EPI_ISL_1395052, EPI_ISL_1395053, EPI_ISL_1395054, EPI_ISL_1395067, EPI_ISL_1395068, EPI_ISL_1395069, EPI_ISL_1395072, EPI_ISL_1395194, EPI_ISL_1395195, EPI_ISL_1395196, EPI_ISL_1395197, EPI_ISL_1395214, EPI_ISL_1395215, EPI_ISL_1395216, EPI_ISL_1395217, EPI_ISL_1395356, EPI_ISL_1395357, EPI_ISL_1395358, EPI_ISL_1395359, EPI_ISL_1395360, EPI_ISL_1395361, EPI_ISL_1395366, EPI_ISL_1395367, EPI_ISL_1395368, EPI_ISL_1395378, EPI_ISL_1395379, EPI_ISL_1395380, EPI_ISL_1395381, EPI_ISL_1395385, EPI_ISL_1395386, EPI_ISL_1395387, EPI_ISL_1395388, EPI_ISL_1395389, EPI_ISL_1395394, EPI_ISL_1395395, EPI_ISL_1395396, EPI_ISL_1395397, EPI_ISL_1395405, EPI_ISL_1395406, EPI_ISL_1395407, EPI_ISL_1395413, EPI_ISL_1395414, EPI_ISL_1395415, EPI_ISL_1395416, EPI_ISL_1395417, EPI_ISL_1395418, EPI_ISL_1395424, EPI_ISL_1395425, EPI_ISL_1395426, EPI_ISL_1395427, EPI_ISL_1395428, EPI_ISL_1395429, EPI_ISL_1395430, EPI_ISL_1395431, EPI_ISL_1395432, EPI_ISL_1395441, EPI_ISL_1395442, EPI_ISL_1395443, EPI_ISL_1395444, EPI_ISL_1395445, EPI_ISL_1395446, EPI_ISL_1395454, EPI_ISL_1395455, EPI_ISL_1395456, EPI_ISL_1395457, EPI_ISL_1395458, EPI_ISL_1395459, EPI_ISL_1395467, EPI_ISL_1395468, EPI_ISL_1395469, EPI_ISL_1395470, EPI_ISL_1395471, EPI_ISL_1395472, EPI_ISL_1395484, EPI_ISL_1395485, EPI_ISL_1395486, EPI_ISL_1395487, EPI_ISL_1395488, EPI_ISL_1395489, EPI_ISL_1395490, EPI_ISL_1395498, EPI_ISL_1395499, EPI_ISL_1395500, EPI_ISL_1395501, EPI_ISL_1395502, EPI_ISL_1395503, EPI_ISL_1395516, EPI_ISL_1395517, EPI_ISL_1395518, EPI_ISL_1395519, EPI_ISL_1395520, EPI_ISL_1395521, EPI_ISL_1395602, EPI_ISL_1395603, EPI_ISL_1395604, EPI_ISL_1395616, EPI_ISL_1395617, EPI_ISL_1395618, EPI_ISL_1395619, EPI_ISL_1395620, EPI_ISL_1395628, EPI_ISL_1395629, EPI_ISL_1395630, EPI_ISL_1395631, EPI_ISL_1395632, EPI_ISL_1395638, EPI_ISL_1395649, EPI_ISL_1395650, EPI_ISL_1395651, EPI_ISL_1395661, EPI_ISL_1395662, EPI_ISL_1395663, EPI_ISL_1395672, EPI_ISL_1395673, EPI_ISL_1395674, EPI_ISL_1395675, EPI_ISL_1395676, EPI_ISL_1395677, EPI_ISL_2449148, EPI_ISL_2449149, EPI_ISL_2449150, EPI_ISL_2449157, EPI_ISL_2449163, EPI_ISL_2449226, EPI_ISL_2449227, EPI_ISL_2449228, EPI_ISL_2449254, EPI_ISL_2449265, EPI_ISL_2449272, EPI_ISL_2449273, EPI_ISL_2449300, EPI_ISL_2449301, EPI_ISL_2449313, EPI_ISL_2449314, EPI_ISL_2449320, EPI_ISL_2449335, EPI_ISL_2449354, EPI_ISL_2449355, EPI_ISL_2449834, EPI_ISL_2449842, EPI_ISL_2449843, EPI_ISL_3277973, EPI_ISL_3277974, EPI_ISL_3277975, EPI_ISL_3277976, EPI_ISL_3277977, EPI_ISL_3277978, EPI_ISL_3277979, EPI_ISL_3277980, EPI_ISL_3277981, EPI_ISL_3277982, EPI_ISL_3277983, EPI_ISL_3277984, EPI_ISL_3277985, EPI_ISL_3277986, EPI_ISL_3277987, EPI_ISL_3277988, EPI_ISL_3277989, EPI_ISL_3277990			
see above	Laboratorio del Hospital Interzonal General de Agudos "Evita"	Área de Secuenciación del Laboratorio de Virología del Hospital de Niños Dr. Ricardo Gutierrez on behalf of 'Proyecto Argentino Interinstitucional de genomica de SARS-CoV-2' (PAIS Consortium)	Acuña; Alejandra Musto; Alexay; D; Erica Luczak; Goya; Isabel Desimone; LE; Lorena Serrano; Lusso; M; MI; Nabaes Jodar; Natale; Omar Grossi; S; Valinotto; Viegas, M.
EPI_ISL_5657053, EPI_ISL_5657059, EPI_ISL_5657767, EPI_ISL_5657774, EPI_ISL_5657986, EPI_ISL_5657993	Laboratorio del Hospital Interzonal General de Agudos “Eva Perón”	Área de Secuenciación del Laboratorio de Virología del Hospital de Niños Dr. Ricardo Gutierrez on behalf of 'Proyecto Argentino Interinstitucional de genomica de SARS-CoV-2' (PAIS Consortium)	Acuña; Alexay; Carolina Piñeyro; Cecilia Corigliano; Claudia Kairiyama; Claudia Pengue; D; Goya; Graciela Radczuk; LE; Lusso; M; MI; Mariana Carulla; Nabaes Jodar; Natale; S; Santiago Rampulla; Valinotto; Viegas
EPI_ISL_5656913, EPI_ISL_5656914, EPI_ISL_5656915, EPI_ISL_5657073, EPI_ISL_5657074, EPI_ISL_5657075, EPI_ISL_5657076, EPI_ISL_5657077			
see above	Laboratorio del Hospital Interzonal General de Agudos “Evita”	Área de Secuenciación del Laboratorio de Virología del Hospital de Niños Dr. Ricardo Gutierrez on behalf of 'Proyecto Argentino Interinstitucional de genomica de SARS-CoV-2' (PAIS Consortium)	Acuña; Alejandra Musto; Alexay; D; Erica Luczak; Goya; Isabel Desimone; LE; Lorena Serrano; Lusso; M; MI; Nabaes Jodar; Natale; Omar Grossi; Rubén Pelagamos; S; Valinotto; Viegas
EPI_ISL_1394916, EPI_ISL_1395392, EPI_ISL_1395564	Laboratorio del Hospital Teresa Germani Laferrere	Área de Secuenciación del Laboratorio de Virología del Hospital de Niños Dr. Ricardo Gutierrez on behalf of 'Proyecto Argentino Interinstitucional de genomica de SARS-CoV-2' (PAIS Consortium)	Acuña; Alexay; Anabel Luzio; D; Gisela Sauco; Goya; LE; Lusso; M; MI; Mauricio Spacaventto; Nabaes Jodar; Natale; S; Valinotto; Viegas, M.
EPI_ISL_5656807, EPI_ISL_5656808, EPI_ISL_5656809, EPI_ISL_5656810, EPI_ISL_5656811, EPI_ISL_5656812, EPI_ISL_5656813, EPI_ISL_5657777, EPI_ISL_5657782, EPI_ISL_5657783			
see above	Laboratorio del Hospital Zonal Especializado Materno Infantil “Argentina Diego”	Área de Secuenciación del Laboratorio de Virología del Hospital de Niños Dr. Ricardo Gutierrez on behalf of 'Proyecto Argentino Interinstitucional de genomica de SARS-CoV-2' (PAIS Consortium)	Acuña; Alexay; D; Farinella Bagnozzi y Santiago Hernán Gauna; Goya; Guillermina; LE; Lusso; M; MI; María Belén Arpaia; Nabaes Jodar; Natale; S; Valinotto; Viegas
EPI_ISL_1394910, EPI_ISL_1394914, EPI_ISL_1394917, EPI_ISL_1394918, EPI_ISL_1394919, EPI_ISL_1394920, EPI_ISL_1394921, EPI_ISL_1394936, EPI_ISL_1394937, EPI_ISL_1394938, EPI_ISL_1394939, EPI_ISL_1394940, EPI_ISL_1394945, EPI_ISL_1394946, EPI_ISL_1394949, EPI_ISL_1395041, EPI_ISL_2449797, EPI_ISL_2449798, EPI_ISL_2449818, EPI_ISL_2449819, EPI_ISL_2449826, EPI_ISL_2449838, EPI_ISL_2449839, EPI_ISL_2449840, EPI_ISL_2449845, EPI_ISL_2449846, EPI_ISL_2449850, EPI_ISL_2449851, EPI_ISL_2449852, EPI_ISL_2449853, EPI_ISL_2449854, EPI_ISL_2449855, EPI_ISL_2449856, EPI_ISL_2449857, EPI_ISL_2449858			
see above	Plataforma de Servicios Biotecnólogicos; UTTIPP/PSB	Área de Secuenciación del Laboratorio de Virología del Hospital de Niños Dr. Ricardo Gutierrez on behalf of 'Proyecto Argentino Interinstitucional de genomica de SARS-CoV-2' (PAIS Consortium)	Acuña; Alejandra Zinni; Alejandro Castello; Alexay; Carla Capobianco; D; Georgina Cardama; Goya; Gustavo Bad; Hernán Farina; Humberto Lamdan; LE; Lusso; M; MI; Marcelo Mandile; Nabaes Jodar; Natale; Norailys Lorenzo; S; Sandra Goñi; Valinotto; Viegas, M.
EPI_ISL_2449500, EPI_ISL_2449557, EPI_ISL_2449558, EPI_ISL_2449676, EPI_ISL_2449677, EPI_ISL_2449695, EPI_ISL_2449696, EPI_ISL_2449697, EPI_ISL_2449698, EPI_ISL_2449713, EPI_ISL_2449714, EPI_ISL_2449715, EPI_ISL_2449716, EPI_ISL_2449742, EPI_ISL_2449821			
see above	Sección Biología Molecular, laboratorio Hospital Schestakow	Área de Secuenciación del Laboratorio de Virología del Hospital de Niños Dr. Ricardo Gutierrez on behalf of 'Proyecto Argentino Interinstitucional de genomica de SARS-CoV-2' (PAIS Consortium)	A; Acuña; Alexay; C; Capone; D; F; Goya; LE; Lucero; Lusso; M; MI; N; Nabaes Jodar; Natale; Oviedo; S; Valinotto; Viegas, M.
EPI_ISL_3759759	TRAILER ALMAFUERTE	Laboratorio Central Mg. Luis Alfredo Pianciola	C Pintos; C Rastellini; C Ziehm; J Ousset; L Pianciola; M Fernandez; M Mazzeo; M Viegas.
EPI_ISL_3759744, EPI_ISL_3759745	TRAILER CENTRO	Laboratorio Central Mg. Luis Alfredo Pianciola	C Pintos; C Rastellini; C Ziehm; J Ousset; L Pianciola; M Fernandez; M Mazzeo; M Viegas.
EPI_ISL_3759738	TRAILER RUCA CHE	Laboratorio Central Mg. Luis Alfredo Pianciola	C Pintos; C Rastellini; C Ziehm; J Ousset; L Pianciola; M Fernandez; M Mazzeo; M Viegas.
EPI_ISL_2348738	Trailer Operativo Detectar	Laboratorio Central Mg. Luis Alfredo Pianciola on behalf of 'Proyecto Argentino Interinstitucional de genomica de SARS-CoV-2' (PAIS Consortium)	C Pintos; C Rastellini; C Ziehm; J Ousset; L Pianciola; M Fernandez; M Mazzeo
EPI_ISL_3568635, EPI_ISL_3568636	UCE DUAM	Laboratorio Central Mg. Luis Alfredo Pianciola	C Pintos; C Rastellini; C Ziehm; J Ousset; L Pianciola; M Fernandez; M Mazzeo; M Viegas.
EPI_ISL_5657190	UFU Hospital Rivadavia	Área de Secuenciación del Laboratorio de Virología del Hospital de Niños Dr. Ricardo Gutierrez on behalf of 'Proyecto Argentino Interinstitucional de genomica de SARS-CoV-2' (PAIS Consortium)	Acuña; Alexay; Alicia Acro; Andrea Lapeire; Claudia Brunetti; D; Daniela Naveira; Goya; Juan Manuel Peyran Ponce; LE; Larissa Cardoso; Lusso; M; MI; Marcela Balsarini; Marta Ledesma; María Inés Debas; Mercedes Borghi; Nabaes Jodar; Natale; Nelson; S; Silvia De La Torre; Solari; Valinotto; Viegas
EPI_ISL_3759736, EPI_ISL_3759737, EPI_ISL_3759757, EPI_ISL_3759761	Unidad de Cuidados Exclusivos DUAM	Laboratorio Central Mg. Luis Alfredo Pianciola	C Pintos; C Rastellini; C Ziehm; J Ousset; L Pianciola; M Fernandez; M Mazzeo; M Viegas.
EPI_ISL_2449367	Unidad de Virología, Centro de Educación Médica en Investigaciones Clínicas "CEMIC"	Área de Secuenciación del Laboratorio de Virología del Hospital de Niños Dr. Ricardo Gutierrez on behalf of 'Proyecto Argentino Interinstitucional de genomica de SARS-CoV-2' (PAIS Consortium)	Acuña; Alexay; D; Goya; LE; Lusso; M; MI; Marcela Echavarría; Nabaes Jodar; Natale; S; Valinotto; Viegas, M.

Table S4

We gratefully acknowledge the following Authors from the Originating laboratories responsible for obtaining the specimens, as well as the Submitting laboratories where the genome data were generated and shared via GISAID, on which this research is based.

All Submitters of data may be contacted directly via www.gisaid.org

Authors are sorted alphabetically.

Accession ID	Originating Laboratory	Submitting Laboratory	Authors
EPI_ISL_1013610	Department for Molecular Diagnostics, Centre for Medical Microbiology, Institute of Public Health, Montenegro	Charité Universitätsmedizin Berlin, Institut für Virologie	Victor M Corman, Barbara Mühlemann, Jörn Beheim-Schwarzbach, Julia Tesch, Tobias Bleicker, Danijela Vujoševi, Marija Govedarica, Talitha Veith, Julia Schneider, Terry Jones, Christian Drosten
EPI_ISL_1051869	Oxford Viromics, NDM, University of Oxford; Oxford University Hospitals; Basingstoke and North Hampshire Hospital	COVID-19 Genomics UK (COG-UK) Consortium	Tanya Golubchik, David Bonsall, George Macintyre, Amy Trebes, Mariateresa de Cesare, Catrin Moore, Alex Mobbs, Anita Justice, Robert Shaw, Monique Andersson, Timothy Peto, Emma Wise, Nathan Moore, Jessica Lynch, Nick Cortes, Matilde Mori, Stephen Kidd, David Buck, John Todd, Christophe Fraser
EPI_ISL_1088294	Quest Diagnostics Incorporated	Respiratory Viruses Branch, Division of Viral Diseases, Centers for Disease Control and Prevention	Peter W. Cook, Dakota Howard, Dhwani Batra, Ben L. Rambo-Martin, S. H. Rosenthal, A. Gerasimova, R. M. Kagan, B. Anderson, M. Hua, Y. Liu, L.E. Bernstein, K.E. Livingston, A. Perez, I. A. Shlyakhter, R. V. Rolando, R. Owen, P. Tanpaiboon, F. Lacbawan, Clinton R. Paden, Suxiang Tong, Duncan MacCannell
EPI_ISL_1092009	Kansas Health and Environmental Lab	Kansas Health and Environmental Lab	Mike Grose, Paige Drury, Carissa Robertson, Ben Olsen, and Phil Adam
EPI_ISL_1093172	Laboratorio de Referencia Nacional de Virus Respiratorio. Instituto Nacional de Salud Perú	Laboratorio de Referencia Nacional de Biotecnología y Biología Molecular. Instituto Nacional de Salud Perú	Carlos Padilla Rojas, Karolyn Vega Chozo, Luis Barcena, Priscila Lope Pari, Omar Caceres Rey, Marco Galarza Perez, Maribel Huaringa Nuñez, Johanna Balbuena Torrez, Henri Bailon Calderon, Nancy Rojas Serrano
EPI_ISL_1150267	Sonic - Labor Dr. von Froreich GmbH	Robert Koch Institute	unknown
EPI_ISL_1172872	Pandemic Response Lab - NYC	Pandemic Response Lab, R&D	Henry Lee, Michael Hammerling, Melissa Hopkins, Cybill del Castillo, Shinyoung Clair Kang, William Ward, Pradeep Bugga, Haiping Hao, Jon Laurent
EPI_ISL_1179460	Originating lab: Wales Specialist Virology Centre Sequencing lab: Pathogen Genomics Unit	Public Health Wales Microbiology Cardiff Wales Specialist Virology Centre	Catherine Moore, Johnathan Evans, Laura Gifford, Malorie Perry, Simon Cottrell, Angela Marchbank, Alec Birchley, Alexander Adams, Amy Gaskin, Bree Gatica-Wilcox, Jason Coombes, Joel Southgate, Lauren Gilbert, Lee Graham, Nicole Pacchiarini, Sara Kumziene-Summerhayes, Sarah Taylor, Sophie Jones, Sara Rey, Matthew Bull, Joanne Watkins, Sally Corden, Tom Connor
EPI_ISL_1196428	CLINICA BIBLICA	Inciensa, Instituto Costarricense de Investigación y Enseñanza en Nutrición y Salud	Francisco Duarte, Hebleen Porras, Claudio Soto-Garita, Estela Cordero, Adriana Godínez, Melany Calderón & Karla Gutiérrez-González
EPI_ISL_1240208	Jessa	Jessa	Cruys et al. on behalf of the Jessa_cmdLab
EPI_ISL_1249076	Oxford Viromics, NDM, University of Oxford; Oxford University Hospitals; Basingstoke and North Hampshire Hospital	COVID-19 Genomics UK (COG-UK) Consortium	Tanya Golubchik, David Bonsall, George Macintyre, Amy Trebes, Mariateresa de Cesare, Catrin Moore, Alex Mobbs, Anita Justice, Robert Shaw, Monique Andersson, Timothy Peto, Emma Wise, Nathan Moore, Jessica Lynch, Nick Cortes, Matilde Mori, Stephen Kidd, David Buck, John Todd, Christophe Fraser
EPI_ISL_1284639	Sonic - Labor Dr. von Froreich GmbH	Robert Koch Institute	unknown
EPI_ISL_1297282, EPI_ISL_1297306	BIOMNIS PARIS	CNR Virus des Infections Respiratoires - France SUD	Antonin Bal, Gregory Destras, Gwendolyne Burfin, Hadrien Regue, Quentin Semanas, Martine Valette, Bruno Lina, Laurence Josset
EPI_ISL_1313062	BIOMNIS LYON	CNR Virus des Infections Respiratoires - France SUD	Antonin Bal, Gregory Destras, Gwendolyne Burfin, Hadrien Regue, Quentin Semanas, Martine Valette, Bruno Lina, Laurence Josset
EPI_ISL_1322212	IrsiCaixa	IrsiCaixa	Marc Noguera-Julian, Mariona Parera, Maria Casadellà, Pilar Armengol, Francesc Catala-Moll, Roger Paredes, Bonaventura Clotet Gloria Trujillo, Rafel Perez Vidal, Jaume Trape Pujol, Carolina Gonzalez Fernandez, Roger Paredes, Eulalia Grau, Bonaventura Clotet
EPI_ISL_1337155	Helix/Illumina	Centers for Disease Control and Prevention Division of Viral Diseases, Pathogen Discovery	Peter W. Cook, Dakota Howard, Dhwani Batra, Ben L. Rambo-Martin, Eileen de Feo, Jan Antico, Christine Tran, Matthew Tolentino, Shannon Wickline, Kim Gietzen, Brad Sickler, Jingtao Liu, Eric Allen, Phil Febbo, Summer Galloway, Nicole L. Washington, Simon White, Geraint Levan, Kelly Schiabor Barrett, Elizabeth Cirulli, Alexandre Bolze, Ary Ascencio, Charlotte Rivera-Garcia, Ryan Cho, Jason Nguyen, Sherry Wang, Jimmy Ramirez, Tyler Cassens, Efren Sandoval, Magnus Isaksson, William Lee, David Becker, Marc Laurent, James Lu, Clinton R. Paden, Suxiang Tong, Duncan MacCannell
EPI_ISL_1355284	Universitätsklinikum Leipzig - Institut für Medizinische Mikrobiologie und Virologie virologisches Labor	Robert Koch Institute	unknown
EPI_ISL_1360284	Hospital Universitari Bellvitge	Microbiology Department	Sara Marti, Aida Gonzalez-Diaz, Laura Calatayud, Jordi Niubó, Miguel Fernandez-Huerta, Carmen Ardanuy, Jordi Camara, M Angeles Domínguez
EPI_ISL_1394456	Hospital Universitari Vall d'Hebron - Vall d'Hebron Institut de Recerca	Hospital Universitari Vall d'Hebron - Vall d'Hebron Institut de Recerca	Cristina Andrés, Maria Piñana, Josep F Abril, Damir Garcia-Cehic, Ariadna Rando, Juliana Esperalba, Maria Gema Codina, Carla Castillo, Maria Carmen Martin, Tomàs Pumarola, Josep Quer, Andrés Antón
EPI_ISL_1395792	Laboratorio de Virología del Hospital de Niños Dr. Ricardo Gutierrez	Área de Secuenciación del Laboratorio de Virología del Hospital de Niños Dr. Ricardo Gutierrez on behalf of 'Proyecto Argentino Interinstitucional de genomica de SARS-CoV-2' (PAIS Consortium)	Alexay, S; Thomas, G; Medina, C; Labarta, N; Streitenberger, C; Villegas, E; Barreda Frank, M; Grandis, E; Acevedo, ME; Alvarez Lopez, C; Jacques, O; Mistchenko, A; Nabaes Jodar, M; Goya, S; Lusso, S; Acuña, D; Natale, MI; Valinotto, LE; Viegas, M.
EPI_ISL_1402899	Johns Hopkins Hospital Department of Pathology	Johns Hopkins Hospital Department of Pathology	C. Paul Morris, Chun Huai Luo, Adannaya Amadi, Matthew Schwartz, Heba H. Mostafa
EPI_ISL_1406037, EPI_ISL_1406062	Microbiology Department, Laboratori Clínic Metropolitana Nord. Hospital Universitari Germans Trias i Pujol.	Can Ruti SARS-CoV-2 Sequencing Hub (HUGTiP/IrsiCaixa/IGTP)	Marc Noguera-Julian, Pilar Armengol, Ignacio Blanco, Antoni E Bordoy, Francesc Catala-Moll, Pere-Joan Cardona, Maria Casadellà, Cristina Casañ, Gemma Clara, Bonaventura Clotet, Cristina Esteban, Montserrat Giménez, Mercedes Guerrero, Anna Not, Roger Paredes, Mariona Parera, Verónica Saludes, Alba Sánchez, and Elisa Martró on behalf of the Can Ruti SARS-CoV-2 Sequencing Hub.
EPI_ISL_1465944	Lighthouse Lab in Cambridge	Wellcome Sanger Institute for the COVID-19 Genomics UK (COG-UK) Consortium	Rob Howes, The Lighthouse Lab in Cambridge and Alex Alderton, Roberto Amato, Jeffrey Barrett, Sonia Goncalves, Ewan Harrison, David K. Jackson, Ian Johnston, Dominic Kwiatkowski, Cordelia Langford, John Sillitoe on behalf of the Wellcome Sanger Institute COVID-19 Surveillance Team
EPI_ISL_1468575	Johns Hopkins Hospital Department of Pathology	Johns Hopkins Hospital Department of Pathology	C. Paul Morris, Chun Huai Luo, Adannaya Amadi, Matthew Schwartz, Heba H. Mostafa
EPI_ISL_1471732	Pandemic Response Lab - NYC	Pandemic Response Lab, R&D	Henry Lee, Michael Hammerling, Melissa Hopkins, Cybill del Castillo, Shinyoung Clair Kang, William Ward, Pradeep Bugga, Sol Rey, Dylan Law, Haiping Hao, Jon Laurent
EPI_ISL_1503243	Wichita State University - Molecular Diagnostics Lab	Kansas Health and Environmental Lab	Mike Grose, Jonathan Barnell, Ben Olsen, and Phil Adam
EPI_ISL_1524827	Hospital General Universitario Gregorio Marañón	Hospital General Universitario Gregorio Marañón	Sergio Buenestado Serrano, Pedro Sola Campoy, Laura Pérez-Lago, Cristina Rodriguez-Grande, Pilar Catalán, Patricia Muñoz, Darío García de Viedma
EPI_ISL_1528573	WVU Rapid Development Lab	WVU and Marshall University Combined Genomics Core Facilities	"James Denvir, Peter Stoilov, Peter Perrotta, Wesley Kimble, Ryan Percifield"
EPI_ISL_1543279	Pandemic Response Lab - NYC	Pandemic Response Lab, R&D	Henry Lee, Michael Hammerling, Melissa Hopkins, Cybill del Castillo, Shinyoung Clair Kang, William Ward, Pradeep Bugga, Sol Rey, Dylan Law, Katharine Nelson, Haiping Hao, Jon Laurent
EPI_ISL_1550427, EPI_ISL_1560274, EPI_ISL_1560525	Aegis Sciences Corporation	Centers for Disease Control and Prevention Division of Viral Diseases, Pathogen Discovery	Dakota Howard, Dhwani Batra, Peter W. Cook, Kara Moser, Adrian Paskey, Jason Caravas, Benjamin Rambo-Martin, Shatavia Morrison, Christopher Gulvick, Scott Sammons, Yvette Unoarumhi, Darlene Wagner, Matthew Schmerer, Cyndi Clark, Patrick Campbell, Rob Case, Vikramsinha Ghorpade, Holly Houdeshell, Ola Kvalvaag, Dillon Nall, Ethan Sanders, Alec Vest, Shaun Westlund, Matthew Hardison, Clinton R. Paden, Duncan MacCannell
EPI_ISL_1575599	Helix/Illumina	Centers for Disease Control and Prevention Division of Viral Diseases, Pathogen Discovery	Dakota Howard, Dhwani Batra, Peter W. Cook, Kara Moser, Adrian Paskey, Jason Caravas, Benjamin Rambo-Martin, Shatavia Morrison, Christopher Gulvick, Scott Sammons, Yvette Unoarumhi, Darlene Wagner, Matthew Schmerer, Eileen de Feo, Jan Antico, Christine Tran, Matthew Tolentino, Shannon

			Wickline, Kim Gietzen, Brad Sickler, Jingtao Liu, Eric Allen, Phil Febbo, Nicole L. Washington, Simon White, Geraint Levan, Kelly Schiabor Barrett, Elizabeth Cirulli, Alexandre Bolze, Ary Ascencio, Charlotte Rivera-Garcia, Ryan Cho, Jason Nguyen, Sherry Wang, Jimmy Ramirez, Tyler Cassens, Efren Sandoval, Magnus Isaksson, William Lee, David Becker, Marc Laurent, James Lu, Clinton R. Paden, Duncan MacCannell
EPI_ISL_1623327	NOVABIO DORDOGNE	CNR Virus des Infections Respiratoires - France SUD	Antonin Bal, Gregory Destras, Gwendolyne Burfin, Hadrien Regue, Quentin Semanas, Martine Valette, Bruno Lina, Laurence Josset
EPI_ISL_1667474	Department of Laboratory Medicine, National Taiwan University Hospital	Microbial Genomics Core Lab, National Taiwan University Centers of Genomic and Precision Medicine	Shiou-Hwei Yeh, You-Yu Lin, Ya-Yun Lai, Chiao-Ling Li, Shan-Chwen Chang, Pei-Jer Chen, Sui-Yuan Chang
EPI_ISL_1669359	Hospital Universitari Vall d'Hebron - Vall d'Hebron Institut de Recerca	Hospital Universitari Vall d'Hebron - Vall d'Hebron Institut de Recerca	Cristina Andrés, Maria Piñana, Damir Garcia-Cehic, Ariadna Rando, Juliana Esperalba, Maria Gema Codina, Carla Castillo, Maria Carmen Martin, Tomàs Pumarola, Josep Quer, Andrés Antón
EPI_ISL_1671609	NORTHWELL HEALTH LABORATORIES	Wadsworth Center, New York State Department of Health	Kirsten St. George, Daryl M. Lamson, Alexis Russell, Matthew Shudt, Melissa A Leisner, Jonathan Plitnick, Catharine Prussing, Navjot Singh, John Kelly, Erasmus Schneider, Erica Lasek-Nesselquist
EPI_ISL_1682599, EPI_ISL_1683246	Laboratory Corporation of America	Centers for Disease Control and Prevention Division of Viral Diseases, Pathogen Discovery	Dakota Howard, Dhwani Batra, Peter W. Cook, Kara Moser, Adrian Paskey, Jason Caravas, Benjamin Rambo-Martin, Shatavia Morrison, Christopher Gulvick, Scott Sammons, Yvette Unoarumhi, Darlene Wagner, Matthew Schmerer, Minoo Agarwal, Eyad Almasri, Debbie Boles, Ayla Burns, Nuthawin Charoensri, Oren Cohen, Susan Countryman, Mary Ann Cristobal, Bobbi Croy, Suzanne Dale, Hrushikesh Deshmukh, Amanda Douglas, Vincent Drouillon, Marcia Eisenberg, Howard Engler, Rama Ghatti, Prashant Gupta, Susan Hicks, Jake Humphrey, Lax Iyer, Manoj Jain, Mohan Kolli, Brian Krueger, Tim Kuphal, Stanley Letovsky, Michael Levandoski, Craig Lukasik, Jonathan Meltzer, Brian Norvell, Mindy Nye, Scott Parker, Christos Petropoulos, John Pruitt, Steven Ragan, Scott Ryan, Mike Sapeta, Jana Schroth, Suresh Babu Selvaraju, Goran Stevovic, Amanda Suchanek, Andrea Throop, Lyndon Tilson, Thomas Urban, Joe Voshell, Kimberly Wagner, Jonathan Williams, Mary Williamson, Qian Zeng, Tricia Zwiefelhofer, Clinton R. Paden, Duncan MacCannell
EPI_ISL_1688958, EPI_ISL_1689915, EPI_ISL_1690014, EPI_ISL_1690016	Aegis Sciences Corporation	Centers for Disease Control and Prevention Division of Viral Diseases, Pathogen Discovery	Dakota Howard, Dhwani Batra, Peter W. Cook, Kara Moser, Adrian Paskey, Jason Caravas, Benjamin Rambo-Martin, Shatavia Morrison, Christopher Gulvick, Scott Sammons, Yvette Unoarumhi, Darlene Wagner, Matthew Schmerer, Cyndi Clark, Patrick Campbell, Rob Case, Vikramsinha Ghorpade, Holly Houdeshell, Ola Kvalvaag, Dillon Nall, Ethan Sanders, Alec Vest, Shaun Westlund, Matthew Hardison, Clinton R. Paden, Duncan MacCannell
EPI_ISL_1694150, EPI_ISL_1694225	Infinity Biologix	Centers for Disease Control and Prevention Division of Viral Diseases, Pathogen Discovery	Dakota Howard, Dhwani Batra, Peter W. Cook, Kara Moser, Adrian Paskey, Jason Caravas, Benjamin Rambo-Martin, Shatavia Morrison, Christopher Gulvick, Scott Sammons, Yvette Unoarumhi, Darlene Wagner, Matthew Schmerer, Christian Bixby, Yihe Wang, Jonathan Schultz, Chirayu Goswami, Russ Hager, Robin Grimwood, Clinton R. Paden, Duncan MacCannell
EPI_ISL_1728276	SYNLAB MVZ Leverkusen	Robert Koch Institute	unknown
EPI_ISL_1737004	Aegis Sciences Corporation	Centers for Disease Control and Prevention Division of Viral Diseases, Pathogen Discovery	Dakota Howard, Dhwani Batra, Peter W. Cook, Kara Moser, Adrian Paskey, Jason Caravas, Benjamin Rambo-Martin, Shatavia Morrison, Christopher Gulvick, Scott Sammons, Yvette Unoarumhi, Darlene Wagner, Matthew Schmerer, Cyndi Clark, Patrick Campbell, Rob Case, Vikramsinha Ghorpade, Holly Houdeshell, Ola Kvalvaag, Dillon Nall, Ethan Sanders, Alec Vest, Shaun Westlund, Matthew Hardison, Clinton R. Paden, Duncan MacCannell
EPI_ISL_1805222	Maryland Genomics, Institute for Genome Sciences, University of Maryland School of Medicine	Maryland Genomics, Institute for Genome Sciences, University of Maryland School of Medicine	Tallon, Luke J; Sadzewicz, Lisa D; Humphrys, Mike; Ott, Sandra; Roussey, Holly; Mehta, Aditya; Vavikolanu, Kranthi; Fraser, Claire M; Ravel, Jacques
EPI_ISL_1807213	GA Department of Public Health	GA Department of Public Health	Stacy Reeves, Jonathan Edwards, Cynthia Dixey, Tonia Parrott, Aliyah Fields, Taylor Smith
EPI_ISL_1836219, EPI_ISL_1838360	Aegis Sciences Corporation	Centers for Disease Control and Prevention Division of Viral Diseases, Pathogen Discovery	Dakota Howard, Dhwani Batra, Peter W. Cook, Kara Moser, Adrian Paskey, Jason Caravas, Benjamin Rambo-Martin, Shatavia Morrison, Christopher Gulvick, Scott Sammons, Yvette Unoarumhi, Darlene Wagner, Matthew Schmerer, Cyndi Clark, Patrick Campbell, Rob Case, Vikramsinha Ghorpade, Holly Houdeshell, Ola Kvalvaag, Dillon Nall, Ethan Sanders, Alec Vest, Shaun Westlund, Matthew Hardison, Clinton R. Paden, Duncan MacCannell
EPI_ISL_1841485, EPI_ISL_1841520, EPI_ISL_1841707	Department of Virology and Immunology, University of Helsinki and Helsinki University Hospital, Huslab Finland	Department of Virology, Faculty of Medicine, University of Helsinki, Helsinki, Finland	Teemu Smura, Ravi Kant, Phuoc Truong, Hussein Alburkat, Hannimari Kallio-Kokko, Jenni Virtanen, Maija Suvanto, Essi Korhonen, Sari Hannula, Harri Kangas, Hanna Liimatainen, Satu Kurkela, Hanna Jarva, Maija Lappalainen, Pekka Ellonen, Olli Vapalahti
EPI_ISL_1843792	Centogene; Dr. Bauer Laboratoriums GmbH	Robert Koch Institute	unknown
EPI_ISL_1844122	Bioscientia Labor Wermsdorf	Robert Koch Institute	unknown
EPI_ISL_1844532	IMD - MVZ Labor Martinsried	Robert Koch Institute	unknown
EPI_ISL_1882007	Department of Virus and Microbiological Special Diagnostics, Statens Serum Institut, Copenhagen, Denmark	Aalborg University	Danish Covid-19 Genome Consortium
EPI_ISL_1922367	Servicio de Microbiología Clínica (Complejo Hospitalario de Navarra, Pamplona)	Centro de Secuenciación NASERTIC	Carmen Ezpeleta Baquedano, Ana Navascués, Ana Miqueleiz
EPI_ISL_1925276	Aegis Sciences Corporation	Centers for Disease Control and Prevention Division of Viral Diseases, Pathogen Discovery	Dakota Howard, Dhwani Batra, Peter W. Cook, Kara Moser, Adrian Paskey, Jason Caravas, Benjamin Rambo-Martin, Shatavia Morrison, Christopher Gulvick, Scott Sammons, Yvette Unoarumhi, Darlene Wagner, Matthew Schmerer, Cyndi Clark, Patrick Campbell, Rob Case, Vikramsinha Ghorpade, Holly Houdeshell, Ola Kvalvaag, Dillon Nall, Ethan Sanders, Alec Vest, Shaun Westlund, Matthew Hardison, Clinton R. Paden, Duncan MacCannell
EPI_ISL_1964068, EPI_ISL_1964073	Hospital Universitari Bellvitge	Microbiology Department	Sara Marti, Aida Gonzalez-Diaz, Laura Calatayud, Jordi Niubó, Miguel Fernandez-Huerta, Carmen Ardanuy, Jordi Camara, M Angeles Domínguez
EPI_ISL_1971160	Weill Cornell Medicine	New York Genome Center	Michael Zody, Andre Corvelo, Dayna M. Oschwald, Samantha Fennessey, Tom Maniatis, Melissa Cushing, Olivier Elemento, Margaret Elizabeth Ross, Chris Mason, Priya Velu, Hanna Rennert, Arryn Craney, Lars F Westblade
EPI_ISL_1972559	Laboratorio HUB -Azienda Ospedaliero Universitaria - AOU - Cagliari	Laboratorio SPOKE Biologia Molecolare -Azienda Ospedaliero Universitaria - AOU - Cagliari	Germano Orrù, Sara Fais, Valentina Medda, Alessandra Scano, Miriam Loddo, Riccardo Cappai, Ferdinando Coghe
EPI_ISL_1991693	Helix/Illumina	Centers for Disease Control and Prevention Division of Viral Diseases, Pathogen Discovery	Dakota Howard, Dhwani Batra, Peter W. Cook, Kara Moser, Adrian Paskey, Jason Caravas, Benjamin Rambo-Martin, Shatavia Morrison, Christopher Gulvick, Scott Sammons, Yvette Unoarumhi, Darlene Wagner, Matthew Schmerer, Eileen de Feo, Jan Antico, Christine Tran, Matthew Tolentino, Shannon Wickline, Kim Gietzen, Brad Sickler, Jingtao Liu, Eric Allen, Phil Febbo, Nicole L. Washington, Simon White, Geraint Levan, Kelly Schiabor Barrett, Elizabeth Cirulli, Alexandre Bolze, Ary Ascencio, Charlotte Rivera-Garcia, Ryan Cho, Jason Nguyen, Sherry Wang, Jimmy Ramirez, Tyler Cassens, Efren Sandoval, Magnus Isaksson, William Lee, David Becker, Marc Laurent, James Lu, Clinton R. Paden, Duncan MacCannell
EPI_ISL_1995941, EPI_ISL_1996423, EPI_ISL_1996557, EPI_ISL_2000300	Aegis Sciences Corporation	Centers for Disease Control and Prevention Division of Viral Diseases, Pathogen Discovery	Dakota Howard, Dhwani Batra, Peter W. Cook, Kara Moser, Adrian Paskey, Jason Caravas, Benjamin Rambo-Martin, Shatavia Morrison, Christopher Gulvick, Scott Sammons, Yvette Unoarumhi, Darlene Wagner, Matthew Schmerer, Cyndi Clark, Patrick Campbell, Rob Case, Vikramsinha Ghorpade, Holly Houdeshell, Ola Kvalvaag, Dillon Nall, Ethan Sanders, Alec Vest, Shaun Westlund, Matthew Hardison, Clinton R. Paden, Duncan MacCannell
EPI_ISL_2003803, EPI_ISL_2003928	Hospital of the University of Pennsylvania Molecular Pathology Lab	Bushman Lab - University of Pennsylvania	John Everett, Kyle Rodino, Shantan Reddy, Pascha Hokama, Aoife M. Roche, Young Hwang, Abigail Glascock, Scott Sherrill-Mix, Samantha A. Whiteside, Jevon Graham-Wooten, Layla A. Khatib, Ayannah S. Fitzgerald, Arupa Ganguly, Mike Feldman, Brendan Kelly, Ronald G. Collman and Frederic Bushman
EPI_ISL_2007474	Hospital General de Agudos Dr. Cosme Argerich	Área de Secuenciación del Laboratorio de Virología del Hospital de Niños Dr. Ricardo Gutierrez on behalf of 'Proyecto Argentino Interinstitucional de genomica de SARS-CoV-2' (PAIS Consortium)	Marcia Pozzati, Jésica Galeano, Florencia Rodríguez, Florencia Funez, Andrea Fernández, Karina Polanski; Alexay, S; Nabaes Jodar, M; Acuña, D; Goya, S; Lusso, S; Natale, MI; Valinotto, LE; Viegas, M.
EPI_ISL_2007479, EPI_ISL_2007483	Laboratorio de Virología del Hospital de Niños Dr. Ricardo Gutierrez	Área de Secuenciación del Laboratorio de Virología del Hospital de Niños Dr. Ricardo Gutierrez on behalf of 'Proyecto Argentino Interinstitucional de genomica de SARS-CoV-2' (PAIS Consortium)	Alexay, S; Thomas, G; Medina, C; Labarta, N; Streitenberger, C; Villegas, E; Barreda Frank, M; Grandis, E; Acevedo, ME; Alvarez Lopez, C; Jacques, O; Mistchenko, A; Nabaes Jodar, M; Goya, S; Lusso, S; Acuña, D; Natale, MI; Valinotto, LE; Viegas, M.
EPI_ISL_2007484	Hospital General de Agudos Dr. Cosme Argerich	Área de Secuenciación del Laboratorio de Virología del Hospital de Niños Dr. Ricardo Gutierrez on behalf of 'Proyecto Argentino Interinstitucional de genomica de SARS-CoV-2' (PAIS Consortium)	Marcia Pozzati, Jésica Galeano, Florencia Rodríguez, Florencia Funez, Andrea Fernández, Karina Polanski; Alexay, S; Nabaes Jodar, M; Acuña, D; Goya, S; Lusso, S; Natale, MI; Valinotto, LE; Viegas, M.
EPI_ISL_2007485, EPI_ISL_2007487,	Laboratorio de Virología del Hospital de Niños Dr. Ricardo	Área de Secuenciación del Laboratorio de Virología del	Alexay, S; Thomas, G; Medina, C; Labarta, N; Streitenberger, C; Villegas, E; Barreda Frank, M; Grandis, E; Acevedo, ME; Alvarez Lopez, C; Jacques, O;

EPI_ISL_2007491, EPI_ISL_2007492, EPI_ISL_2007497, EPI_ISL_2007498, EPI_ISL_2007499, EPI_ISL_2007500	Gutierrez	Hospital de Niños Dr. Ricardo Gutierrez on behalf of 'Proyecto Argentino Interinstitucional de genomica de SARS-CoV-2' (PAIS Consortium)	Mistchenko, A; Nabaes Jodar, M; Goya, S; Lusso, S; Acuña, D; Natale, MI; Valinotto, LE; Viegas, M.
EPI_ISL_2007501	Laboratorio Central, Ministerio de Salud Córdoba	Instituto de Patologia Vegetal (CIAP-INTA) on behalf of 'Proyecto Argentino Interinstitucional de genomica de SARS-CoV-2' (PAIS Consortium)	Fernández, FD; Marquez, N.; Debat, HJ.; Amadio, A; Irazoqui, M; Re, V.; Pisano, M.B.; Castro, G.; Barbas, G.
EPI_ISL_2007505	Hospital General de Agudos Dr. Cosme Argerich	Área de Secuenciación del Laboratorio de Virología del Hospital de Niños Dr. Ricardo Gutierrez on behalf of 'Proyecto Argentino Interinstitucional de genomica de SARS-CoV-2' (PAIS Consortium)	Marcia Pozzati, Jésica Galeano, Florencia Rodríguez, Florencia Funez, Andrea Fernández, Karina Polanski; Alexay, S; Nabaes Jodar, M; Acuña, D; Goya, S; Lusso, S; Natale, MI; Valinotto, LE; Viegas, M.
EPI_ISL_2007506, EPI_ISL_2007507, EPI_ISL_2007508, EPI_ISL_2007509, EPI_ISL_2007510, EPI_ISL_2007511, EPI_ISL_2007512, EPI_ISL_2007513	Laboratorio de Virología del Hospital de Niños Dr. Ricardo Gutierrez	Área de Secuenciación del Laboratorio de Virología del Hospital de Niños Dr. Ricardo Gutierrez on behalf of 'Proyecto Argentino Interinstitucional de genomica de SARS-CoV-2' (PAIS Consortium)	Alexay, S; Thomas, G; Medina, C; Labarta, N; Streitenberger, C; Villegas, E; Barreda Frank, M; Grandis, E; Acevedo, ME; Alvarez Lopez, C; Jacques, O; Mistchenko, A; Nabaes Jodar, M; Goya, S; Lusso, S; Acuña, D; Natale, MI; Valinotto, LE; Viegas, M.
EPI_ISL_2007514, EPI_ISL_2007515, EPI_ISL_2007516	Laboratorio Central, Ministerio de Salud Córdoba	Instituto de Patologia Vegetal (CIAP-INTA) on behalf of 'Proyecto Argentino Interinstitucional de genomica de SARS-CoV-2' (PAIS Consortium)	Fernández, FD; Marquez, N.; Debat, HJ.; Amadio, A; Irazoqui, M; Re, V.; Pisano, M.B.; Castro, G.; Barbas, G.
EPI_ISL_2007517, EPI_ISL_2007518	Laboratorio de Virología del Hospital de Niños Dr. Ricardo Gutierrez	Área de Secuenciación del Laboratorio de Virología del Hospital de Niños Dr. Ricardo Gutierrez on behalf of 'Proyecto Argentino Interinstitucional de genomica de SARS-CoV-2' (PAIS Consortium)	Alexay, S; Thomas, G; Medina, C; Labarta, N; Streitenberger, C; Villegas, E; Barreda Frank, M; Grandis, E; Acevedo, ME; Alvarez Lopez, C; Jacques, O; Mistchenko, A; Nabaes Jodar, M; Goya, S; Lusso, S; Acuña, D; Natale, MI; Valinotto, LE; Viegas, M.
EPI_ISL_2007521	Hospital General de Agudos Dr. Cosme Argerich	Área de Secuenciación del Laboratorio de Virología del Hospital de Niños Dr. Ricardo Gutierrez on behalf of 'Proyecto Argentino Interinstitucional de genomica de SARS-CoV-2' (PAIS Consortium)	Marcia Pozzati, Jésica Galeano, Florencia Rodríguez, Florencia Funez, Andrea Fernández, Karina Polanski; Alexay, S; Nabaes Jodar, M; Acuña, D; Goya, S; Lusso, S; Natale, MI; Valinotto, LE; Viegas, M.
EPI_ISL_2007522, EPI_ISL_2007537, EPI_ISL_2007544	Laboratorio de Virología del Hospital de Niños Dr. Ricardo Gutierrez	Área de Secuenciación del Laboratorio de Virología del Hospital de Niños Dr. Ricardo Gutierrez on behalf of 'Proyecto Argentino Interinstitucional de genomica de SARS-CoV-2' (PAIS Consortium)	Alexay, S; Thomas, G; Medina, C; Labarta, N; Streitenberger, C; Villegas, E; Barreda Frank, M; Grandis, E; Acevedo, ME; Alvarez Lopez, C; Jacques, O; Mistchenko, A; Nabaes Jodar, M; Goya, S; Lusso, S; Acuña, D; Natale, MI; Valinotto, LE; Viegas, M.
EPI_ISL_2010570	Helix/Illumina	Centers for Disease Control and Prevention Division of Viral Diseases, Pathogen Discovery	Dakota Howard, Dhwani Batra, Peter W. Cook, Kara Moser, Adrian Paskey, Jason Caravas, Benjamin Rambo-Martin, Shatavia Morrison, Christopher Gulvick, Scott Sammons, Yvette Unoarumhi, Darlene Wagner, Matthew Schmerer, Eileen de Feo, Jan Antico, Christine Tran, Matthew Tolentino, Shannon Wickline, Kim Gietzen, Brad Sickler, Jingtao Liu, Eric Allen, Phil Febbo, Nicole L. Washington, Simon White, Geraint Levan, Kelly Schiabor Barrett, Elizabeth Cirulli, Alexandre Bolze, Ary Ascencio, Charlotte Rivera-Garcia, Ryan Cho, Jason Nguyen, Sherry Wang, Jimmy Ramirez, Tyler Cassens, Efren Sandoval, Magnus Isaksson, William Lee, David Becker, Marc Laurent, James Lu, Clinton R. Paden, Duncan MacCannell
EPI_ISL_2023146	Yale Clinical Virology Lab	Grubaugh Lab - Yale School of Public Health	Joseph Fauver, Mallery Breban, Isabel Ott, Tara Alpert, Mary Petrone, Anderson Brito, Chantal Vogels, Annie Watkins, Chaney Kalinich, Jessica Rothman, Marie L. Landry, Nathan Grubaugh
EPI_ISL_2040651, EPI_ISL_2042190, EPI_ISL_2042280	Aegis Sciences Corporation	Centers for Disease Control and Prevention Division of Viral Diseases, Pathogen Discovery	Dakota Howard, Dhwani Batra, Peter W. Cook, Kara Moser, Adrian Paskey, Jason Caravas, Benjamin Rambo-Martin, Shatavia Morrison, Christopher Gulvick, Scott Sammons, Yvette Unoarumhi, Darlene Wagner, Matthew Schmerer, Cyndi Clark, Patrick Campbell, Rob Case, Vikramsinha Ghorpade, Holly Houdeshell, Ola Kvalvaag, Dillon Nall, Ethan Sanders, Alec Vest, Shaun Westlund, Matthew Hardison, Clinton R. Paden, Duncan MacCannell
EPI_ISL_2083473	Israel Central Virology laboratory	Israel National Consortium for SARS-CoV-2 sequencing	Neta Zuckerman, Efrat Dahan Bucris, Michal Mandelboim, Dana Bar-Ilan, Oran Erster, Tzvia Mann, Omer Murik, David A. Zeevi, Assaf Rokney, Joseph Jaffe, Eva Nachum, Maya Davidovich Cohen, Ephraim Fass, Gal Zizelski Valenci, Mor Rubinstein, Efrat Rorman, Israel Nissan, Efrat Glick-Saar, Omri Nayshool, Gideon Rechavi, Ella Mendelson, Orna Mor
EPI_ISL_2085580	Israel Central Virology laboratory	Israel National Consortium for SARS-CoV-2 sequencing	Neta Zuckerman, Efrat Dahan Bucris, Michal Mandelboim, Dana Bar-Ilan, Miranda Geva, Netanel Abu, Oran Erster, Efrat Glick-Saar, Omri Nayshool, Gideon Rechavi, Ella Mendelson, Orna Mor
EPI_ISL_2089827	Aegis Sciences Corporation	Centers for Disease Control and Prevention Division of Viral Diseases, Pathogen Discovery	Dakota Howard, Dhwani Batra, Peter W. Cook, Kara Moser, Adrian Paskey, Jason Caravas, Benjamin Rambo-Martin, Shatavia Morrison, Christopher Gulvick, Scott Sammons, Yvette Unoarumhi, Darlene Wagner, Matthew Schmerer, Cyndi Clark, Patrick Campbell, Rob Case, Vikramsinha Ghorpade, Holly Houdeshell, Ola Kvalvaag, Dillon Nall, Ethan Sanders, Alec Vest, Shaun Westlund, Matthew Hardison, Clinton R. Paden, Duncan MacCannell
EPI_ISL_2096430	Broad Institute Clinical Research Sequencing Platform	Infectious Disease Program, Broad Institute of Harvard and MIT	Siddle,K.J., Adams,G., Pearlman,L., Gladden-Young,A., Vicente,G., Blumenstiel,B., DeFelice,M., Lee,M., McGovern,S., Lagerborg,K., Rudy,M., DeRuff,K., Carter,A., Normandin,E., Bauer,M., Reilly,S., Tomkins-Tinch,C., Loreth,C., Chaluvadi,S., Meldrim,J., Granger,B., Lemieux,J.E., Birren,B.W., Sabeti,P.C., Larkin,K., Dodge,S., Lennon,N., Madoff,L., Brown,C., Gallagher,G., Smole,S., Park,D.J., Gabriel,S., and MacInnis,B.L.
EPI_ISL_2096846, EPI_ISL_2096856	Hadassah Medical Center Clinical Virology Laboratory, Hadassah Ein Kerem	Hadassah Hebrew University Viral Sequencing Group, Hadassah Hebrew University Medical Center	Hadar Golan Berman, Esther Oiknine-Djian, Mila Rivkin, Sheera Adar, Dana G. Wolf
EPI_ISL_2101894	LESP Guanajuato	Instituto de Diagnostico y Referencia Epidemiologicos (INDRE)	Claudia Wong-Arambula, Abril Rodriguez-Maldonado, Vanessa Rivero-Arredondo, Ariadna Medina-Benitez, Joaquin Quiroz-Mercado, Sergio Rangel-Guerrero, Natividad Cruz-Ortiz, Tatiana Nunez-Garcia, Gisela Barrera-Badillo, Lucia Hernandez-Rivas, Irma Lopez-Martinez, Ernesto Ramirez-Gonzalez.
EPI_ISL_2101899	LESP Quintana Roo	Instituto de Diagnostico y Referencia Epidemiologicos (INDRE)	Claudia Wong-Arambula, Abril Rodriguez-Maldonado, Vanessa Rivero-Arredondo, Ariadna Medina-Benitez, Joaquin Quiroz-Mercado, Sergio Rangel-Guerrero, Natividad Cruz-Ortiz, Tatiana Nunez-Garcia, Gisela Barrera-Badillo, Lucia Hernandez-Rivas, Irma Lopez-Martinez, Ernesto Ramirez-Gonzalez.
EPI_ISL_2102372, EPI_ISL_2102374, EPI_ISL_2102375	Maryland Genomics, Institute for Genome Sciences, University of Maryland School of Medicine	Maryland Genomics, Institute for Genome Sciences, University of Maryland School of Medicine	Tallon, Luke J; Sadzewicz, Lisa D; Humphrys, Mike; Ott, Sandra; Roussey, Holly; Mehta, Aditya; Vavikolanu, Kranthi; Fraser, Claire M; Ravel, Jacques
EPI_ISL_2105822	Salud Digna	Instituto Nacional de Medicina Genomica	Hidalgo-Miranda A, Cedro-Tanda A, Mendoza-Vargas A, Reyes-Grajeda JP, Abraham Campos-Romero, Moreno-Camacho José Luis, Rodriguez-Gallegos Jorge, Luna-Ruiz Marco, Gonzalez-Barrera D, Rangel-DeLeon D, Munguia-Garza P, Ramirez-Vega O, Escobar-Arrazola, M, Herrera-Montalvo LA.
EPI_ISL_2109730	Sonic - Labor Dr. von Froreich GmbH	Robert Koch Institute	unknown
EPI_ISL_2122995	Labor Dr. Heidrich & Kollegen MVZ GmbH Hamburg	Robert Koch Institute	unknown
EPI_ISL_2133176	Quest Diagnostics Incorporated	Centers for Disease Control and Prevention Division of Viral Diseases, Pathogen Discovery	Dakota Howard, Dhwani Batra, Peter W. Cook, Kara Moser, Adrian Paskey, Jason Caravas, Benjamin Rambo-Martin, Shatavia Morrison, Christopher Gulvick, Scott Sammons, Yvette Unoarumhi, Darlene Wagner, Matthew Schmerer, S. H. Rosenthal, A. Gerasimova, R. M. Kagan, B. Anderson, M. Hua, Y. Liu, L.E. Bernstein, K.E. Livingston, A. Perez, I. A. Shlyakhter, R. V. Rolando, R. Owen, P. Tanpaiboon, F. Lacbawan, Clinton R. Paden, Duncan MacCannell
EPI_ISL_2133399, EPI_ISL_2133401	Servicio Virosis Respiratorias-Departamento Virología-INEI	Instituto Nacional Enfermedades Infecciosas C.G.Malbran	Baumeister E., Avaro M., Benedetti E., Russo M., Dattero ME, Pontoriero A., Cisterna D., Molina V., Perandones C., Tuduri E., Lorenzo F., Poklepovich T., Campos J.
EPI_ISL_2133705	Quest Diagnostics Incorporated	Centers for Disease Control and Prevention Division of Viral Diseases, Pathogen Discovery	Dakota Howard, Dhwani Batra, Peter W. Cook, Kara Moser, Adrian Paskey, Jason Caravas, Benjamin Rambo-Martin, Shatavia Morrison, Christopher Gulvick, Scott Sammons, Yvette Unoarumhi, Darlene Wagner, Matthew Schmerer, S. H. Rosenthal, A. Gerasimova, R. M. Kagan, B. Anderson, M. Hua, Y. Liu, L.E. Bernstein, K.E. Livingston, A. Perez, I. A. Shlyakhter, R. V. Rolando, R. Owen, P. Tanpaiboon, F. Lacbawan, Clinton R. Paden, Duncan MacCannell

EPI_ISL_2135137, EPI_ISL_2135150, EPI_ISL_2135255, EPI_ISL_2135326, EPI_ISL_2135327, EPI_ISL_2135336, EPI_ISL_2135337, EPI_ISL_2135338, EPI_ISL_2135342, EPI_ISL_2135697, EPI_ISL_2135698, EPI_ISL_2135699, EPI_ISL_2135722, EPI_ISL_2135723, EPI_ISL_2135725, EPI_ISL_2136003, EPI_ISL_2136010,

EPI_ISL_2136023, EPI_ISL_2136027, EPI_ISL_2136028, EPI_ISL_2136036, EPI_ISL_2136044, EPI_ISL_2136046, EPI_ISL_2136049, EPI_ISL_2136078, EPI_ISL_2136079, EPI_ISL_2136080, EPI_ISL_2136081, EPI_ISL_2136083, EPI_ISL_2136084, EPI_ISL_2136085, EPI_ISL_2136086, EPI_ISL_2136087, EPI_ISL_2136088, EPI_ISL_2136089, EPI_ISL_2136091, EPI_ISL_2136093, EPI_ISL_2136095, EPI_ISL_2136098, EPI_ISL_2136140, EPI_ISL_2136149, EPI_ISL_2136164, EPI_ISL_2136178, EPI_ISL_2136179, EPI_ISL_2136180			
see above	Servicio Virosis Respiratorias-Departamento Virología-INEI	Instituto Nacional Enfermedades Infecciosas C.G.Malbran	Baumeister E., Avaro M., Benedetti E., Russo M., Dattero ME, Pontoriero A., Cisterna D., Molina V., Perandones C., Tuduri E., Lorenzo F., Poklepovich T., Campos J.
EPI_ISL_2139974	Illinois Department of Public Health	Illinois Department of Public Health - Chicago Lab	Vineet K. Dhiman, Ira Heimler, Joel Price
EPI_ISL_2140053, EPI_ISL_2140056, EPI_ISL_2140064, EPI_ISL_2140068, EPI_ISL_2140070, EPI_ISL_2140074, EPI_ISL_2140104, EPI_ISL_2140105, EPI_ISL_2140110, EPI_ISL_2140132	Servicio Virosis Respiratorias-Departamento Virología-INEI	Instituto Nacional Enfermedades Infecciosas C.G.Malbran	Baumeister E., Avaro M., Benedetti E., Russo M., Dattero ME, Pontoriero A., Cisterna D., Molina V., Perandones C., Tuduri E., Lorenzo F., Poklepovich T., Campos J.
EPI_ISL_2148039, EPI_ISL_2148642, EPI_ISL_2149693, EPI_ISL_2149704, EPI_ISL_2149794, EPI_ISL_2150432, EPI_ISL_2150525, EPI_ISL_2150807	Aegis Sciences Corporation	Centers for Disease Control and Prevention Division of Viral Diseases, Pathogen Discovery	Dakota Howard, Dhwani Batra, Peter W. Cook, Kara Moser, Adrian Paskey, Jason Caravas, Benjamin Rambo-Martin, Shatavia Morrison, Christopher Gulvick, Scott Sammons, Yvette Unoarumhi, Darlene Wagner, Matthew Schmerer, Cyndi Clark, Patrick Campbell, Rob Case, Vikramsinha Ghorpade, Holly Houdeshell, Ola Kvalvaag, Dillon Nall, Ethan Sanders, Alec Vest, Shaun Westlund, Matthew Hardison, Clinton R. Paden, Duncan MacCannell
EPI_ISL_2158694, EPI_ISL_2158696, EPI_ISL_2158699, EPI_ISL_2158711, EPI_ISL_2158719, EPI_ISL_2158725, EPI_ISL_2158726, EPI_ISL_2158731, EPI_ISL_2158733, EPI_ISL_2158736, EPI_ISL_2158743, EPI_ISL_2158749, EPI_ISL_2158751, EPI_ISL_2158775, EPI_ISL_2158779, EPI_ISL_2158780, EPI_ISL_2158784, EPI_ISL_2158786, EPI_ISL_2158791, EPI_ISL_2158804, EPI_ISL_2158806, EPI_ISL_2158819, EPI_ISL_2158826, EPI_ISL_2158830, EPI_ISL_2158837, EPI_ISL_2158842, EPI_ISL_2158848			
see above	Servicio Virosis Respiratorias-Departamento Virología-INEI	Instituto Nacional Enfermedades Infecciosas C.G.Malbran	Baumeister E., Avaro M., Benedetti E., Russo M., Dattero ME, Pontoriero A., Cisterna D., Molina V., Perandones C., Tuduri E., Lorenzo F., Poklepovich T., Campos J.
EPI_ISL_2159924, EPI_ISL_2159930, EPI_ISL_2159981, EPI_ISL_2160142	Helix/Illumina	Centers for Disease Control and Prevention Division of Viral Diseases, Pathogen Discovery	Dakota Howard, Dhwani Batra, Peter W. Cook, Kara Moser, Adrian Paskey, Jason Caravas, Benjamin Rambo-Martin, Shatavia Morrison, Christopher Gulvick, Scott Sammons, Yvette Unoarumhi, Darlene Wagner, Matthew Schmerer, Eileen de Feo, Jan Antico, Christine Tran, Matthew Tolentino, Shannon Wickline, Kim Gietzen, Brad Sickler, Jingtao Liu, Eric Allen, Phil Febbo, Nicole L. Washington, Simon White, Geraint Levan, Kelly Schiabor Barrett, Elizabeth Cirulli, Alexandre Bolze, Ary Ascencio, Charlotte Rivera-Garcia, Ryan Cho, Jason Nguyen, Sherry Wang, Jimmy Ramirez, Tyler Cassens, Efren Sandoval, Magnus Isaksson, William Lee, David Becker, Marc Laurent, James Lu, Clinton R. Paden, Duncan MacCannell
EPI_ISL_2161453	Maryland Genomics, Institute for Genome Sciences, University of Maryland School of Medicine	Maryland Genomics, Institute for Genome Sciences, University of Maryland School of Medicine	Tallon, Luke J; Sadzewicz, Lisa D; Humphrys, Mike; Ott, Sandra; Roussey, Holly; Mehta, Aditya; Vavikolanu, Kranthi; Fraser, Claire M; Ravel, Jacques
EPI_ISL_2180667, EPI_ISL_2181126	Aegis Sciences Corporation	Centers for Disease Control and Prevention Division of Viral Diseases, Pathogen Discovery	Dakota Howard, Dhwani Batra, Peter W. Cook, Kara Moser, Adrian Paskey, Jason Caravas, Benjamin Rambo-Martin, Shatavia Morrison, Christopher Gulvick, Scott Sammons, Yvette Unoarumhi, Darlene Wagner, Matthew Schmerer, Cyndi Clark, Patrick Campbell, Rob Case, Vikramsinha Ghorpade, Holly Houdeshell, Ola Kvalvaag, Dillon Nall, Ethan Sanders, Alec Vest, Shaun Westlund, Matthew Hardison, Clinton R. Paden, Duncan MacCannell
EPI_ISL_2182966, EPI_ISL_2183218, EPI_ISL_2183609, EPI_ISL_2183795	Israel Central Virology laboratory	Israel National Consortium for SARS-CoV-2 sequencing	Neta Zuckerman, Efrat Dahan Bucris, Michal Mandelboim, Dana Bar-Ilan, Miranda Geva, Netanel Abu, Oran Erster, Efrat Glick-Saar, Omri Nayshool, Gideon Rechavi, Ella Mendelson, Orna Mor
EPI_ISL_2186247, EPI_ISL_2187561	Aegis Sciences Corporation	Centers for Disease Control and Prevention Division of Viral Diseases, Pathogen Discovery	Dakota Howard, Dhwani Batra, Peter W. Cook, Kara Moser, Adrian Paskey, Jason Caravas, Benjamin Rambo-Martin, Shatavia Morrison, Christopher Gulvick, Scott Sammons, Yvette Unoarumhi, Darlene Wagner, Matthew Schmerer, Cyndi Clark, Patrick Campbell, Rob Case, Vikramsinha Ghorpade, Holly Houdeshell, Ola Kvalvaag, Dillon Nall, Ethan Sanders, Alec Vest, Shaun Westlund, Matthew Hardison, Clinton R. Paden, Duncan MacCannell
EPI_ISL_2192957	Broad Institute Clinical Research Sequencing Platform	Infectious Disease Program, Broad Institute of Harvard and MIT	Siddle,K.J., Adams,G., Pearlman,L., Gladden-Young,A., Vicente,G., Blumenstiel,B., DeFelice,M., Lee,M., McGovern,S., Lagerborg,K., Rudy,M., DeRuff,K., Carter,A., Normandin,E., Bauer,M., Reilly,S., Tomkins-Tinch,C., Loreth,C., Chaluvadi,S., Meldrim,J., Granger,B., Lemieux,J.E., Birren,B.W., Sabeti,P.C., Larkin,K., Dodge,S., Lennon,N., Madoff,L., Brown,C., Gallagher,G., Smole,S., Park,D.J., Gabriel,S., and MacInnis,B.L.
EPI_ISL_2201766	Aegis Sciences Corporation	Centers for Disease Control and Prevention Division of Viral Diseases, Pathogen Discovery	Dakota Howard, Dhwani Batra, Peter W. Cook, Kara Moser, Adrian Paskey, Jason Caravas, Benjamin Rambo-Martin, Shatavia Morrison, Christopher Gulvick, Scott Sammons, Yvette Unoarumhi, Darlene Wagner, Matthew Schmerer, Cyndi Clark, Patrick Campbell, Rob Case, Vikramsinha Ghorpade, Holly Houdeshell, Ola Kvalvaag, Dillon Nall, Ethan Sanders, Alec Vest, Shaun Westlund, Matthew Hardison, Clinton R. Paden, Duncan MacCannell
EPI_ISL_2211703	Viollier AG	Department of Biosystems Science and Engineering, ETH Zürich	Christian Beisel, Sarah Nadeau, Chaoran Chen, Ivan Topolsky, Philipp Jablonski, Lara Fuhrmann, David Dreifuss, Katharina Jahn, Rebecca Denes, Mirjam Feldkamp, Ina Nissen, Natascha Santacroce, Elodie Burcklen, Christiane Beckmann, Maurice Redondo, Olivier Kobel, Christoph Noppen, Sophie Seidel, Noemie Santamaria de Souza, Niko Beerenwinkel, Tanja Stadler
EPI_ISL_2230870	Salud Digna	Instituto Nacional de Medicina Genomica	Hidalgo-Miranda A, Cedro-Tanda A, Mendoza-Vargas A, Reyes-Grajeda JP, Abraham Campos-Romero, Moreno-Camacho José Luis, Rodriguez-Gallegos Jorge, Luna-Ruiz Marco, Gonzalez-Barrera D, Rangel-DeLeon D, Munguia-Garza P, Ramirez-Vega O, Escobar-Arrazola, M, Herrera-Montalvo LA.
EPI_ISL_2235272, EPI_ISL_2235283, EPI_ISL_2235291	Department of Microbiology, AHEPA University Hospital	Institute of Applied Biosciences, Centre for Research and Technology Hellas	Anastasia Chatzidimitriou et al.
EPI_ISL_2238524	Lighthouse Lab in Glasgow	Wellcome Sanger Institute for the COVID-19 Genomics UK (COG-UK) Consortium	Harper VanSteenhouse, Yumi Kasai, David Gray, Carol Clugston, Anna Dominiczak and Alex Alderton, Roberto Amato, Jeffrey Barrett, Sonia Goncalves, Ewan Harrison, David K. Jackson, Ian Johnston, Dominic Kwiatkowski, Cordelia Langford, John Sillitoe on behalf of the Wellcome Sanger Institute COVID-19 Surveillance Team
EPI_ISL_2239796	Northumbria University / South Tees Hospitals NHS Foundation Trust / North Cumbria Integrated Care NHS Foundation Trust / North Tees and Hartlepool NHS Foundation Trust / Newcastle Hospitals NHS Foundation Trust	COVID-19 Genomics UK (COG-UK) Consortium	Darren L Smith,Andrew Nelson,Matthew Bashton,Greg R Young,Joshua Loh,John Allan,Mohammad A Tariq,Giles S Holt,Gary Black,Wen C Yew,Lynn Dover,Paul Baker,Steve Liggett,Sarah Essex,Jane Greenaway,Debra Padgett,Clive Graham,Garren Scott,Edward Barton,Emma Swindells,Brendan Payne,Jennifer Collins,Yusri Taha,Gary Eltringham
EPI_ISL_2240466	Oxford Viromics, NDM, University of Oxford; Oxford University Hospitals; Basingstoke and North Hampshire Hospital	COVID-19 Genomics UK (COG-UK) Consortium	Tanya Golubchik, David Bonsall, George Macintyre, Amy Trebes, Mariateresa de Cesare, Catrin Moore, Alex Mobbs, Anita Justice, Robert Shaw, Monique Andersson, Timothy Peto, Emma Wise, Nathan Moore, Jessica Lynch, Nick Cortes, Matilde Mori, Stephen Kidd, David Buck, John Todd, Christophe Fraser
EPI_ISL_2241736, EPI_ISL_2241972, EPI_ISL_2242219, EPI_ISL_2242331, EPI_ISL_2242452	Aegis Sciences Corporation	Centers for Disease Control and Prevention Division of Viral Diseases, Pathogen Discovery	Dakota Howard, Dhwani Batra, Peter W. Cook, Kara Moser, Adrian Paskey, Jason Caravas, Benjamin Rambo-Martin, Shatavia Morrison, Christopher Gulvick, Scott Sammons, Yvette Unoarumhi, Darlene Wagner, Matthew Schmerer, Cyndi Clark, Patrick Campbell, Rob Case, Vikramsinha Ghorpade, Holly Houdeshell, Ola Kvalvaag, Dillon Nall, Ethan Sanders, Alec Vest, Shaun Westlund, Matthew Hardison, Clinton R. Paden, Duncan MacCannell
EPI_ISL_2249836, EPI_ISL_2250151	Laboratory Medicine	Department of Laboratory Medicine, Lin-Kou Chang Gung Memorial Hospital, Taoyuan, Taiwan	Kuo-Chien Tsao, Yu-Nong Gong, Shu-Li Yang, Yi-Chun Liu, Chung-Guei Huang, Mei-Jen Hsiao, Po-Wei Huang, Cheng-Ta Yang, Cheng-Hsun Chiu, Peng-Nien Huang, Kuo-Ming Lee, Guang-Wu Chen, Shin-Ru Shih
EPI_ISL_2258492	Department of Virology and Immunology, University of Helsinki and Helsinki University Hospital, Huslab Finland	Department of Virology, Faculty of Medicine, University of Helsinki, Helsinki, Finland	Teemu Smura, Ravi Kant, Phuoc Truong, Hussein Alburkat, Hannimari Kallio-Kokko, Jenni Virtanen, Maija Suvanto, Essi Korhonen, Sari Hannula, Harri Kangas, Hanna Liimatainen, Satu Kurkela, Hanna Jarva, Maija Lappalainen, Pekka Ellonen, Olli Vapalahti
EPI_ISL_2260334	Labor 28 MVZ GmbH	Robert Koch Institute	unknown
EPI_ISL_2268394, EPI_ISL_2268404, EPI_ISL_2268427	Quest Diagnostics Incorporated	Centers for Disease Control and Prevention Division of Viral Diseases, Pathogen Discovery	Dakota Howard, Dhwani Batra, Peter W. Cook, Kara Moser, Adrian Paskey, Jason Caravas, Benjamin Rambo-Martin, Shatavia Morrison, Christopher Gulvick, Scott Sammons, Yvette Unoarumhi, Darlene Wagner, Matthew Schmerer, S. H. Rosenthal, A. Gerasimova, R. M. Kagan, B. Anderson, M. Hua, Y. Liu, L.E. Bernstein, K.E. Livingston, A. Perez, I. A. Shlyakhter, R. V. Rolando, R. Owen, P. Tanpaiboon, F. Lacbawan, Clinton R. Paden, Duncan MacCannell
EPI_ISL_2270925	SIESP DIPARTIMENTO DI PREVENZIONE TERAMO	Istituto Zooprofilattico Sperimentale dell'Abruzzo e Molise "G. Caporale"	Lorusso A, Marcacci M, Di Domenico M, Ancora M, Curini V, Di Lollo Valeria, Mangone I, Rinaldi A, Delli Compagni E, Scialabba S, Caporale M, Di Pasquale A, Cammà C, Puglia I, Calistri P, Savini G
EPI_ISL_2272583	GA Department of Public Health	GA Department of Public Health	Stacy Reeves, Jonathan Edwards, Cynthia Dixey, Tonia Parrott, Aliyah Fields, Taylor Smith
EPI_ISL_2272976	AREA DE SALUD SAN JUAN-SAN DIEGO-CONCEPCION 2	Inciensa, Instituto Costarricense de Investigación y Enseñanza en Nutrición y Salud	Francisco Duarte, Hebleen Porras, Claudio Soto-Garita, Estela Cordero, Adriana Godínez, Melany Calderón, José Luis Vargas, Mariela Gutiérrez, Joselyn Prado & Mariel López
EPI_ISL_2272985	HOSPITAL CIUDAD NEILY	Inciensa, Instituto Costarricense de Investigación y	Francisco Duarte, Hebleen Porras, Claudio Soto-Garita, Estela Cordero, Adriana Godínez, Melany Calderón, José Luis Vargas, Mariela Gutiérrez, Joselyn

		Enseñanza en Nutrición y Salud	Prado & Raúl Zeledón-Mayorga
EPI_ISL_2277760, EPI_ISL_2277780	Florida Bureau of Public Health Laboratories	Florida Bureau of Public Health Laboratories	Sarah Schmedes, Jason Blanton
EPI_ISL_2279168	Wisconsin State Laboratory of Hygiene Communicable Disease Division	Wisconsin State Laboratory of Hygiene Communicable Disease Division	Abigail C. Shockey, Alicia J. Mooney, Erika M. Hanson, Tonya Danz, Richard Griesser, Sara Wagner, Kelsey R. Florek
EPI_ISL_2280581	Aegis Sciences Corporation	Centers for Disease Control and Prevention Division of Viral Diseases, Pathogen Discovery	Dakota Howard, Dhwani Batra, Peter W. Cook, Kara Moser, Adrian Paskey, Jason Caravas, Benjamin Rambo-Martin, Shatavia Morrison, Christopher Gulvick, Scott Sammons, Yvette Unoarumhi, Darlene Wagner, Matthew Schmerer, Cyndi Clark, Patrick Campbell, Rob Case, Vikramsinha Ghorpade, Holly Houdeshell, Ola Kvalvaag, Dillon Nall, Ethan Sanders, Alec Vest, Shaun Westlund, Matthew Hardison, Clinton R. Paden, Duncan MacCannell
EPI_ISL_2283205	Weill Cornell Medicine	New York Genome Center	Michael Zody, Andre Corvelo, Dayna M. Oschwald, Samantha Fennessey, Tom Maniatis, Melissa Cushing, Olivier Elemento, Margaret Elizabeth Ross, Chris Mason, Priya Velu, Hanna Rennert, Arryn Craney, Lars F Westblade
EPI_ISL_2283558	Infinity Biologix	Centers for Disease Control and Prevention Division of Viral Diseases, Pathogen Discovery	Dakota Howard, Dhwani Batra, Peter W. Cook, Kara Moser, Adrian Paskey, Jason Caravas, Benjamin Rambo-Martin, Shatavia Morrison, Christopher Gulvick, Scott Sammons, Yvette Unoarumhi, Darlene Wagner, Matthew Schmerer, Christian Bixby, Yihe Wang, Jonathan Schultz, Chirayu Goswami, Russ Hager, Robin Grimwood, Clinton R. Paden, Duncan MacCannell
EPI_ISL_2283696	LESP Michoacan	Instituto de Diagnostico y Referencia Epidemiologicos (INDRE)	Claudia Wong-Arambula, Abril Rodriguez-Maldonado, Vanessa Rivero-Arredondo, Ariadna Medina-Benitez, Joaquin Quiroz-Mercado, Sergio Rangel-Guerrero, Natividad Cruz-Ortiz, Tatiana Nunez-Garcia, Gisela Barrera-Badillo, Lucia Hernandez-Rivas, Irma Lopez-Martinez, Ernesto Ramirez-Gonzalez.
EPI_ISL_2293455, EPI_ISL_2293463	Labo Analyses Med	National Reference Center for Viruses of Respiratory Infections, Institut Pasteur, Paris	Marion Barbet, Sylvie Behillil, Méline Bizard, Angela Brisebarre, Camille Capel, Vincent Enouf, Louise Lefrançois, Frédéric Lemoine, Christophe Malabat, Corinne Maufrais, Etienne Simon-Lorière, Maud Vanpeene, Sylvie Van der Werf ,Philippe Miara
EPI_ISL_2295590	LESP Guanajuato	Instituto de Diagnostico y Referencia Epidemiologicos (INDRE)	Claudia Wong-Arambula, Abril Rodriguez-Maldonado, Vanessa Rivero-Arredondo, Ariadna Medina-Benitez, Joaquin Quiroz-Mercado, Sergio Rangel-Guerrero, Natividad Cruz-Ortiz, Tatiana Nunez-Garcia, Gisela Barrera-Badillo, Lucia Hernandez-Rivas, Irma Lopez-Martinez, Ernesto Ramirez-Gonzalez.
EPI_ISL_2296385	Yale Clinical Virology Lab	Grubaugh Lab - Yale School of Public Health	Joseph Fauver, Mallery Breban, Isabel Ott, Tara Alpert, Mary Petrone, Anderson Brito, Chantal Vogels, Annie Watkins, Chaney Kalinich, Jessica Rothman, Marie L. Landry, Nathan Grubaugh
EPI_ISL_2301695	Laboratory of Hygiene and Epidemiology, Department of Medicine, University of Thessaly	Greek Genome Center, Biomedical Research Foundation of the Academy of Athens (BRFAA)	Emmanouil Athanasiadis, Giannis Vatsellas, Theodoros Loupis, Katerina Zoi, Christos Hadjichristodoulou, Dimitrios Thanos
EPI_ISL_2308592	OSPEDALE CIVILE SANT'OMERO - PRONTO SOCCORSO	Istituto Zooprofilattico Sperimentale dell'Abruzzo e Molise "G. Caporale"	Lorusso A, Marcacci M, Di Domenico M, Ancora M, Curini V, Di Lollo Valeria, Mangone I, Rinaldi A, Delli Compagni E, Scialabba S, Caporale M, Di Pasquale A, Cammà C, Puglia I, Calistri P, Savini G
EPI_ISL_2319317	INDRE	Instituto Nacional de Medicina Genomica	Hidalgo-Miranda A, Mendoza-Vargas A, Reyes-Grajeda JP, Cedro-Tanda A, Gisela Barrera-Badillo, Irma Lopez-Martinez, Jose Ernesto Ramirez González, Gonzalez-Barrera D, Rangel-DeLeon D, Munguia-Garza P, Garcia-Cardenas FJ, Gonzalez-Woge MA, Herrera-Montalvo LA.
EPI_ISL_2319475, EPI_ISL_2319486, EPI_ISL_2319547, EPI_ISL_2319550	Maryland Genomics, Institute for Genome Sciences, University of Maryland School of Medicine	Maryland Genomics, Institute for Genome Sciences, University of Maryland School of Medicine	Tallon, Luke J; Sadzewicz, Lisa D; Humphrys, Mike; Ott, Sandra; Roussey, Holly; Mehta, Aditya; Vavikolanu, Kranthi; Fraser, Claire M; Ravel, Jacques
EPI_ISL_2319894	Reditus Laboratories	Reditus Laboratories	Joshua J. Geltz, Ph.D., Robert M. Sgambelluri, Ph.D., Rex Dyer, Ph.D., Cassy Philips, M.S., Alexa Eichelberger, M.S.
EPI_ISL_2320418	Helix/Illumina	Centers for Disease Control and Prevention Division of Viral Diseases, Pathogen Discovery	Dakota Howard, Dhwani Batra, Peter W. Cook, Kara Moser, Adrian Paskey, Jason Caravas, Benjamin Rambo-Martin, Shatavia Morrison, Christopher Gulvick, Scott Sammons, Yvette Unoarumhi, Darlene Wagner, Matthew Schmerer, Eileen de Feo, Jan Antico, Christine Tran, Matthew Tolentino, Shannon Wickline, Kim Gietzen, Brad Sickler, Jingtao Liu, Eric Allen, Phil Febbo, Nicole L. Washington, Simon White, Geraint Levan, Kelly Schiabor Barrett, Elizabeth Cirulli, Alexandre Bolze, Ary Ascencio, Charlotte Rivera-Garcia, Ryan Cho, Jason Nguyen, Sherry Wang, Jimmy Ramirez, Tyler Cassens, Efren Sandoval, Magnus Isaksson, William Lee, David Becker, Marc Laurent, James Lu, Clinton R. Paden, Duncan MacCannell
EPI_ISL_2331480, EPI_ISL_2331509, EPI_ISL_2331622, EPI_ISL_2331624, EPI_ISL_2331625, EPI_ISL_2331644	Johns Hopkins Hospital Department of Pathology	Johns Hopkins Hospital Department of Pathology	C. Paul Morris, Chun Huai Luo, Adannaya Amadi, Matthew Schwartz, Nicholas Gallagher, Heba H. Mostafa
EPI_ISL_2331853	Platform BIS UZA/UAntwerpen	Labo Klinische Biologie, UZA	Marie Le Mercier, Jasmine Coppens, Basil Britto Xavier, Christine Lammens, Veerle Matheeussen, Herman Goossens
EPI_ISL_2332660	Maple Grove Hospital	Minnesota Department of Health, Public Health Laboratory	Alexandra Lorentz, Jacob Garfin, Matt Plumb, and Xiong Wang
EPI_ISL_2333996	BIOMNIS EUROFINS IVRY	Department of Virology, Henri Mondor University Hospital, Assistance Publique Hôpitaux de Paris, Université Paris-Est Créteil, INSERM U955	Christophe Rodriguez, Slim Fourati, Vanessa Demontant, Guillaume Gricourt, Melissa N'Debi, Alexandre Soulier, Elisabeth Trawinski, Jean-Michel Pawlotsky
EPI_ISL_2336204	Washington State Department of Health Public Health Laboratories	Washington State Department of Health Public Health Laboratories	Drew MacKellar, Philip Dykema, Denny Russell, Joenice Gonzalez, Hannah Gray, Geoff Melly, Vanessa De Los Santos, Darren Lucas, JohnAric Peterson, Avi Singh, Rebecca Cao
EPI_ISL_2339310	Oregon State Public Health Laboratory	Oregon State Public Health Laboratory	Rafia Razzaque, Eugene Yeboah, Vanda Makris, Laura Tsaknaridis, John Fontana and Shane Sevey
EPI_ISL_2341367, EPI_ISL_2341390, EPI_ISL_2341407	The Ohio State University Applied Microbiology Services Laboratory	The Ohio State University Applied Microbiology Services Laboratory	Seth A. Faith PhD
EPI_ISL_2341501	Humboldt County Public Health Laboratory	Chan-Zuckerberg Biohub	CZB Cliahub Consortium
EPI_ISL_2341515	Contra Costa County Public Health Lab	Chan-Zuckerberg Biohub	CZB Cliahub Consortium
EPI_ISL_2341644	CA DPH Viral and Rickettsial Disease Laboratory	Chan-Zuckerberg Biohub	CZB Cliahub Consortium
EPI_ISL_2343034	University Hospitals of Geneva, Laboratory of Virology	HUG, Laboratory of Virology and the Health2030 Genome Center	Samuel Cordey, Ana Rita Goncalves, Laurent Kaiser, Lorenzo Cerutti, Henri Pegeot, Melyssa Elies, Deborah Penet, Keith Harshman, Ioannis Xenarios, Emmanouil Dermitzakis
EPI_ISL_2343466, EPI_ISL_2343697, EPI_ISL_2343792	Greek Genome Center, Biomedical Research Foundation of the Academy of Athens (BRFAA)	Greek Genome Center, Biomedical Research Foundation of the Academy of Athens (BRFAA)	Emmanouil Athanasiadis, Giannis Vatsellas, Theodoros Loupis, Katerina Zoi, Dimitrios Thanos
EPI_ISL_2346103	Oregon State Public Health Laboratory	Oregon State Public Health Laboratory	Rafia Razzaque, Eugene Yeboah, Vanda Makris, Laura Tsaknaridis, John Fontana and Shane Sevey
EPI_ISL_2346127	Emory Molecular Diagnostics Laboratory, Emory Healthcare	Piantadosi Lab, Emory Department of Pathology	Ahmed Babiker, Anne Piantadosi
EPI_ISL_2347612	Health Services Laboratories	Wellcome Sanger Institute for the COVID-19 Genomics UK (COG-UK) Consortium	Health Services Laboratories and Alex Alderton, Roberto Amato, Jeffrey Barrett, Sonia Goncalves, Ewan Harrison, David K. Jackson, Ian Johnston, Dominic Kwiatkowski, Cordelia Langford, John Sillitoe on behalf of the Wellcome Sanger Institute COVID-19 Surveillance Team
EPI_ISL_2347863	Lighthouse Laboratory Plymouth	Wellcome Sanger Institute for the COVID-19 Genomics UK (COG-UK) Consortium	Lighthouse Laboratory Plymouth and Alex Alderton, Roberto Amato, Jeffrey Barrett, Sonia Goncalves, Ewan Harrison, David K. Jackson, Ian Johnston, Dominic Kwiatkowski, Cordelia Langford, John Sillitoe on behalf of the Wellcome Sanger Institute COVID-19 Surveillance Team
EPI_ISL_2348326	Lighthouse Lab in Alderley Park	Wellcome Sanger Institute for the COVID-19 Genomics UK (COG-UK) Consortium	Jacquelyn Wynn, Mairead Hyland, The Lighthouse Lab in Alderley Park and Alex Alderton, Roberto Amato, Jeffrey Barrett, Sonia Goncalves, Ewan Harrison, David K. Jackson, Ian Johnston, Dominic Kwiatkowski, Cordelia Langford, John Sillitoe on behalf of the Wellcome Sanger Institute COVID-19 Surveillance Team
EPI_ISL_2348722, EPI_ISL_2348723	Gyncentrum	Gyncentrum	Emilia Morawiec, Pawel Czerwinski, Adam Pudelko, Aleksandra Skubis-Sikora, Magdalena Samul, Agnieszka Polak, Celina Kruszniewska-Rajs, Tomasz Wasik, Robert Wojtyczka, Jolanta Bartosiewicz- Wasik, Maria Maklasinska-Majdanik
EPI_ISL_2349657	Pandemic Response Lab - NYC	Pandemic Response Lab, R&D	Henry Lee, Michael Hammerling, Melissa Hopkins, Cybill del Castillo, Shinyoung Clair Kang, William Ward, Pradeep Bugga, Sol Rey, Dylan Law, Katharine Nelson, Haiping Hao, Jon Laurent
EPI_ISL_2350025, EPI_ISL_2350065	Salud Digna	Instituto Nacional de Medicina Genomica	Hidalgo-Miranda A, Cedro-Tanda A, Mendoza-Vargas A, Reyes-Grajeda JP, Abraham Campos-Romero, Moreno-Camacho José Luis, Rodriguez-Gallegos

			Jorge, Luna-Ruiz Marco, Gonzalez-Barrera D, Rangel-DeLeon D, Munguia-Garza P, Ramirez-Vega O, Escobar-Arrazola, M, Herrera-Montalvo LA.
EPI_ISL_2353310	Lighthouse Lab in Milton Keynes	Wellcome Sanger Institute for the COVID-19 Genomics UK (COG-UK) Consortium	The Lighthouse Lab in Milton Keynes and Alex Alderton, Roberto Amato, Jeffrey Barrett, Sonia Goncalves, Ewan Harrison, David K. Jackson, Ian Johnston, Dominic Kwiatkowski, Cordelia Langford, John Sillitoe on behalf of the Wellcome Sanger Institute COVID-19 Surveillance Team
EPI_ISL_2353315	Lighthouse Laboratory Plymouth	Wellcome Sanger Institute for the COVID-19 Genomics UK (COG-UK) Consortium	Lighthouse Laboratory Plymouth and Alex Alderton, Roberto Amato, Jeffrey Barrett, Sonia Goncalves, Ewan Harrison, David K. Jackson, Ian Johnston, Dominic Kwiatkowski, Cordelia Langford, John Sillitoe on behalf of the Wellcome Sanger Institute COVID-19 Surveillance Team
EPI_ISL_2353404	Randox Laboratories	Wellcome Sanger Institute for the COVID-19 Genomics UK (COG-UK) Consortium	Randox Laboratories and Alex Alderton, Roberto Amato, Jeffrey Barrett, Sonia Goncalves, Ewan Harrison, David K. Jackson, Ian Johnston, Dominic Kwiatkowski, Cordelia Langford, John Sillitoe on behalf of the Wellcome Sanger Institute COVID-19 Surveillance Team
EPI_ISL_2353558	Lighthouse Laboratory Plymouth	Wellcome Sanger Institute for the COVID-19 Genomics UK (COG-UK) Consortium	Lighthouse Laboratory Plymouth and Alex Alderton, Roberto Amato, Jeffrey Barrett, Sonia Goncalves, Ewan Harrison, David K. Jackson, Ian Johnston, Dominic Kwiatkowski, Cordelia Langford, John Sillitoe on behalf of the Wellcome Sanger Institute COVID-19 Surveillance Team
EPI_ISL_2355576, EPI_ISL_2355948, EPI_ISL_2356492	Northumbria University / South Tees Hospitals NHS Foundation Trust / North Cumbria Integrated Care NHS Foundation Trust / North Tees and Hartlepool NHS Foundation Trust / Newcastle Hospitals NHS Foundation Trust	COVID-19 Genomics UK (COG-UK) Consortium	Darren L Smith,Andrew Nelson,Matthew Bashton,Greg R Young,Joshua Loh,John Allan,Mohammad A Tariq,Giles S Holt,Gary Black,Wen C Yew,Lynn Dover,Paul Baker,Steve Liggett,Sarah Essex,Jane Greenaway,Debra Padgett,Clive Graham,Garren Scott,Edward Barton,Emma Swindells,Brendan Payne,Jennifer Collins,Yusri Taha,Gary Eltringham
EPI_ISL_2362010, EPI_ISL_2362022, EPI_ISL_2362032	Virginia Division of Consolidated Laboratory Services	Virginia Division of Consolidated Laboratory Services	Virginia DCLS
EPI_ISL_2362145, EPI_ISL_2362197	Virology Laboratory, Scientific Department, Army Medical Center	Virology Laboratory, Scientific Department, Army Medical Center	Silvia Fillo, Riccardo De Sanctis, Antonella Fortunato, Anella Monte, Rossella Brandi, Giulia Campoli, Marzia Cavalli, Lucia Nicosia, Anna Anselmo, Vanessa Vera Fain, Francesco Giordani, Giandomenico Cerreto, Filippo Molinari, Giancarlo Petralito, Florigio Lista, Maria Anna Spinelli, Margherita De Santis
EPI_ISL_2363530, EPI_ISL_2363549, EPI_ISL_2363551, EPI_ISL_2363552, EPI_ISL_2363571, EPI_ISL_2363574	Laboratorio Central de la Ciudad de Santa Fe	Grupo de Genómica y Bioinformática del Instituto de Investigación de la Cadena Láctea CONICET-INTA on behalf of 'Proyecto Argentino Interinstitucional de genomica de SARS-CoV-2' (PAIS Consortium)	Eberhardt, MF; Irazoqui, JM; Ojeda, G; Rompato, G; Mugna, V; Pastor, C; Amadio, AF
EPI_ISL_443600	Department of Pathology, University of Cambridge	Wellcome Sanger Institute for the COVID-19 Genomics UK (COG-UK) consortium	Luke W Meredith, M. Estée Török , Myra Hosmillo, William L. Hamilton, Martin D. Curran, Theresa Feltwell, Grant Hall, Anna Yakovleva, Fahad A Khokhar, Charlotte J. Houldcroft, Laura G Caller, Aminu S. Jahun, Sarah L. Caddy, Ian Goodfellow; and Alex Alderton, Roberto Amato, Sonia Goncalves, Ewan Harrison, David K. Jackson, Ian Johnston, Dominic Kwiatkowski, Cordelia Langford, John Sillitoe on behalf of the Wellcome Sanger Institute COVID-19 Surveillance Team (http://www.sanger.ac.uk/covid-team)
EPI_ISL_451934	Research Unit, University Hospital for Infectious Diseases "Dr. Fran Mihaljevi"	Cicin Sain lab, Helmholtz Centre for Infection Research	Zeeshan Chaudhry, Kathrin Eschke, Željka Maak Šafranko, Ivan-Christian Kurolt
EPI_ISL_477779	University of Birmingham	COVID-19 Genomics UK (COG-UK) Consortium	Institute of Microbiology, University of Birmingham: Claire McMurray, Joanne Stockton, Samuel Nicholls, Radoslaw Poplawski, Will Rowe, Josh Quick, Nicholas Loman. University of Birmingham Testing Laboratory: Celina M Whalley, Andrew Bosworth, Charlotte Poxon, Kasun Wanigasooriya, Oliver Pickles, Mike Kidd, Alex Richter, Andrew D Beggs PHE Heartlands Lab: Husam Osman, Andrew Bosworth. Queen Elizabeth Hospital: Anna Casey
EPI_ISL_500152	Liverpool Clinical Laboratories	COVID-19 Genomics UK (COG-UK) Consortium	Sam Haldenby, Anita Lucaci, Steve Paterson, Julian Hiscox, Alistair Darby, M Almsaud, A Alrezaihi, Muhannad Alruwaili, Stuart D Armstrong, Jones Benjamin, Eleanor G Bentley, Anu Chawla, Jordan J Clark, Angela Cowell, Richard Eccles, Isabel García-Dorival, Matthew Gemmell, Alessandro Gerada, PKF Gilmore, Richard Gregory, Ximeng Han, Catherine Hartley, Margaret Hughes, Miren Iturriza-Gomara, James Johnson, L Luu, Jenifer Manson, Charlotte Nelson, Elaine O'Toole, Cassie Olateju, Rebekah Penrice-Randal , Lucille Rainbow, N.P Randle, Trevor Ian Robinson, Parul Sharma, Ghada T Shawli, James P Stewart, Neil Swainston, Ecaterina Vamos, Joanne Watts, Mark Whitehead
EPI_ISL_532975	Lighthouse Lab in Glasgow	Wellcome Sanger Institute for the COVID-19 Genomics UK (COG-UK) consortium	Harper VanSteenhouse, Yumi Kasai, David Gray, Carol Clugston, Anna Dominiczak and Alex Alderton, Roberto Amato, Sonia Goncalves, Ewan Harrison, David K. Jackson, Ian Johnston, Dominic Kwiatkowski, Cordelia Langford, John Sillitoe
EPI_ISL_554361, EPI_ISL_554494	Lighthouse Lab in Alderley Park	Wellcome Sanger Institute for the COVID-19 Genomics UK (COG-UK) consortium	The Lighthouse Lab in Alderley Park and Alex Alderton, Roberto Amato, Sonia Goncalves, Ewan Harrison, David K. Jackson, Ian Johnston, Dominic Kwiatkowski, Cordelia Langford, John Sillitoe on behalf of the Wellcome Sanger Institute COVID-19 Surveillance Team
EPI_ISL_569876	Amedeo di savoia	Crosetto lab, Karolinska Institutet, SciLifeLab	Michele Simonetti, Maria Grazia Milia, Luuk Harbers, Ning Zhang, Anna Sapino, Valeria Ghisetti, Nicola Crosetto
EPI_ISL_600093, EPI_ISL_601443	Lighthouse Lab in Milton Keynes	Wellcome Sanger Institute for the COVID-19 Genomics UK (COG-UK) consortium	The Lighthouse Lab in Milton Keynes and Alex Alderton, Roberto Amato, Sonia Goncalves, Ewan Harrison, David K. Jackson, Ian Johnston, Dominic Kwiatkowski, Cordelia Langford, John Sillitoe on behalf of the Wellcome Sanger Institute COVID-19 Surveillance Team (http://www.sanger.ac.uk/covid-team)
EPI_ISL_633642	Lighthouse Lab in Glasgow	Wellcome Sanger Institute for the COVID-19 Genomics UK (COG-UK) consortium	Harper VanSteenhouse, Yumi Kasai, David Gray, Carol Clugston, Anna Dominiczak and Alex Alderton, Roberto Amato, Sonia Goncalves, Ewan Harrison, David K. Jackson, Ian Johnston, Dominic Kwiatkowski, Cordelia Langford, John Sillitoe on behalf of the Wellcome Sanger Institute COVID-19 Surveillance Team
EPI_ISL_634338	Lighthouse Lab in Milton Keynes	Wellcome Sanger Institute for the COVID-19 Genomics UK (COG-UK) consortium	The Lighthouse Lab in Milton Keynes and Alex Alderton, Roberto Amato, Sonia Goncalves, Ewan Harrison, David K. Jackson, Ian Johnston, Dominic Kwiatkowski, Cordelia Langford, John Sillitoe on behalf of the Wellcome Sanger Institute COVID-19 Surveillance Team
EPI_ISL_655523	Lighthouse Lab in Alderley Park	Wellcome Sanger Institute for the COVID-19 Genomics UK (COG-UK) Consortium	Jacquelyn Wynn, Mairead Hyland, The Lighthouse Lab in Alderley Park and Alex Alderton, Roberto Amato, Sonia Goncalves, Ewan Harrison, David K. Jackson, Ian Johnston, Dominic Kwiatkowski, Cordelia Langford, John Sillitoe on behalf of the Wellcome Sanger Institute COVID-19 Surveillance Team
EPI_ISL_658409	Lighthouse Lab in Glasgow	Wellcome Sanger Institute for the COVID-19 Genomics UK (COG-UK) Consortium	Harper VanSteenhouse, Yumi Kasai, David Gray, Carol Clugston, Anna Dominiczak and Alex Alderton, Roberto Amato, Sonia Goncalves, Ewan Harrison, David K. Jackson, Ian Johnston, Dominic Kwiatkowski, Cordelia Langford, John Sillitoe on behalf of the Wellcome Sanger Institute COVID-19 Surveillance Team
EPI_ISL_659106	Lighthouse Lab in Cambridge	Wellcome Sanger Institute for the COVID-19 Genomics UK (COG-UK) Consortium	Rob Howes, The Lighthouse Lab in Cambridge and Alex Alderton, Roberto Amato, Sonia Goncalves, Ewan Harrison, David K. Jackson, Ian Johnston, Dominic Kwiatkowski, Cordelia Langford, John Sillitoe on behalf of the Wellcome Sanger Institute COVID-19 Surveillance Team
EPI_ISL_659955	Lighthouse Lab in Glasgow	Wellcome Sanger Institute for the COVID-19 Genomics UK (COG-UK) Consortium	Harper VanSteenhouse, Yumi Kasai, David Gray, Carol Clugston, Anna Dominiczak and Alex Alderton, Roberto Amato, Sonia Goncalves, Ewan Harrison, David K. Jackson, Ian Johnston, Dominic Kwiatkowski, Cordelia Langford, John Sillitoe on behalf of the Wellcome Sanger Institute COVID-19 Surveillance Team
EPI_ISL_673868	Lighthouse Lab in Cambridge	Wellcome Sanger Institute for the COVID-19 Genomics UK (COG-UK) Consortium	Rob Howes, The Lighthouse Lab in Cambridge and Alex Alderton, Roberto Amato, Sonia Goncalves, Ewan Harrison, David K. Jackson, Ian Johnston, Dominic Kwiatkowski, Cordelia Langford, John Sillitoe on behalf of the Wellcome Sanger Institute COVID-19 Surveillance Team
EPI_ISL_674180	Lighthouse Lab in Milton Keynes	Wellcome Sanger Institute for the COVID-19 Genomics UK (COG-UK) Consortium	The Lighthouse Lab in Milton Keynes and Alex Alderton, Roberto Amato, Sonia Goncalves, Ewan Harrison, David K. Jackson, Ian Johnston, Dominic Kwiatkowski, Cordelia Langford, John Sillitoe on behalf of the Wellcome Sanger Institute COVID-19 Surveillance Team
EPI_ISL_683466	Respiratory Virus Unit, Microbiology Services Colindale, Public Health England	COVID-19 Genomics UK (COG-UK) Consortium	PHE Covid Sequencing Team
EPI_ISL_702193, EPI_ISL_719083, EPI_ISL_719450	Lighthouse Lab in Cambridge	Wellcome Sanger Institute for the COVID-19 Genomics UK (COG-UK) Consortium	Rob Howes, The Lighthouse Lab in Cambridge and Alex Alderton, Roberto Amato, Sonia Goncalves, Ewan Harrison, David K. Jackson, Ian Johnston, Dominic Kwiatkowski, Cordelia Langford, John Sillitoe on behalf of the Wellcome Sanger Institute COVID-19 Surveillance Team
EPI_ISL_720147, EPI_ISL_735825	Lighthouse Lab in Milton Keynes	Wellcome Sanger Institute for the COVID-19 Genomics UK (COG-UK) Consortium	The Lighthouse Lab in Milton Keynes and Alex Alderton, Roberto Amato, Sonia Goncalves, Ewan Harrison, David K. Jackson, Ian Johnston, Dominic Kwiatkowski, Cordelia Langford, John Sillitoe on behalf of the Wellcome Sanger Institute COVID-19 Surveillance Team
EPI_ISL_738108	Instituto Nacional de Saude (INSA)	Instituto Nacional de Saude (INSA)	Borges et al
EPI_ISL_756739	Lighthouse Lab in Milton Keynes	Wellcome Sanger Institute for the COVID-19 Genomics UK (COG-UK) Consortium	The Lighthouse Lab in Milton Keynes and Alex Alderton, Roberto Amato, Sonia Goncalves, Ewan Harrison, David K. Jackson, Ian Johnston, Dominic Kwiatkowski, Cordelia Langford, John Sillitoe on behalf of the Wellcome Sanger Institute COVID-19 Surveillance Team
EPI_ISL_760287	Lighthouse Lab in Glasgow	Wellcome Sanger Institute for the COVID-19 Genomics UK	Harper VanSteenhouse, Yumi Kasai, David Gray, Carol Clugston, Anna Dominiczak and Alex Alderton, Roberto Amato, Sonia Goncalves, Ewan Harrison,

		(COG-UK) Consortium	David K. Jackson, Ian Johnston, Dominic Kwiatkowski, Cordelia Langford, John Sillitoe on behalf of the Wellcome Sanger Institute COVID-19 Surveillance Team
EPI_ISL_783232	Lighthouse Lab in Milton Keynes	Wellcome Sanger Institute for the COVID-19 Genomics UK (COG-UK) Consortium	The Lighthouse Lab in Milton Keynes and Alex Alderton, Roberto Amato, Sonia Goncalves, Ewan Harrison, David K. Jackson, Ian Johnston, Dominic Kwiatkowski, Cordelia Langford, John Sillitoe on behalf of the Wellcome Sanger Institute COVID-19 Surveillance Team
EPI_ISL_783422	Lighthouse Lab in Cambridge	Wellcome Sanger Institute for the COVID-19 Genomics UK (COG-UK) Consortium	Rob Howes, The Lighthouse Lab in Cambridge and Alex Alderton, Roberto Amato, Sonia Goncalves, Ewan Harrison, David K. Jackson, Ian Johnston, Dominic Kwiatkowski, Cordelia Langford, John Sillitoe on behalf of the Wellcome Sanger Institute COVID-19 Surveillance Team
EPI_ISL_799577	Lighthouse Lab in Milton Keynes	Wellcome Sanger Institute for the COVID-19 Genomics UK (COG-UK) Consortium	The Lighthouse Lab in Milton Keynes and Alex Alderton, Roberto Amato, Sonia Goncalves, Ewan Harrison, David K. Jackson, Ian Johnston, Dominic Kwiatkowski, Cordelia Langford, John Sillitoe on behalf of the Wellcome Sanger Institute COVID-19 Surveillance Team
EPI_ISL_802384	MSHS Clinical Microbiology Laboratories	MSHS Pathogen Surveillance Program	Ana S. Gonzalez-Reiche, Hala Alshammary, Mitchell J. Sullivan, Brianne Ciferri, Ajay Obla, Angela Amoako, Mahmoud Awawda, Elena Hirsch, Ashley S. Salimbangon, Levy Sominsky, Katherine Beach, Kayla Russo, Charles Gleason, Shelcie Fabre, Giulio Kleiner, Zenab Khan, Bremy Alburquerque, Adriana van de Guchte, Komal Srivastava, Matthew M. Hernandez, Jayeeta Dutta, Denise Jurczyszak, Emily Ferreri, Rachel Chernet, Nancy Francoeur, Betsaida Salom Melo, Irina Oussenko, Gintaras Deikus, Juan Soto, Shwetha Hara Sridhar, Ying-Chih Wang, Kathryn Twyman, Andrew Kasarskis, Deena R. Altman, Robert Sebra, Adolfo García-Sastre, Marta Luksza, Gopi Patel, Sarah Schaefer, Melissa Gitman, Michael D. Nowak, Alberto Paniz-Mondolfi, Emilia Mia Sordillo, Viviana Simon, Harm van Bakel
EPI_ISL_804972	Hospital Comarcal de Melilla	Instituto de Salud Carlos III	Iglesias-Caballero, M. Molinero Calamita, M. González-Esguevillas, M. Camarero, S. Pozo, F. Casas, I. Jiménez, P. Jiménez, M. Zaballos, A. Monzón, S. Varona, S. Juliá, M. Cuesta, I, J. López
EPI_ISL_822254	Lighthouse Lab in Alderley Park	Wellcome Sanger Institute for the COVID-19 Genomics UK (COG-UK) Consortium	Jacquelyn Wynn, Mairead Hyland, The Lighthouse Lab in Alderley Park and Alex Alderton, Roberto Amato, Sonia Goncalves, Ewan Harrison, David K. Jackson, Ian Johnston, Dominic Kwiatkowski, Cordelia Langford, John Sillitoe on behalf of the Wellcome Sanger Institute COVID-19 Surveillance Team
EPI_ISL_835147	Lighthouse Lab in Glasgow	Wellcome Sanger Institute for the COVID-19 Genomics UK (COG-UK) Consortium	Harper VanSteenhouse, Yumi Kasai, David Gray, Carol Clugston, Anna Dominiczak and Alex Alderton, Roberto Amato, Sonia Goncalves, Ewan Harrison, David K. Jackson, Ian Johnston, Dominic Kwiatkowski, Cordelia Langford, John Sillitoe on behalf of the Wellcome Sanger Institute COVID-19 Surveillance Team
EPI_ISL_835999	Lighthouse Lab in Milton Keynes	Wellcome Sanger Institute for the COVID-19 Genomics UK (COG-UK) Consortium	The Lighthouse Lab in Milton Keynes and Alex Alderton, Roberto Amato, Sonia Goncalves, Ewan Harrison, David K. Jackson, Ian Johnston, Dominic Kwiatkowski, Cordelia Langford, John Sillitoe on behalf of the Wellcome Sanger Institute COVID-19 Surveillance Team
EPI_ISL_838018	Department of Pathology, University of Cambridge	COVID-19 Genomics UK (COG-UK) Consortium	Aminu S. Jahun, Yasmin Chaudhry, Grant Hall, Iliana Georgana, Myra Hosmillo, Martin D. Curran, Malte Pinckert, Surendra Parmar, Ian Goodfellow
EPI_ISL_840744	Originating lab: Wales Specialist Virology Centre Sequencing lab: Pathogen Genomics Unit	Public Health Wales Microbiology Cardiff Wales Specialist Virology Centre	Catherine Moore, Johnathan Evans, Laura Gifford, Malorie Perry, Simon Cottrell, Angela Marchbank, Alec Birchley, Alexander Adams, Amy Gaskin, Bree Gatica-Wilcox, Jason Coombes, Joel Southgate, Lauren Gilbert, Lee Graham, Nicole Pacchiarini, Sara Kumziene-Summerhayes, Sarah Taylor, Sophie Jones, Sara Rey, Matthew Bull, Joanne Watkins, Sally Corden, Tom Connor
EPI_ISL_842652	Laboratorio de Biología Molecular Hospital Pedro de Elizalde	Grupo de Genómica y Bioinformática del Instituto de Investigación de la Cadena Láctea CONICET-INTA on behalf of 'Proyecto Argentino Interinstitucional de genomica de SARS-CoV-2' (PAIS Consortium)	Amadio, AF, Eberhardt, MF; Irazoqui, M; Indart, J; Rocovich, J; Montoto Piazza, L; Wenk, G; Martín, ME; Sanchez, MF; Marchetti, P; Morandi, F; Sueiro, ML; Claps, A; Bressan, L; Torres, FJ; Chamorro, J; Gondolessi, J; Gómez, ML; Diaz, B; Rosales, D; Alegre, F; Zamora, N; Osaba, E; Paez, E; Lorenzo, F; Torres, C; Aulicino, P; König, G; Alexay, S; Natale, M; Valinotto, L; Lusso, S; Goya, S; Nabaes Jodar, MS; Viegas, M.
EPI_ISL_843045, EPI_ISL_843060	Barts Health NHS Trust	COVID-19 Genomics UK (COG-UK) Consortium	CUTINO-MOGUEL, Maria-Teresa; HARRINGTON, David; OWOYEMI, Dola; SHYLINI, Raghavendran; BROAD, Claire; KELE, Beatrix
EPI_ISL_863793	Lighthouse Lab in Milton Keynes	Wellcome Sanger Institute for the COVID-19 Genomics UK (COG-UK) Consortium	The Lighthouse Lab in Milton Keynes and Alex Alderton, Roberto Amato, Sonia Goncalves, Ewan Harrison, David K. Jackson, Ian Johnston, Dominic Kwiatkowski, Cordelia Langford, John Sillitoe on behalf of the Wellcome Sanger Institute COVID-19 Surveillance Team
EPI_ISL_924084	Virology Department, Sheffield Teaching Hospitals NHS Foundation Trust/Department of Infection, Immunity and Cardiovascular Disease, The Medical School, University of Sheffield	COVID-19 Genomics UK (COG-UK) Consortium	Thushan de Silva, Matthew Parker, Nikki Smith, Adri Angyal, Rebecca Brown, Luke Green, Rachel Tucker, Paul Parsons, Danielle Groves, Katie Johnson, Laura Carrilero, Alex Keeley, Dave Partridge, Matthew Wyles, Benjamin Lindsey, Mehmet Yavuz, Mohammad Raza, Cariad Evans
EPI_ISL_977235	ULSS 5 Polesana	Istituto Zooprofilattico Sperimentale delle Venezie	Adelaide Milani, Alessia Schivo, Annalisa Salviato, Erika Giorgia Quaranta, Ambra Pastori, Bianca Zecchin, Alice Fusaro, Isabella Monne, Calogero Terregino, Antonia Ricci
EPI_ISL_981967	Microbiology Service, Hospital Universitario Clinico San Cecilio, Granada	Microbiology Service, Hospital Universitario Clinico San Cecilio, Granada	Adolfo de Salazar, Natalia Chueca, Laura Viñuela, Ana Fuentes, Federico García
EPI_ISL_997769	University College London, Great Ormond Street Hospital for Children NHS Foundation Trust, Imperial College Healthcare NHS Trust	COVID-19 Genomics UK (COG-UK) Consortium	Sergi Castellano, Rachel Williams, Mark Kristiansen, Paola Resende Silva, Sunando Roy, Tony Brooks, Helena Tutill, Paola Niola, Patricia Dyal, Charlotte Williams, Leysa Forrest, Yasmin Panchbhaya, Jacqueline Findlay, Samuel Weeks, Julianne Brown, Kathryn Harris, Paul Randell, James Price, Alison Holmes, Judith Breuer

Table S5

We gratefully acknowledge the following Authors from the Originating laboratories responsible for obtaining the specimens, as well as the Submitting laboratories where the genome data were generated and shared via GISAID, on which this research is based.

All Submitters of data may be contacted directly via www.gisaid.org

Authors are sorted alphabetically.

Accession ID	Originating Laboratory	Submitting Laboratory	Authors
EPI_ISL_1014675	Department of Infectious Diseases, Istituto Superiore di Sanità, Rome, Italy; Università degli Studi di Perugia, Perugia, Italy	Istituto Superiore di Sanità (ISS)	Paola Stefanelli, Alessandra Lo Presti, Angela Di Martino, Stefano Fiore, Antonella Mencacci, Barbara Camilloni, Manuela Marra, Maria Carollo, Marco Crescenzi, Luca De Sabato
EPI_ISL_1036239	Center of Advanced Studies and Technology, Molecular Genetics Laboratory	Center of Advanced Studies and Technology, Molecular Genetics Laboratory	Ferrante Rossella, Mandatori Domitilla, De Fabritiis Simone, Damiani Verena, Anaclerio Federico
EPI_ISL_1039708	Instituto Adolfo Lutz Central	Instituto Adolfo Lutz, Interdiciplinary Procedures Center, Strategic Laboratory	Claudio Tavares Sacchi, Claudia Regina Gonçalves, Erica Valessa Ramos Gomes, Karoline Rodrigues Campos
EPI_ISL_1041509	LACEN do Estado de Goias	Instituto Adolfo Lutz, Interdiciplinary Procedures Center, Strategic Laboratory	Claudio Tavares Sacchi, Claudia Regina Gonçalves, Erica Valessa Ramos Gomes, Karoline Rodrigues Campos
EPI_ISL_1060877, EPI_ISL_1060879, EPI_ISL_1060898	CDL Laboratorio Santos e Vidal LTDA.	Instituto de Medicina Tropical de Sao Paulo	Brazil-UK Centre for Arbovirus Discovery Diagnosis Genomics and Epidemiology (CADDE) Genomic Network - Instituto de Medicina Tropical
EPI_ISL_1063910	Ospedale San Filippo Neri	INMI Lazzaro Spallanzani IRCCS	M Rueca, O Butera, F Messina, CEM Gruber, B Bartolini, E Giombini, M Melandri, ML Schiavone, A Di Caro, MR Capobianchi
EPI_ISL_1064736	CDL Laboratorio Santos e Vidal LTDA.	Instituto de Medicina Tropical de Sao Paulo	Brazil-UK Centre for Arbovirus Discovery Diagnosis Genomics and Epidemiology (CADDE) Genomic Network - Instituto de Medicina Tropical
EPI_ISL_1068114, EPI_ISL_1068197, EPI_ISL_1068221, EPI_ISL_1068226, EPI_ISL_1068256, EPI_ISL_1068267, EPI_ISL_1068268	Laboratorio de Ecologia de Doencas Transmissiveis na Amazonia, Instituto Leonidas e Maria Deane - Fiocruz Amazonia	Laboratorio de Ecologia de Doencas Transmissiveis na Amazonia, Instituto Leonidas e Maria Deane - Fiocruz Amazonia	Valdinete Nascimento, Victor Souza, André Corado, Fernanda Nascimento, George Silva, Ágatha Costa, Debora Duarte, Karina Pessoa, Matilde Mejía, Luciana Gonçalves, Maria Júlia Brandão, Michele Jesus, Felipe Naveca on behalf of the Fiocruz COVID-19 Genomic Surveillance Network
EPI_ISL_1073034	ASL Napoli 1 Centro	AMES Centro Polidiagnostico Strumentale S.r.l.	Giovanni Savarese, Raffaella Ruggiero, Eloisa Evangelista, Antonella Di Carlo, Luisa Circelli, Luigi D'Amore, Roberto Sirica, Antonio Fico
EPI_ISL_1086044	IAL Regional de Bauru	Instituto Adolfo Lutz, Interdiciplinary Procedures Center, Strategic Laboratory	Claudio Tavares Sacchi, Claudia Regina Gonçalves, Erica Valessa Ramos Gomes, Karoline Rodrigues Campos, Caio Vinicius Dias Lopes
EPI_ISL_1111143, EPI_ISL_1111469, EPI_ISL_1111472	Laboratorio de Referencia Nacional de Virus Respiratorio. Instituto Nacional de Salud Perú	Laboratorio de Referencia Nacional de Enteropatógenos. Instituto Nacional de Salud del Perú	Ronnie Gavilan Chavez, Junior Caro Castro, Willi Quino Sifuentes, Veronica Hurtado Vela, Iris Silva Molina, Fiorella Orellana Peralta
EPI_ISL_1121316	LACEN do Rio Grande do Sul	Instituto Adolfo Lutz, Interdiciplinary Procedures Center, Strategic Laboratory	Claudio Tavares Sacchi, Claudia Regina Gonçalves, Erica Valessa Ramos Gomes, Karoline Rodrigues Campos, Caio Vinicius Dias Lopes
EPI_ISL_1123373	Grupo Tecnico de Vigilancia Sanitaria e Epidemiologica	Instituto Adolfo Lutz, Interdiciplinary Procedures Center, Strategic Laboratory	Claudio Tavares Sacchi, Claudia Regina Gonçalves, Erica Valessa Ramos Gomes, Karoline Rodrigues Campos, Caio Vinicius Dias Lopes
EPI_ISL_1133124	LABCOVID_HCPA	LABRESIS_HCPA	Martins AF, Wink PL, Volpato F, Rosset C, de Paris F, Monteiro F, Barth AL
EPI_ISL_1156521	LabKom - Labor Augsburg MVZ GmbH	Robert Koch Institute	unknown
EPI_ISL_1157575	SYNLAB MVZ Weiden	Robert Koch Institute	unknown
EPI_ISL_1157733	Synlab MVZ Augsburg	Robert Koch Institute	unknown
EPI_ISL_1163690, EPI_ISL_1163691	UOC Microbiologia e Virologia, Azienda Ospedaliera Universitaria Senese, Siena, Italy	Dipartimento di Biotecnologie Mediche	Maria Grazia Cusi, David Pinzauti, Claudia Gandolfo, Gabriele Anichini, Gianni Pozzi, Gianni Gori Savellini, Francesco Santoro
EPI_ISL_1164972	LACEN - Laboratório Central de Saúde Pública do Pará	Evandro Chagas Institute	Santos, M.C.; Silva, A.M.; Junior, W.D.C.; Barbagelata, L.S.; Ferreira, J.A.; Sousa, E.M.A.; da Silva, P.S.; Pinheiro, K.C.; L.C.; Sousa Junior, E.C.
EPI_ISL_1164980	LACEN - Laboratório Central de Saúde Pública do Ceará	Evandro Chagas Institute	Santos, M.C.; Silva, A.M.; Junior, W.D.C.; Barbagelata, L.S.; Ferreira, J.A.; Sousa, E.M.A.; da Silva, P.S.; Pinheiro, K.C.; L.C.; Sousa Junior, E.C.
EPI_ISL_1166165	Università degli Studi di Perugia	Istituto Zooprofilattico Sperimentale dell'Abruzzo e Molise "G. Caporale"	Mencacci A, Camilloni B, Lorusso A, Marcacci M, Di Domenico M, Ancora M, Curini V, Mangone I, Rinaldi A, Scialabba S, Di Pasquale A, Cammà C, Puglia I, Calistri P, Savini G
EPI_ISL_1169197	ASL Napoli 1 Centro	AMES Centro Polidiagnostico Strumentale S.r.l.	"Giovanni Savarese, Raffaella Ruggiero, Eloisa Evangelista, Antonella Di Carlo, Luisa Circelli, Luigi D'Amore, Nadia Petrillo, Monica Ianniello, Roberto Sirica,Maurizio D'Amora, Antonio Fico"
EPI_ISL_1213196	IMT-UFRN/RN	Bioinformatics Laboratory / LNCC	Alessandra P Lamarca, Luiz G P de Almeida, Ronaldo da Silva Francisco Jr, Lucymara Fassarella Agnez Lima, Kátia Castanho Scortecci, Vinícius Pietta Perez, Otavio J. Brustolini, Eduardo Sérgio Soares Sousa, Danielle Angst Secco, Angela Maria Guimarães Santos, George Rego Albuquerque, Ana Paula Melo Mariano, Bianca Mendes Maciel, Alexandra L Gerber, Ana Paula de C Guimarães, Paulo Ricardo Nascimento, Francisco Paulo Freire Neto, Sandra Rocha Gadelha, Luís Cristóvão Porto, Eloiza Helena Campana, Selma Maria Bezerra Jeronimo, Ana Tereza R Vasconcelos
EPI_ISL_1213401	LAFEM/UESC	Bioinformatics Laboratory / LNCC	Alessandra P Lamarca, Luiz G P de Almeida, Ronaldo da Silva Francisco Jr, Lucymara Fassarella Agnez Lima, Kátia Castanho Scortecci, Vinícius Pietta Perez, Otavio J. Brustolini, Eduardo Sérgio Soares Sousa, Danielle Angst Secco, Angela Maria Guimarães Santos, George Rego Albuquerque, Ana Paula Melo Mariano, Bianca Mendes Maciel, Alexandra L Gerber, Ana Paula de C Guimarães, Paulo Ricardo Nascimento, Francisco Paulo Freire Neto, Sandra Rocha Gadelha, Luís Cristóvão Porto, Eloiza Helena Campana, Selma Maria Bezerra Jeronimo, Ana Tereza R Vasconcelos
EPI_ISL_1219132	Laboratorio Central de Saude Publica do Estado do Parana (LACEN-PR)	Laboratory of Respiratory Viruses and Measles, Oswaldo Cruz Institute, FIOCRUZ	Paola Resende, Luciana Appolinario, Fernando Motta, Anna Carolina Paixao, Ana Carolina Mendonca, Alice Sampaio Rocha, Renata Serrano Lopes, Maria do Carmo Debur, Irina Nastassja Riediger, Marilda Siqueira on behalf of the Fiocruz COVID-19 Genomic Surveillance Network
EPI_ISL_1261122, EPI_ISL_1261123	Laboratorio de Ecologia de Doencas Transmissiveis na Amazonia, Instituto Leonidas e Maria Deane - Fiocruz Amazonia	Laboratorio de Ecologia de Doencas Transmissiveis na Amazonia, Instituto Leonidas e Maria Deane - Fiocruz Amazonia	Valdinete Nascimento, Victor Souza, André Corado, Fernanda Nascimento, George Silva, Ágatha Costa, Debora Duarte, Karina Pessoa, Matilde Mejía, Luciana Gonçalves, Maria Júlia Brandão, Michele Jesus, Felipe Naveca
EPI_ISL_1261690	LACEN - Laboratório Central de Saúde Pública do Amazonas	Evandro Chagas Institute	Santos, M.C.; Silva, A.M.; Junior, W.D.C.; Barbagelata, L.S.; Ferreira, J.A.; Sousa, E.M.A.; da Silva, P.S.; Pinheiro, K.C.; L.C.; Sousa Junior, E.C.
EPI_ISL_1286876	Synlab MVZ Augsburg	Robert Koch Institute	unknown
EPI_ISL_1289956	Laboratory of Virology, Ribeirão Preto General Hospital, Ribeirão Preto Medical School, University of São Paulo	Laboratory of Oncology, Blood Center of Ribeirão Preto, Ribeirão Preto School of Medicine, University of São Paulo	CAMPOS, MR; SANTOS, A.L.P.; YAMAMOTO, A.Y.; COLLI, L.M.; FONSECA, B.A.L.; BELLISSIMO-RODRIGUES, F.
EPI_ISL_1293054	LACEN de Rondonia	Instituto Adolfo Lutz, Interdiciplinary Procedures Center, Strategic Laboratory	Claudio Tavares Sacchi, Claudia Regina Gonçalves, Erica Valessa Ramos Gomes, Karoline Rodrigues Campos, Caio Vinicius Dias Lopes
EPI_ISL_1303549	UPA Vila Santa Catarina	Instituto Adolfo Lutz, Interdiciplinary Procedures Center, Strategic Laboratory	Claudio Tavares Sacchi, Claudia Regina Gonçalves, Erica Valessa Ramos Gomes, Karoline Rodrigues Campos, Caio Vinicius Dias Lopes
EPI_ISL_1323769	National Virus Reference Laboratory	National Virus Reference Laboratory	Zoe Yandle, Charlene Bennett, Gabriel Gonzalez, Michael Carr, Jonathan Dean, Cillian F De Gascun

EPI_ISL_1381059	IAL Regional de Santo Andre	Instituto Adolfo Lutz, Interdiciplinary Procedures Center, Strategic Laboratory	Claudio Tavares Sacchi, Claudia Regina Gonçalves, Erica Valessa Ramos Gomes, Karoline Rodrigues Campos, Caio Vinicius Dias Lopes
EPI_ISL_1443197	Hospital Aliança	Hospital São Rafael - IDOR	Isadora Cristina de Siqueira, Aquiles Assunção Camelier, Elves A.P. Maciel, Margarida Celia L. C. Neves, Carolina Kymie Vasques Nonaka, Karoline Almeida Félix de Sousa, Victor Costa Araujo, Yasmin Santos Freitas Macêdo, Aurea Angelica Paste, Bruno Solano de Freitas Souza, Tiago Gräf
EPI_ISL_1445072	USF JARDIM SAO DIMAS	Instituto Butantan / Mendelics	Dimas Tadeu Covas, Sandra Coccuzzo Sampaio, Maria Carolina Elias, José Salvatore Leister Patané, Vincent Louis Viala, Antonio Jorge Martins, Ricardo Haddad, Claudia Renata dos Santos Barros, Elaine Cristina Marqueze, Raul Machado Neto, Debora Botequio Moretti, Bibiana Santos, João Paulo Kitajima, Erika Freitas, David Schlesinger, Simone Kashima, Evandra Strazza Rodrigues, Svetoslav Nanev Slavov, Elaine Vieira dos Santos, Rafael dos Santos Bezerra, Luiz Carlos Junior de Alcantara, Marta Giovanetti, Vagner Fonseca, Flavia Aburjaile, Rodrigo Tocantins Calado.
EPI_ISL_1445120	UNIDADE DE PRONTO ATENDIMENTO UPA DRA ANA OLIVIA BENTIVOGLIO	Instituto Butantan / Mendelics	Dimas Tadeu Covas, Sandra Coccuzzo Sampaio, Maria Carolina Elias, José Salvatore Leister Patané, Vincent Louis Viala, Antonio Jorge Martins, Ricardo Haddad, Claudia Renata dos Santos Barros, Elaine Cristina Marqueze, Raul Machado Neto, Debora Botequio Moretti, Bibiana Santos, João Paulo Kitajima, Erika Freitas, David Schlesinger, Simone Kashima, Evandra Strazza Rodrigues, Svetoslav Nanev Slavov, Elaine Vieira dos Santos, Rafael dos Santos Bezerra, Luiz Carlos Junior de Alcantara, Marta Giovanetti, Vagner Fonseca, Flavia Aburjaile, Rodrigo Tocantins Calado.
EPI_ISL_1445142	CS II EGIDIO BRUNHARA MORRO AGUDO	Instituto Butantan / Mendelics	Dimas Tadeu Covas, Sandra Coccuzzo Sampaio, Maria Carolina Elias, José Salvatore Leister Patané, Vincent Louis Viala, Antonio Jorge Martins, Ricardo Haddad, Claudia Renata dos Santos Barros, Elaine Cristina Marqueze, Raul Machado Neto, Debora Botequio Moretti, Bibiana Santos, João Paulo Kitajima, Erika Freitas, David Schlesinger, Simone Kashima, Evandra Strazza Rodrigues, Svetoslav Nanev Slavov, Elaine Vieira dos Santos, Rafael dos Santos Bezerra, Luiz Carlos Junior de Alcantara, Marta Giovanetti, Vagner Fonseca, Flavia Aburjaile, Rodrigo Tocantins Calado.
EPI_ISL_1445194	PRONTO ATENDIMENTO SAO JOSE	Instituto Butantan / Mendelics	Dimas Tadeu Covas, Sandra Coccuzzo Sampaio, Maria Carolina Elias, José Salvatore Leister Patané, Vincent Louis Viala, Antonio Jorge Martins, Ricardo Haddad, Claudia Renata dos Santos Barros, Elaine Cristina Marqueze, Raul Machado Neto, Debora Botequio Moretti, Bibiana Santos, João Paulo Kitajima, Erika Freitas, David Schlesinger, Simone Kashima, Evandra Strazza Rodrigues, Svetoslav Nanev Slavov, Elaine Vieira dos Santos, Rafael dos Santos Bezerra, Luiz Carlos Junior de Alcantara, Marta Giovanetti, Vagner Fonseca, Flavia Aburjaile, Rodrigo Tocantins Calado.
EPI_ISL_1445223	UBS HELENA MARREY	Instituto Butantan / Mendelics	Dimas Tadeu Covas, Sandra Coccuzzo Sampaio, Maria Carolina Elias, José Salvatore Leister Patané, Vincent Louis Viala, Antonio Jorge Martins, Ricardo Haddad, Claudia Renata dos Santos Barros, Elaine Cristina Marqueze, Raul Machado Neto, Debora Botequio Moretti, Bibiana Santos, João Paulo Kitajima, Erika Freitas, David Schlesinger, Simone Kashima, Evandra Strazza Rodrigues, Svetoslav Nanev Slavov, Elaine Vieira dos Santos, Rafael dos Santos Bezerra, Luiz Carlos Junior de Alcantara, Marta Giovanetti, Vagner Fonseca, Flavia Aburjaile, Rodrigo Tocantins Calado.
EPI_ISL_1445236, EPI_ISL_1445265	VIGILANCIA EPIDEMIOLOGICA	Instituto Butantan / Mendelics	Dimas Tadeu Covas, Sandra Coccuzzo Sampaio, Maria Carolina Elias, José Salvatore Leister Patané, Vincent Louis Viala, Antonio Jorge Martins, Ricardo Haddad, Claudia Renata dos Santos Barros, Elaine Cristina Marqueze, Raul Machado Neto, Debora Botequio Moretti, Bibiana Santos, João Paulo Kitajima, Erika Freitas, David Schlesinger, Simone Kashima, Evandra Strazza Rodrigues, Svetoslav Nanev Slavov, Elaine Vieira dos Santos, Rafael dos Santos Bezerra, Luiz Carlos Junior de Alcantara, Marta Giovanetti, Vagner Fonseca, Flavia Aburjaile, Rodrigo Tocantins Calado.
EPI_ISL_1456646	Dutch COVID-19 response team	National Institute for Public Health and the Environment (RIVM)	Adam Meijer, Harry Vennema, Dirk Eggink, Jeroen Cremer, Sharon van den Brink, Bas van der Veer, AnneMarie van den Brandt, Lisa Wijsman, Kim Freriks, Ryanne Jaarsma, Euníce Then, Jolienke Hardeman, Lynn Aarts, Sanne Bos, Melissa van Tuil, Robert Kohl, Linda van de Nes, Sjoerd Kuiling, James Groot, Florian Zwagemaker, Dennis Schmitz, Annelies Kroneman, Karim Hajji, Chantal Reusken, on behalf of the national COVID-19 response team
EPI_ISL_1464628, EPI_ISL_1464659	Laboratório de Virologia - UNIFESP	Laboratory of Respiratory Viruses and Measles, Oswaldo Cruz Institute, FIOCRUZ	Paola Resende, Nancy Belei, Luciana Appolinario, Fernando Motta, Anna Carolina Paixao, Ana Carolina Mendonca, Alice Sampaio Rocha, Renata Serrano Lopes, Marilda Siqueira on behalf of the Fiocruz COVID-19 Genomic Surveillance Network
EPI_ISL_1465189	Laboratorio Central de Saude Publica do Estado do Maranhao (LACEN-MA)	Laboratory of Respiratory Viruses and Measles, Oswaldo Cruz Institute, FIOCRUZ	Paola Resende, Luciana Appolinario, Fernando Motta, Anna Carolina Paixao, Ana Carolina Mendonca, Alice Sampaio Rocha, Renata Serrano Lopes, Lidio Gonçalves Lima Neto, Marilda Siqueira on behalf of the Fiocruz COVID-19 Genomic Surveillance Network
EPI_ISL_1468414	LACEN do Estado de Goias	Instituto Adolfo Lutz, Interdiciplinary Procedures Center, Strategic Laboratory	Claudio Tavares Sacchi, Claudia Regina Gonçalves, Erica Valessa Ramos Gomes, Karoline Rodrigues Campos, Caio Vinicius Dias Lopes
EPI_ISL_1468428	SAE Servico de Atendimento Especializado	Instituto Adolfo Lutz, Interdiciplinary Procedures Center, Strategic Laboratory	Claudio Tavares Sacchi, Claudia Regina Gonçalves, Erica Valessa Ramos Gomes, Karoline Rodrigues Campos, Caio Vinicius Dias Lopes
EPI_ISL_1468931, EPI_ISL_1468935	San Diego County Public Health Laboratory	Andersen lab at Scripps Research	SEARCH Alliance San Diego with Tracy Basler, Jovan Shephard, Brett Austin
EPI_ISL_1469620	FUNDACAO DE SAUDE PUBLICA DE NOVO HAMBURGO FSNH	Epiclin	Fernando Hayashi Sant'Anna, Ana Paula Muterle, Janira Prichula, Juliana Comerlato, Carolina Comerlato, Eliana Márcia Da Ros Wendland
EPI_ISL_1470440, EPI_ISL_1470457, EPI_ISL_1470478, EPI_ISL_1470491	Genetica Molecular and Subdepartamento de Virologia ISP Chile	Instituto de Salud Publica de Chile	Javier Tognarelli, Karen Orostica, Barbara Parra, Loredana Arata, Jaime Lagos, Gisselle Barra, Patricia Bustos, Rodrigo Fasce, Andres Castillo, Jorge Fernandez
EPI_ISL_1495031	Laboratório de Biologia Integrativa	Laboratório de Biologia Integrativa	Filipe Romero Rebello Moreira, Diego Menezes Bonfim, Victor Emmanuel Viana Geddes, Danielle Alves Gomes Zauli, Joice do Prado Silva, Aline Brito de Lima, Frederico Scott Varella Malta, Alessandro Clayton de Souza Ferreira, Victor Cavalcanti Pardini, Daniel Costa Queiroz, Rafael Marques de Souza, Lucyene Miguita Luiz, Paula Luize Camargos Fonseca, Rennan Garcias Moreira, Nuno Rodrigues Faria, Carolina Moreira Voloch, Renan Pedra de Souza, Renato Santana Aguiar
EPI_ISL_1511207	Hospital General Universitario Gregorio Marañón	Hospital General Universitario Gregorio Marañón	Sergio Buenestado Serrano, Pedro Sola Campoy, Laura Pérez-Lago, Cristina Rodriguez-Grande, Pilar Catalán, Patricia Muñoz, Darío García de Viedma
EPI_ISL_1520109	LACEN do Estado de Rondonia	Instituto Adolfo Lutz, Interdiciplinary Procedures Center, Strategic Laboratory	Claudio Tavares Sacchi, Claudia Regina Gonçalves, Erica Valessa Ramos Gomes, Karoline Rodrigues Campos, Caio Vinicius Dias Lopes
EPI_ISL_1520129	Centro de Saude II Dr Jose Paione Mococa	Instituto Adolfo Lutz, Interdiciplinary Procedures Center, Strategic Laboratory	Claudio Tavares Sacchi, Claudia Regina Gonçalves, Erica Valessa Ramos Gomes, Karoline Rodrigues Campos, Caio Vinicius Dias Lopes
EPI_ISL_1521309, EPI_ISL_1521313, EPI_ISL_1521335	Dutch COVID-19 response team	National Institute for Public Health and the Environment (RIVM)	Adam Meijer, Harry Vennema, Dirk Eggink, Jeroen Cremer, Sharon van den Brink, Bas van der Veer, AnneMarie van den Brandt, Lisa Wijsman, Kim Freriks, Ryanne Jaarsma, Euníce Then, Jolienke Hardeman, Lynn Aarts, Sanne Bos, Melissa van Tuil, Robert Kohl, Linda van de Nes, Sjoerd Kuiling, James Groot, Florian Zwagemaker, Dennis Schmitz, Annelies Kroneman, Karim Hajji, Chantal Reusken, on behalf of the national COVID-19 response team
EPI_ISL_1533015, EPI_ISL_1533017	Lab voor klinische biologie	Lab voor klinische biologie	Marija Janevska, Hannelore Hamerlinck, Bruno Verhasselt
EPI_ISL_1533725	Hospital Geral de Guarulhos	Instituto Adolfo Lutz, Interdiciplinary Procedures Center, Strategic Laboratory	Claudio Tavares Sacchi, Claudia Regina Gonçalves, Erica Valessa Ramos Gomes, Karoline Rodrigues Campos, Caio Vinicius Dias Lopes, Leonardo Jose Tadeu de Araujo
EPI_ISL_1534009, EPI_ISL_1534010	Laboratorio Central de Saude Publica do Estado de Santa Catarina (LACEN-SC)	Laboratory of Respiratory Viruses and Measles, Oswaldo Cruz Institute, FIOCRUZ	Paola Resende, Luciana Appolinario, Fernando Motta, Anna Carolina Paixao, Ana Carolina Mendonca, Alice Sampaio Rocha, Renata Serrano Lopes, Darcita Buerger Rovaris, Sandra Bianchini Fernandes, Marilda Siqueira on behalf of the Fiocruz COVID-19 Genomic Surveillance Network
EPI_ISL_1534522	Ministry of Health Turkey	Ministry of Health Turkey	Fatma Bayrakdar, Yasemin Cosgun, Suleyman Yalcin, Gulay Korukluoglu
EPI_ISL_1554691	Helix/Illumina	Centers for Disease Control and Prevention Division of Viral Diseases, Pathogen Discovery	Dakota Howard, Dhwani Batra, Peter W. Cook, Kara Moser, Adrian Paskey, Jason Caravas, Benjamin Rambo-Martin, Shatavia Morrison, Christopher Gulvick, Scott Sammons, Yvette Unoarumhi, Darlene Wagner, Matthew Schmerer, Eileen de Feo, Jan Antico, Christine Tran, Matthew Tolentino, Shannon Wickline, Kim Gietzen, Brad Sickler, Jingtao Liu, Eric Allen, Phil Febbo, Nicole L. Washington, Simon White, Geraint Levan, Kelly Schiabor Barrett, Elizabeth Cirulli, Alexandre Bolze, Ary Ascencio, Charlotte Rivera-Garcia, Ryan Cho, Jason Nguyen, Sherry Wang, Jimmy Ramirez, Tyler Cassens, Efren Sandoval, Magnus Isaksson, William Lee, David Becker, Marc Laurent, James Lu, Clinton R. Paden, Duncan MacCannell
EPI_ISL_1559815	Aegis Sciences Corporation	Centers for Disease Control and Prevention Division of Viral Diseases, Pathogen Discovery	Dakota Howard, Dhwani Batra, Peter W. Cook, Kara Moser, Adrian Paskey, Jason Caravas, Benjamin Rambo-Martin, Shatavia Morrison, Christopher Gulvick, Scott Sammons, Yvette Unoarumhi, Darlene Wagner, Matthew Schmerer, Cyndi Clark, Patrick Campbell, Rob Case, Vikramsinha Ghorpade, Holly Houdeshell, Ola Kvalvaag, Dillon Nall, Ethan Sanders, Alec Vest, Shaun Westlund, Matthew Hardison, Clinton R. Paden, Duncan MacCannell
EPI_ISL_1583665, EPI_ISL_1583725	Central Public Health Laboratory - LACEN -Bahia, Salvador, Brazil	Central Public Health Laboratory - LACEN -Bahia, Salvador, Brazil	Stephane Tosta, Luciana Oliveira, Vanessa Nardy,Patrícia Cajado,Marcela Gómez, Breno Dominguez, Jaqueline Gomes, Vagner Fonseca,Marta Giovanetti,Luiz Alcantara, Felicidade Pereira, Arabela Leal

EPI_ISL_1608161	Hospital São Rafael - IDOR	Hospital São Rafael - IDOR	Carolina Kymie Vasques Nonaka, Tiago Gräf, Camila Araújo de Lorenzo Barcia, Vanessa Ferreira Costa, Janderson Lopes de Oliveira, Rogério da Hora Passos, Clarissa Araújo Gurgel Rocha, Iasmin Nogueira Bastos, Maria Clara Brito de Santana, Ian Marinho Santos, Karoline Almeida Felix de Sousa, Isadora Cristina de Siqueira, Thamires Gomes Lopes Weber, Ana Verena Almeida Mendes, Bruno Solano de Freitas Souza
EPI_ISL_1612742	Laboratory Corporation of America	Centers for Disease Control and Prevention Division of Viral Diseases, Pathogen Discovery	Dakota Howard, Dhwani Batra, Peter W. Cook, Kara Moser, Adrian Paskey, Jason Caravas, Benjamin Rambo-Martin, Shatavia Morrison, Christopher Gulvick, Scott Sammons, Yvette Unoarumhi, Darlene Wagner, Matthew Schmerer, Minoo Agarwal, Eyad Almasri, Debbie Boles, Ayla Burns, Nuthawin Charoensri, Oren Cohen, Susan Countryman, Mary Ann Cristobal, Bobbi Croy, Suzanne Dale, Hrushikesh Deshmukh, Amanda Douglas, Vincent Drouillon, Marcia Eisenberg, Howard Engler, Rama Ghatti, Prashant Gupta, Susan Hicks, Jake Humphrey, Lax Iyer, Manoj Jain, Mohan Kolli, Brian Krueger, Tim Kuphal, Stanley Letovsky, Michael Levandoski, Craig Lukasik, Jonathan Meltzer, Brian Norvell, Mindy Nye, Scott Parker, Christos Petropoulos, John Pruitt, Steven Ragan, Scott Ryan, Mike Sapeta, Jana Schroth, Suresh Babu Selvaraju, Goran Stevovic, Amanda Suchanek, Andrea Throop, Lyndon Tilson, Thomas Urban, Joe Voshell, Kimberly Wagner, Jonathan Williams, Mary Williamson, Qian Zeng, Tricia Zwiefelhofer, Clinton R. Paden, Duncan MacCannell
EPI_ISL_1640682	MVZ Labor Krone GbR	Robert Koch Institute	unknown
EPI_ISL_1649154, EPI_ISL_1650624	Aegis Sciences Corporation	Centers for Disease Control and Prevention Division of Viral Diseases, Pathogen Discovery	Dakota Howard, Dhwani Batra, Peter W. Cook, Kara Moser, Adrian Paskey, Jason Caravas, Benjamin Rambo-Martin, Shatavia Morrison, Christopher Gulvick, Scott Sammons, Yvette Unoarumhi, Darlene Wagner, Matthew Schmerer, Cyndi Clark, Patrick Campbell, Rob Case, Vikramsinha Ghorpade, Holly Houdeshell, Ola Kvalvaag, Dillon Nall, Ethan Sanders, Alec Vest, Shaun Westlund, Matthew Hardison, Clinton R. Paden, Duncan MacCannell
EPI_ISL_1664164, EPI_ISL_1664169	Laboratorio Central Noel Nutels	Bioinformatics Laboratory / LNCC	Luiz G P de Almeida, Alessandra P Lamarca, Ronaldo da Silva F Jr, Liliane Cavalcante, Alexandra L Gerber, Ana Paula de C Guimarães, Douglas Terra Machado, Cassia Alves, Diana Mariani, Thais Felix Cruz, Mario Sergio Ribeiro, Silvia Carvalho, Flávio Dias da Silva, Marcio Henrique de Oliveira Garcia, Leandro Magalhães de Souza, Cristiane Gomes da Silva, Caio Luiz Pereira Ribeiro, Andréa Cony Cavalcanti, Claudia Maria Braga de Mello, Amilcar Tanuri, Ana Tereza R Vasconcelos
EPI_ISL_1686186, EPI_ISL_1690099	Aegis Sciences Corporation	Centers for Disease Control and Prevention Division of Viral Diseases, Pathogen Discovery	Dakota Howard, Dhwani Batra, Peter W. Cook, Kara Moser, Adrian Paskey, Jason Caravas, Benjamin Rambo-Martin, Shatavia Morrison, Christopher Gulvick, Scott Sammons, Yvette Unoarumhi, Darlene Wagner, Matthew Schmerer, Cyndi Clark, Patrick Campbell, Rob Case, Vikramsinha Ghorpade, Holly Houdeshell, Ola Kvalvaag, Dillon Nall, Ethan Sanders, Alec Vest, Shaun Westlund, Matthew Hardison, Clinton R. Paden, Duncan MacCannell
EPI_ISL_1712329	Genetica Molecular and Subdepartamento de Virologia ISP Chile	Instituto de Salud Publica de Chile	Javier Tognarelli, Karen Orostica, Barbara Parra, Loredana Arata, Gisselle Barra, Patricia Bustos, Rodrigo Fasce, Andres Castillo, Soledad Ulloa, Jorge Fernandez
EPI_ISL_1717000	Pathology and Laboratory Medicine Institute, Cleveland Clinic, Ohio, USA	Pathology and Laboratory Medicine Institute, Cleveland Clinic, Ohio, USA	Jessica Spildener, Joy Nakitandwe, Kristen McDonnell, David Plunkett, Zheng Jin Tu, Jay Brock, Yu-Wei Cheng, Gary Procop, Daniel Rhoads, Daniel H. Farkas, David Bosler
EPI_ISL_1760757	Minnesota Department of Health, Public Health Laboratory	Minnesota Department of Health, Public Health Laboratory	Alexandra Lorentz, Jacob Garfin, Matt Plumb, and Xiong Wang
EPI_ISL_1785271	National Virus Reference Laboratory	National Virus Reference Laboratory	Zoe Yandle, Charlene Bennett, Gabriel Gonzalez, Michael Carr, Jonathan Dean, Cillian F De Gascun
EPI_ISL_1794316	Wisconsin State Laboratory of Hygiene Communicable Disease Division	Wisconsin State Laboratory of Hygiene Communicable Disease Division	Kelsey R. Florek, Abigail C. Shockey, Alicia J. Mooney, Sara Wagner
EPI_ISL_1794737	Scripps Medical Laboratory	Andersen lab at Scripps Research	SEARCH Alliance San Diego with Michael Quigley, Ellen Stefanski, Ian Mchardy
EPI_ISL_1794755	Sharp HealthCare Laboratory	Andersen lab at Scripps Research	SEARCH Alliance San Diego with Aaron Harding, Jacquelyn Berumen, Cathy Woerle, Liam McGinnis, Art Mendoza, Omid Bakhtar
EPI_ISL_1795141	POLICLINICA HORTOLANDIA	Instituto Butantan / ESALQ-Piracicaba	Instituto Butantan: Alexander Roberto Precioso, Dimas Tadeu Covas, Sandra Coccuzzo Sampaio, Maria Carolina Elias, José Salvatore Leister Patané, Vincent Louis Viala, Antonio Jorge Martins, Ricardo Haddad, Claudia Renata dos Santos Barros, Elaine Cristina Marqueze, Raul Machado Neto, Debora Botequio Moretti. Centro de Genômica Funcional da ESALQ: Luiz Lehmann Coutinho, Ricardo Augusto Brassaloti, Raquel de Lello Rocha Campos Cassano. NGS Soluções Genômicas: Pilar Drummond Sampaio Corrêa Mariani. FZEA-USP Pirassununga: Mirele Daiana Poleti, Jessika Cristina Chagas Lesbon, Elisangela Chicaroni Mattos, Heidge Fukumasu. USP-Botucatu: Rejane Maria Tommasini Grotto, Jayme A. Souza-Neto, Guilherme Targino Valente, Patricia Akemi Assato, Felipe Allan da Silva da Costa, Bianca Cechetto Carlos. Mendelics: Bibiana Santos, João Paulo Kitajima, Erika Freitas, David Schlesinger. Hemocentro Ribeirão Preto: Simone Kashima, Evandra Strazza Rodrigues, Svetoslav Nanev Slavov, Elaine Vieira dos Santos, Rafael dos Santos Bezerra, Luiz Carlos Junior de Alcantara, Marta Giovanetti, Vagner Fonseca, Flavia Aburjaile, Rodrigo Tocantins Calado.
EPI_ISL_1795161	CS III VILA ODILON	Instituto Butantan / ESALQ-Piracicaba	Instituto Butantan: Alexander Roberto Precioso, Dimas Tadeu Covas, Sandra Coccuzzo Sampaio, Maria Carolina Elias, José Salvatore Leister Patané, Vincent Louis Viala, Antonio Jorge Martins, Ricardo Haddad, Claudia Renata dos Santos Barros, Elaine Cristina Marqueze, Raul Machado Neto, Debora Botequio Moretti. Centro de Genômica Funcional da ESALQ: Luiz Lehmann Coutinho, Ricardo Augusto Brassaloti, Raquel de Lello Rocha Campos Cassano. NGS Soluções Genômicas: Pilar Drummond Sampaio Corrêa Mariani. FZEA-USP Pirassununga: Mirele Daiana Poleti, Jessika Cristina Chagas Lesbon, Elisangela Chicaroni Mattos, Heidge Fukumasu. USP-Botucatu: Rejane Maria Tommasini Grotto, Jayme A. Souza-Neto, Guilherme Targino Valente, Patricia Akemi Assato, Felipe Allan da Silva da Costa, Bianca Cechetto Carlos. Mendelics: Bibiana Santos, João Paulo Kitajima, Erika Freitas, David Schlesinger. Hemocentro Ribeirão Preto: Simone Kashima, Evandra Strazza Rodrigues, Svetoslav Nanev Slavov, Elaine Vieira dos Santos, Rafael dos Santos Bezerra, Luiz Carlos Junior de Alcantara, Marta Giovanetti, Vagner Fonseca, Flavia Aburjaile, Rodrigo Tocantins Calado.
EPI_ISL_1795184	POLICLINICA HORTOLANDIA	Instituto Butantan / ESALQ-Piracicaba	Instituto Butantan: Alexander Roberto Precioso, Dimas Tadeu Covas, Sandra Coccuzzo Sampaio, Maria Carolina Elias, José Salvatore Leister Patané, Vincent Louis Viala, Antonio Jorge Martins, Ricardo Haddad, Claudia Renata dos Santos Barros, Elaine Cristina Marqueze, Raul Machado Neto, Debora Botequio Moretti. Centro de Genômica Funcional da ESALQ: Luiz Lehmann Coutinho, Ricardo Augusto Brassaloti, Raquel de Lello Rocha Campos Cassano. NGS Soluções Genômicas: Pilar Drummond Sampaio Corrêa Mariani. FZEA-USP Pirassununga: Mirele Daiana Poleti, Jessika Cristina Chagas Lesbon, Elisangela Chicaroni Mattos, Heidge Fukumasu. USP-Botucatu: Rejane Maria Tommasini Grotto, Jayme A. Souza-Neto, Guilherme Targino Valente, Patricia Akemi Assato, Felipe Allan da Silva da Costa, Bianca Cechetto Carlos. Mendelics: Bibiana Santos, João Paulo Kitajima, Erika Freitas, David Schlesinger. Hemocentro Ribeirão Preto: Simone Kashima, Evandra Strazza Rodrigues, Svetoslav Nanev Slavov, Elaine Vieira dos Santos, Rafael dos Santos Bezerra, Luiz Carlos Junior de Alcantara, Marta Giovanetti, Vagner Fonseca, Flavia Aburjaile, Rodrigo Tocantins Calado.
EPI_ISL_1795200	AMBULATORIO DE ESPECIALIDADE V E MOGI MIRIM	Instituto Butantan / ESALQ-Piracicaba	Instituto Butantan: Alexander Roberto Precioso, Dimas Tadeu Covas, Sandra Coccuzzo Sampaio, Maria Carolina Elias, José Salvatore Leister Patané, Vincent Louis Viala, Antonio Jorge Martins, Ricardo Haddad, Claudia Renata dos Santos Barros, Elaine Cristina Marqueze, Raul Machado Neto, Debora Botequio Moretti. Centro de Genômica Funcional da ESALQ: Luiz Lehmann Coutinho, Ricardo Augusto Brassaloti, Raquel de Lello Rocha Campos Cassano. NGS Soluções Genômicas: Pilar Drummond Sampaio Corrêa Mariani. FZEA-USP Pirassununga: Mirele Daiana Poleti, Jessika Cristina Chagas Lesbon, Elisangela Chicaroni Mattos, Heidge Fukumasu. USP-Botucatu: Rejane Maria Tommasini Grotto, Jayme A. Souza-Neto, Guilherme Targino Valente, Patricia Akemi Assato, Felipe Allan da Silva da Costa, Bianca Cechetto Carlos. Mendelics: Bibiana Santos, João Paulo Kitajima, Erika Freitas, David Schlesinger. Hemocentro Ribeirão Preto: Simone Kashima, Evandra Strazza Rodrigues, Svetoslav Nanev Slavov, Elaine Vieira dos Santos, Rafael dos Santos Bezerra, Luiz Carlos Junior de Alcantara, Marta Giovanetti, Vagner Fonseca, Flavia Aburjaile, Rodrigo Tocantins Calado.
EPI_ISL_1795309, EPI_ISL_1795323	LABORATORIO DE FRANCA	Instituto Butantan / ESALQ-Piracicaba	Instituto Butantan: Alexander Roberto Precioso, Dimas Tadeu Covas, Sandra Coccuzzo Sampaio, Maria Carolina Elias, José Salvatore Leister Patané, Vincent Louis Viala, Antonio Jorge Martins, Ricardo Haddad, Claudia Renata dos Santos Barros, Elaine Cristina Marqueze, Raul Machado Neto, Debora Botequio Moretti. Centro de Genômica Funcional da ESALQ: Luiz Lehmann Coutinho, Ricardo Augusto Brassaloti, Raquel de Lello Rocha Campos Cassano. NGS Soluções Genômicas: Pilar Drummond Sampaio Corrêa Mariani. FZEA-USP Pirassununga: Mirele Daiana Poleti, Jessika Cristina Chagas Lesbon, Elisangela Chicaroni Mattos, Heidge Fukumasu. USP-Botucatu: Rejane Maria Tommasini Grotto, Jayme A. Souza-Neto, Guilherme Targino Valente, Patricia Akemi Assato, Felipe Allan da Silva da Costa, Bianca Cechetto Carlos. Mendelics: Bibiana Santos, João Paulo Kitajima, Erika Freitas, David Schlesinger. Hemocentro Ribeirão Preto: Simone Kashima, Evandra Strazza Rodrigues, Svetoslav Nanev Slavov, Elaine Vieira dos Santos, Rafael dos Santos Bezerra, Luiz Carlos Junior de Alcantara, Marta Giovanetti, Vagner Fonseca, Flavia Aburjaile, Rodrigo Tocantins Calado.
EPI_ISL_1795362, EPI_ISL_1795363	UNIDADE BASICA DE SAUDE DE PIQUETE	Instituto Butantan / ESALQ-Piracicaba	Instituto Butantan: Alexander Roberto Precioso, Dimas Tadeu Covas, Sandra Coccuzzo Sampaio, Maria Carolina Elias, José Salvatore Leister Patané, Vincent Louis Viala, Antonio Jorge Martins, Ricardo Haddad, Claudia Renata dos Santos Barros, Elaine Cristina Marqueze, Raul Machado Neto, Debora Botequio Moretti. Centro de Genômica Funcional da ESALQ: Luiz Lehmann Coutinho, Ricardo Augusto Brassaloti, Raquel de Lello Rocha Campos Cassano. NGS Soluções Genômicas: Pilar Drummond Sampaio Corrêa Mariani. FZEA-USP Pirassununga: Mirele Daiana Poleti, Jessika Cristina Chagas Lesbon, Elisangela Chicaroni Mattos, Heidge Fukumasu. USP-Botucatu: Rejane Maria Tommasini Grotto, Jayme A. Souza-Neto, Guilherme Targino Valente, Patricia Akemi Assato, Felipe Allan da Silva da Costa, Bianca Cechetto Carlos. Mendelics: Bibiana Santos, João Paulo Kitajima, Erika Freitas,

			David Schlesinger. Hemocentro Ribeirão Preto: Simone Kashima, Evandra Strazza Rodrigues, Svetoslav Nanev Slavov, Elaine Vieira dos Santos, Rafael dos Santos Bezerra, Luiz Carlos Junior de Alcantara, Marta Giovanetti, Vagner Fonseca, Flavia Aburjaile, Rodrigo Tocantins Calado.
EPI_ISL_1795384	USF SALERNO	Instituto Butantan / ESALQ-Piracicaba	Instituto Butantan: Alexander Roberto Precioso, Dimas Tadeu Covas, Sandra Coccuzzo Sampaio, Maria Carolina Elias, José Salvatore Leister Patané, Vincent Louis Viala, Antonio Jorge Martins, Ricardo Haddad, Claudia Renata dos Santos Barros, Elaine Cristina Marqueze, Raul Machado Neto, Debora Botequio Moretti. Centro de Genômica Funcional da ESALQ: Luiz Lehmann Coutinho, Ricardo Augusto Brassaloti, Raquel de Lello Rocha Campos Cassano. NGS Soluções Genômicas: Pilar Drummond Sampaio Corrêa Mariani. FZEA-USP Pirassununga: Mirele Daiana Poleti, Jessika Cristina Chagas Lesbon, Elisangela Chicaroni Mattos, Heidge Fukumasu. USP-Botucatu: Rejane Maria Tommasini Grotto, Jayme A. Souza-Neto, Guilherme Targino Valente, Patricia Akemi Assato, Felipe Allan da Silva da Costa, Bianca Cechetto Carlos. Mendelics: Bibiana Santos, João Paulo Kitajima, Erika Freitas, David Schlesinger. Hemocentro Ribeirão Preto: Simone Kashima, Evandra Strazza Rodrigues, Svetoslav Nanev Slavov, Elaine Vieira dos Santos, Rafael dos Santos Bezerra, Luiz Carlos Junior de Alcantara, Marta Giovanetti, Vagner Fonseca, Flavia Aburjaile, Rodrigo Tocantins Calado.
EPI_ISL_1798542	NE Public Health Laboratory	Centers for Disease Control and Prevention Division of Viral Diseases, Pathogen Discovery	Mili Sheth, Sarah Nobles, Jasmine Padilla, Mark Burroughs, Shoshona Le, Katie Dillon, Peter Cook, Clinton R. Paden, Dhwani Batra, Krista Queen, Kristen Knipe, Dakota Howard, Yvette Unoarumhi, Darlene Wagner, Matthew Schmerer, Ben L. Rambo-Martin, Kristine Lacek, Sam Shepard, Alison Laufer Halpin, Dave Wentworth, Vivien Dugan, Suxiang Tong, Justin Lee
EPI_ISL_1836536	Aegis Sciences Corporation	Centers for Disease Control and Prevention Division of Viral Diseases, Pathogen Discovery	Dakota Howard, Dhwani Batra, Peter W. Cook, Kara Moser, Adrian Paskey, Jason Caravas, Benjamin Rambo-Martin, Shatavia Morrison, Christopher Gulvick, Scott Sammons, Yvette Unoarumhi, Darlene Wagner, Matthew Schmerer, Cyndi Clark, Patrick Campbell, Rob Case, Vikramsinha Ghorpade, Holly Houdeshell, Ola Kvalvaag, Dillon Nall, Ethan Sanders, Alec Vest, Shaun Westlund, Matthew Hardison, Clinton R. Paden, Duncan MacCannell
EPI_ISL_1846787	Labor Becker & Kollegen (Standort MÃ¼nchen)	Robert Koch Institute	unknown
EPI_ISL_1858391	Laboratorio Central Noel Nutels	Bioinformatics Laboratory / LNCC	Luiz G P de Almeida, Alessandra P Lamarca, Ronaldo da Silva F Jr, Liliane Cavalcante, Alexandra L Gerber, Ana Paula de C Guimarães, Douglas Terra Machado, Cassia Alves, Diana Mariani, Thais Felix Cruz, Mario Sergio Ribeiro, Silvia Carvalho, Flávio Dias da Silva, Marcio Henrique de Oliveira Garcia, Leandro Magalhães de Souza, Cristiane Gomes da Silva, Caio Luiz Pereira Ribeiro, Andréa Cony Cavalcanti, Claudia Maria Braga de Mello, Amilcar Tanuri, Ana Tereza R Vasconcelos
EPI_ISL_1858777, EPI_ISL_1858792, EPI_ISL_1858800	Unidade de apoio ao diagnóstico da COVID - UNADIG	Bioinformatics Laboratory / LNCC	Luiz G P de Almeida, Alessandra P Lamarca, Ronaldo da Silva F Jr, Liliane Cavalcante, Alexandra L Gerber, Ana Paula de C Guimarães, Douglas Terra Machado, Cassia Alves, Diana Mariani, Thais Felix Cruz, Mario Sergio Ribeiro, Silvia Carvalho, Flávio Dias da Silva, Marcio Henrique de Oliveira Garcia, Leandro Magalhães de Souza, Cristiane Gomes da Silva, Caio Luiz Pereira Ribeiro, Andréa Cony Cavalcanti, Claudia Maria Braga de Mello, Amilcar Tanuri, Ana Tereza R Vasconcelos
EPI_ISL_1864209, EPI_ISL_1868237	Department of Virus and Microbiological Special Diagnostics, Statens Serum Institut, Copenhagen, Denmark	Aalborg University	Danish Covid-19 Genome Consortium
EPI_ISL_1897379	Washington State Department of Health Public Health Laboratories	Washington State Department of Health Public Health Laboratories	Drew MacKellar, Philip Dykema, Denny Russell, Joenice Gonzalez, Hannah Gray, Geoff Melly, Vanessa De Los Santos, Darren Lucas, JohnAric Peterson, Avi Singh, Rebecca Cao
EPI_ISL_1904855	Canterbury Health Laboratories	Institute of Environmental Science and Research (ESR)	Rachel Boyle, SallyAnn Harbison, Olivia Stroeven, Xiaoyun Ren, Matt Storey, Nikki Freed, Muhammad Faisal, Jing Wang, Hermes Perez, Anja Werno, Antje van der Linden, Arlo Upton, Chris Mansell, David Hammer, Dragana Drinkovic, Gary McAuliffe, Hana Sofia Andersson, James Ussher, Jill Sherwood, Josh Freeman, Julia Howard, Juliet Elvy, Mary DeAlmeida, Matt Blakiston, Matthew Rogers, Max Bloomfield, Michael Addidle, Michelle Balm, Sally Roberts, Sarah Jefferies, Sharmini Muttaiyah, Susan Morpeth, Susan Taylor, Timothy Blackmore, Vani Sathyendran, Veronica Playle, Virginia Hope, Erasmus Smit, Lauren Jelly, Olin Silander, Joep de Ligt
EPI_ISL_1911765	Ministry of Health Turkey	Ministry of Health Turkey	Fatma Bayrakdar, Yasemin Cosgun, Suleyman Yalcin, Gulay Korukluoglu
EPI_ISL_1929424, EPI_ISL_1929427	Chiba Prefectural Institute of Public Health	Pathogen Genomics Center, National Institute of Infectious Diseases	Tsuyoshi Sekizuka, Kentaro Itokawa, Rina Tanaka, Masanori Hashino, Hidemasa Izumiya, Sunao Iyoda, Shouji Yamamoto, Masatomo Morita, Ken-ichi Lee, Nobuo Koizumi, Makoto Kuroda
EPI_ISL_1935561, EPI_ISL_1935566, EPI_ISL_1935578, EPI_ISL_1935581	Quest Diagnostics Incorporated	Centers for Disease Control and Prevention Division of Viral Diseases, Pathogen Discovery	Dakota Howard, Dhwani Batra, Peter W. Cook, Kara Moser, Adrian Paskey, Jason Caravas, Benjamin Rambo-Martin, Shatavia Morrison, Christopher Gulvick, Scott Sammons, Yvette Unoarumhi, Darlene Wagner, Matthew Schmerer, S. H. Rosenthal, A. Gerasimova, R. M. Kagan, B. Anderson, M. Hua, Y. Liu, L.E. Bernstein, K.E. Livingston, A. Perez, I. A. Shlyakhter, R. V. Rolando, R. Owen, P. Tanpaiboon, F. Lacbawan, Clinton R. Paden, Duncan MacCannell
EPI_ISL_1966113	CS II EGIDIO BRUNHARA MORRO AGUDO	Instituto Butantan / Mendelics	Instituto Butantan: Dimas Tadeu Covas, Sandra Coccuzzo Sampaio, Maria Carolina Elias, José Salvatore Leister Patané, Vincent Louis Viala, Antonio Jorge Martins, Ricardo Haddad, Claudia Renata dos Santos Barros, Elaine Cristina Marqueze, Raul Machado Neto, Debora Botequio Moretti, Jardelina de Souza Todao Bernardino, Loyze Paola Oliveira de Lima, Luiz Aurelio de Campos Crispin. Centro de Genômica Funcional da ESALQ: Luiz Lehmann Coutinho, Ricardo Augusto Brassaloti, Raquel de Lello Rocha Campos Cassano. NGS Soluções Genômicas: Pilar Drummond Sampaio Corrêa Mariani. FZEA-USP Pirassununga: Mirele Daiana Poleti, Jessika Cristina Chagas Lesbon, Elisangela Chicaroni Mattos, Heidge Fukumasu. USP-Botucatu: Rejane Maria Tommasini Grotto, Jayme A. Souza-Neto, Guilherme Targino Valente, Patricia Akemi Assato, Felipe Allan da Silva da Costa, Bianca Cechetto Carlos. Mendelics: Bibiana Santos, João Paulo Kitajima, Erika Freitas, David Schlesinger. Hemocentro Ribeirão Preto: Simone Kashima, Evandra Strazza Rodrigues, Svetoslav Nanev Slavov, Elaine Vieira dos Santos, Rafael dos Santos Bezerra, Luiz Carlos Junior de Alcantara, Marta Giovanetti, Vagner Fonseca, Flavia Aburjaile, Rodrigo Tocantins Calado. FAMERP-SJRP: Cecília Artico Banho, Lívia Sacchetto, Fábio Sossai Possebon, Leila Sabrina Ullmann, Cíntia Bittar, Guilherme Campos, Helena Lage Ferreira, Jorge A. Petroli Marchesi, Maisa C. Pereira Parra, Marília Moraes, Paula Rahal, Paulo Inacio da Costa, João Pessoa Araújo Jr., Maurício Lacerda Nogueira. Prefeitura de Sao Paulo: Melissa Palmieri.
EPI_ISL_1966358	SMS SECRETARIA MUNICIPAL DE SAUDE DE BOITUVA	Instituto Butantan / Mendelics	Instituto Butantan: Dimas Tadeu Covas, Sandra Coccuzzo Sampaio, Maria Carolina Elias, José Salvatore Leister Patané, Vincent Louis Viala, Antonio Jorge Martins, Ricardo Haddad, Claudia Renata dos Santos Barros, Elaine Cristina Marqueze, Raul Machado Neto, Debora Botequio Moretti, Jardelina de Souza Todao Bernardino, Loyze Paola Oliveira de Lima, Luiz Aurelio de Campos Crispin. Centro de Genômica Funcional da ESALQ: Luiz Lehmann Coutinho, Ricardo Augusto Brassaloti, Raquel de Lello Rocha Campos Cassano. NGS Soluções Genômicas: Pilar Drummond Sampaio Corrêa Mariani. FZEA-USP Pirassununga: Mirele Daiana Poleti, Jessika Cristina Chagas Lesbon, Elisangela Chicaroni Mattos, Heidge Fukumasu. USP-Botucatu: Rejane Maria Tommasini Grotto, Jayme A. Souza-Neto, Guilherme Targino Valente, Patricia Akemi Assato, Felipe Allan da Silva da Costa, Bianca Cechetto Carlos. Mendelics: Bibiana Santos, João Paulo Kitajima, Erika Freitas, David Schlesinger. Hemocentro Ribeirão Preto: Simone Kashima, Evandra Strazza Rodrigues, Svetoslav Nanev Slavov, Elaine Vieira dos Santos, Rafael dos Santos Bezerra, Luiz Carlos Junior de Alcantara, Marta Giovanetti, Vagner Fonseca, Flavia Aburjaile, Rodrigo Tocantins Calado. FAMERP-SJRP: Cecília Artico Banho, Lívia Sacchetto, Fábio Sossai Possebon, Leila Sabrina Ullmann, Cíntia Bittar, Guilherme Campos, Helena Lage Ferreira, Jorge A. Petroli Marchesi, Maisa C. Pereira Parra, Marília Moraes, Paula Rahal, Paulo Inacio da Costa, João Pessoa Araújo Jr., Maurício Lacerda Nogueira. Prefeitura de Sao Paulo: Melissa Palmieri.
EPI_ISL_1966380	HOSPITAL REGIONAL DE ITAPETININGA	Instituto Butantan / Mendelics	Instituto Butantan: Dimas Tadeu Covas, Sandra Coccuzzo Sampaio, Maria Carolina Elias, José Salvatore Leister Patané, Vincent Louis Viala, Antonio Jorge Martins, Ricardo Haddad, Claudia Renata dos Santos Barros, Elaine Cristina Marqueze, Raul Machado Neto, Debora Botequio Moretti, Jardelina de Souza Todao Bernardino, Loyze Paola Oliveira de Lima, Luiz Aurelio de Campos Crispin. Centro de Genômica Funcional da ESALQ: Luiz Lehmann Coutinho, Ricardo Augusto Brassaloti, Raquel de Lello Rocha Campos Cassano. NGS Soluções Genômicas: Pilar Drummond Sampaio Corrêa Mariani. FZEA-USP Pirassununga: Mirele Daiana Poleti, Jessika Cristina Chagas Lesbon, Elisangela Chicaroni Mattos, Heidge Fukumasu. USP-Botucatu: Rejane Maria Tommasini Grotto, Jayme A. Souza-Neto, Guilherme Targino Valente, Patricia Akemi Assato, Felipe Allan da Silva da Costa, Bianca Cechetto Carlos. Mendelics: Bibiana Santos, João Paulo Kitajima, Erika Freitas, David Schlesinger. Hemocentro Ribeirão Preto: Simone Kashima, Evandra Strazza Rodrigues, Svetoslav Nanev Slavov, Elaine Vieira dos Santos, Rafael dos Santos Bezerra, Luiz Carlos Junior de Alcantara, Marta Giovanetti, Vagner Fonseca, Flavia Aburjaile, Rodrigo Tocantins Calado. FAMERP-SJRP: Cecília Artico Banho, Lívia Sacchetto, Fábio Sossai Possebon, Leila Sabrina Ullmann, Cíntia Bittar, Guilherme Campos, Helena Lage Ferreira, Jorge A. Petroli Marchesi, Maisa C. Pereira Parra, Marília Moraes, Paula Rahal, Paulo Inacio da Costa, João Pessoa Araújo Jr., Maurício Lacerda Nogueira. Prefeitura de Sao Paulo: Melissa Palmieri.
EPI_ISL_1966410	HOSPITAL GERAL DE VILA NOVA CACHOEIRINHA SAO PAULO	Instituto Butantan / Mendelics	Instituto Butantan: Dimas Tadeu Covas, Sandra Coccuzzo Sampaio, Maria Carolina Elias, José Salvatore Leister Patané, Vincent Louis Viala, Antonio Jorge Martins, Ricardo Haddad, Claudia Renata dos Santos Barros, Elaine Cristina Marqueze, Raul Machado Neto, Debora Botequio Moretti, Jardelina de Souza Todao Bernardino, Loyze Paola Oliveira de Lima, Luiz Aurelio de Campos Crispin. Centro de Genômica Funcional da ESALQ: Luiz Lehmann Coutinho, Ricardo Augusto Brassaloti, Raquel de Lello Rocha Campos Cassano. NGS Soluções Genômicas: Pilar Drummond Sampaio Corrêa Mariani. FZEA-USP Pirassununga: Mirele Daiana Poleti, Jessika Cristina Chagas Lesbon, Elisangela Chicaroni Mattos, Heidge Fukumasu. USP-Botucatu: Rejane

			Maria Tommasini Grotto, Jayme A. Souza-Neto, Guilherme Targino Valente, Patricia Akemi Assato, Felipe Allan da Silva da Costa, Bianca Cechetto Carlos. Mendelics: Bibiana Santos, João Paulo Kitajima, Erika Freitas, David Schlesinger. Hemocentro Ribeirão Preto: Simone Kashima, Evandra Strazza Rodrigues, Svetoslav Nanev Slavov, Elaine Vieira dos Santos, Rafael dos Santos Bezerra, Luiz Carlos Junior de Alcantara, Marta Giovanetti, Vagner Fonseca, Flavia Aburjaile, Rodrigo Tocantins Calado. FAMERP-SJRP: Cecília Artico Banho, Lívia Sacchetto, Fábio Sossai Possebon, Leila Sabrina Ullmann, Cíntia Bittar, Guilherme Campos, Helena Lage Ferreira, Jorge A. Petroli Marchesi, Maisa C. Pereira Parra, Marília Moraes, Paula Rahal, Paulo Inacio da Costa, João Pessoa Araújo Jr., Maurício Lacerda Nogueira. Prefeitura de Sao Paulo: Melissa Palmieri.
EPI_ISL_1966534	UBS DR HELIO MIGLIARI	Instituto Butantan / FZEA-USP (Pirassununga)	Instituto Butantan: Dimas Tadeu Covas, Sandra Coccuzzo Sampaio, Maria Carolina Elias, José Salvatore Leister Patané, Vincent Louis Viala, Antonio Jorge Martins, Ricardo Haddad, Claudia Renata dos Santos Barros, Elaine Cristina Marqueze, Raul Machado Neto, Debora Botequio Moretti, Jardelina de Souza Todao Bernardino, Loyze Paola Oliveira de Lima, Luiz Aurelio de Campos Crispin. Centro de Genômica Funcional da ESALQ: Luiz Lehmann Coutinho, Ricardo Augusto Brassaloti, Raquel de Lello Rocha Campos Cassano. NGS Soluções Genômicas: Pilar Drummond Sampaio Corrêa Mariani. FZEA-USP Pirassununga: Mirele Daiana Poleti, Jessika Cristina Chagas Lesbon, Elisangela Chicaroni Mattos, Heidge Fukumasu. USP-Botucatu: Rejane Maria Tommasini Grotto, Jayme A. Souza-Neto, Guilherme Targino Valente, Patricia Akemi Assato, Felipe Allan da Silva da Costa, Bianca Cechetto Carlos. Mendelics: Bibiana Santos, João Paulo Kitajima, Erika Freitas, David Schlesinger. Hemocentro Ribeirão Preto: Simone Kashima, Evandra Strazza Rodrigues, Svetoslav Nanev Slavov, Elaine Vieira dos Santos, Rafael dos Santos Bezerra, Luiz Carlos Junior de Alcantara, Marta Giovanetti, Vagner Fonseca, Flavia Aburjaile, Rodrigo Tocantins Calado. FAMERP-SJRP: Cecília Artico Banho, Lívia Sacchetto, Fábio Sossai Possebon, Leila Sabrina Ullmann, Cíntia Bittar, Guilherme Campos, Helena Lage Ferreira, Jorge A. Petroli Marchesi, Maisa C. Pereira Parra, Marília Moraes, Paula Rahal, Paulo Inacio da Costa, João Pessoa Araújo Jr., Maurício Lacerda Nogueira. Prefeitura de Sao Paulo: Melissa Palmieri.
EPI_ISL_1966721	UNIDADE DE PRONTO ATENDIMENTO DE VARZEA PAULISTA UPA II	Instituto Butantan / Mendelics	Instituto Butantan: Dimas Tadeu Covas, Sandra Coccuzzo Sampaio, Maria Carolina Elias, José Salvatore Leister Patané, Vincent Louis Viala, Antonio Jorge Martins, Ricardo Haddad, Claudia Renata dos Santos Barros, Elaine Cristina Marqueze, Raul Machado Neto, Debora Botequio Moretti, Jardelina de Souza Todao Bernardino, Loyze Paola Oliveira de Lima, Luiz Aurelio de Campos Crispin. Centro de Genômica Funcional da ESALQ: Luiz Lehmann Coutinho, Ricardo Augusto Brassaloti, Raquel de Lello Rocha Campos Cassano. NGS Soluções Genômicas: Pilar Drummond Sampaio Corrêa Mariani. FZEA-USP Pirassununga: Mirele Daiana Poleti, Jessika Cristina Chagas Lesbon, Elisangela Chicaroni Mattos, Heidge Fukumasu. USP-Botucatu: Rejane Maria Tommasini Grotto, Jayme A. Souza-Neto, Guilherme Targino Valente, Patricia Akemi Assato, Felipe Allan da Silva da Costa, Bianca Cechetto Carlos. Mendelics: Bibiana Santos, João Paulo Kitajima, Erika Freitas, David Schlesinger. Hemocentro Ribeirão Preto: Simone Kashima, Evandra Strazza Rodrigues, Svetoslav Nanev Slavov, Elaine Vieira dos Santos, Rafael dos Santos Bezerra, Luiz Carlos Junior de Alcantara, Marta Giovanetti, Vagner Fonseca, Flavia Aburjaile, Rodrigo Tocantins Calado. FAMERP-SJRP: Cecília Artico Banho, Lívia Sacchetto, Fábio Sossai Possebon, Leila Sabrina Ullmann, Cíntia Bittar, Guilherme Campos, Helena Lage Ferreira, Jorge A. Petroli Marchesi, Maisa C. Pereira Parra, Marília Moraes, Paula Rahal, Paulo Inacio da Costa, João Pessoa Araújo Jr., Maurício Lacerda Nogueira. Prefeitura de Sao Paulo: Melissa Palmieri.
EPI_ISL_1966827, EPI_ISL_1966829	CENTRO DE SAUDE DR RENATO DE CARVALHO RIBEIRO ANGATUBA	Instituto Butantan / ESALQ-USP (Piracicaba)	Instituto Butantan: Dimas Tadeu Covas, Sandra Coccuzzo Sampaio, Maria Carolina Elias, José Salvatore Leister Patané, Vincent Louis Viala, Antonio Jorge Martins, Ricardo Haddad, Claudia Renata dos Santos Barros, Elaine Cristina Marqueze, Raul Machado Neto, Debora Botequio Moretti, Jardelina de Souza Todao Bernardino, Loyze Paola Oliveira de Lima, Luiz Aurelio de Campos Crispin. Centro de Genômica Funcional da ESALQ: Luiz Lehmann Coutinho, Ricardo Augusto Brassaloti, Raquel de Lello Rocha Campos Cassano. NGS Soluções Genômicas: Pilar Drummond Sampaio Corrêa Mariani. FZEA-USP Pirassununga: Mirele Daiana Poleti, Jessika Cristina Chagas Lesbon, Elisangela Chicaroni Mattos, Heidge Fukumasu. USP-Botucatu: Rejane Maria Tommasini Grotto, Jayme A. Souza-Neto, Guilherme Targino Valente, Patricia Akemi Assato, Felipe Allan da Silva da Costa, Bianca Cechetto Carlos. Mendelics: Bibiana Santos, João Paulo Kitajima, Erika Freitas, David Schlesinger. Hemocentro Ribeirão Preto: Simone Kashima, Evandra Strazza Rodrigues, Svetoslav Nanev Slavov, Elaine Vieira dos Santos, Rafael dos Santos Bezerra, Luiz Carlos Junior de Alcantara, Marta Giovanetti, Vagner Fonseca, Flavia Aburjaile, Rodrigo Tocantins Calado. FAMERP-SJRP: Cecília Artico Banho, Lívia Sacchetto, Fábio Sossai Possebon, Leila Sabrina Ullmann, Cíntia Bittar, Guilherme Campos, Helena Lage Ferreira, Jorge A. Petroli Marchesi, Maisa C. Pereira Parra, Marília Moraes, Paula Rahal, Paulo Inacio da Costa, João Pessoa Araújo Jr., Maurício Lacerda Nogueira. Prefeitura de Sao Paulo: Melissa Palmieri.
EPI_ISL_1966847	UBS SAO JUDAS	Instituto Butantan / ESALQ-USP (Piracicaba)	Instituto Butantan: Dimas Tadeu Covas, Sandra Coccuzzo Sampaio, Maria Carolina Elias, José Salvatore Leister Patané, Vincent Louis Viala, Antonio Jorge Martins, Ricardo Haddad, Claudia Renata dos Santos Barros, Elaine Cristina Marqueze, Raul Machado Neto, Debora Botequio Moretti, Jardelina de Souza Todao Bernardino, Loyze Paola Oliveira de Lima, Luiz Aurelio de Campos Crispin. Centro de Genômica Funcional da ESALQ: Luiz Lehmann Coutinho, Ricardo Augusto Brassaloti, Raquel de Lello Rocha Campos Cassano. NGS Soluções Genômicas: Pilar Drummond Sampaio Corrêa Mariani. FZEA-USP Pirassununga: Mirele Daiana Poleti, Jessika Cristina Chagas Lesbon, Elisangela Chicaroni Mattos, Heidge Fukumasu. USP-Botucatu: Rejane Maria Tommasini Grotto, Jayme A. Souza-Neto, Guilherme Targino Valente, Patricia Akemi Assato, Felipe Allan da Silva da Costa, Bianca Cechetto Carlos. Mendelics: Bibiana Santos, João Paulo Kitajima, Erika Freitas, David Schlesinger. Hemocentro Ribeirão Preto: Simone Kashima, Evandra Strazza Rodrigues, Svetoslav Nanev Slavov, Elaine Vieira dos Santos, Rafael dos Santos Bezerra, Luiz Carlos Junior de Alcantara, Marta Giovanetti, Vagner Fonseca, Flavia Aburjaile, Rodrigo Tocantins Calado. FAMERP-SJRP: Cecília Artico Banho, Lívia Sacchetto, Fábio Sossai Possebon, Leila Sabrina Ullmann, Cíntia Bittar, Guilherme Campos, Helena Lage Ferreira, Jorge A. Petroli Marchesi, Maisa C. Pereira Parra, Marília Moraes, Paula Rahal, Paulo Inacio da Costa, João Pessoa Araújo Jr., Maurício Lacerda Nogueira. Prefeitura de Sao Paulo: Melissa Palmieri.
EPI_ISL_1967169	HOSPITAL DE CAMPANHA COVID 19 MUNICIPIO DE TAUBATE	Instituto Butantan / ESALQ-USP (Piracicaba)	Instituto Butantan: Dimas Tadeu Covas, Sandra Coccuzzo Sampaio, Maria Carolina Elias, José Salvatore Leister Patané, Vincent Louis Viala, Antonio Jorge Martins, Ricardo Haddad, Claudia Renata dos Santos Barros, Elaine Cristina Marqueze, Raul Machado Neto, Debora Botequio Moretti, Jardelina de Souza Todao Bernardino, Loyze Paola Oliveira de Lima, Luiz Aurelio de Campos Crispin. Centro de Genômica Funcional da ESALQ: Luiz Lehmann Coutinho, Ricardo Augusto Brassaloti, Raquel de Lello Rocha Campos Cassano. NGS Soluções Genômicas: Pilar Drummond Sampaio Corrêa Mariani. FZEA-USP Pirassununga: Mirele Daiana Poleti, Jessika Cristina Chagas Lesbon, Elisangela Chicaroni Mattos, Heidge Fukumasu. USP-Botucatu: Rejane Maria Tommasini Grotto, Jayme A. Souza-Neto, Guilherme Targino Valente, Patricia Akemi Assato, Felipe Allan da Silva da Costa, Bianca Cechetto Carlos. Mendelics: Bibiana Santos, João Paulo Kitajima, Erika Freitas, David Schlesinger. Hemocentro Ribeirão Preto: Simone Kashima, Evandra Strazza Rodrigues, Svetoslav Nanev Slavov, Elaine Vieira dos Santos, Rafael dos Santos Bezerra, Luiz Carlos Junior de Alcantara, Marta Giovanetti, Vagner Fonseca, Flavia Aburjaile, Rodrigo Tocantins Calado. FAMERP-SJRP: Cecília Artico Banho, Lívia Sacchetto, Fábio Sossai Possebon, Leila Sabrina Ullmann, Cíntia Bittar, Guilherme Campos, Helena Lage Ferreira, Jorge A. Petroli Marchesi, Maisa C. Pereira Parra, Marília Moraes, Paula Rahal, Paulo Inacio da Costa, João Pessoa Araújo Jr., Maurício Lacerda Nogueira. Prefeitura de Sao Paulo: Melissa Palmieri.
EPI_ISL_1967502, EPI_ISL_1967511, EPI_ISL_1967518, EPI_ISL_1967539	Maryland Genomics, Institute for Genome Sciences, University of Maryland School of Medicine	Maryland Genomics, Institute for Genome Sciences, University of Maryland School of Medicine	Tallon, Luke J; Sadzewicz, Lisa D; Humphrys, Mike; Ott, Sandra; Roussey, Holly; Mehta, Aditya; Vavikolanu, Kranthi; Fraser, Claire M; Ravel, Jacques
EPI_ISL_1969548	Scripps Medical Laboratory	Andersen lab at Scripps Research	SEARCH Alliance San Diego with Michael Quigley, Ellen Stefanski, Ian Mchardy
EPI_ISL_1989610	Medical Laboratories Duesseldorf	Center of Medical Microbiology, Virology, and Hospital Hygiene, University of Duesseldorf	Maximilian Damagnez;Alexander Dilthey;Angelika Helmer;Torsten Houwaart;Lisanna Hülse;Christian Lange;Malte Kohns Vasconcelos;Nadine Lübke;Jessica Nicolai;Klaus Pfeffer;Daniel Strelow;Jörg Timm;Andreas Walker;Tobias Wienemann
EPI_ISL_1999267, EPI_ISL_2000296, EPI_ISL_2000512	Aegis Sciences Corporation	Centers for Disease Control and Prevention Division of Viral Diseases, Pathogen Discovery	Dakota Howard, Dhwani Batra, Peter W. Cook, Kara Moser, Adrian Paskey, Jason Caravas, Benjamin Rambo-Martin, Shatavia Morrison, Christopher Gulvick, Scott Sammons, Yvette Unoarumhi, Darlene Wagner, Matthew Schmerer, Cyndi Clark, Patrick Campbell, Rob Case, Vikramsinha Ghorpade, Holly Houdeshell, Ola Kvalvaag, Dillon Nall, Ethan Sanders, Alec Vest, Shaun Westlund, Matthew Hardison, Clinton R. Paden, Duncan MacCannell
EPI_ISL_2003125, EPI_ISL_2003127	Instituto Adolfo Lutz Central	Instituto Adolfo Lutz, Interdiciplinary Procedures Center, Strategic Laboratory	Claudio Tavares Sacchi, Claudia Regina Gonçalves, Erica Valessa Ramos Gomes, Karoline Rodrigues Campos, Caio Vinicius Dias Lopes, Leonardo Jose Tadeu de Araujo
EPI_ISL_2003938	Hospital of the University of Pennsylvania Molecular Pathology Lab	Bushman Lab - University of Pennsylvania	John Everett, Kyle Rodino, Shantan Reddy, Pascha Hokama, Aoife M. Roche, Young Hwang, Abigail Glascock, Scott Sherrill-Mix, Samantha A. Whiteside, Jevon Graham-Wooten, Layla A. Khatib, Ayannah S. Fitzgerald, Arupa Ganguly, Mike Feldman, Brendan Kelly, Ronald G. Collman and Frederic Bushman
EPI_ISL_2004015	Servicio de Microbiología. Hospital Clínico Universitario de Valencia	SeqCOVID-SPAIN consortium/IBV(CSIC)	David Navarro Ortega, Eliseo Albert Vicent, Ignacio Torres and SeqCOVID-SPAIN consortium
EPI_ISL_2007472, EPI_ISL_2007475	Laboratorio de Virología del Hospital de Niños Dr. Ricardo Gutierrez	Área de Secuenciación del Laboratorio de Virología del Hospital de Niños Dr. Ricardo Gutierrez on behalf of 'Proyecto Argentino Interinstitucional de genomica de SARS-CoV-2' (PAIS Consortium)	Alexay, S; Thomas, G; Medina, C; Labarta, N; Streitenberger, C; Villegas, E; Barreda Frank, M; Grandis, E; Acevedo, ME; Alvarez Lopez, C; Jacques, O; Mistchenko, A; Nabaes Jodar, M; Goya, S; Lusso, S; Acuña, D; Natale, MI; Valinotto, LE; Viegas, M.
EPI_ISL_2007476	Hospital General de Agudos Dr. Cosme Argerich	Área de Secuenciación del Laboratorio de Virología del	Marcia Pozzati, Jésica Galeano, Florencia Rodríguez, Florencia Funez, Andrea Fernández, Karina Polanski; Alexay, S; Nabaes Jodar, M; Acuña, D; Goya,

		Hospital de Niños Dr. Ricardo Gutierrez on behalf of 'Proyecto Argentino Interinstitucional de genomica de SARS-CoV-2' (PAIS Consortium)	S; Lusso, S; Natale, MI; Valinotto, LE; Viegas, M.
EPI_ISL_2007477, EPI_ISL_2007481, EPI_ISL_2007482	Laboratorio de Virología del Hospital de Niños Dr. Ricardo Gutierrez	Área de Secuenciación del Laboratorio de Virología del Hospital de Niños Dr. Ricardo Gutierrez on behalf of 'Proyecto Argentino Interinstitucional de genomica de SARS-CoV-2' (PAIS Consortium)	Alexay, S; Thomas, G; Medina, C; Labarta, N; Streitenberger, C; Villegas, E; Barreda Frank, M; Grandis, E; Acevedo, ME; Alvarez Lopez, C; Jacques, O; Mistchenko, A; Nabaes Jodar, M; Goya, S; Lusso, S; Acuña, D; Natale, MI; Valinotto, LE; Viegas, M.
EPI_ISL_2007486	Hospital de Clínicas "José de San Martín"	Área de Secuenciación del Laboratorio de Virología del Hospital de Niños Dr. Ricardo Gutierrez on behalf of 'Proyecto Argentino Interinstitucional de genomica de SARS-CoV-2' (PAIS Consortium)	Marcelo Rodríguez Fermepin, Dra. Maria Lucia Gallo Vaulet, Analia Patricia Toledano, Alexay, S; Nabaes Jodar, M; Acuña, D; Goya, S; Lusso, S; Natale, MI; Valinotto, LE; Viegas, M.
EPI_ISL_2007488, EPI_ISL_2007490, EPI_ISL_2007520, EPI_ISL_2007527, EPI_ISL_2007528, EPI_ISL_2007529, EPI_ISL_2007530, EPI_ISL_2007531	Laboratorio de Virología del Hospital de Niños Dr. Ricardo Gutierrez	Área de Secuenciación del Laboratorio de Virología del Hospital de Niños Dr. Ricardo Gutierrez on behalf of 'Proyecto Argentino Interinstitucional de genomica de SARS-CoV-2' (PAIS Consortium)	Alexay, S; Thomas, G; Medina, C; Labarta, N; Streitenberger, C; Villegas, E; Barreda Frank, M; Grandis, E; Acevedo, ME; Alvarez Lopez, C; Jacques, O; Mistchenko, A; Nabaes Jodar, M; Goya, S; Lusso, S; Acuña, D; Natale, MI; Valinotto, LE; Viegas, M.
EPI_ISL_2007532	Hospital General de Agudos Dr. Cosme Argerich	Área de Secuenciación del Laboratorio de Virología del Hospital de Niños Dr. Ricardo Gutierrez on behalf of 'Proyecto Argentino Interinstitucional de genomica de SARS-CoV-2' (PAIS Consortium)	Marcia Pozzati, Jésica Galeano, Florencia Rodríguez, Florencia Funez, Andrea Fernández, Karina Polanski; Alexay, S; Nabaes Jodar, M; Acuña, D; Goya, S; Lusso, S; Natale, MI; Valinotto, LE; Viegas, M.
EPI_ISL_2007533, EPI_ISL_2007534, EPI_ISL_2007535, EPI_ISL_2007549	Laboratorio de Virología del Hospital de Niños Dr. Ricardo Gutierrez	Área de Secuenciación del Laboratorio de Virología del Hospital de Niños Dr. Ricardo Gutierrez on behalf of 'Proyecto Argentino Interinstitucional de genomica de SARS-CoV-2' (PAIS Consortium)	Alexay, S; Thomas, G; Medina, C; Labarta, N; Streitenberger, C; Villegas, E; Barreda Frank, M; Grandis, E; Acevedo, ME; Alvarez Lopez, C; Jacques, O; Mistchenko, A; Nabaes Jodar, M; Goya, S; Lusso, S; Acuña, D; Natale, MI; Valinotto, LE; Viegas, M.
EPI_ISL_2009287	Genetica Molecular and Subdepartamento de Virologia ISP Chile	Instituto de Salud Publica de Chile	Javier Tognarelli, Karen Orostica, Barbara Parra, Loredana Arata, Gisselle Barra, Patricia Bustos, Rodrigo Fasce, Andres Castillo, Soledad Ulloa, Jorge Fernandez
EPI_ISL_2009549	Genetica Molecular and Subdepartamento de Virologia ISP Chile	Instituto de Salud Publica de Chile	Karen Orostica, Constanza Campano, Barbara Parra, Loredana Arata, Gisselle Barra, Patricia Bustos, Rodrigo Fasce, Javier Tognarelli, Andres Castillo, Soledad Ulloa, Jorge Fernandez
EPI_ISL_2009613, EPI_ISL_2009614, EPI_ISL_2009615	Genetica Molecular and Subdepartamento de Virologia ISP Chile	Instituto de Salud Publica de Chile	Javier Tognarelli, Karen Orostica, Barbara Parra, Loredana Arata, Gisselle Barra, Patricia Bustos, Rodrigo Fasce, Andres Castillo, Soledad Ulloa, Jorge Fernandez
EPI_ISL_2009725	Helix/Illumina	Centers for Disease Control and Prevention Division of Viral Diseases, Pathogen Discovery	Dakota Howard, Dhwani Batra, Peter W. Cook, Kara Moser, Adrian Paskey, Jason Caravas, Benjamin Rambo-Martin, Shatavia Morrison, Christopher Gulvick, Scott Sammons, Yvette Unoarumhi, Darlene Wagner, Matthew Schmerer, Eileen de Feo, Jan Antico, Christine Tran, Matthew Tolentino, Shannon Wickline, Kim Gietzen, Brad Sickler, Jingtao Liu, Eric Allen, Phil Febbo, Nicole L. Washington, Simon White, Geraint Levan, Kelly Schiabor Barrett, Elizabeth Cirulli, Alexandre Bolze, Ary Ascencio, Charlotte Rivera-Garcia, Ryan Cho, Jason Nguyen, Sherry Wang, Jimmy Ramirez, Tyler Cassens, Efren Sandoval, Magnus Isaksson, William Lee, David Becker, Marc Laurent, James Lu, Clinton R. Paden, Duncan MacCannell
EPI_ISL_2020105, EPI_ISL_2020179	HOSPITAL UNIVERSITARIO SON ESPASES	HOSPITAL UNIVERSITARIO SON ESPASES	Carla López-Causapé, Pablo Fraile-Ribot, Antonio Oliver, SeqCovid
EPI_ISL_2022037	Lighthouse Lab in Milton Keynes	Wellcome Sanger Institute for the COVID-19 Genomics UK (COG-UK) Consortium	The Lighthouse Lab in Milton Keynes and Alex Alderton, Roberto Amato, Jeffrey Barrett, Sonia Goncalves, Ewan Harrison, David K. Jackson, Ian Johnston, Dominic Kwiatkowski, Cordelia Langford, John Sillitoe on behalf of the Wellcome Sanger Institute COVID-19 Surveillance Team
EPI_ISL_2029739	Labo Analyses Med	National Reference Center for Viruses of Respiratory Infections, Institut Pasteur, Paris	Marion Barbet, Sylvie Behillil, Méline Bizard, Angela Brisebarre, Camille Capel, Vincent Enouf, Louise Lefrançois, Frédéric Lemoine, Christophe Malabat, Corinne Maufrais, Pierre Lechat, Etienne Simon-Lorière, Maud Vanpeene, Sylvie Van der Werf ,Arditi Cocco
EPI_ISL_2031725, EPI_ISL_2031739	Centro de Innovación en Vigilancia Epidemiológica (CiVE), Institut Pasteur Montevideo, Uruguay	Centro de Innovación en Vigilancia Epidemiológica (CiVE), Institut Pasteur Montevideo, Uruguay	Natalia Rego, Alicia Costábile, Mercedes Paz, Cecilia Salazar, Paula Perbolianachis, Tamara Fernández, Ignacio Ferrés, Rodrigo Arce, Alvaro Fajardo, Mailen Arleo, Tania Possi, Inés Bellini, Lucia Bilbao, Natalia Reyes, Ma Noel Bentancor, Andrés Lizosaín, María José Benítez, Odhille Chappos, Melissa Duquía, Belén González, Luciana Griffero, Mauricio Méndez, Ma Pía Techera, Juan Zanetti, Bernardina Rivera, Matías Maidana, Martina Alonso, Cecilia Alonso, Julio Medina, Henry Albornoz, Rodney Colina, Gregorio Iraola, Lucía Spangenberg, Gonzalo Moratorio, Pilar Moreno
EPI_ISL_2037442	Instituto de Virologia "Dr. J. M. Vanella", Facultad de Ciencias Médicas, Universidad Nacional de Córdoba.	Centro de Investigaciones Agropecuarias (CIAP), Instituto Nacional de Tecnologia Agropecuaria (INTA) Cordoba, Argentina, on behalf of 'Proyecto Argentino Interinstitucional de Genomica de SARS-CoV-2 (PAIS Consortium)	Brenda Konigheim, Lorena Spinsanti, Adrian Diaz, Javier Aguilar, Sebastian Blanco, Mauricio Beranek, Maria Elisa Rivarola, Maria Belen Pisano, Viviana Re, Gonzalo Castro, Gabriela Barbas, Franco Fernandez, Humberto Debat, Nathalie Marquez, Sandra Gallego.
EPI_ISL_2038926, EPI_ISL_2038927, EPI_ISL_2038928, EPI_ISL_2038929, EPI_ISL_2038931, EPI_ISL_2038935, EPI_ISL_2038937, EPI_ISL_2038938, EPI_ISL_2038939, EPI_ISL_2038941, EPI_ISL_2038942, EPI_ISL_2038945, EPI_ISL_2038946, EPI_ISL_2038947, EPI_ISL_2038948, EPI_ISL_2038949, EPI_ISL_2038950, EPI_ISL_2038951			
see above	Laboratorio Central de Saude Publica do Estado de Santa Catarina (LACEN-SC)	Laboratory of Respiratory Viruses and Measles, Oswaldo Cruz Institute, FIOCRUZ	Paola Resende, Luciana Appolinario, Fernando Motta, Anna Carolina Paixao, Ana Carolina Mendonca, Alice Sampaio Rocha, Taina Venas, Elisa Cavalcante Pereira, Renata Serrano Lopes, Darcita Buerger Rovaris, Sandra Bianchini Fernandes, Marilda Siqueira on behalf of the Fiocruz COVID-19 Genomic Surveillance Network
EPI_ISL_2038955	Laboratorio Central de Saude Publica do Estado do Parana (LACEN-PR)	Laboratory of Respiratory Viruses and Measles, Oswaldo Cruz Institute, FIOCRUZ	Paola Resende, Luciana Appolinario, Fernando Motta, Anna Carolina Paixao, Ana Carolina Mendonca, Alice Sampaio Rocha, Taina Venas, Elisa Cavalcante Pereira, Renata Serrano Lopes, Irina Riediger, Marilda Siqueira on behalf of the Fiocruz COVID-19 Genomic Surveillance Network
EPI_ISL_2038958, EPI_ISL_2038959, EPI_ISL_2038960	Laboratorio Central de Saude Publica do Estado do Rio Grande do Sul (LACEN-RS)	Laboratory of Respiratory Viruses and Measles, Oswaldo Cruz Institute, FIOCRUZ	Paola Resende, Luciana Appolinario, Fernando Motta, Anna Carolina Paixao, Ana Carolina Mendonca, Alice Sampaio Rocha, Taina Venas, Elisa Cavalcante Pereira, Renata Serrano Lopes, Tatiana Schaffer Gregianini, Richard Salvato, Marilda Siqueira on behalf of the Fiocruz COVID-19 Genomic Surveillance Network
EPI_ISL_2038961, EPI_ISL_2038963	Laboratory of Respiratory Viruses and Measles, Oswaldo Cruz Institute, FIOCRUZ	Laboratory of Respiratory Viruses and Measles, Oswaldo Cruz Institute, FIOCRUZ	Paola Resende, Luciana Appolinario, Fernando Motta, Anna Carolina Paixao, Ana Carolina Mendonca, Alice Sampaio Rocha, Taina Venas, Elisa Cavalcante Pereira, Renata Serrano Lopes, Marilda Siqueira on behalf of the Fiocruz COVID-19 Genomic Surveillance Network
EPI_ISL_2038965	Laboratorio Central de Saude Publica do Estado de Minas Gerais (LACEN-MG)	Laboratory of Respiratory Viruses and Measles, Oswaldo Cruz Institute, FIOCRUZ	Paola Resende, Luciana Appolinario, Fernando Motta, Anna Carolina Paixao, Ana Carolina Mendonca, Alice Sampaio Rocha, Taina Venas, Elisa Cavalcante Pereira, Renata Serrano Lopes, Andre Felipe Leal Bernardes, Marilda Siqueira on behalf of the Fiocruz COVID-19 Genomic Surveillance Network
EPI_ISL_2038966, EPI_ISL_2038967	Laboratorio Central de Saude Publica do Estado de Santa Catarina (LACEN-SC)	Laboratory of Respiratory Viruses and Measles, Oswaldo Cruz Institute, FIOCRUZ	Paola Resende, Luciana Appolinario, Fernando Motta, Anna Carolina Paixao, Ana Carolina Mendonca, Alice Sampaio Rocha, Taina Venas, Elisa Cavalcante Pereira, Renata Serrano Lopes, Darcita Buerger Rovaris, Sandra Bianchini Fernandes, Marilda Siqueira on behalf of the Fiocruz COVID-19 Genomic Surveillance Network
EPI_ISL_2038968	Laboratorio Central de Saude Publica do Estado do Espitito Santo (LACEN-ES)	Laboratory of Respiratory Viruses and Measles, Oswaldo Cruz Institute, FIOCRUZ	Paola Resende, Luciana Appolinario, Fernando Motta, Anna Carolina Paixao, Ana Carolina Mendonca, Alice Sampaio Rocha, Renata Serrano Lopes, Rodrigo Ribeiro Rodrigues, Marilda Siqueira on behalf of the Fiocruz COVID-19 Genomic Surveillance Network
EPI_ISL_2042220	Aegis Sciences Corporation	Centers for Disease Control and Prevention Division of Viral Diseases, Pathogen Discovery	Dakota Howard, Dhwani Batra, Peter W. Cook, Kara Moser, Adrian Paskey, Jason Caravas, Benjamin Rambo-Martin, Shatavia Morrison, Christopher Gulvick, Scott Sammons, Yvette Unoarumhi, Darlene Wagner, Matthew Schmerer, Cyndi Clark, Patrick Campbell, Rob Case, Vikramsinha Ghorpade, Holly Houdeshell, Ola Kvalvaag, Dillon Nall, Ethan Sanders, Alec Vest, Shaun Westlund, Matthew Hardison, Clinton R. Paden, Duncan MacCannell
EPI_ISL_2080492	POOLE MICROBIOLOGY LABORATORY	COVID-19 Genomics UK (COG-UK) Consortium	PHE Covid Sequencing Team
EPI_ISL_2086594, EPI_ISL_2086596, EPI_ISL_2086605, EPI_ISL_2086616	LABCOVID_HCPA	LABRESIS_HCPA	Wink PL, Martins AF, Volpato F, Monteiro F, Zavascki AP, Barth AL
EPI_ISL_2089479	Aegis Sciences Corporation	Centers for Disease Control and Prevention Division of Viral	Dakota Howard, Dhwani Batra, Peter W. Cook, Kara Moser, Adrian Paskey, Jason Caravas, Benjamin Rambo-Martin, Shatavia Morrison, Christopher

		Diseases, Pathogen Discovery	Gulvick, Scott Sammons, Yvette Unoarumhi, Darlene Wagner, Matthew Schmerer, Cyndi Clark, Patrick Campbell, Rob Case, Vikramsinha Ghorpade, Holly Houdeshell, Ola Kvalvaag, Dillon Nall, Ethan Sanders, Alec Vest, Shaun Westlund, Matthew Hardison, Clinton R. Paden, Duncan MacCannell
EPI_ISL_2097314	WESTCHESTER MEDICAL CENTER	Wadsworth Center, New York State Department of Health	Kirsten St. George, Daryl M. Lamson, Alexis Russell, Matthew Shudt, Melissa A Leisner, Jonathan Plitnick, Catharine Prussing, Navjot Singh, John Kelly, Erasmus Schneider, Erica Lasek-Nesselquist
EPI_ISL_2100319	HOSPITAL UNIVERSITARIO SON ESPASES	HOSPITAL UNIVERSITARIO SON ESPASES	Carla López-Causapé, Pablo Fraile-Ribot, Antonio Oliver, SeqCovid
EPI_ISL_2101463	Unidade de apoio ao diagnóstico da COVID - UNADIG	Bioinformatics Laboratory / LNCC	Luiz G P de Almeida, Alessandra P Lamarca, Ronaldo da Silva F Jr, Liliane Cavalcante, Alexandra L Gerber, Ana Paula de C Guimaraes, Douglas Terra Machado, Cassia Alves, Diana Mariani, Cintia Policarpo, Gleidson da Silva de Oliveira, Mario Sergio Ribeiro, Silvia Carvalho, Flavio Dias da Silva, Marcio Henrique de Oliveira Garcia, Leandro Magalhaes de Souza, Cristiane Gomes da Silva, Caio Luiz Pereira Ribeiro, Andrea Cony Cavalcanti, Claudia Maria Braga de Mello, Amilcar Tanuri, Ana Tereza R Vasconcelos
EPI_ISL_2101561	Laboratorio Central Noel Nutels	Bioinformatics Laboratory / LNCC	Luiz G P de Almeida, Alessandra P Lamarca, Ronaldo da Silva F Jr, Liliane Cavalcante, Alexandra L Gerber, Ana Paula de C Guimaraes, Douglas Terra Machado, Cassia Alves, Diana Mariani, Cintia Policarpo, Gleidson da Silva de Oliveira, Mario Sergio Ribeiro, Silvia Carvalho, Flavio Dias da Silva, Marcio Henrique de Oliveira Garcia, Leandro Magalhaes de Souza, Cristiane Gomes da Silva, Caio Luiz Pereira Ribeiro, Andrea Cony Cavalcanti, Claudia Maria Braga de Mello, Amilcar Tanuri, Ana Tereza R Vasconcelos
EPI_ISL_2101566, EPI_ISL_2101572	Unidade de apoio ao diagnóstico da COVID - UNADIG	Bioinformatics Laboratory / LNCC	Luiz G P de Almeida, Alessandra P Lamarca, Ronaldo da Silva F Jr, Liliane Cavalcante, Alexandra L Gerber, Ana Paula de C Guimaraes, Douglas Terra Machado, Cassia Alves, Diana Mariani, Cintia Policarpo, Gleidson da Silva de Oliveira, Mario Sergio Ribeiro, Silvia Carvalho, Flavio Dias da Silva, Marcio Henrique de Oliveira Garcia, Leandro Magalhaes de Souza, Cristiane Gomes da Silva, Caio Luiz Pereira Ribeiro, Andrea Cony Cavalcanti, Claudia Maria Braga de Mello, Amilcar Tanuri, Ana Tereza R Vasconcelos
EPI_ISL_2101689, EPI_ISL_2101709	Laboratorio Central Noel Nutels	Bioinformatics Laboratory / LNCC	Luiz G P de Almeida, Alessandra P Lamarca, Ronaldo da Silva F Jr, Liliane Cavalcante, Alexandra L Gerber, Ana Paula de C Guimaraes, Douglas Terra Machado, Cassia Alves, Diana Mariani, Cintia Policarpo, Gleidson da Silva de Oliveira, Mario Sergio Ribeiro, Silvia Carvalho, Flavio Dias da Silva, Marcio Henrique de Oliveira Garcia, Leandro Magalhaes de Souza, Cristiane Gomes da Silva, Caio Luiz Pereira Ribeiro, Andrea Cony Cavalcanti, Claudia Maria Braga de Mello, Amilcar Tanuri, Ana Tereza R Vasconcelos
EPI_ISL_2104823, EPI_ISL_2105572, EPI_ISL_2105574	Servicio Virosis Respiratorias-Departamento Virología-INEI	Instituto Nacional Enfermedades Infecciosas C.G.Malbran	Baumeister E., Avaro M., Benedetti E., Russo M., Dattero ME, Pontoriero A., Cisterna D., Molina V., Perandones C., Tuduri E., Lorenzo F., Poklepovich T., Campos J.
EPI_ISL_2105689	LESP Quintana Roo	Instituto de Diagnostico y Referencia Epidemiologicos (INDRE)	Claudia Wong-Arambula, Abril Rodriguez-Maldonado, Vanessa Rivero-Arredondo, Ariadna Medina-Benitez, Joaquin Quiroz-Mercado, Sergio Rangel-Guerrero, Natividad Cruz-Ortiz, Tatiana Nunez-Garcia, Gisela Barrera-Badillo, Lucia Hernandez-Rivas, Irma Lopez-Martinez, Ernesto Ramirez-Gonzalez.
EPI_ISL_2118938	Lighthouse Lab in Milton Keynes	Wellcome Sanger Institute for the COVID-19 Genomics UK (COG-UK) Consortium	The Lighthouse Lab in Milton Keynes and Alex Alderton, Roberto Amato, Jeffrey Barrett, Sonia Goncalves, Ewan Harrison, David K. Jackson, Ian Johnston, Dominic Kwiatkowski, Cordelia Langford, John Sillitoe on behalf of the Wellcome Sanger Institute COVID-19 Surveillance Team
EPI_ISL_2127876	Wales Specialist Virology Centre Sequencing lab: Pathogen Genomics Unit	Public Health Wales Microbiology Cardiff Wales Specialist Virology Centre	Catherine Moore, Johnathan Evans, Laura Gifford, Malorie Perry, Simon Cottrell, Angela Marchbank, Alec Birchley, Alexander Adams, Amy Gaskin, Bree Gatica-Wilcox, Jason Coombes, Joel Southgate, Lauren Gilbert, Lee Graham, Nicole Pacchiarini, Sara Kumziene-Summerhayes, Sarah Taylor, Sophie Jones, Sara Rey, Matthew Bull, Joanne Watkins, Sally Corden, Tom Connor
EPI_ISL_2135142, EPI_ISL_2135152, EPI_ISL_2135156, EPI_ISL_2135158, EPI_ISL_2135162, EPI_ISL_2135168, EPI_ISL_2135171, EPI_ISL_2135172, EPI_ISL_2135254, EPI_ISL_2135265, EPI_ISL_2135267, EPI_ISL_2135269, EPI_ISL_2135271, EPI_ISL_2135280, EPI_ISL_2135281, EPI_ISL_2135302, EPI_ISL_2135331, EPI_ISL_2135335, EPI_ISL_2135339, EPI_ISL_2135685, EPI_ISL_2135688, EPI_ISL_2135692, EPI_ISL_2135693, EPI_ISL_2135694, EPI_ISL_2135700, EPI_ISL_2135702, EPI_ISL_2135703, EPI_ISL_2135705, EPI_ISL_2135706, EPI_ISL_2135709, EPI_ISL_2135713, EPI_ISL_2135715, EPI_ISL_2135716, EPI_ISL_2135717, EPI_ISL_2135718, EPI_ISL_2135719, EPI_ISL_2135720, EPI_ISL_2135726, EPI_ISL_2135988, EPI_ISL_2135989, EPI_ISL_2135990, EPI_ISL_2135991, EPI_ISL_2135992, EPI_ISL_2135993, EPI_ISL_2135994, EPI_ISL_2135999, EPI_ISL_2136000, EPI_ISL_2136006, EPI_ISL_2136011, EPI_ISL_2136012, EPI_ISL_2136013, EPI_ISL_2136014, EPI_ISL_2136015, EPI_ISL_2136029, EPI_ISL_2136043, EPI_ISL_2136051, EPI_ISL_2136059, EPI_ISL_2136060, EPI_ISL_2136061, EPI_ISL_2136063, EPI_ISL_2136065, EPI_ISL_2136066, EPI_ISL_2136067, EPI_ISL_2136077, EPI_ISL_2136090, EPI_ISL_2136094, EPI_ISL_2136097, EPI_ISL_2136099, EPI_ISL_2136100, EPI_ISL_2136101, EPI_ISL_2136102, EPI_ISL_2136103, EPI_ISL_2136105, EPI_ISL_2136107, EPI_ISL_2136110, EPI_ISL_2136112, EPI_ISL_2136113, EPI_ISL_2136114, EPI_ISL_2136115, EPI_ISL_2136116, EPI_ISL_2136117, EPI_ISL_2136119, EPI_ISL_2136120, EPI_ISL_2136122, EPI_ISL_2136124, EPI_ISL_2136125, EPI_ISL_2136126, EPI_ISL_2136127, EPI_ISL_2136128, EPI_ISL_2136130, EPI_ISL_2136132, EPI_ISL_2136133, EPI_ISL_2136134, EPI_ISL_2136136, EPI_ISL_2136137, EPI_ISL_2136141, EPI_ISL_2136143, EPI_ISL_2136153, EPI_ISL_2136155, EPI_ISL_2136160, EPI_ISL_2136168, EPI_ISL_2136175, EPI_ISL_2136176, EPI_ISL_2136177, EPI_ISL_2136182			
see above	Servicio Virosis Respiratorias-Departamento Virología-INEI	Instituto Nacional Enfermedades Infecciosas C.G.Malbran	Baumeister E., Avaro M., Benedetti E., Russo M., Dattero ME, Pontoriero A., Cisterna D., Molina V., Perandones C., Tuduri E., Lorenzo F., Poklepovich T., Campos J.
EPI_ISL_2139543	Laboratorio Exame	Universidade Federal de Ciencias da Saude de Porto Alegre	Vinícius Bonetti Franceschi, Gabriel Dickin Caldana et al.
EPI_ISL_2140039, EPI_ISL_2140040, EPI_ISL_2140041, EPI_ISL_2140042, EPI_ISL_2140044, EPI_ISL_2140045, EPI_ISL_2140046, EPI_ISL_2140047, EPI_ISL_2140052, EPI_ISL_2140054, EPI_ISL_2140055, EPI_ISL_2140057, EPI_ISL_2140060, EPI_ISL_2140066, EPI_ISL_2140069, EPI_ISL_2140073, EPI_ISL_2140075, EPI_ISL_2140077, EPI_ISL_2140078, EPI_ISL_2140079, EPI_ISL_2140080, EPI_ISL_2140082, EPI_ISL_2140083, EPI_ISL_2140084, EPI_ISL_2140085, EPI_ISL_2140086, EPI_ISL_2140087, EPI_ISL_2140093, EPI_ISL_2140096, EPI_ISL_2140097, EPI_ISL_2140098, EPI_ISL_2140101, EPI_ISL_2140106, EPI_ISL_2140108, EPI_ISL_2140109, EPI_ISL_2140112, EPI_ISL_2140113, EPI_ISL_2140116, EPI_ISL_2140133, EPI_ISL_2140135, EPI_ISL_2140136, EPI_ISL_2140143, EPI_ISL_2140144, EPI_ISL_2140145, EPI_ISL_2140146			
see above	Servicio Virosis Respiratorias-Departamento Virología-INEI	Instituto Nacional Enfermedades Infecciosas C.G.Malbran	Baumeister E., Avaro M., Benedetti E., Russo M., Dattero ME, Pontoriero A., Cisterna D., Molina V., Perandones C., Tuduri E., Lorenzo F., Poklepovich T., Campos J.
EPI_ISL_2141201	Public Health Ontario Laboratory	Public Health Ontario Laboratory	Vanessa G Allen, Philip Banh, Yao Chen, Richard de Borja, Alireza Eshaghi, Nahuel Fittipaldi, Christine Frantz, Jonathan B Gubbay, Jennifer L Guthrie, Lawrence Heisler, Esha Joshi, Michael Laszloffy, Aimin Li, Michael CY Li, Dean Maxwell, Sandeep Nagra, Samir N Patel, Jared Simpson, Karthikeyan Sivaraman, Ashleigh Sullivan, Yogi Sundaravadanam, Sarah Teatero, Andre Villegas, Matthew Watson, Sandra Zittermann
EPI_ISL_2148239, EPI_ISL_2149333, EPI_ISL_2149527, EPI_ISL_2150898	Aegis Sciences Corporation	Centers for Disease Control and Prevention Division of Viral Diseases, Pathogen Discovery	Dakota Howard, Dhwani Batra, Peter W. Cook, Kara Moser, Adrian Paskey, Jason Caravas, Benjamin Rambo-Martin, Shatavia Morrison, Christopher Gulvick, Scott Sammons, Yvette Unoarumhi, Darlene Wagner, Matthew Schmerer, Cyndi Clark, Patrick Campbell, Rob Case, Vikramsinha Ghorpade, Holly Houdeshell, Ola Kvalvaag, Dillon Nall, Ethan Sanders, Alec Vest, Shaun Westlund, Matthew Hardison, Clinton R. Paden, Duncan MacCannell
EPI_ISL_2151882	Biogroup Bio Lam-LCD Saint-Denis	Department of Virology, Henri Mondor University Hospital, Assistance Publique Hôpitaux de Paris, Université Paris-Est Créteil, INSERM U955	Christophe Rodriguez, Slim Fourati, Vanessa Demontant, Guillaume Gricourt, Melissa N'Debi, Alexandre Soulier, Elisabeth Trawinski, Jean-Michel Pawlotsky
EPI_ISL_2157208	Laboratorio de Salud Pública	Gencore - Universidad de los Andes	Marcela Guevara, Luisa Sacristan, David Gonzalez, Silvia Restrepo, Johana Hernandez, Gabriela Delgado, Alejandro Gomez
EPI_ISL_2157389	Laboratorio Central de Saude Publica do Estado de Sergipe (LACEN/SE)	Laboratory of Respiratory Viruses and Measles, Oswaldo Cruz Institute, FIOCRUZ	Paola Resende, Luciana Appolinario, Fernando Motta, Anna Carolina Paixao, Ana Carolina Mendonca, Alice Sampaio Rocha, Tainá Moreira Martins Venas, Elisa Cavalcante Pereira, Renata Serrano Lopes, Cliomar Alves dos Santos, Marilda Siqueira on behalf of the Fiocruz COVID-19 Genomic Surveillance Network
EPI_ISL_2157396, EPI_ISL_2157399	Laboratório Central de Saude Publica do Estado de Santa Catarina (LACEN/SC)	Laboratory of Respiratory Viruses and Measles, Oswaldo Cruz Institute, FIOCRUZ	Paola Resende, Luciana Appolinario, Fernando Motta, Anna Carolina Paixao, Ana Carolina Mendonca, Alice Sampaio Rocha, Taina Venas, Elisa Cavalcante Pereira, Renata Serrano Lopes, Darcita Buerger Rovaris, Sandra Bianchini Fernandes, Marilda Siqueira on behalf of the Fiocruz COVID-19 Genomic Surveillance Network
EPI_ISL_2157408, EPI_ISL_2157421	Lboratorio Central de Saude Publica do Estado do Parana (LACEN/PR)	Laboratory of Respiratory Viruses and Measles, Oswaldo Cruz Institute, FIOCRUZ	Paola Resende, Luciana Appolinario, Fernando Motta, Anna Carolina Paixao, Ana Carolina Mendonca, Alice Sampaio Rocha, Taina Venas, Elisa Cavalcante Pereira, Renata Serrano Lopes, Irina Riediger, Marilda Siqueira on behalf of the Fiocruz COVID-19 Genomic Surveillance Network
EPI_ISL_2157447	Laboratório Central de Saude Publica do Estado de Santa Catarina (LACEN/SC)	Laboratory of Respiratory Viruses and Measles, Oswaldo Cruz Institute, FIOCRUZ	Paola Resende, Luciana Appolinario, Fernando Motta, Anna Carolina Paixao, Ana Carolina Mendonca, Alice Sampaio Rocha, Taina Venas, Elisa Cavalcante Pereira, Renata Serrano Lopes, Darcita Buerger Rovaris, Sandra Bianchini Fernandes, Marilda Siqueira on behalf of the Fiocruz COVID-19 Genomic Surveillance Network
EPI_ISL_2157488	Lboratorio Central de Saude Publica do Estado do Parana (LACEN/PR)	Laboratory of Respiratory Viruses and Measles, Oswaldo Cruz Institute, FIOCRUZ	Paola Resende, Luciana Appolinario, Fernando Motta, Anna Carolina Paixao, Ana Carolina Mendonca, Alice Sampaio Rocha, Taina Venas, Elisa Cavalcante Pereira, Renata Serrano Lopes, Irina Riediger, Marilda Siqueira on behalf of the Fiocruz COVID-19 Genomic Surveillance Network
EPI_ISL_2157499	Laboratório Central de Saude Publica do Estado de Santa Catarina (LACEN/SC)	Laboratory of Respiratory Viruses and Measles, Oswaldo Cruz Institute, FIOCRUZ	Paola Resende, Luciana Appolinario, Fernando Motta, Anna Carolina Paixao, Ana Carolina Mendonca, Alice Sampaio Rocha, Taina Venas, Elisa Cavalcante Pereira, Renata Serrano Lopes, Darcita Buerger Rovaris, Sandra Bianchini Fernandes, Marilda Siqueira on behalf of the Fiocruz COVID-19 Genomic Surveillance Network
EPI_ISL_2157541	Laboratorio Central de Saude Publica do Esatado de Alagoas (LACEN/AL)	Laboratory of Respiratory Viruses and Measles, Oswaldo Cruz Institute, FIOCRUZ	Paola Resende, Luciana Appolinario, Fernando Motta, Anna Carolina Paixao, Ana Carolina Mendonca, Alice Sampaio Rocha, Taina Venas, Elisa Cavalcante Pereira, Renata Serrano Lopes, Anderson Brandao Leite, Marilda Siqueira on behalf of the Fiocruz COVID-19 Genomic Surveillance Network

EPI_ISL_2158695, EPI_ISL_2158697, EPI_ISL_2158700, EPI_ISL_2158701, EPI_ISL_2158703, EPI_ISL_2158704, EPI_ISL_2158705, EPI_ISL_2158706, EPI_ISL_2158709, EPI_ISL_2158710, EPI_ISL_2158720, EPI_ISL_2158730, EPI_ISL_2158734, EPI_ISL_2158735, EPI_ISL_2158737, EPI_ISL_2158738, EPI_ISL_2158740, EPI_ISL_2158741, EPI_ISL_2158742, EPI_ISL_2158746, EPI_ISL_2158747, EPI_ISL_2158748, EPI_ISL_2158750, EPI_ISL_2158754, EPI_ISL_2158755, EPI_ISL_2158756, EPI_ISL_2158764, EPI_ISL_2158765, EPI_ISL_2158766, EPI_ISL_2158767, EPI_ISL_2158769, EPI_ISL_2158770, EPI_ISL_2158772, EPI_ISL_2158776, EPI_ISL_2158777, EPI_ISL_2158778, EPI_ISL_2158781, EPI_ISL_2158782, EPI_ISL_2158783, EPI_ISL_2158785, EPI_ISL_2158787, EPI_ISL_2158794, EPI_ISL_2158796, EPI_ISL_2158797, EPI_ISL_2158798, EPI_ISL_2158799, EPI_ISL_2158803, EPI_ISL_2158813, EPI_ISL_2158814, EPI_ISL_2158817, EPI_ISL_2158818, EPI_ISL_2158824, EPI_ISL_2158825, EPI_ISL_2158827, EPI_ISL_2158828, EPI_ISL_2158829, EPI_ISL_2158831, EPI_ISL_2158834, EPI_ISL_2158840, EPI_ISL_2158843, EPI_ISL_2158844, EPI_ISL_2158845, EPI_ISL_2158847, EPI_ISL_2158849			
see above	Servicio Virosis Respiratorias-Departamento Virología-INEI	Instituto Nacional Enfermedades Infecciosas C.G.Malbran	Baumeister E., Avaro M., Benedetti E., Russo M., Dattero ME, Pontoriero A., Cisterna D., Molina V., Perandones C., Tuduri E., Lorenzo F., Poklepovich T., Campos J.
EPI_ISL_2177509	TXDSHS	TXDSHS	Rashmi Tuladhar, Bonnie Oh, Jenny Zhang, Maliha Rahman, Mayela Pedrueza, Anita Pokharel, Karen Bobier, Lorraine Rodriguez, Myong Koag, Chun Wang, Rachel Lee, Grace Kubin
EPI_ISL_2178659	Minnesota Department of Health, Public Health Laboratory	Minnesota Department of Health, Public Health Laboratory	Alexandra Lorentz, Jacob Garfin, Matt Plumb, and Xiong Wang
EPI_ISL_2187256	Quest Diagnostics Incorporated	Centers for Disease Control and Prevention Division of Viral Diseases, Pathogen Discovery	Dakota Howard, Dhwani Batra, Peter W. Cook, Kara Moser, Adrian Paskey, Jason Caravas, Benjamin Rambo-Martin, Shatavia Morrison, Christopher Gulvick, Scott Sammons, Yvette Unoarumhi, Darlene Wagner, Matthew Schmerer, S. H. Rosenthal, A. Gerasimova, R. M. Kagan, B. Anderson, M. Hua, Y. Liu, L.E. Bernstein, K.E. Livingston, A. Perez, I. A. Shlyakhter, R. V. Rolando, R. Owen, P. Tanpaiboon, F. Lacbawan, Clinton R. Paden, Duncan MacCannell
EPI_ISL_2187734, EPI_ISL_2187754, EPI_ISL_2187764, EPI_ISL_2187775, EPI_ISL_2187852, EPI_ISL_2187928	HLAGYN - Laboratorio de Imunologia de Transplantes de Goias	HLAGYN - Laboratorio de Imunologia de Transplantes de Goias	Fernando Antonio Vinhal dos Santos, Erika Lopes Rocha Batista, Alessandro Leonardo Alvares Magalhaes, Frederico Rodrigues Vinhal, Sabrina Sara Moreira Duarte, Lucas Carlos Gomes Pereira, Daniel Ferreira de Sousa
EPI_ISL_2191860, EPI_ISL_2191862	Arcispedale Santa Maria Nuova, Autoimmunità, Allergologia e Biotecnologie Innovative	Istituto Zooprofilattico Sperimentale della Lombardia e dell'Emilia Romagna (IZSLER), Risk Analysis and Genomic Epidemiology Unit	Alessandro Zerbini, Lucia Belloni, Stefania Croci, Marina Morganti, Ilaria Menozzi, Erika Scaltriti, Stefano Pongolini
EPI_ISL_2191999	Lab voor klinische biologie	Lab voor klinische biologie	Marija Janevska, Hannelore Hamerlinck, Bruno Verhasselt
EPI_ISL_2196226, EPI_ISL_2196227	Laboratorio Central de Saude Publica do Estado de Sergipe (LACEN/SE)	Laboratory of Respiratory Viruses and Measles, Oswaldo Cruz Institute, FIOCRUZ	Paola Resende, Luciana Appolinario, Fernando Motta, Anna Carolina Paixao, Ana Carolina Mendonca, Alice Sampaio Rocha, Tainá Moreira Martins Venas, Elisa Cavalcante Pereira, Renata Serrano Lopes, Cliomar Alves dos Santos, Marilda Siqueira on behalf of the Fiocruz COVID-19 Genomic Surveillance Network
EPI_ISL_2196259	Lboratorio Central de Saude Publica do Estado do Parana (LACEN/PR)	Laboratory of Respiratory Viruses and Measles, Oswaldo Cruz Institute, FIOCRUZ	Paola Resende, Luciana Appolinario, Fernando Motta, Anna Carolina Paixao, Ana Carolina Mendonca, Alice Sampaio Rocha, Taina Venas, Elisa Cavalcante Pereira, Renata Serrano Lopes, Irina Riediger, Marilda Siqueira on behalf of the Fiocruz COVID-19 Genomic Surveillance Network
EPI_ISL_2196337	Labortorio Central de Saude Publica do Estado de Santa Catarina (LACEN/SC)	Laboratory of Respiratory Viruses and Measles, Oswaldo Cruz Institute, FIOCRUZ	Paola Resende, Luciana Appolinario, Fernando Motta, Anna Carolina Paixao, Ana Carolina Mendonca, Alice Sampaio Rocha, Taina Venas, Elisa Cavalcante Pereira, Renata Serrano Lopes, Darcita Buerger Rovaris, Sandra Bianchini Fernandes, Marilda Siqueira on behalf of the Fiocruz COVID-19 Genomic Surveillance Network
EPI_ISL_2202410, EPI_ISL_2204736	Aegis Sciences Corporation	Centers for Disease Control and Prevention Division of Viral Diseases, Pathogen Discovery	Dakota Howard, Dhwani Batra, Peter W. Cook, Kara Moser, Adrian Paskey, Jason Caravas, Benjamin Rambo-Martin, Shatavia Morrison, Christopher Gulvick, Scott Sammons, Yvette Unoarumhi, Darlene Wagner, Matthew Schmerer, Cyndi Clark, Patrick Campbell, Rob Case, Vikramsinha Ghorpade, Holly Houdeshell, Ola Kvalvaag, Dillon Nall, Ethan Sanders, Alec Vest, Shaun Westlund, Matthew Hardison, Clinton R. Paden, Duncan MacCannell
EPI_ISL_2209271	SECRETARIA MUNICIPAL DE SAUDE DE CORDEIROPOLIS	Instituto Butantan / FZEA-USP-Pirassununga	Dimas Tadeu Covas, Antonio Jorge Martins, Claudia Renata dos Santos Barros, David Schlesinger, Debora Botequio Moretti, Elaine Cristina Marqueze, Elaine Vieira Santos, Evandra Strazza Rodrigues, Heidge Fukumasu, Jayme Augusto de Souza-Neto, José Salvatore Leister Patané, Luiz Alcantara, Luiz Lehmann Coutinho, Maria Carolina Elias, Maurício Lacerda Nogueira, Rafael dos Santos Bezerra, Raul Machado Neto, Rejane Maria Tommasini Grotto, Ricardo Haddad, Sandra Coccuzzo Sampaio Vessoni, Simone Kashima, Svetoslav Nanev Slavov, Vincent Louis Viala
EPI_ISL_2210133	SANTA CASA DE MISERICORDIA DE UBATUBA	Instituto Butantan / Mendelics	Dimas Tadeu Covas, Antonio Jorge Martins, Claudia Renata dos Santos Barros, David Schlesinger, Debora Botequio Moretti, Elaine Cristina Marqueze, Elaine Vieira Santos, Evandra Strazza Rodrigues, Heidge Fukumasu, Jayme Augusto de Souza-Neto, José Salvatore Leister Patané, Luiz Alcantara, Luiz Lehmann Coutinho, Maria Carolina Elias, Maurício Lacerda Nogueira, Rafael dos Santos Bezerra, Raul Machado Neto, Rejane Maria Tommasini Grotto, Ricardo Haddad, Sandra Coccuzzo Sampaio Vessoni, Simone Kashima, Svetoslav Nanev Slavov, Vincent Louis Viala
EPI_ISL_2210295	VIGILANCIA SANITARIA E VIG EPIDEMIOLOGICA DE ITAPEVI	Instituto Butantan	Dimas Tadeu Covas, Antonio Jorge Martins, Claudia Renata dos Santos Barros, David Schlesinger, Debora Botequio Moretti, Elaine Cristina Marqueze, Elaine Vieira Santos, Evandra Strazza Rodrigues, Heidge Fukumasu, Jayme Augusto de Souza-Neto, José Salvatore Leister Patané, Luiz Alcantara, Luiz Lehmann Coutinho, Maria Carolina Elias, Maurício Lacerda Nogueira, Rafael dos Santos Bezerra, Raul Machado Neto, Rejane Maria Tommasini Grotto, Ricardo Haddad, Sandra Coccuzzo Sampaio Vessoni, Simone Kashima, Svetoslav Nanev Slavov, Vincent Louis Viala.
EPI_ISL_2222845	Houston Methodist Hospital	Houston Methodist Hospital	Randall J. Olsen, Paul A. Christensen, S. Wesley Long, Sishir Subedi, Robert Olson, Marcus Nguyen, James J. Davis, Matthew Ojeda Saavedra, Prasanti Yerramilli, Layne Pruitt, Kristina Reppond, Madison N. Shyer, Jessica Cambric, Ryan Gadd, Ilya J. Finkelstein, Jimmy Gollihar, and James M. Musser
EPI_ISL_2229749	Kaleida Health Laboratories	University at Buffalo Genomics and Bioinformatics Core	Jonathan Bard, Natalie Lamb, Alyssa Pohlman, Brandon Marzullo, Amanda Boccolucci, Norma Nowak, Donald Yergeau, Jennifer Surtees
EPI_ISL_2229992	Florida Bureau of Public Health Laboratories	Florida Bureau of Public Health Laboratories	Sarah Schmedes, Jason Blanton
EPI_ISL_2230735	Southern Nevada Public Health Laboratory	Southern Nevada Public Health Laboratory	Michael Picker
EPI_ISL_2230902	Salud Digna	Instituto Nacional de Medicina Genomica	Hidalgo-Miranda A, Cedro-Tanda A, Mendoza-Vargas A, Reyes-Grajeda JP, Abraham Campos-Romero, Moreno-Camacho José Luis, Rodriguez-Gallegos Jorge, Luna-Ruiz Marco, Gonzalez-Barrera D, Rangel-DeLeon D, Munguia-Garza P, Ramirez-Vega O, Escobar-Arrazola, M, Herrera-Montalvo LA.
EPI_ISL_2234875	IICS-UNA	IICS-UNA	Magaly Martinez, Adriana Valenzuela, Alejandra Rojas, Chyntia Diaz, Eva Nara, Fatima Cardozo, Florencia del Puerto, Joel Ortiz, Jonas Fernandez, Laura Franco, Laura Mendoza, Leticia Rojas, Maria Eugenia Galeano.
EPI_ISL_2241678, EPI_ISL_2241689, EPI_ISL_2242272	Aegis Sciences Corporation	Centers for Disease Control and Prevention Division of Viral Diseases, Pathogen Discovery	Dakota Howard, Dhwani Batra, Peter W. Cook, Kara Moser, Adrian Paskey, Jason Caravas, Benjamin Rambo-Martin, Shatavia Morrison, Christopher Gulvick, Scott Sammons, Yvette Unoarumhi, Darlene Wagner, Matthew Schmerer, Cyndi Clark, Patrick Campbell, Rob Case, Vikramsinha Ghorpade, Holly Houdeshell, Ola Kvalvaag, Dillon Nall, Ethan Sanders, Alec Vest, Shaun Westlund, Matthew Hardison, Clinton R. Paden, Duncan MacCannell
EPI_ISL_2245089	Laboratório Central de Saúde Pública do Pará	Coordenação Geral de Laboratórios de Saúde Pública (CGLAB/DAEVS/SVS/MS)	Vagner Fonseca, et al.
EPI_ISL_2245111	Laboratório Central de Saúde Pública de Roraima	Coordenação Geral de Laboratórios de Saúde Pública (CGLAB/DAEVS/SVS/MS)	Vagner Fonseca, et al.
EPI_ISL_2249394	Laboratório Central de Saúde Pública de Santa Catarina	Coordenação Geral de Laboratórios de Saúde Pública (CGLAB/DAEVS/SVS/MS)	Vagner Fonseca, et al.
EPI_ISL_2254438	Respiratory Viruses Branch, Centers for Disease Control and Prevention	Respiratory Viruses Branch, Centers for Disease Control and Prevention	Howard,D., Batra,D., Cook,P.W., Moser,K., Paskey,A., Caravas,J., Rambo-Martin,B., Morrison,S., Gulvick,C., Sammons,S., Unoarumhi,Y., Wagner,D., Schmerer,M., Clark,C., Campbell,P., Case,R., Ghorpade,V., Houdeshell,H., Kvalvaag,O., Nall,D., Sanders,E., Vest,A., Westlund,S., Hardison,M., Paden,C.R., MacCannell,D.
EPI_ISL_2260350	Bioscientia Labor Wermsdorf	Robert Koch Institute	unknown
EPI_ISL_2268148	Quest Diagnostics Incorporated	Centers for Disease Control and Prevention Division of Viral Diseases, Pathogen Discovery	Dakota Howard, Dhwani Batra, Peter W. Cook, Kara Moser, Adrian Paskey, Jason Caravas, Benjamin Rambo-Martin, Shatavia Morrison, Christopher Gulvick, Scott Sammons, Yvette Unoarumhi, Darlene Wagner, Matthew Schmerer, S. H. Rosenthal, A. Gerasimova, R. M. Kagan, B. Anderson, M. Hua, Y. Liu, L.E. Bernstein, K.E. Livingston, A. Perez, I. A. Shlyakhter, R. V. Rolando, R. Owen, P. Tanpaiboon, F. Lacbawan, Clinton R. Paden, Duncan MacCannell
EPI_ISL_2270111, EPI_ISL_2270260	Helix/Illumina	Centers for Disease Control and Prevention Division of Viral Diseases, Pathogen Discovery	Dakota Howard, Dhwani Batra, Peter W. Cook, Kara Moser, Adrian Paskey, Jason Caravas, Benjamin Rambo-Martin, Shatavia Morrison, Christopher Gulvick, Scott Sammons, Yvette Unoarumhi, Darlene Wagner, Matthew Schmerer, Eileen de Feo, Jan Antico, Christine Tran, Matthew Tolentino, Shannon

			Wickline, Kim Gietzen, Brad Sickler, Jingtao Liu, Eric Allen, Phil Febbo, Nicole L. Washington, Simon White, Geraint Levan, Kelly Schiabor Barrett, Elizabeth Cirulli, Alexandre Bolze, Ary Ascencio, Charlotte Rivera-Garcia, Ryan Cho, Jason Nguyen, Sherry Wang, Jimmy Ramirez, Tyler Cassens, Efren Sandoval, Magnus Isaksson, William Lee, David Becker, Marc Laurent, James Lu, Clinton R. Paden, Duncan MacCannell
EPI_ISL_2271687, EPI_ISL_2271688, EPI_ISL_2271689, EPI_ISL_2271690, EPI_ISL_2271694, EPI_ISL_2271697, EPI_ISL_2271699, EPI_ISL_2271704, EPI_ISL_2271705, EPI_ISL_2271706, EPI_ISL_2271708			
see above	Laboratorio Central, Ministerio de Salud Cordoba	Instituto de Patologia Vegetal (CIAP-INTA) on behalf of 'Proyecto Argentino Interinstitucional de genomica de SARS-CoV-2' (PAIS Consortium)	Fernandez, FD; Marquez, N.; Debat, HJ.; M; Re, V.; Pisano, M.B.; Castro, G.; Barbas, G.
EPI_ISL_2274087	Laboratorio Central de Saude Publica do Estado Maranhao (LACEN-MA)	Laboratory of Respiratory Viruses and Measles, Oswaldo Cruz Institute, FIOCRUZ	Paola Resende, Luciana Appolinario, Fernando Motta, Anna Carolina Paixao, Ana Carolina Mendonca, Alice Sampaio Rocha, Taina Venas, Elisa Cavalcante Pereira, Renata Serrano Lopes, Lidio Gonçalves Lima Neto, Marilda Siqueira on behalf of the Fiocruz COVID-19 Genomic Surveillance Network
EPI_ISL_2274118, EPI_ISL_2274121	Laboratorio Central de Saude Publica do Estado do Rio Grande do Sul (LACEN-RS)	Laboratory of Respiratory Viruses and Measles, Oswaldo Cruz Institute, FIOCRUZ	Paola Resende, Luciana Appolinario, Fernando Motta, Anna Carolina Paixao, Ana Carolina Mendonca, Alice Sampaio Rocha, Taina Venas, Elisa Cavalcante Pereira, Renata Serrano Lopes, Tatiana Schaffer Gregianini, Richard Salvato, Marilda Siqueira on behalf of the Fiocruz COVID-19 Genomic Surveillance Network
EPI_ISL_2277962	Florida Bureau of Public Health Laboratories	Florida Bureau of Public Health Laboratories	Sarah Schmedes, Jason Blanton
EPI_ISL_2279669	Labo analyses med	National Reference Center for Viruses of Respiratory Infections, Institut Pasteur, Paris	Marion Barbet, Sylvie Behillil, Méline Bizard, Angela Brisebarre, Camille Capel, Vincent Enouf, Louise Lefrançois, Frédéric Lemoine, Christophe Malabat, Corinne Maufrais, Victoire Baillet, Etienne Simon-Lorière, Maud Vanpeene, Sylvie Van der Werf ,OphéLie Said-Delattre
EPI_ISL_2280119, EPI_ISL_2280136	Quest Diagnostics Incorporated	Centers for Disease Control and Prevention Division of Viral Diseases, Pathogen Discovery	Dakota Howard, Dhwani Batra, Peter W. Cook, Kara Moser, Adrian Paskey, Jason Caravas, Benjamin Rambo-Martin, Shatavia Morrison, Christopher Gulvick, Scott Sammons, Yvette Unoarumhi, Darlene Wagner, Matthew Schmerer, S. H. Rosenthal, A. Gerasimova, R. M. Kagan, B. Anderson, M. Hua, Y. Liu, L.E. Bernstein, K.E. Livingston, A. Perez, I. A. Shlyakhter, R. V. Rolando, R. Owen, P. Tanpaiboon, F. Lacbawan, Clinton R. Paden, Duncan MacCannell
EPI_ISL_2280917	Aegis Sciences Corporation	Centers for Disease Control and Prevention Division of Viral Diseases, Pathogen Discovery	Dakota Howard, Dhwani Batra, Peter W. Cook, Kara Moser, Adrian Paskey, Jason Caravas, Benjamin Rambo-Martin, Shatavia Morrison, Christopher Gulvick, Scott Sammons, Yvette Unoarumhi, Darlene Wagner, Matthew Schmerer, Cyndi Clark, Patrick Campbell, Rob Case, Vikramsinha Ghorpade, Holly Houdeshell, Ola Kvalvaag, Dillon Nall, Ethan Sanders, Alec Vest, Shaun Westlund, Matthew Hardison, Clinton R. Paden, Duncan MacCannell
EPI_ISL_2283321	Infinity Biologix	Centers for Disease Control and Prevention Division of Viral Diseases, Pathogen Discovery	Dakota Howard, Dhwani Batra, Peter W. Cook, Kara Moser, Adrian Paskey, Jason Caravas, Benjamin Rambo-Martin, Shatavia Morrison, Christopher Gulvick, Scott Sammons, Yvette Unoarumhi, Darlene Wagner, Matthew Schmerer, Christian Bixby, Yihe Wang, Jonathan Schultz, Chirayu Goswami, Russ Hager, Robin Grimwood, Clinton R. Paden, Duncan MacCannell
EPI_ISL_2284932	Hospital Universitari Vall d'Hebron - Vall d'Hebron Institut de Recerca	Hospital Universitari Vall d'Hebron - Vall d'Hebron Institut de Recerca	Cristina Andrés, Maria Piñana, Alejandra González-Sánchez, Damir Garcia-Cehic, Ariadna Rando, Juliana Esperalba, Maria Gema Codina, Carla Castillo, Maria Carmen Martin, Tomàs Pumarola, Josep Quer, Andrés Antón
EPI_ISL_2293000, EPI_ISL_2293003, EPI_ISL_2293004	Laboratório Central de Saúde Pública de Santa Catarina	Coordenação Geral de Laboratórios de Saúde Pública (CGLAB/DAEVS/SVS/MS)	Vagner Fonseca, et al.
EPI_ISL_2296061	LESP Quintana Roo	Instituto de Diagnostico y Referencia Epidemiologicos (INDRE)	Claudia Wong-Arambula, Abril Rodriguez-Maldonado, Vanessa Rivero-Arredondo, Ariadna Medina-Benitez, Joaquin Quiroz-Mercado, Sergio Rangel-Guerrero, Natividad Cruz-Ortiz, Tatiana Nunez-Garcia, Gisela Barrera-Badillo, Lucia Hernandez-Rivas, Irma Lopez-Martinez, Ernesto Ramirez-Gonzalez.
EPI_ISL_2296083	LESP Ciudad de Mexico	Instituto de Diagnostico y Referencia Epidemiologicos (INDRE)	Claudia Wong-Arambula, Abril Rodriguez-Maldonado, Vanessa Rivero-Arredondo, Ariadna Medina-Benitez, Joaquin Quiroz-Mercado, Sergio Rangel-Guerrero, Natividad Cruz-Ortiz, Tatiana Nunez-Garcia, Gisela Barrera-Badillo, Lucia Hernandez-Rivas, Irma Lopez-Martinez, Ernesto Ramirez-Gonzalez.
EPI_ISL_2298755	Laboratório Central de Saúde Pública do Maranhão	Coordenação Geral de Laboratórios de Saúde Pública (CGLAB/DAEVS/SVS/MS)	Vagner Fonseca, et al.
EPI_ISL_2298769	Laboratório Central de Saúde Pública de Roraima	Coordenação Geral de Laboratórios de Saúde Pública (CGLAB/DAEVS/SVS/MS)	Vagner Fonseca, et al.
EPI_ISL_2298812	Laboratório Central de Saúde Pública do Pará	Coordenação Geral de Laboratórios de Saúde Pública (CGLAB/DAEVS/SVS/MS)	Vagner Fonseca, et al.
EPI_ISL_2298856, EPI_ISL_2298867	Laboratório Central de Saúde Pública do Amazonas	Coordenação Geral de Laboratórios de Saúde Pública (CGLAB/DAEVS/SVS/MS)	Vagner Fonseca, et al.
EPI_ISL_2300486	Illinois Department of Public Health	Illinois Department of Public Health - Chicago Lab	Vineet K. Dhiman, Ira Heimler, Joel Price
EPI_ISL_2304185	Hospital General Universitario Gregorio Marañón	Hospital General Universitario Gregorio Marañón	Sergio Buenestado Serrano, Pedro Sola Campoy, Laura Pérez-Lago, Cristina Rodriguez-Grande, Marta Herranz Martin, Victor Manuel de la Cueva, Julia Suárez, Pilar Catalán, Patricia Muñoz, Darío García de Viedma
EPI_ISL_2306038	Laboratory Corporation of America	Centers for Disease Control and Prevention Division of Viral Diseases, Pathogen Discovery	Dakota Howard, Dhwani Batra, Peter W. Cook, Kara Moser, Adrian Paskey, Jason Caravas, Benjamin Rambo-Martin, Shatavia Morrison, Christopher Gulvick, Scott Sammons, Yvette Unoarumhi, Darlene Wagner, Matthew Schmerer, Minoo Agarwal, Eyad Almasri, Debbie Boles, Ayla Burns, Nuthawin Charoensri, Oren Cohen, Susan Countryman, Mary Ann Cristobal, Bobbi Croy, Suzanne Dale, Hrushikesh Deshmukh, Amanda Douglas, Vincent Drouillon, Marcia Eisenberg, Howard Engler, Rama Ghatti, Prashant Gupta, Susan Hicks, Jake Humphrey, Lax Iyer, Manoj Jain, Mohan Kolli, Brian Krueger, Tim Kuphal, Stanley Letovsky, Michael Levandoski, Craig Lukasik, Jonathan Meltzer, Brian Norvell, Mindy Nye, Scott Parker, Christos Petropoulos, John Pruitt, Steven Ragan, Scott Ryan, Mike Sapeta, Jana Schroth, Suresh Babu Selvaraju, Goran Stevovic, Amanda Suchanek, Andrea Throop, Lyndon Tilson, Thomas Urban, Joe Voshell, Kimberly Wagner, Jonathan Williams, Mary Williamson, Qian Zeng, Tricia Zwiefelhofer, Clinton R. Paden, Duncan MacCannell
EPI_ISL_2308476	Laboratório Central de Saúde Pública de Sergipe	Coordenação Geral de Laboratórios de Saúde Pública (CGLAB/DAEVS/SVS/MS)	Vagner Fonseca, et al.
EPI_ISL_2323476	Infinity Biologix	Centers for Disease Control and Prevention Division of Viral Diseases, Pathogen Discovery	Dakota Howard, Dhwani Batra, Peter W. Cook, Kara Moser, Adrian Paskey, Jason Caravas, Benjamin Rambo-Martin, Shatavia Morrison, Christopher Gulvick, Scott Sammons, Yvette Unoarumhi, Darlene Wagner, Matthew Schmerer, Christian Bixby, Yihe Wang, Jonathan Schultz, Chirayu Goswami, Russ Hager, Robin Grimwood, Clinton R. Paden, Duncan MacCannell
EPI_ISL_2325066, EPI_ISL_2325068, EPI_ISL_2325069	Minnesota Department of Health, Public Health Laboratory	Minnesota Department of Health, Public Health Laboratory	Alexandra Lorentz, Jacob Garfin, Matt Plumb, and Xiong Wang
EPI_ISL_2331392, EPI_ISL_2331444	Azienda Ospedaliero - Universitaria di Modena Policlinico - Virologia e Microbiologia Molecolare	Istituto Zooprofilattico Sperimentale della Lombardia e dell'Emilia Romagna (IZSLER), Risk Analysis and Genomic Epidemiology Unit	Monica Pecorari, William Gennari, Giulia Fregni Serpini, Marina Morganti, Ilaria Menozzi, Erika Scaltriti, Stefano Pongolini
EPI_ISL_2341385	The Ohio State University Applied Microbiology Services Laboratory	The Ohio State University Applied Microbiology Services Laboratory	Seth A. Faith PhD
EPI_ISL_2344602	Prefeirtura de SP	Instituto Butantan	Dimas Tadeu Covas, Antonio Jorge Martins, Claudia Renata dos Santos Barros, David Schlesinger, Debora Botequio Moretti, Elaine Cristina Marqueze, Elaine Vieira Santos, Evandra Strazza Rodrigues, Heidge Fukumasu, Jayme Augusto de Souza-Neto, José Salvatore Leister Patané, Luiz Alcantara, Luiz Lehmann Coutinho, Maria Carolina Elias, Maurício Lacerda Nogueira, Rafael dos Santos Bezerra, Raul Machado Neto, Rejane Maria Tommasini Grotto, Ricardo Haddad, Sandra Coccuzzo Sampaio Vessoni, Simone Kashima, Svetoslav Nanev Slavov, Vincent Louis Viala
EPI_ISL_2344769	CS DE RINOPOLIS	Instituto Butantan / ESALQ-Piracicaba	Dimas Tadeu Covas, Antonio Jorge Martins, Claudia Renata dos Santos Barros, David Schlesinger, Debora Botequio Moretti, Elaine Cristina Marqueze, Elaine Vieira Santos, Evandra Strazza Rodrigues, Heidge Fukumasu, Jayme Augusto de Souza-Neto, José Salvatore Leister Patané, Luiz Alcantara, Luiz Lehmann Coutinho, Maria Carolina Elias, Maurício Lacerda Nogueira, Rafael dos Santos Bezerra, Raul Machado Neto, Rejane Maria Tommasini Grotto,

			Ricardo Haddad, Sandra Coccuzzo Sampaio Vessoni, Simone Kashima, Svetoslav Nanev Slavov, Vincent Louis Viala
EPI_ISL_2344815, EPI_ISL_2344817	SAE CTA SERV ATEND ESPEC TESTAGEM E ACONSELHAMENTO	Instituto Butantan / ESALQ-Piracicaba	Dimas Tadeu Covas, Antonio Jorge Martins, Claudia Renata dos Santos Barros, David Schlesinger, Debora Botequio Moretti, Elaine Cristina Marqueze, Elaine Vieira Santos, Evandra Strazza Rodrigues, Heidge Fukumasu, Jayme Augusto de Souza-Neto, José Salvatore Leister Patané, Luiz Alcantara, Luiz Lehmann Coutinho, Maria Carolina Elias, Maurício Lacerda Nogueira, Rafael dos Santos Bezerra, Raul Machado Neto, Rejane Maria Tommasini Grotto, Ricardo Haddad, Sandra Coccuzzo Sampaio Vessoni, Simone Kashima, Svetoslav Nanev Slavov, Vincent Louis Viala
EPI_ISL_2344849	HOSPITAL DE MISERICORDIA ALTINOPOLIS	Instituto Butantan / ESALQ-Piracicaba	Dimas Tadeu Covas, Antonio Jorge Martins, Claudia Renata dos Santos Barros, David Schlesinger, Debora Botequio Moretti, Elaine Cristina Marqueze, Elaine Vieira Santos, Evandra Strazza Rodrigues, Heidge Fukumasu, Jayme Augusto de Souza-Neto, José Salvatore Leister Patané, Luiz Alcantara, Luiz Lehmann Coutinho, Maria Carolina Elias, Maurício Lacerda Nogueira, Rafael dos Santos Bezerra, Raul Machado Neto, Rejane Maria Tommasini Grotto, Ricardo Haddad, Sandra Coccuzzo Sampaio Vessoni, Simone Kashima, Svetoslav Nanev Slavov, Vincent Louis Viala
EPI_ISL_2344860	CS DE CATIGUA	Instituto Butantan / ESALQ-Piracicaba	Dimas Tadeu Covas, Antonio Jorge Martins, Claudia Renata dos Santos Barros, David Schlesinger, Debora Botequio Moretti, Elaine Cristina Marqueze, Elaine Vieira Santos, Evandra Strazza Rodrigues, Heidge Fukumasu, Jayme Augusto de Souza-Neto, José Salvatore Leister Patané, Luiz Alcantara, Luiz Lehmann Coutinho, Maria Carolina Elias, Maurício Lacerda Nogueira, Rafael dos Santos Bezerra, Raul Machado Neto, Rejane Maria Tommasini Grotto, Ricardo Haddad, Sandra Coccuzzo Sampaio Vessoni, Simone Kashima, Svetoslav Nanev Slavov, Vincent Louis Viala
EPI_ISL_2344900	HOSPITAL E PRONTO SOCORRO PORTINARI	Instituto Butantan	Dimas Tadeu Covas, Antonio Jorge Martins, Claudia Renata dos Santos Barros, David Schlesinger, Debora Botequio Moretti, Elaine Cristina Marqueze, Elaine Vieira Santos, Evandra Strazza Rodrigues, Heidge Fukumasu, Jayme Augusto de Souza-Neto, José Salvatore Leister Patané, Luiz Alcantara, Luiz Lehmann Coutinho, Maria Carolina Elias, Maurício Lacerda Nogueira, Rafael dos Santos Bezerra, Raul Machado Neto, Rejane Maria Tommasini Grotto, Ricardo Haddad, Sandra Coccuzzo Sampaio Vessoni, Simone Kashima, Svetoslav Nanev Slavov, Vincent Louis Viala
EPI_ISL_2344955	HOSPITAL DE CAMPANHA COVID 19 MUNICIPIO DE TAUBATE	Instituto Butantan / ESALQ-Piracicaba	Dimas Tadeu Covas, Antonio Jorge Martins, Claudia Renata dos Santos Barros, David Schlesinger, Debora Botequio Moretti, Elaine Cristina Marqueze, Elaine Vieira Santos, Evandra Strazza Rodrigues, Heidge Fukumasu, Jayme Augusto de Souza-Neto, José Salvatore Leister Patané, Luiz Alcantara, Luiz Lehmann Coutinho, Maria Carolina Elias, Maurício Lacerda Nogueira, Rafael dos Santos Bezerra, Raul Machado Neto, Rejane Maria Tommasini Grotto, Ricardo Haddad, Sandra Coccuzzo Sampaio Vessoni, Simone Kashima, Svetoslav Nanev Slavov, Vincent Louis Viala
EPI_ISL_2344981, EPI_ISL_2344994	CENTRO DE APOIO EPIDEMIOLOGICO	Instituto Butantan / ESALQ-Piracicaba	Dimas Tadeu Covas, Antonio Jorge Martins, Claudia Renata dos Santos Barros, David Schlesinger, Debora Botequio Moretti, Elaine Cristina Marqueze, Elaine Vieira Santos, Evandra Strazza Rodrigues, Heidge Fukumasu, Jayme Augusto de Souza-Neto, José Salvatore Leister Patané, Luiz Alcantara, Luiz Lehmann Coutinho, Maria Carolina Elias, Maurício Lacerda Nogueira, Rafael dos Santos Bezerra, Raul Machado Neto, Rejane Maria Tommasini Grotto, Ricardo Haddad, Sandra Coccuzzo Sampaio Vessoni, Simone Kashima, Svetoslav Nanev Slavov, Vincent Louis Viala
EPI_ISL_2345007	SANTA CASA DE MISERICORDIA SAO JOSE	Instituto Butantan / ESALQ-Piracicaba	Dimas Tadeu Covas, Antonio Jorge Martins, Claudia Renata dos Santos Barros, David Schlesinger, Debora Botequio Moretti, Elaine Cristina Marqueze, Elaine Vieira Santos, Evandra Strazza Rodrigues, Heidge Fukumasu, Jayme Augusto de Souza-Neto, José Salvatore Leister Patané, Luiz Alcantara, Luiz Lehmann Coutinho, Maria Carolina Elias, Maurício Lacerda Nogueira, Rafael dos Santos Bezerra, Raul Machado Neto, Rejane Maria Tommasini Grotto, Ricardo Haddad, Sandra Coccuzzo Sampaio Vessoni, Simone Kashima, Svetoslav Nanev Slavov, Vincent Louis Viala
EPI_ISL_2345053	UNIDADE RESPIRATORIA NOVA HORTOLANDIA	Instituto Butantan / ESALQ-Piracicaba	Dimas Tadeu Covas, Antonio Jorge Martins, Claudia Renata dos Santos Barros, David Schlesinger, Debora Botequio Moretti, Elaine Cristina Marqueze, Elaine Vieira Santos, Evandra Strazza Rodrigues, Heidge Fukumasu, Jayme Augusto de Souza-Neto, José Salvatore Leister Patané, Luiz Alcantara, Luiz Lehmann Coutinho, Maria Carolina Elias, Maurício Lacerda Nogueira, Rafael dos Santos Bezerra, Raul Machado Neto, Rejane Maria Tommasini Grotto, Ricardo Haddad, Sandra Coccuzzo Sampaio Vessoni, Simone Kashima, Svetoslav Nanev Slavov, Vincent Louis Viala
EPI_ISL_2345130	HOSPITAL NOSSA SENHORA APARECIDA	Instituto Butantan / ESALQ-Piracicaba	Dimas Tadeu Covas, Antonio Jorge Martins, Claudia Renata dos Santos Barros, David Schlesinger, Debora Botequio Moretti, Elaine Cristina Marqueze, Elaine Vieira Santos, Evandra Strazza Rodrigues, Heidge Fukumasu, Jayme Augusto de Souza-Neto, José Salvatore Leister Patané, Luiz Alcantara, Luiz Lehmann Coutinho, Maria Carolina Elias, Maurício Lacerda Nogueira, Rafael dos Santos Bezerra, Raul Machado Neto, Rejane Maria Tommasini Grotto, Ricardo Haddad, Sandra Coccuzzo Sampaio Vessoni, Simone Kashima, Svetoslav Nanev Slavov, Vincent Louis Viala
EPI_ISL_2345220	CENTRO DE SAUDE DE AVAI	Instituto Butantan / ESALQ-Piracicaba	Dimas Tadeu Covas, Antonio Jorge Martins, Claudia Renata dos Santos Barros, David Schlesinger, Debora Botequio Moretti, Elaine Cristina Marqueze, Elaine Vieira Santos, Evandra Strazza Rodrigues, Heidge Fukumasu, Jayme Augusto de Souza-Neto, José Salvatore Leister Patané, Luiz Alcantara, Luiz Lehmann Coutinho, Maria Carolina Elias, Maurício Lacerda Nogueira, Rafael dos Santos Bezerra, Raul Machado Neto, Rejane Maria Tommasini Grotto, Ricardo Haddad, Sandra Coccuzzo Sampaio Vessoni, Simone Kashima, Svetoslav Nanev Slavov, Vincent Louis Viala
EPI_ISL_2345223, EPI_ISL_2345225, EPI_ISL_2345228	UBS IV GUARARAPES	Instituto Butantan / ESALQ-Piracicaba	Dimas Tadeu Covas, Antonio Jorge Martins, Claudia Renata dos Santos Barros, David Schlesinger, Debora Botequio Moretti, Elaine Cristina Marqueze, Elaine Vieira Santos, Evandra Strazza Rodrigues, Heidge Fukumasu, Jayme Augusto de Souza-Neto, José Salvatore Leister Patané, Luiz Alcantara, Luiz Lehmann Coutinho, Maria Carolina Elias, Maurício Lacerda Nogueira, Rafael dos Santos Bezerra, Raul Machado Neto, Rejane Maria Tommasini Grotto, Ricardo Haddad, Sandra Coccuzzo Sampaio Vessoni, Simone Kashima, Svetoslav Nanev Slavov, Vincent Louis Viala
EPI_ISL_2345267	LABORATORIO MUNICIPAL DE PIRACICABA	Instituto Butantan / ESALQ-Piracicaba	Dimas Tadeu Covas, Antonio Jorge Martins, Claudia Renata dos Santos Barros, David Schlesinger, Debora Botequio Moretti, Elaine Cristina Marqueze, Elaine Vieira Santos, Evandra Strazza Rodrigues, Heidge Fukumasu, Jayme Augusto de Souza-Neto, José Salvatore Leister Patané, Luiz Alcantara, Luiz Lehmann Coutinho, Maria Carolina Elias, Maurício Lacerda Nogueira, Rafael dos Santos Bezerra, Raul Machado Neto, Rejane Maria Tommasini Grotto, Ricardo Haddad, Sandra Coccuzzo Sampaio Vessoni, Simone Kashima, Svetoslav Nanev Slavov, Vincent Louis Viala
EPI_ISL_2345332, EPI_ISL_2345335, EPI_ISL_2345336	UNIDADE DE VIGILANCIA EM SAUDE	Instituto Butantan / ESALQ-Piracicaba	Dimas Tadeu Covas, Antonio Jorge Martins, Claudia Renata dos Santos Barros, David Schlesinger, Debora Botequio Moretti, Elaine Cristina Marqueze, Elaine Vieira Santos, Evandra Strazza Rodrigues, Heidge Fukumasu, Jayme Augusto de Souza-Neto, José Salvatore Leister Patané, Luiz Alcantara, Luiz Lehmann Coutinho, Maria Carolina Elias, Maurício Lacerda Nogueira, Rafael dos Santos Bezerra, Raul Machado Neto, Rejane Maria Tommasini Grotto, Ricardo Haddad, Sandra Coccuzzo Sampaio Vessoni, Simone Kashima, Svetoslav Nanev Slavov, Vincent Louis Viala
EPI_ISL_2345372	CS III VILA ODILON	Instituto Butantan / ESALQ-Piracicaba	Dimas Tadeu Covas, Antonio Jorge Martins, Claudia Renata dos Santos Barros, David Schlesinger, Debora Botequio Moretti, Elaine Cristina Marqueze, Elaine Vieira Santos, Evandra Strazza Rodrigues, Heidge Fukumasu, Jayme Augusto de Souza-Neto, José Salvatore Leister Patané, Luiz Alcantara, Luiz Lehmann Coutinho, Maria Carolina Elias, Maurício Lacerda Nogueira, Rafael dos Santos Bezerra, Raul Machado Neto, Rejane Maria Tommasini Grotto, Ricardo Haddad, Sandra Coccuzzo Sampaio Vessoni, Simone Kashima, Svetoslav Nanev Slavov, Vincent Louis Viala
EPI_ISL_2345402	POLICLINICA HORTOLANDIA	Instituto Butantan / ESALQ-Piracicaba	Dimas Tadeu Covas, Antonio Jorge Martins, Claudia Renata dos Santos Barros, David Schlesinger, Debora Botequio Moretti, Elaine Cristina Marqueze, Elaine Vieira Santos, Evandra Strazza Rodrigues, Heidge Fukumasu, Jayme Augusto de Souza-Neto, José Salvatore Leister Patané, Luiz Alcantara, Luiz Lehmann Coutinho, Maria Carolina Elias, Maurício Lacerda Nogueira, Rafael dos Santos Bezerra, Raul Machado Neto, Rejane Maria Tommasini Grotto, Ricardo Haddad, Sandra Coccuzzo Sampaio Vessoni, Simone Kashima, Svetoslav Nanev Slavov, Vincent Louis Viala
EPI_ISL_2345406	USF SALERNO	Instituto Butantan / ESALQ-Piracicaba	Dimas Tadeu Covas, Antonio Jorge Martins, Claudia Renata dos Santos Barros, David Schlesinger, Debora Botequio Moretti, Elaine Cristina Marqueze, Elaine Vieira Santos, Evandra Strazza Rodrigues, Heidge Fukumasu, Jayme Augusto de Souza-Neto, José Salvatore Leister Patané, Luiz Alcantara, Luiz Lehmann Coutinho, Maria Carolina Elias, Maurício Lacerda Nogueira, Rafael dos Santos Bezerra, Raul Machado Neto, Rejane Maria Tommasini Grotto, Ricardo Haddad, Sandra Coccuzzo Sampaio Vessoni, Simone Kashima, Svetoslav Nanev Slavov, Vincent Louis Viala
EPI_ISL_2345423	AMBULATORIO DE ESPECIALIDADE V E MOGI MIRIM	Instituto Butantan / ESALQ-Piracicaba	Dimas Tadeu Covas, Antonio Jorge Martins, Claudia Renata dos Santos Barros, David Schlesinger, Debora Botequio Moretti, Elaine Cristina Marqueze, Elaine Vieira Santos, Evandra Strazza Rodrigues, Heidge Fukumasu, Jayme Augusto de Souza-Neto, José Salvatore Leister Patané, Luiz Alcantara, Luiz Lehmann Coutinho, Maria Carolina Elias, Maurício Lacerda Nogueira, Rafael dos Santos Bezerra, Raul Machado Neto, Rejane Maria Tommasini Grotto, Ricardo Haddad, Sandra Coccuzzo Sampaio Vessoni, Simone Kashima, Svetoslav Nanev Slavov, Vincent Louis Viala
EPI_ISL_2345440	CENTRO DE SAUDE II DR ALCIDES FACUNDO ARROYO	Instituto Butantan / ESALQ-Piracicaba	Dimas Tadeu Covas, Antonio Jorge Martins, Claudia Renata dos Santos Barros, David Schlesinger, Debora Botequio Moretti, Elaine Cristina Marqueze, Elaine Vieira Santos, Evandra Strazza Rodrigues, Heidge Fukumasu, Jayme Augusto de Souza-Neto, José Salvatore Leister Patané, Luiz Alcantara, Luiz Lehmann Coutinho, Maria Carolina Elias, Maurício Lacerda Nogueira, Rafael dos Santos Bezerra, Raul Machado Neto, Rejane Maria Tommasini Grotto, Ricardo Haddad, Sandra Coccuzzo Sampaio Vessoni, Simone Kashima, Svetoslav Nanev Slavov, Vincent Louis Viala
EPI_ISL_2345456	UNIDADE BASICA DE SAUDE DR WILSON HAYEK SAIHG	Instituto Butantan / ESALQ-Piracicaba	Dimas Tadeu Covas, Antonio Jorge Martins, Claudia Renata dos Santos Barros, David Schlesinger, Debora Botequio Moretti, Elaine Cristina Marqueze, Elaine Vieira Santos, Evandra Strazza Rodrigues, Heidge Fukumasu, Jayme Augusto de Souza-Neto, José Salvatore Leister Patané, Luiz Alcantara, Luiz Lehmann Coutinho, Maria Carolina Elias, Maurício Lacerda Nogueira, Rafael dos Santos Bezerra, Raul Machado Neto, Rejane Maria Tommasini Grotto, Ricardo Haddad, Sandra Coccuzzo Sampaio Vessoni, Simone Kashima, Svetoslav Nanev Slavov, Vincent Louis Viala

EPI_ISL_2345458	UNIDADE BASICA DE SAUDE DR LOTFALLAH MIZIARA	Instituto Butantan / ESALQ-Piracicaba	Dimas Tadeu Covas, Antonio Jorge Martins, Claudia Renata dos Santos Barros, David Schlesinger, Debora Botequio Moretti, Elaine Cristina Marqueze, Elaine Vieira Santos, Evandra Strazza Rodrigues, Heidge Fukumasu, Jayme Augusto de Souza-Neto, José Salvatore Leister Patané, Luiz Alcantara, Luiz Lehmann Coutinho, Maria Carolina Elias, Maurício Lacerda Nogueira, Rafael dos Santos Bezerra, Raul Machado Neto, Rejane Maria Tommasini Grotto, Ricardo Haddad, Sandra Coccuzzo Sampaio Vessoni, Simone Kashima, Svetoslav Nanev Slavov, Vincent Louis Viala
EPI_ISL_2345461	UNIDADE DE SAUDE DA FAMILIA JOSE ADALBERTO LELLIS GARCIA	Instituto Butantan / ESALQ-Piracicaba	Dimas Tadeu Covas, Antonio Jorge Martins, Claudia Renata dos Santos Barros, David Schlesinger, Debora Botequio Moretti, Elaine Cristina Marqueze, Elaine Vieira Santos, Evandra Strazza Rodrigues, Heidge Fukumasu, Jayme Augusto de Souza-Neto, José Salvatore Leister Patané, Luiz Alcantara, Luiz Lehmann Coutinho, Maria Carolina Elias, Maurício Lacerda Nogueira, Rafael dos Santos Bezerra, Raul Machado Neto, Rejane Maria Tommasini Grotto, Ricardo Haddad, Sandra Coccuzzo Sampaio Vessoni, Simone Kashima, Svetoslav Nanev Slavov, Vincent Louis Viala
EPI_ISL_2345558, EPI_ISL_2345584	LABORATORIO DE FRANCA	Instituto Butantan / ESALQ-Piracicaba	Dimas Tadeu Covas, Antonio Jorge Martins, Claudia Renata dos Santos Barros, David Schlesinger, Debora Botequio Moretti, Elaine Cristina Marqueze, Elaine Vieira Santos, Evandra Strazza Rodrigues, Heidge Fukumasu, Jayme Augusto de Souza-Neto, José Salvatore Leister Patané, Luiz Alcantara, Luiz Lehmann Coutinho, Maria Carolina Elias, Maurício Lacerda Nogueira, Rafael dos Santos Bezerra, Raul Machado Neto, Rejane Maria Tommasini Grotto, Ricardo Haddad, Sandra Coccuzzo Sampaio Vessoni, Simone Kashima, Svetoslav Nanev Slavov, Vincent Louis Viala
EPI_ISL_2345588	CENTRO DE SAUDE II JUNQUEIROPOLIS	Instituto Butantan / ESALQ-Piracicaba	Dimas Tadeu Covas, Antonio Jorge Martins, Claudia Renata dos Santos Barros, David Schlesinger, Debora Botequio Moretti, Elaine Cristina Marqueze, Elaine Vieira Santos, Evandra Strazza Rodrigues, Heidge Fukumasu, Jayme Augusto de Souza-Neto, José Salvatore Leister Patané, Luiz Alcantara, Luiz Lehmann Coutinho, Maria Carolina Elias, Maurício Lacerda Nogueira, Rafael dos Santos Bezerra, Raul Machado Neto, Rejane Maria Tommasini Grotto, Ricardo Haddad, Sandra Coccuzzo Sampaio Vessoni, Simone Kashima, Svetoslav Nanev Slavov, Vincent Louis Viala
EPI_ISL_2345602	PAS JOAO ANTONIO DO NASCIMENTO	Instituto Butantan / ESALQ-Piracicaba	Dimas Tadeu Covas, Antonio Jorge Martins, Claudia Renata dos Santos Barros, David Schlesinger, Debora Botequio Moretti, Elaine Cristina Marqueze, Elaine Vieira Santos, Evandra Strazza Rodrigues, Heidge Fukumasu, Jayme Augusto de Souza-Neto, José Salvatore Leister Patané, Luiz Alcantara, Luiz Lehmann Coutinho, Maria Carolina Elias, Maurício Lacerda Nogueira, Rafael dos Santos Bezerra, Raul Machado Neto, Rejane Maria Tommasini Grotto, Ricardo Haddad, Sandra Coccuzzo Sampaio Vessoni, Simone Kashima, Svetoslav Nanev Slavov, Vincent Louis Viala
EPI_ISL_2345606	VIGILANCIA EM SAUDE	Instituto Butantan / ESALQ-Piracicaba	Dimas Tadeu Covas, Antonio Jorge Martins, Claudia Renata dos Santos Barros, David Schlesinger, Debora Botequio Moretti, Elaine Cristina Marqueze, Elaine Vieira Santos, Evandra Strazza Rodrigues, Heidge Fukumasu, Jayme Augusto de Souza-Neto, José Salvatore Leister Patané, Luiz Alcantara, Luiz Lehmann Coutinho, Maria Carolina Elias, Maurício Lacerda Nogueira, Rafael dos Santos Bezerra, Raul Machado Neto, Rejane Maria Tommasini Grotto, Ricardo Haddad, Sandra Coccuzzo Sampaio Vessoni, Simone Kashima, Svetoslav Nanev Slavov, Vincent Louis Viala
EPI_ISL_2345613	LABORATORIO DR PAULO EMILIO DALESSANDRO PINDAMONHANGABA	Instituto Butantan / ESALQ-Piracicaba	Dimas Tadeu Covas, Antonio Jorge Martins, Claudia Renata dos Santos Barros, David Schlesinger, Debora Botequio Moretti, Elaine Cristina Marqueze, Elaine Vieira Santos, Evandra Strazza Rodrigues, Heidge Fukumasu, Jayme Augusto de Souza-Neto, José Salvatore Leister Patané, Luiz Alcantara, Luiz Lehmann Coutinho, Maria Carolina Elias, Maurício Lacerda Nogueira, Rafael dos Santos Bezerra, Raul Machado Neto, Rejane Maria Tommasini Grotto, Ricardo Haddad, Sandra Coccuzzo Sampaio Vessoni, Simone Kashima, Svetoslav Nanev Slavov, Vincent Louis Viala
EPI_ISL_2345637, EPI_ISL_2345638	UNIDADE BASICA DE SAUDE DE PIQUETE	Instituto Butantan / ESALQ-Piracicaba	Dimas Tadeu Covas, Antonio Jorge Martins, Claudia Renata dos Santos Barros, David Schlesinger, Debora Botequio Moretti, Elaine Cristina Marqueze, Elaine Vieira Santos, Evandra Strazza Rodrigues, Heidge Fukumasu, Jayme Augusto de Souza-Neto, José Salvatore Leister Patané, Luiz Alcantara, Luiz Lehmann Coutinho, Maria Carolina Elias, Maurício Lacerda Nogueira, Rafael dos Santos Bezerra, Raul Machado Neto, Rejane Maria Tommasini Grotto, Ricardo Haddad, Sandra Coccuzzo Sampaio Vessoni, Simone Kashima, Svetoslav Nanev Slavov, Vincent Louis Viala
EPI_ISL_2345647	HOSPITAL MUNICIPAL DR WALDEMAR TEBALDI	Instituto Butantan / Mendelics	Dimas Tadeu Covas, Antonio Jorge Martins, Claudia Renata dos Santos Barros, David Schlesinger, Debora Botequio Moretti, Elaine Cristina Marqueze, Elaine Vieira Santos, Evandra Strazza Rodrigues, Heidge Fukumasu, Jayme Augusto de Souza-Neto, José Salvatore Leister Patané, Luiz Alcantara, Luiz Lehmann Coutinho, Maria Carolina Elias, Maurício Lacerda Nogueira, Rafael dos Santos Bezerra, Raul Machado Neto, Rejane Maria Tommasini Grotto, Ricardo Haddad, Sandra Coccuzzo Sampaio Vessoni, Simone Kashima, Svetoslav Nanev Slavov, Vincent Louis Viala
EPI_ISL_2345690	HOSPITAL MUNICIPAL DE ITAPIRA	Instituto Butantan / Mendelics	Dimas Tadeu Covas, Antonio Jorge Martins, Claudia Renata dos Santos Barros, David Schlesinger, Debora Botequio Moretti, Elaine Cristina Marqueze, Elaine Vieira Santos, Evandra Strazza Rodrigues, Heidge Fukumasu, Jayme Augusto de Souza-Neto, José Salvatore Leister Patané, Luiz Alcantara, Luiz Lehmann Coutinho, Maria Carolina Elias, Maurício Lacerda Nogueira, Rafael dos Santos Bezerra, Raul Machado Neto, Rejane Maria Tommasini Grotto, Ricardo Haddad, Sandra Coccuzzo Sampaio Vessoni, Simone Kashima, Svetoslav Nanev Slavov, Vincent Louis Viala
EPI_ISL_2345693	HOSPITAL MUNICIPAL DR WALDEMAR TEBALDI	Instituto Butantan / Mendelics	Dimas Tadeu Covas, Antonio Jorge Martins, Claudia Renata dos Santos Barros, David Schlesinger, Debora Botequio Moretti, Elaine Cristina Marqueze, Elaine Vieira Santos, Evandra Strazza Rodrigues, Heidge Fukumasu, Jayme Augusto de Souza-Neto, José Salvatore Leister Patané, Luiz Alcantara, Luiz Lehmann Coutinho, Maria Carolina Elias, Maurício Lacerda Nogueira, Rafael dos Santos Bezerra, Raul Machado Neto, Rejane Maria Tommasini Grotto, Ricardo Haddad, Sandra Coccuzzo Sampaio Vessoni, Simone Kashima, Svetoslav Nanev Slavov, Vincent Louis Viala
EPI_ISL_2345711, EPI_ISL_2345725	UPA DR AKIRA TADA	Instituto Butantan / Mendelics	Dimas Tadeu Covas, Antonio Jorge Martins, Claudia Renata dos Santos Barros, David Schlesinger, Debora Botequio Moretti, Elaine Cristina Marqueze, Elaine Vieira Santos, Evandra Strazza Rodrigues, Heidge Fukumasu, Jayme Augusto de Souza-Neto, José Salvatore Leister Patané, Luiz Alcantara, Luiz Lehmann Coutinho, Maria Carolina Elias, Maurício Lacerda Nogueira, Rafael dos Santos Bezerra, Raul Machado Neto, Rejane Maria Tommasini Grotto, Ricardo Haddad, Sandra Coccuzzo Sampaio Vessoni, Simone Kashima, Svetoslav Nanev Slavov, Vincent Louis Viala
EPI_ISL_2345755	SECRETARIA MUNICIPAL DE SAUDE SOROCABA	Instituto Butantan / Mendelics	Dimas Tadeu Covas, Antonio Jorge Martins, Claudia Renata dos Santos Barros, David Schlesinger, Debora Botequio Moretti, Elaine Cristina Marqueze, Elaine Vieira Santos, Evandra Strazza Rodrigues, Heidge Fukumasu, Jayme Augusto de Souza-Neto, José Salvatore Leister Patané, Luiz Alcantara, Luiz Lehmann Coutinho, Maria Carolina Elias, Maurício Lacerda Nogueira, Rafael dos Santos Bezerra, Raul Machado Neto, Rejane Maria Tommasini Grotto, Ricardo Haddad, Sandra Coccuzzo Sampaio Vessoni, Simone Kashima, Svetoslav Nanev Slavov, Vincent Louis Viala
EPI_ISL_2345760	SECRETARIA DE SAUDE PUBLICA DE PRAIA GRANDE	Instituto Butantan / Mendelics	Dimas Tadeu Covas, Antonio Jorge Martins, Claudia Renata dos Santos Barros, David Schlesinger, Debora Botequio Moretti, Elaine Cristina Marqueze, Elaine Vieira Santos, Evandra Strazza Rodrigues, Heidge Fukumasu, Jayme Augusto de Souza-Neto, José Salvatore Leister Patané, Luiz Alcantara, Luiz Lehmann Coutinho, Maria Carolina Elias, Maurício Lacerda Nogueira, Rafael dos Santos Bezerra, Raul Machado Neto, Rejane Maria Tommasini Grotto, Ricardo Haddad, Sandra Coccuzzo Sampaio Vessoni, Simone Kashima, Svetoslav Nanev Slavov, Vincent Louis Viala
EPI_ISL_2345794	CS II DR MIGUEL VITALIANO ORLANDIA	Instituto Butantan / Mendelics	Dimas Tadeu Covas, Antonio Jorge Martins, Claudia Renata dos Santos Barros, David Schlesinger, Debora Botequio Moretti, Elaine Cristina Marqueze, Elaine Vieira Santos, Evandra Strazza Rodrigues, Heidge Fukumasu, Jayme Augusto de Souza-Neto, José Salvatore Leister Patané, Luiz Alcantara, Luiz Lehmann Coutinho, Maria Carolina Elias, Maurício Lacerda Nogueira, Rafael dos Santos Bezerra, Raul Machado Neto, Rejane Maria Tommasini Grotto, Ricardo Haddad, Sandra Coccuzzo Sampaio Vessoni, Simone Kashima, Svetoslav Nanev Slavov, Vincent Louis Viala
EPI_ISL_2345870	HOSPITAL DE CAMPANHA COVID 19 MUNICIPIO DE TAUBATE	Instituto Butantan / Mendelics	Dimas Tadeu Covas, Antonio Jorge Martins, Claudia Renata dos Santos Barros, David Schlesinger, Debora Botequio Moretti, Elaine Cristina Marqueze, Elaine Vieira Santos, Evandra Strazza Rodrigues, Heidge Fukumasu, Jayme Augusto de Souza-Neto, José Salvatore Leister Patané, Luiz Alcantara, Luiz Lehmann Coutinho, Maria Carolina Elias, Maurício Lacerda Nogueira, Rafael dos Santos Bezerra, Raul Machado Neto, Rejane Maria Tommasini Grotto, Ricardo Haddad, Sandra Coccuzzo Sampaio Vessoni, Simone Kashima, Svetoslav Nanev Slavov, Vincent Louis Viala
EPI_ISL_2345874	CENTRO DE APOIO EPIDEMIOLOGICO	Instituto Butantan / Mendelics	Dimas Tadeu Covas, Antonio Jorge Martins, Claudia Renata dos Santos Barros, David Schlesinger, Debora Botequio Moretti, Elaine Cristina Marqueze, Elaine Vieira Santos, Evandra Strazza Rodrigues, Heidge Fukumasu, Jayme Augusto de Souza-Neto, José Salvatore Leister Patané, Luiz Alcantara, Luiz Lehmann Coutinho, Maria Carolina Elias, Maurício Lacerda Nogueira, Rafael dos Santos Bezerra, Raul Machado Neto, Rejane Maria Tommasini Grotto, Ricardo Haddad, Sandra Coccuzzo Sampaio Vessoni, Simone Kashima, Svetoslav Nanev Slavov, Vincent Louis Viala
EPI_ISL_2345885	VIGILANCIA SANITARIA E VIG EPIDEMIOLOGICA DE ITAPEVI	Instituto Butantan / FZEA-USP-Pirassununga	Dimas Tadeu Covas, Antonio Jorge Martins, Claudia Renata dos Santos Barros, David Schlesinger, Debora Botequio Moretti, Elaine Cristina Marqueze, Elaine Vieira Santos, Evandra Strazza Rodrigues, Heidge Fukumasu, Jayme Augusto de Souza-Neto, José Salvatore Leister Patané, Luiz Alcantara, Luiz Lehmann Coutinho, Maria Carolina Elias, Maurício Lacerda Nogueira, Rafael dos Santos Bezerra, Raul Machado Neto, Rejane Maria Tommasini Grotto, Ricardo Haddad, Sandra Coccuzzo Sampaio Vessoni, Simone Kashima, Svetoslav Nanev Slavov, Vincent Louis Viala
EPI_ISL_2345893	UBS DO CIPO EMBU GUACU	Instituto Butantan / FZEA-USP-Pirassununga	Dimas Tadeu Covas, Antonio Jorge Martins, Claudia Renata dos Santos Barros, David Schlesinger, Debora Botequio Moretti, Elaine Cristina Marqueze, Elaine Vieira Santos, Evandra Strazza Rodrigues, Heidge Fukumasu, Jayme Augusto de Souza-Neto, José Salvatore Leister Patané, Luiz Alcantara, Luiz Lehmann Coutinho, Maria Carolina Elias, Maurício Lacerda Nogueira, Rafael dos Santos Bezerra, Raul Machado Neto, Rejane Maria Tommasini Grotto, Ricardo Haddad, Sandra Coccuzzo Sampaio Vessoni, Simone Kashima, Svetoslav Nanev Slavov, Vincent Louis Viala
EPI_ISL_2345896	LABORATORIO LOCAL DE ITAPECERICA DA SERRA	Instituto Butantan / FZEA-USP-Pirassununga	Dimas Tadeu Covas, Antonio Jorge Martins, Claudia Renata dos Santos Barros, David Schlesinger, Debora Botequio Moretti, Elaine Cristina Marqueze, Elaine Vieira Santos, Evandra Strazza Rodrigues, Heidge Fukumasu, Jayme Augusto de Souza-Neto, José Salvatore Leister Patané, Luiz Alcantara, Luiz

			Lehmann Coutinho, Maria Carolina Elias, Maurício Lacerda Nogueira, Rafael dos Santos Bezerra, Raul Machado Neto, Rejane Maria Tommasini Grotto, Ricardo Haddad, Sandra Coccuzzo Sampaio Vessoni, Simone Kashima, Svetoslav Nanev Slavov, Vincent Louis Viala
EPI_ISL_2345901	UBS SAO LOURENCO DA SERRA	Instituto Butantan / FZEA-USP-Pirassununga	Dimas Tadeu Covas, Antonio Jorge Martins, Claudia Renata dos Santos Barros, David Schlesinger, Debora Botequio Moretti, Elaine Cristina Marqueze, Elaine Vieira Santos, Evandra Strazza Rodrigues, Heidge Fukumasu, Jayme Augusto de Souza-Neto, José Salvatore Leister Patané, Luiz Alcantara, Luiz Lehmann Coutinho, Maria Carolina Elias, Maurício Lacerda Nogueira, Rafael dos Santos Bezerra, Raul Machado Neto, Rejane Maria Tommasini Grotto, Ricardo Haddad, Sandra Coccuzzo Sampaio Vessoni, Simone Kashima, Svetoslav Nanev Slavov, Vincent Louis Viala
EPI_ISL_2345926	Prefeirtura de SP	Instituto Butantan	Dimas Tadeu Covas, Antonio Jorge Martins, Claudia Renata dos Santos Barros, David Schlesinger, Debora Botequio Moretti, Elaine Cristina Marqueze, Elaine Vieira Santos, Evandra Strazza Rodrigues, Heidge Fukumasu, Jayme Augusto de Souza-Neto, José Salvatore Leister Patané, Luiz Alcantara, Luiz Lehmann Coutinho, Maria Carolina Elias, Maurício Lacerda Nogueira, Rafael dos Santos Bezerra, Raul Machado Neto, Rejane Maria Tommasini Grotto, Ricardo Haddad, Sandra Coccuzzo Sampaio Vessoni, Simone Kashima, Svetoslav Nanev Slavov, Vincent Louis Viala
EPI_ISL_2345940	AMBULATORIO MEDICO MUNICIPAL DE AGUDOS	Instituto Butantan / ESALQ-Piracicaba	Dimas Tadeu Covas, Antonio Jorge Martins, Claudia Renata dos Santos Barros, David Schlesinger, Debora Botequio Moretti, Elaine Cristina Marqueze, Elaine Vieira Santos, Evandra Strazza Rodrigues, Heidge Fukumasu, Jayme Augusto de Souza-Neto, José Salvatore Leister Patané, Luiz Alcantara, Luiz Lehmann Coutinho, Maria Carolina Elias, Maurício Lacerda Nogueira, Rafael dos Santos Bezerra, Raul Machado Neto, Rejane Maria Tommasini Grotto, Ricardo Haddad, Sandra Coccuzzo Sampaio Vessoni, Simone Kashima, Svetoslav Nanev Slavov, Vincent Louis Viala
EPI_ISL_2345965	PA NOVO OSASCO	Instituto Butantan	Dimas Tadeu Covas, Antonio Jorge Martins, Claudia Renata dos Santos Barros, David Schlesinger, Debora Botequio Moretti, Elaine Cristina Marqueze, Elaine Vieira Santos, Evandra Strazza Rodrigues, Heidge Fukumasu, Jayme Augusto de Souza-Neto, José Salvatore Leister Patané, Luiz Alcantara, Luiz Lehmann Coutinho, Maria Carolina Elias, Maurício Lacerda Nogueira, Rafael dos Santos Bezerra, Raul Machado Neto, Rejane Maria Tommasini Grotto, Ricardo Haddad, Sandra Coccuzzo Sampaio Vessoni, Simone Kashima, Svetoslav Nanev Slavov, Vincent Louis Viala
EPI_ISL_2345973	VIGILANCIA SANITARIA E VIG EPIDEMIOLOGICA DE ITAPEVI	Instituto Butantan	Dimas Tadeu Covas, Antonio Jorge Martins, Claudia Renata dos Santos Barros, David Schlesinger, Debora Botequio Moretti, Elaine Cristina Marqueze, Elaine Vieira Santos, Evandra Strazza Rodrigues, Heidge Fukumasu, Jayme Augusto de Souza-Neto, José Salvatore Leister Patané, Luiz Alcantara, Luiz Lehmann Coutinho, Maria Carolina Elias, Maurício Lacerda Nogueira, Rafael dos Santos Bezerra, Raul Machado Neto, Rejane Maria Tommasini Grotto, Ricardo Haddad, Sandra Coccuzzo Sampaio Vessoni, Simone Kashima, Svetoslav Nanev Slavov, Vincent Louis Viala
EPI_ISL_2346028	UBS MORRO BRANCO	Instituto Butantan	Dimas Tadeu Covas, Antonio Jorge Martins, Claudia Renata dos Santos Barros, David Schlesinger, Debora Botequio Moretti, Elaine Cristina Marqueze, Elaine Vieira Santos, Evandra Strazza Rodrigues, Heidge Fukumasu, Jayme Augusto de Souza-Neto, José Salvatore Leister Patané, Luiz Alcantara, Luiz Lehmann Coutinho, Maria Carolina Elias, Maurício Lacerda Nogueira, Rafael dos Santos Bezerra, Raul Machado Neto, Rejane Maria Tommasini Grotto, Ricardo Haddad, Sandra Coccuzzo Sampaio Vessoni, Simone Kashima, Svetoslav Nanev Slavov, Vincent Louis Viala
EPI_ISL_2346033	UBS PARQUE MARENGO	Instituto Butantan	Dimas Tadeu Covas, Antonio Jorge Martins, Claudia Renata dos Santos Barros, David Schlesinger, Debora Botequio Moretti, Elaine Cristina Marqueze, Elaine Vieira Santos, Evandra Strazza Rodrigues, Heidge Fukumasu, Jayme Augusto de Souza-Neto, José Salvatore Leister Patané, Luiz Alcantara, Luiz Lehmann Coutinho, Maria Carolina Elias, Maurício Lacerda Nogueira, Rafael dos Santos Bezerra, Raul Machado Neto, Rejane Maria Tommasini Grotto, Ricardo Haddad, Sandra Coccuzzo Sampaio Vessoni, Simone Kashima, Svetoslav Nanev Slavov, Vincent Louis Viala
EPI_ISL_2346036, EPI_ISL_2346038	UPA DR FRANCO DA ROCHA	Instituto Butantan	Dimas Tadeu Covas, Antonio Jorge Martins, Claudia Renata dos Santos Barros, David Schlesinger, Debora Botequio Moretti, Elaine Cristina Marqueze, Elaine Vieira Santos, Evandra Strazza Rodrigues, Heidge Fukumasu, Jayme Augusto de Souza-Neto, José Salvatore Leister Patané, Luiz Alcantara, Luiz Lehmann Coutinho, Maria Carolina Elias, Maurício Lacerda Nogueira, Rafael dos Santos Bezerra, Raul Machado Neto, Rejane Maria Tommasini Grotto, Ricardo Haddad, Sandra Coccuzzo Sampaio Vessoni, Simone Kashima, Svetoslav Nanev Slavov, Vincent Louis Viala
EPI_ISL_2346041	UBS JARDIM MARAGOGIPE	Instituto Butantan	Dimas Tadeu Covas, Antonio Jorge Martins, Claudia Renata dos Santos Barros, David Schlesinger, Debora Botequio Moretti, Elaine Cristina Marqueze, Elaine Vieira Santos, Evandra Strazza Rodrigues, Heidge Fukumasu, Jayme Augusto de Souza-Neto, José Salvatore Leister Patané, Luiz Alcantara, Luiz Lehmann Coutinho, Maria Carolina Elias, Maurício Lacerda Nogueira, Rafael dos Santos Bezerra, Raul Machado Neto, Rejane Maria Tommasini Grotto, Ricardo Haddad, Sandra Coccuzzo Sampaio Vessoni, Simone Kashima, Svetoslav Nanev Slavov, Vincent Louis Viala
EPI_ISL_2346042	UBS JARDIM CAIUBY	Instituto Butantan	Dimas Tadeu Covas, Antonio Jorge Martins, Claudia Renata dos Santos Barros, David Schlesinger, Debora Botequio Moretti, Elaine Cristina Marqueze, Elaine Vieira Santos, Evandra Strazza Rodrigues, Heidge Fukumasu, Jayme Augusto de Souza-Neto, José Salvatore Leister Patané, Luiz Alcantara, Luiz Lehmann Coutinho, Maria Carolina Elias, Maurício Lacerda Nogueira, Rafael dos Santos Bezerra, Raul Machado Neto, Rejane Maria Tommasini Grotto, Ricardo Haddad, Sandra Coccuzzo Sampaio Vessoni, Simone Kashima, Svetoslav Nanev Slavov, Vincent Louis Viala
EPI_ISL_2346072	SERRANA	Instituto Butantan / Mendelics	Dimas Tadeu Covas, Antonio Jorge Martins, Claudia Renata dos Santos Barros, David Schlesinger, Debora Botequio Moretti, Elaine Cristina Marqueze, Elaine Vieira Santos, Evandra Strazza Rodrigues, Heidge Fukumasu, Jayme Augusto de Souza-Neto, José Salvatore Leister Patané, Luiz Alcantara, Luiz Lehmann Coutinho, Maria Carolina Elias, Maurício Lacerda Nogueira, Rafael dos Santos Bezerra, Raul Machado Neto, Rejane Maria Tommasini Grotto, Ricardo Haddad, Sandra Coccuzzo Sampaio Vessoni, Simone Kashima, Svetoslav Nanev Slavov, Vincent Louis Viala
EPI_ISL_2346088, EPI_ISL_2346089, EPI_ISL_2346090, EPI_ISL_2346091, EPI_ISL_2346092, EPI_ISL_2346093, EPI_ISL_2346098	Prefeirtura de SP	Instituto Butantan	Dimas Tadeu Covas, Antonio Jorge Martins, Claudia Renata dos Santos Barros, David Schlesinger, Debora Botequio Moretti, Elaine Cristina Marqueze, Elaine Vieira Santos, Evandra Strazza Rodrigues, Heidge Fukumasu, Jayme Augusto de Souza-Neto, José Salvatore Leister Patané, Luiz Alcantara, Luiz Lehmann Coutinho, Maria Carolina Elias, Maurício Lacerda Nogueira, Rafael dos Santos Bezerra, Raul Machado Neto, Rejane Maria Tommasini Grotto, Ricardo Haddad, Sandra Coccuzzo Sampaio Vessoni, Simone Kashima, Svetoslav Nanev Slavov, Vincent Louis Viala
EPI_ISL_2348599, EPI_ISL_2348611, EPI_ISL_2348614	HLAGYN - Laboratorio de Imunologia de Transplantes de Goias	HLAGYN - Laboratorio de Imunologia de Transplantes de Goias	Fernando Antonio Vinhal dos Santos, Erika Lopes Rocha Batista, Alessandro Leonardo Alvares Magalhaes, Frederico Rodrigues Vinhal, Sabrina Sara Moreira Duarte, Lucas Carlos Gomes Pereira, Daniel Ferreira de Sousa
EPI_ISL_2348790	Instituto Nacional De Investigación En Salud Pública-Crn De Influenza Y Otros Virus Respiratorios	NIC-Instituto Nacional de Investigación en Salud Pública	Alfredo Bruno , Maritza Olmedo, Michelle Páez, Jimmy Garcés, Johanna Laines, Lizbeth Patiño, Manuel Gonzalez, Domenica de Mora.
EPI_ISL_2348849, EPI_ISL_2348868	Cliniques universitaires Saint-Luc	UCLouvain/IREC/MBLG	Jean Ruelle, Ophélie Simon, Nicolas Pinte, Benoit Kabamba Mukadi
EPI_ISL_2350401	CSL, Maryland Department of Health	CSL, Maryland Department of Health	Keller,E., Washington,Y.
EPI_ISL_2362237	DIRETORIA MUNICIPAL DE SAUDE DE ENGENHEIRO COELHO	Instituto Butantan / FZEA-USP-Pirassununga	Dimas Tadeu Covas, Antonio Jorge Martins, Claudia Renata dos Santos Barros, David Schlesinger, Debora Botequio Moretti, Elaine Cristina Marqueze, Elaine Vieira Santos, Evandra Strazza Rodrigues, Heidge Fukumasu, Jayme Augusto de Souza-Neto, José Salvatore Leister Patané, Luiz Alcantara, Luiz Lehmann Coutinho, Maria Carolina Elias, Maurício Lacerda Nogueira, Rafael dos Santos Bezerra, Raul Machado Neto, Rejane Maria Tommasini Grotto, Ricardo Haddad, Sandra Coccuzzo Sampaio Vessoni, Simone Kashima, Svetoslav Nanev Slavov, Vincent Louis Viala
EPI_ISL_2362241	LABORATORIO DR PAULO EMILIO DALESSANDRO PINDAMONHANGABA	Instituto Butantan / Mendelics	Dimas Tadeu Covas, Antonio Jorge Martins, Claudia Renata dos Santos Barros, David Schlesinger, Debora Botequio Moretti, Elaine Cristina Marqueze, Elaine Vieira Santos, Evandra Strazza Rodrigues, Heidge Fukumasu, Jayme Augusto de Souza-Neto, José Salvatore Leister Patané, Luiz Alcantara, Luiz Lehmann Coutinho, Maria Carolina Elias, Maurício Lacerda Nogueira, Rafael dos Santos Bezerra, Raul Machado Neto, Rejane Maria Tommasini Grotto, Ricardo Haddad, Sandra Coccuzzo Sampaio Vessoni, Simone Kashima, Svetoslav Nanev Slavov, Vincent Louis Viala
EPI_ISL_2362252	SECRETARIA DE SAUDE	Instituto Butantan / Mendelics	Dimas Tadeu Covas, Antonio Jorge Martins, Claudia Renata dos Santos Barros, David Schlesinger, Debora Botequio Moretti, Elaine Cristina Marqueze, Elaine Vieira Santos, Evandra Strazza Rodrigues, Heidge Fukumasu, Jayme Augusto de Souza-Neto, José Salvatore Leister Patané, Luiz Alcantara, Luiz Lehmann Coutinho, Maria Carolina Elias, Maurício Lacerda Nogueira, Rafael dos Santos Bezerra, Raul Machado Neto, Rejane Maria Tommasini Grotto, Ricardo Haddad, Sandra Coccuzzo Sampaio Vessoni, Simone Kashima, Svetoslav Nanev Slavov, Vincent Louis Viala
EPI_ISL_2362259	HOSPITAL MUNICIPAL DR WALDEMAR TEBALDI	Instituto Butantan / Mendelics	Dimas Tadeu Covas, Antonio Jorge Martins, Claudia Renata dos Santos Barros, David Schlesinger, Debora Botequio Moretti, Elaine Cristina Marqueze, Elaine Vieira Santos, Evandra Strazza Rodrigues, Heidge Fukumasu, Jayme Augusto de Souza-Neto, José Salvatore Leister Patané, Luiz Alcantara, Luiz Lehmann Coutinho, Maria Carolina Elias, Maurício Lacerda Nogueira, Rafael dos Santos Bezerra, Raul Machado Neto, Rejane Maria Tommasini Grotto, Ricardo Haddad, Sandra Coccuzzo Sampaio Vessoni, Simone Kashima, Svetoslav Nanev Slavov, Vincent Louis Viala
EPI_ISL_2362262	CENTRO DE SAUDE II DR GABRIEL MESQUITA VARGEM GDE DO SUL	Instituto Butantan / Mendelics	Dimas Tadeu Covas, Antonio Jorge Martins, Claudia Renata dos Santos Barros, David Schlesinger, Debora Botequio Moretti, Elaine Cristina Marqueze, Elaine Vieira Santos, Evandra Strazza Rodrigues, Heidge Fukumasu, Jayme Augusto de Souza-Neto, José Salvatore Leister Patané, Luiz Alcantara, Luiz Lehmann Coutinho, Maria Carolina Elias, Maurício Lacerda Nogueira, Rafael dos Santos Bezerra, Raul Machado Neto, Rejane Maria Tommasini Grotto, Ricardo Haddad, Sandra Coccuzzo Sampaio Vessoni, Simone Kashima, Svetoslav Nanev Slavov, Vincent Louis Viala

EPI_ISL_2362263	LABORATORIO DR PAULO EMILIO DALESSANDRO PINDAMONHANGABA	Instituto Butantan / Mendelics	Dimas Tadeu Covas, Antonio Jorge Martins, Claudia Renata dos Santos Barros, David Schlesinger, Debora Botequio Moretti, Elaine Cristina Marqueze, Elaine Vieira Santos, Evandra Strazza Rodrigues, Heidge Fukumasu, Jayme Augusto de Souza-Neto, José Salvatore Leister Patané, Luiz Alcantara, Luiz Lehmann Coutinho, Maria Carolina Elias, Maurício Lacerda Nogueira, Rafael dos Santos Bezerra, Raul Machado Neto, Rejane Maria Tommasini Grotto, Ricardo Haddad, Sandra Coccuzzo Sampaio Vessoni, Simone Kashima, Svetoslav Nanev Slavov, Vincent Louis Viala
EPI_ISL_2362266	HOSPITAL DE CAMPANHA COVID 19 CAIEIRAS	Instituto Butantan / Mendelics	Dimas Tadeu Covas, Antonio Jorge Martins, Claudia Renata dos Santos Barros, David Schlesinger, Debora Botequio Moretti, Elaine Cristina Marqueze, Elaine Vieira Santos, Evandra Strazza Rodrigues, Heidge Fukumasu, Jayme Augusto de Souza-Neto, José Salvatore Leister Patané, Luiz Alcantara, Luiz Lehmann Coutinho, Maria Carolina Elias, Maurício Lacerda Nogueira, Rafael dos Santos Bezerra, Raul Machado Neto, Rejane Maria Tommasini Grotto, Ricardo Haddad, Sandra Coccuzzo Sampaio Vessoni, Simone Kashima, Svetoslav Nanev Slavov, Vincent Louis Viala
EPI_ISL_2362610	Platform BIS UZA/UAntwerpen	Labo Klinische Biologie, UZA	Marie Le Mercier, Jasmine Coppens, Basil Britto Xavier, Christine Lammens, Veerle Matheeussen, Herman Goossens
EPI_ISL_2363545, EPI_ISL_2363547	Laboratorio Central de la Ciudad de Santa Fe	Grupo de Genómica y Bioinformática del Instituto de Investigación de la Cadena Láctea CONICET-INTA on behalf of 'Proyecto Argentino Interinstitucional de genomica de SARS-CoV-2' (PAIS Consortium)	Eberhardt, MF; Irazoqui, JM; Ojeda, G; Rompato, G; Mugna, V; Pastor, C; Amadio, AF
EPI_ISL_2363555, EPI_ISL_2363558	Hospital Jaime Ferre - SAMCO Rafaela	Grupo de Genómica y Bioinformática del Instituto de Investigación de la Cadena Láctea CONICET-INTA on behalf of 'Proyecto Argentino Interinstitucional de genomica de SARS-CoV-2' (PAIS Consortium)	Eberhardt, MF; Irazoqui, JM; Pandolfi, V; Quaranta, JF; Isaia, C; Amadio, AF
EPI_ISL_2363559, EPI_ISL_2363560, EPI_ISL_2363561	Laboratorio Central de la Ciudad de Santa Fe	Grupo de Genómica y Bioinformática del Instituto de Investigación de la Cadena Láctea CONICET-INTA on behalf of 'Proyecto Argentino Interinstitucional de genomica de SARS-CoV-2' (PAIS Consortium)	Eberhardt, MF; Irazoqui, JM; Ojeda, G; Rompato, G; Mugna, V; Pastor, C; Amadio, AF
EPI_ISL_2363562, EPI_ISL_2363563, EPI_ISL_2363564, EPI_ISL_2363565, EPI_ISL_2363566	Hospital Jaime Ferre - SAMCO Rafaela	Grupo de Genómica y Bioinformática del Instituto de Investigación de la Cadena Láctea CONICET-INTA on behalf of 'Proyecto Argentino Interinstitucional de genomica de SARS-CoV-2' (PAIS Consortium)	Eberhardt, MF; Irazoqui, JM; Pandolfi, V; Quaranta, JF; Isaia, C; Amadio, AF
EPI_ISL_2363568, EPI_ISL_2363569, EPI_ISL_2363570, EPI_ISL_2363573	Laboratorio Central de la Ciudad de Santa Fe	Grupo de Genómica y Bioinformática del Instituto de Investigación de la Cadena Láctea CONICET-INTA on behalf of 'Proyecto Argentino Interinstitucional de genomica de SARS-CoV-2' (PAIS Consortium)	Eberhardt, MF; Irazoqui, JM; Ojeda, G; Rompato, G; Mugna, V; Pastor, C; Amadio, AF
EPI_ISL_2365892	Microbiology Department. Complexo Hospitalario Universitario de Vigo	Microbiology Department. Complexo Hospitalario Universitario de Vigo	Alfaya N, Alonso I, Alvarez M, Cabrera JJ, Carballo R, Cores O, Cortizo S, del-Campo V, Martinez L, Mediero G, Perez S, Potel C, Regueiro B, Rey S, Vasallo FJ
EPI_ISL_2367609, EPI_ISL_2367628	Helix/Illumina	Centers for Disease Control and Prevention Division of Viral Diseases, Pathogen Discovery	Dakota Howard, Dhwani Batra, Peter W. Cook, Kara Moser, Adrian Paskey, Jason Caravas, Benjamin Rambo-Martin, Shatavia Morrison, Christopher Gulvick, Scott Sammons, Yvette Unoarumhi, Darlene Wagner, Matthew Schmerer, Eileen de Feo, Jan Antico, Christine Tran, Matthew Tolentino, Shannon Wickline, Kim Gietzen, Brad Sickler, Jingtao Liu, Eric Allen, Phil Febbo, Nicole L. Washington, Simon White, Geraint Levan, Kelly Schiabor Barrett, Elizabeth Cirulli, Alexandre Bolze, Ary Ascencio, Charlotte Rivera-Garcia, Ryan Cho, Jason Nguyen, Sherry Wang, Jimmy Ramirez, Tyler Cassens, Efren Sandoval, Magnus Isaksson, William Lee, David Becker, Marc Laurent, James Lu, Clinton R. Paden, Duncan MacCannell
EPI_ISL_2368918, EPI_ISL_2369972, EPI_ISL_2370627, EPI_ISL_2370713	Aegis Sciences Corporation	Centers for Disease Control and Prevention Division of Viral Diseases, Pathogen Discovery	Dakota Howard, Dhwani Batra, Peter W. Cook, Kara Moser, Adrian Paskey, Jason Caravas, Benjamin Rambo-Martin, Shatavia Morrison, Christopher Gulvick, Scott Sammons, Yvette Unoarumhi, Darlene Wagner, Matthew Schmerer, Cyndi Clark, Patrick Campbell, Rob Case, Vikramsinha Ghorpade, Holly Houdeshell, Ola Kvalvaag, Dillon Nall, Ethan Sanders, Alec Vest, Shaun Westlund, Matthew Hardison, Clinton R. Paden, Duncan MacCannell
EPI_ISL_2371700	Quest Diagnostics Incorporated	Centers for Disease Control and Prevention Division of Viral Diseases, Pathogen Discovery	Dakota Howard, Dhwani Batra, Peter W. Cook, Kara Moser, Adrian Paskey, Jason Caravas, Benjamin Rambo-Martin, Shatavia Morrison, Christopher Gulvick, Scott Sammons, Yvette Unoarumhi, Darlene Wagner, Matthew Schmerer, S. H. Rosenthal, A. Gerasimova, R. M. Kagan, B. Anderson, M. Hua, Y. Liu, L.E. Bernstein, K.E. Livingston, A. Perez, I. A. Shlyakhter, R. V. Rolando, R. Owen, P. Tanpaiboon, F. Lacbawan, Clinton R. Paden, Duncan MacCannell
EPI_ISL_2372690	Microbiology Department, Laboratori Clínic Metropolitana Nord. Hospital Universitari Germans Trias i Pujol	Can Ruti SARS-CoV-2 Sequencing Hub (HUGTiP/IrsiCaixa/IGTP)	Marc Noguera-Julian, Pilar Armengol, Ignacio Blanco, Antoni E Bordoy, Francesc Catala-Moll, Pere-Joan Cardona, Maria Casadellà, Cristina Casañ, Gemma Clara, Bonaventura Clotet, Cristina Esteban, Montserrat Giménez, Mercedes Guerrero, Anna Not, Roger Pared
EPI_ISL_2372900, EPI_ISL_2372914, EPI_ISL_2372921, EPI_ISL_2372946, EPI_ISL_2372952	Quest Diagnostics Incorporated	Centers for Disease Control and Prevention Division of Viral Diseases, Pathogen Discovery	Dakota Howard, Dhwani Batra, Peter W. Cook, Kara Moser, Adrian Paskey, Jason Caravas, Benjamin Rambo-Martin, Shatavia Morrison, Christopher Gulvick, Scott Sammons, Yvette Unoarumhi, Darlene Wagner, Matthew Schmerer, S. H. Rosenthal, A. Gerasimova, R. M. Kagan, B. Anderson, M. Hua, Y. Liu, L.E. Bernstein, K.E. Livingston, A. Perez, I. A. Shlyakhter, R. V. Rolando, R. Owen, P. Tanpaiboon, F. Lacbawan, Clinton R. Paden, Duncan MacCannell
EPI_ISL_2374120	Hospital General Universitario de Alicante - Instituto de Investigación Sanitaria y Biomédica de Alicante	SeqCOVID-SPAIN consortium/IBV(CSIC)	Maripaz Ventero Martín, Carmen Molina Pardines and SeqCOVID-SPAIN consortium
EPI_ISL_2375845	Laboratório de Microbiologia Molecular - Universidade FEEVALE	Molecular Microbiology Laboratory	Alana Witt Hansen, Fágner Henrique Heldt, Fernando Rosado Spilki, Flávio Silveira, Juliana Schons Gularte, Juliane Deise Fleck, Mariana Soares da Silva, Meriane Demoliner, Matheus Nunes Weber, Paula Rodrigues de Almeida, Micheli Filippi.
EPI_ISL_2376089	Alaska State Virology Laboratory	Alaska State Virology Laboratory	Stephanie DeRonde, Elva House, Jacob Zidek, Lisa Smith, Ph.D., Jack Chen, Ph.D.
EPI_ISL_2376106	NJDOH, Public Health and Environmental Laboratories	NJ_PHEL	Lindsey Bodnar, Shiv K. Verma, Jacquelyn Deverell, Dana Woell, Allison Roder, Byeong Jeong
EPI_ISL_2376215	Heilig hart Lier	Imeda Hospital	Johan Frans, Dagmar Obbels, Hanne Valgaeren
EPI_ISL_2376267	Wexner Medical Center	The Ohio State University College of Medicine	Koenig,S., Seminetta,J.
EPI_ISL_2378740	UPA DE BEBEDOURO	Instituto Butantan	Dimas Tadeu Covas, Antonio Jorge Martins, Claudia Renata dos Santos Barros, David Schlesinger, Debora Botequio Moretti, Elaine Cristina Marqueze, Elaine Vieira Santos, Evandra Strazza Rodrigues, Heidge Fukumasu, Jayme Augusto de Souza-Neto, José Salvatore Leister Patané, Luiz Alcantara, Luiz Lehmann Coutinho, Maria Carolina Elias, Maurício Lacerda Nogueira, Rafael dos Santos Bezerra, Raul Machado Neto, Rejane Maria Tommasini Grotto, Ricardo Haddad, Sandra Coccuzzo Sampaio Vessoni, Simone Kashima, Svetoslav Nanev Slavov, Vincent Louis Viala
EPI_ISL_2378742	UNIDADE DE SAUDE DR PHEBO DE OLIVEIRA ROGE FERREIRA	Instituto Butantan	Dimas Tadeu Covas, Antonio Jorge Martins, Claudia Renata dos Santos Barros, David Schlesinger, Debora Botequio Moretti, Elaine Cristina Marqueze, Elaine Vieira Santos, Evandra Strazza Rodrigues, Heidge Fukumasu, Jayme Augusto de Souza-Neto, José Salvatore Leister Patané, Luiz Alcantara, Luiz Lehmann Coutinho, Maria Carolina Elias, Maurício Lacerda Nogueira, Rafael dos Santos Bezerra, Raul Machado Neto, Rejane Maria Tommasini Grotto, Ricardo Haddad, Sandra Coccuzzo Sampaio Vessoni, Simone Kashima, Svetoslav Nanev Slavov, Vincent Louis Viala
EPI_ISL_2385520	Laboratorio Central Noel Nutels	Bioinformatics Laboratory / LNCC	Luiz G P de Almeida, Alessandra P Lamarca, Ronaldo da Silva F Jr, Liliane Cavalcante, Alexandra L Gerber, Ana Paula de C Guimaraes, Douglas Terra Machado, Cassia Alves, Diana Mariani, Cintia Policarpo, Gleidson da Silva de Oliveira, Mario Sergio Ribeiro, Silvia Carvalho, Flavio Dias da Silva, Marcio Henrique de Oliveira Garcia, Leandro Magalhaes de Souza, Cristiane Gomes da Silva, Caio Luiz Pereira Ribeiro, Andrea Cony Cavalcanti, Claudia Maria Braga de Mello, Amilcar Tanuri, Ana Tereza R Vasconcelos
EPI_ISL_2385736	Unidade de apoio ao diagnóstico da COVID - UNADIG	Bioinformatics Laboratory / LNCC	Luiz G P de Almeida, Alessandra P Lamarca, Ronaldo da Silva F Jr, Liliane Cavalcante, Alexandra L Gerber, Ana Paula de C Guimaraes, Douglas Terra Machado, Cassia Alves, Diana Mariani, Cintia Policarpo, Gleidson da Silva de Oliveira, Mario Sergio Ribeiro, Silvia Carvalho, Flavio Dias da Silva, Marcio Henrique de Oliveira Garcia, Leandro Magalhaes de Souza, Cristiane Gomes da Silva, Caio Luiz Pereira Ribeiro, Andrea Cony Cavalcanti, Claudia Maria Braga de Mello, Amilcar Tanuri, Ana Tereza R Vasconcelos
EPI_ISL_2391068	Genetica Molecular and Subdepartamento de Virologia ISP Chile	Instituto de Salud Publica de Chile	Karen Orostica, Constanza Campano, Barbara Parra, Loredana Arata, Gisselle Barra, Patricia Bustos, Rodrigo Fasce, Javier Tognarelli, Andres Castillo, Soledad Ulloa, Jorge Fernandez

EPI_ISL_2406247, EPI_ISL_2406269	Dutch COVID-19 response team	National Institute for Public Health and the Environment (RIVM)	Adam Meijer, Harry Vennema, Dirk Eggink, Jeroen Cremer, Sharon van den Brink, Bas van der Veer, AnneMarie van den Brandt, Lisa Wijsman, Kim Freriks, Ryanne Jaarsma, Euníce Then, Lynn Aarts, Sanne Bos, Melissa van Tuil, Linda van de Nes, Sjoerd Kuiling, James Groot, Florian Zwagemaker, Dennis Schmitz, Annelies Kroneman, Karim Hajji, Chantal Reusken, on behalf of the national COVID-19 response team
EPI_ISL_2421897, EPI_ISL_2421946	Aegis Sciences Corporation	Centers for Disease Control and Prevention Division of Viral Diseases, Pathogen Discovery	Dakota Howard, Dhwani Batra, Peter W. Cook, Kara Moser, Adrian Paskey, Jason Caravas, Benjamin Rambo-Martin, Shatavia Morrison, Christopher Gulvick, Scott Sammons, Yvette Unoarumhi, Darlene Wagner, Matthew Schmerer, Cyndi Clark, Patrick Campbell, Rob Case, Vikramsinha Ghorpade, Holly Houdeshell, Ola Kvalvaag, Dillon Nall, Ethan Sanders, Alec Vest, Shaun Westlund, Matthew Hardison, Clinton R. Paden, Duncan MacCannell
EPI_ISL_2443552	Labortorio Central de Saude Publica do Estado de Santa Catarina (LACEN/SC)	Laboratory of Respiratory Viruses and Measles, Oswaldo Cruz Institute, FIOCRUZ	Paola Resende, Luciana Appolinario, Fernando Motta, Anna Carolina Paixao, Ana Carolina Mendonca, Alice Sampaio Rocha, Taina Venas, Elisa Cavalcante Pereira, Renata Serrano Lopes, Darcita Buerger Rovaris, Sandra Bianchini Fernandes, Marilda Siqueira on behalf of the Fiocruz COVID-19 Genomic Surveillance Network
EPI_ISL_2445239	HOSPITAL MUNICIPAL REYNALDO GUERRA CAJATI	Instituto Butantan	Dimas Tadeu Covas, Antonio Jorge Martins, Claudia Renata dos Santos Barros, David Schlesinger, Debora Botequio Moretti, Elaine Cristina Marqueze, Elaine Vieira Santos, Evandra Strazza Rodrigues, Heidge Fukumasu, Jayme Augusto de Souza-Neto, José Salvatore Leister Patané, Luiz Alcantara, Luiz Lehmann Coutinho, Maria Carolina Elias, Maurício Lacerda Nogueira, Rafael dos Santos Bezerra, Raul Machado Neto, Rejane Maria Tommasini Grotto, Ricardo Haddad, Sandra Coccuzzo Sampaio Vessoni, Simone Kashima, Svetoslav Nanev Slavov, Vincent Louis Viala
EPI_ISL_2445603	CENTRO DE SAUDE II SAO MIGUEL ARCANJO	Instituto Butantan	Dimas Tadeu Covas, Antonio Jorge Martins, Claudia Renata dos Santos Barros, David Schlesinger, Debora Botequio Moretti, Elaine Cristina Marqueze, Elaine Vieira Santos, Evandra Strazza Rodrigues, Heidge Fukumasu, Jayme Augusto de Souza-Neto, José Salvatore Leister Patané, Luiz Alcantara, Luiz Lehmann Coutinho, Maria Carolina Elias, Maurício Lacerda Nogueira, Rafael dos Santos Bezerra, Raul Machado Neto, Rejane Maria Tommasini Grotto, Ricardo Haddad, Sandra Coccuzzo Sampaio Vessoni, Simone Kashima, Svetoslav Nanev Slavov, Vincent Louis Viala
EPI_ISL_2466126, EPI_ISL_2466133, EPI_ISL_2466135	Laboratorio Central de Saude Publica do Estado do Rio Grande do Sul (LACEN-RS)	Laboratory of Respiratory Viruses and Measles, Oswaldo Cruz Institute, FIOCRUZ	Paola Resende, Luciana Appolinario, Fernando Motta, Anna Carolina Paixao, Ana Carolina Mendonca, Alice Sampaio Rocha, Taina Venas, Elisa Cavalcante Pereira, Renata Serrano Lopes, Tatiana Schaffer Gregianini, Richard Salvato, Marilda Siqueira on behalf of the Fiocruz COVID-19 Genomic Surveillance Network
EPI_ISL_2466265	Laboratório de Ecologia de Doenças Transmissíveis na Amazônia (EDTA), Instituto Leônidas e Maria Deane, FIOCRUZ, Manaus, Amazonas, Brazil.	Laboratory of Respiratory Viruses and Measles, Oswaldo Cruz Institute, FIOCRUZ	Paola Resende, Felipe Naveca, Alex Pauvolid-Corrêa, Mia Ferreira de Araujo, Ana Beatriz Machado Lima, Luciana Appolinario, Fernando Motta, Anna Carolina Paixao, Ana Carolina Mendonca, Alice Sampaio Rocha, Taina Venas, Elisa Cavalcante Pereira, Renata Serrano Lopes, Marilda Siqueira on behalf of the Fiocruz COVID-19 Genomic Surveillance Network
EPI_ISL_2480522, EPI_ISL_2481652	Laboratory Corporation of America	Centers for Disease Control and Prevention Division of Viral Diseases, Pathogen Discovery	Dakota Howard, Dhwani Batra, Peter W. Cook, Kara Moser, Adrian Paskey, Jason Caravas, Benjamin Rambo-Martin, Shatavia Morrison, Christopher Gulvick, Scott Sammons, Yvette Unoarumhi, Darlene Wagner, Matthew Schmerer, Minoo Agarwal, Eyad Almasri, Debbie Boles, Ayla Burns, Nuthawin Charoensri, Oren Cohen, Susan Countryman, Mary Ann Cristobal, Bobbi Croy, Suzanne Dale, Hrushikesh Deshmukh, Amanda Douglas, Vincent Drouillon, Marcia Eisenberg, Howard Engler, Rama Ghatti, Prashant Gupta, Susan Hicks, Jake Humphrey, Lax Iyer, Lisa Pfefferle, Manoj Jain, Matthew Robinson, Mohan Kolli, Brian Krueger, Tim Kuphal, Stanley Letovsky, Michael Levandoski, Craig Lukasik, Jonathan Meltzer, Brian Norvell, Mindy Nye, Scott Parker, Christos Petropoulos, John Pruitt, Steven Ragan, Scott Ryan, Mike Sapeta, Jana Schroth, Suresh Babu Selvaraju, Goran Stevovic, Amanda Suchanek, Andrea Throop, Lyndon Tilson, Thomas Urban, Joe Voshell, Kimberly Wagner, Jonathan Williams, Mary Williamson, Qian Zeng, Tricia Zwiefelhofer, Clinton R. Paden, Duncan MacCannell
EPI_ISL_2488772	LACEN - Laboratório Central de Saúde Pública do Roraima	Evandro Chagas Institute	Santos, M.C.; Silva, A.M.; Junior, W.D.C.; Barbagelata, L.S.; Ferreira, J.A.; Sousa, E.M.A.; da Silva, P.S.; Pinheiro, K.C.; L.C.; Sousa Junior, E.C.
EPI_ISL_2493202	BIOFAST	Instituto Butantan	Dimas Tadeu Covas, Antonio Jorge Martins, Claudia Renata dos Santos Barros, David Schlesinger, Debora Botequio Moretti, Elaine Cristina Marqueze, Elaine Vieira Santos, Evandra Strazza Rodrigues, Heidge Fukumasu, Jayme Augusto de Souza-Neto, José Salvatore Leister Patané, Luiz Alcantara, Luiz Lehmann Coutinho, Maria Carolina Elias, Maurício Lacerda Nogueira, Rafael dos Santos Bezerra, Raul Machado Neto, Rejane Maria Tommasini Grotto, Ricardo Haddad, Sandra Coccuzzo Sampaio Vessoni, Simone Kashima, Svetoslav Nanev Slavov, Vincent Louis Viala
EPI_ISL_2493627	SECRETARIA DE SAUDE	Instituto Butantan	Dimas Tadeu Covas, Antonio Jorge Martins, Claudia Renata dos Santos Barros, David Schlesinger, Debora Botequio Moretti, Elaine Cristina Marqueze, Elaine Vieira Santos, Evandra Strazza Rodrigues, Heidge Fukumasu, Jayme Augusto de Souza-Neto, José Salvatore Leister Patané, Luiz Alcantara, Luiz Lehmann Coutinho, Maria Carolina Elias, Maurício Lacerda Nogueira, Rafael dos Santos Bezerra, Raul Machado Neto, Rejane Maria Tommasini Grotto, Ricardo Haddad, Sandra Coccuzzo Sampaio Vessoni, Simone Kashima, Svetoslav Nanev Slavov, Vincent Louis Viala
EPI_ISL_2493820	UBS JARDIM CAIUBY	Instituto Butantan	Dimas Tadeu Covas, Antonio Jorge Martins, Claudia Renata dos Santos Barros, David Schlesinger, Debora Botequio Moretti, Elaine Cristina Marqueze, Elaine Vieira Santos, Evandra Strazza Rodrigues, Heidge Fukumasu, Jayme Augusto de Souza-Neto, José Salvatore Leister Patané, Luiz Alcantara, Luiz Lehmann Coutinho, Maria Carolina Elias, Maurício Lacerda Nogueira, Rafael dos Santos Bezerra, Raul Machado Neto, Rejane Maria Tommasini Grotto, Ricardo Haddad, Sandra Coccuzzo Sampaio Vessoni, Simone Kashima, Svetoslav Nanev Slavov, Vincent Louis Viala
EPI_ISL_2493828	PA DE IBITIUVA DR OTAVIO BENETTI PITANGUEIRAS	Instituto Butantan	Dimas Tadeu Covas, Antonio Jorge Martins, Claudia Renata dos Santos Barros, David Schlesinger, Debora Botequio Moretti, Elaine Cristina Marqueze, Elaine Vieira Santos, Evandra Strazza Rodrigues, Heidge Fukumasu, Jayme Augusto de Souza-Neto, José Salvatore Leister Patané, Luiz Alcantara, Luiz Lehmann Coutinho, Maria Carolina Elias, Maurício Lacerda Nogueira, Rafael dos Santos Bezerra, Raul Machado Neto, Rejane Maria Tommasini Grotto, Ricardo Haddad, Sandra Coccuzzo Sampaio Vessoni, Simone Kashima, Svetoslav Nanev Slavov, Vincent Louis Viala
EPI_ISL_2493854	HOSP MUN DE MOGI DAS CRUZES PREF WALDEMAR COSTA FILHO	Instituto Butantan	Dimas Tadeu Covas, Antonio Jorge Martins, Claudia Renata dos Santos Barros, David Schlesinger, Debora Botequio Moretti, Elaine Cristina Marqueze, Elaine Vieira Santos, Evandra Strazza Rodrigues, Heidge Fukumasu, Jayme Augusto de Souza-Neto, José Salvatore Leister Patané, Luiz Alcantara, Luiz Lehmann Coutinho, Maria Carolina Elias, Maurício Lacerda Nogueira, Rafael dos Santos Bezerra, Raul Machado Neto, Rejane Maria Tommasini Grotto, Ricardo Haddad, Sandra Coccuzzo Sampaio Vessoni, Simone Kashima, Svetoslav Nanev Slavov, Vincent Louis Viala
EPI_ISL_2493990	INSIDE CRSS PARELHEIROS	Instituto Butantan	Dimas Tadeu Covas, Antonio Jorge Martins, Claudia Renata dos Santos Barros, David Schlesinger, Debora Botequio Moretti, Elaine Cristina Marqueze, Elaine Vieira Santos, Evandra Strazza Rodrigues, Heidge Fukumasu, Jayme Augusto de Souza-Neto, José Salvatore Leister Patané, Luiz Alcantara, Luiz Lehmann Coutinho, Maria Carolina Elias, Maurício Lacerda Nogueira, Rafael dos Santos Bezerra, Raul Machado Neto, Rejane Maria Tommasini Grotto, Ricardo Haddad, Sandra Coccuzzo Sampaio Vessoni, Simone Kashima, Svetoslav Nanev Slavov, Vincent Louis Viala
EPI_ISL_2494056	AFIP LESTE	Instituto Butantan	Dimas Tadeu Covas, Antonio Jorge Martins, Claudia Renata dos Santos Barros, David Schlesinger, Debora Botequio Moretti, Elaine Cristina Marqueze, Elaine Vieira Santos, Evandra Strazza Rodrigues, Heidge Fukumasu, Jayme Augusto de Souza-Neto, José Salvatore Leister Patané, Luiz Alcantara, Luiz Lehmann Coutinho, Maria Carolina Elias, Maurício Lacerda Nogueira, Rafael dos Santos Bezerra, Raul Machado Neto, Rejane Maria Tommasini Grotto, Ricardo Haddad, Sandra Coccuzzo Sampaio Vessoni, Simone Kashima, Svetoslav Nanev Slavov, Vincent Louis Viala
EPI_ISL_2494090	AFIP SUL	Instituto Butantan	Dimas Tadeu Covas, Antonio Jorge Martins, Claudia Renata dos Santos Barros, David Schlesinger, Debora Botequio Moretti, Elaine Cristina Marqueze, Elaine Vieira Santos, Evandra Strazza Rodrigues, Heidge Fukumasu, Jayme Augusto de Souza-Neto, José Salvatore Leister Patané, Luiz Alcantara, Luiz Lehmann Coutinho, Maria Carolina Elias, Maurício Lacerda Nogueira, Rafael dos Santos Bezerra, Raul Machado Neto, Rejane Maria Tommasini Grotto, Ricardo Haddad, Sandra Coccuzzo Sampaio Vessoni, Simone Kashima, Svetoslav Nanev Slavov, Vincent Louis Viala
EPI_ISL_2494116	UPA DE BEBEDOURO	Instituto Butantan	Dimas Tadeu Covas, Antonio Jorge Martins, Claudia Renata dos Santos Barros, David Schlesinger, Debora Botequio Moretti, Elaine Cristina Marqueze, Elaine Vieira Santos, Evandra Strazza Rodrigues, Heidge Fukumasu, Jayme Augusto de Souza-Neto, José Salvatore Leister Patané, Luiz Alcantara, Luiz Lehmann Coutinho, Maria Carolina Elias, Maurício Lacerda Nogueira, Rafael dos Santos Bezerra, Raul Machado Neto, Rejane Maria Tommasini Grotto, Ricardo Haddad, Sandra Coccuzzo Sampaio Vessoni, Simone Kashima, Svetoslav Nanev Slavov, Vincent Louis Viala
EPI_ISL_2494175	UNIDADE BASICA DE SAUDE DR MATHEUS GABRIEL BONASSA	Instituto Butantan	Dimas Tadeu Covas, Antonio Jorge Martins, Claudia Renata dos Santos Barros, David Schlesinger, Debora Botequio Moretti, Elaine Cristina Marqueze, Elaine Vieira Santos, Evandra Strazza Rodrigues, Heidge Fukumasu, Jayme Augusto de Souza-Neto, José Salvatore Leister Patané, Luiz Alcantara, Luiz Lehmann Coutinho, Maria Carolina Elias, Maurício Lacerda Nogueira, Rafael dos Santos Bezerra, Raul Machado Neto, Rejane Maria Tommasini Grotto, Ricardo Haddad, Sandra Coccuzzo Sampaio Vessoni, Simone Kashima, Svetoslav Nanev Slavov, Vincent Louis Viala
EPI_ISL_2494202	BIOFAST SUL	Instituto Butantan	Dimas Tadeu Covas, Antonio Jorge Martins, Claudia Renata dos Santos Barros, David Schlesinger, Debora Botequio Moretti, Elaine Cristina Marqueze, Elaine Vieira Santos, Evandra Strazza Rodrigues, Heidge Fukumasu, Jayme Augusto de Souza-Neto, José Salvatore Leister Patané, Luiz Alcantara, Luiz Lehmann Coutinho, Maria Carolina Elias, Maurício Lacerda Nogueira, Rafael dos Santos Bezerra, Raul Machado Neto, Rejane Maria Tommasini Grotto, Ricardo Haddad, Sandra Coccuzzo Sampaio Vessoni, Simone Kashima, Svetoslav Nanev Slavov, Vincent Louis Viala
EPI_ISL_2494282	CENTRO DE SAUDE III DE AGUAS DE SAO PEDRO	Instituto Butantan	Dimas Tadeu Covas, Antonio Jorge Martins, Claudia Renata dos Santos Barros, David Schlesinger, Debora Botequio Moretti, Elaine Cristina Marqueze,

			Elaine Vieira Santos, Evandra Strazza Rodrigues, Heidge Fukumasu, Jayme Augusto de Souza-Neto, José Salvatore Leister Patané, Luiz Alcantara, Luiz Lehmann Coutinho, Maria Carolina Elias, Maurício Lacerda Nogueira, Rafael dos Santos Bezerra, Raul Machado Neto, Rejane Maria Tommasini Grotto, Ricardo Haddad, Sandra Coccuzzo Sampaio Vessoni, Simone Kashima, Svetoslav Nanev Slavov, Vincent Louis Viala
EPI_ISL_2494287	SECRETARIA DE SAUDE DE SAO PEDRO	Instituto Butantan	Dimas Tadeu Covas, Antonio Jorge Martins, Claudia Renata dos Santos Barros, David Schlesinger, Debora Botequio Moretti, Elaine Cristina Marqueze, Elaine Vieira Santos, Evandra Strazza Rodrigues, Heidge Fukumasu, Jayme Augusto de Souza-Neto, José Salvatore Leister Patané, Luiz Alcantara, Luiz Lehmann Coutinho, Maria Carolina Elias, Maurício Lacerda Nogueira, Rafael dos Santos Bezerra, Raul Machado Neto, Rejane Maria Tommasini Grotto, Ricardo Haddad, Sandra Coccuzzo Sampaio Vessoni, Simone Kashima, Svetoslav Nanev Slavov, Vincent Louis Viala
EPI_ISL_2534951	Laboratorio Central Noel Nutels	Bioinformatics Laboratory / LNCC	Luiz G P de Almeida, Alessandra P Lamarca, Ronaldo da Silva F Jr, Liliane Cavalcante, Alexandra L Gerber, Ana Paula de C Guimaraes, Douglas Terra Machado, Cassia Alves, Diana Mariani, Cintia Policarpo, Gleidson da Silva de Oliveira, Mario Sergio Ribeiro, Silvia Carvalho, Flavio Dias da Silva, Marcio Henrique de Oliveira Garcia, Leandro Magalhaes de Souza, Cristiane Gomes da Silva, Caio Luiz Pereira Ribeiro, Andrea Cony Cavalcanti, Claudia Maria Braga de Mello, Amilcar Tanuri, Ana Tereza R Vasconcelos
EPI_ISL_2535219	Unidade de apoio ao diagnostico da COVID - UNADIG	Bioinformatics Laboratory / LNCC	Luiz G P de Almeida, Alessandra P Lamarca, Ronaldo da Silva F Jr, Liliane Cavalcante, Alexandra L Gerber, Ana Paula de C Guimaraes, Douglas Terra Machado, Cassia Alves, Diana Mariani, Cintia Policarpo, Gleidson da Silva de Oliveira, Mario Sergio Ribeiro, Silvia Carvalho, Flavio Dias da Silva, Marcio Henrique de Oliveira Garcia, Leandro Magalhaes de Souza, Cristiane Gomes da Silva, Caio Luiz Pereira Ribeiro, Andrea Cony Cavalcanti, Claudia Maria Braga de Mello, Amilcar Tanuri, Ana Tereza R Vasconcelos
EPI_ISL_2536245, EPI_ISL_2536249	Labortorio Central de Saude Publica do Estado de Santa Catarina (LACEN/SC)	Laboratory of Respiratory Viruses and Measles, Oswaldo Cruz Institute, FIOCRUZ	Paola Resende, Luciana Appolinario, Fernando Motta, Anna Carolina Paixao, Ana Carolina Mendonca, Alice Sampaio Rocha, Taina Venas, Elisa Cavalcante Pereira, Renata Serrano Lopes, Darcita Buerger Rovaris, Sandra Bianchini Fernandes, Marilda Siqueira on behalf of the Fiocruz COVID-19 Genomic Surveillance Network
EPI_ISL_2543500	Genetica Molecular and Subdepartamento de Virologia ISP Chile	Instituto de Salud Publica de Chile	Karen Orostica, Constanza Campano, Barbara Parra, Loredana Arata, Gisselle Barra, Patricia Bustos, Rodrigo Fasce, Javier Tognarelli, Andres Castillo, Soledad Ulloa, Jorge Fernandez
EPI_ISL_2551528	Belo Horizonte center-south emergency care unit - UPA-BH	Laboratório de Virologia Clínica e Molecular	Erick Gustavo Dorlass, Karine Lima Lourenço, Rubens Daniel Miserani Magalhães, Hugo Sato, Alex Fiorini, Renata Peixoto, Helena Perez Coelho, Ana Paula Salles Fernandes, Bruna Larotonda Telezynski, Guilherme Pereira Scagion, Tatiana Ometto, Luciano Matsumiya Thomazelli, Danielle Bruna Leal Oliveira, Edison Luiz Durigon, Flavio Fonseca e Santuza Teixeira
EPI_ISL_2557280, EPI_ISL_2557290	Genetica Molecular and Subdepartamento de Virologia ISP Chile	Instituto de Salud Publica de Chile	Karen Orostica, Constanza Campano, Barbara Parra, Loredana Arata, Gisselle Barra, Patricia Bustos, Rodrigo Fasce, Javier Tognarelli, Andres Castillo, Soledad Ulloa, Jorge Fernandez
EPI_ISL_2557359, EPI_ISL_2557361	Lboratorio Central de Saude Publica do Estado do Parana (LACEN/PR)	Laboratory of Respiratory Viruses and Measles, Oswaldo Cruz Institute, FIOCRUZ	Paola Resende, Luciana Appolinario, Fernando Motta, Anna Carolina Paixao, Ana Carolina Mendonca, Alice Sampaio Rocha, Taina Venas, Elisa Cavalcante Pereira, Renata Serrano Lopes, Irina Riediger, Marilda Siqueira on behalf of the Fiocruz COVID-19 Genomic Surveillance Network
EPI_ISL_2557588	Infinity Biologix	Centers for Disease Control and Prevention Division of Viral Diseases, Pathogen Discovery	Dakota Howard, Dhwani Batra, Peter W. Cook, Kara Moser, Adrian Paskey, Jason Caravas, Benjamin Rambo-Martin, Shatavia Morrison, Christopher Gulvick, Scott Sammons, Yvette Unoarumhi, Darlene Wagner, Matthew Schmerer, Christian Bixby, Yihe Wang, Jonathan Schultz, Chirayu Goswami, Russ Hager, Robin Grimwood, Clinton R. Paden, Duncan MacCannell
EPI_ISL_2597263, EPI_ISL_2597280	Genetica Molecular and Subdepartamento de Virologia ISP Chile	Instituto de Salud Publica de Chile	Karen Orostica, Constanza Campano, Barbara Parra, Loredana Arata, Gisselle Barra, Patricia Bustos, Rodrigo Fasce, Javier Tognarelli, Andres Castillo, Soledad Ulloa, Jorge Fernandez
EPI_ISL_2601693	Hospital General Universitario Gregorio Marañón	Hospital General Universitario Gregorio Marañón	Sergio Buenestado Serrano, Pedro Sola Campoy, Laura Pérez-Lago, Cristina Rodriguez-Grande, Marta Herranz Martin, Victor Manuel de la Cueva, Julia Suárez, Pilar Catalán, Patricia Muñoz, Darío García de Viedma
EPI_ISL_2603447, EPI_ISL_2603449, EPI_ISL_2603584	Laboratorio Central de Saude Publica do Estado do Rio Grande do Sul (LACEN-RS)	Laboratory of Respiratory Viruses and Measles, Oswaldo Cruz Institute, FIOCRUZ	Paola Resende, Luciana Appolinario, Fernando Motta, Anna Carolina Paixao, Ana Carolina Mendonca, Alice Sampaio Rocha, Taina Venas, Elisa Cavalcante Pereira, Renata Serrano Lopes, Anderson Brandao Leite, Marilda Siqueira on behalf of the Fiocruz COVID-19 Genomic Surveillance Network
EPI_ISL_2614210, EPI_ISL_2614253, EPI_ISL_2614319	Laboratory of Respiratory Viruses and Measles, Oswaldo Cruz Institute, FIOCRUZ	Laboratory of Respiratory Viruses and Measles, Oswaldo Cruz Institute, FIOCRUZ	Paola Resende, Luciana Appolinario, Fernando Motta, Anna Carolina Paixao, Ana Carolina Mendonca, Alice Sampaio Rocha, Taina Venas, Elisa Cavalcante Pereira, Renata Serrano Lopes, Marilda Siqueira on behalf of the Fiocruz COVID-19 Genomic Surveillance Network
EPI_ISL_2614582	Unidade Mista de Saude	Instituto Adolfo Lutz, Interdiciplinary Procedures Center, Strategic Laboratory	Claudio Tavares Sacchi, Claudia Regina Gonçalves, Erica Valessa Ramos Gomes, Karoline Rodrigues Campos, Caio Vinicius Dias Lopes, Leonardo Jose Tadeu de Araujo
EPI_ISL_2631900, EPI_ISL_2634510, EPI_ISL_2634637	Eurofins LifeCodexx GmbH	Robert Koch Institute	unknown
EPI_ISL_2663430	Genetica Molecular and Subdepartamento de Virologia ISP Chile	Instituto de Salud Publica de Chile	Karen Orostica, Constanza Campano, Barbara Parra, Loredana Arata, Gisselle Barra, Patricia Bustos, Rodrigo Fasce, Javier Tognarelli, Andres Castillo, Soledad Ulloa, Jorge Fernandez
EPI_ISL_2677094, EPI_ISL_2677134, EPI_ISL_2677135	Labortorio Central de Saude Publica do Estado de Santa Catarina (LACEN/SC)	Laboratory of Respiratory Viruses and Measles, Oswaldo Cruz Institute, FIOCRUZ	Paola Resende, Luciana Appolinario, Fernando Motta, Anna Carolina Paixao, Ana Carolina Mendonca, Alice Sampaio Rocha, Taina Venas, Elisa Cavalcante Pereira, Renata Serrano Lopes, Darcita Buerger Rovaris, Sandra Bianchini Fernandes, Marilda Siqueira on behalf of the Fiocruz COVID-19 Genomic Surveillance Network
EPI_ISL_416036	National Influenza Center - Instituto Adolfo Lutz	Instituto Adolfo Lutz, Interdiciplinary Procedures Center, Strategic Laboratory	Claudio Tavares Sacchi, Claudia Regina Gonçalves, Carlos Henrique Camargo, Erica Valessa Ramos Gomes, Fabiana Cristina Pereira dos Santos, Daniela Bernardes Borges da Silva, Simone Guadagnucci Morillo, Adriano Abbud, Adriana Bugno, Maria do Carmo Sampaio Tavares Timenetsky, Terezinha Maria de Paiva
EPI_ISL_515548	Hospital Municipal Dr. Jose Soares Hungria	Instituto Adolfo Lutz, Interdiciplinary Procedures Center, Strategic Laboratory	Claudio Tavares Sacchi, Claudia Regina Gonçalves, Erica Valessa Ramos Gomes
EPI_ISL_527862	Hospital Municipal de Urgência	Instituto Adolfo Lutz, Interdiciplinary Procedures Center, Strategic Laboratory	Claudio Tavares Sacchi, Claudia Regina Gonçalves, Erica Valessa Ramos Gomes
EPI_ISL_770585	Laboratório de Microbiologia Molecular - Universidade FEEVALE	Bioinformatics Laboratory / LNCC	Felipe Benites, Fernando Rosado Spilki, Alana Witt Hansen, Juliane Deise Fleck, Juliana Schons, Meriane Demoliner, Ana Karolina Eisen Antunes, Fagner Henrique Heldt, Larissa Mallmann, Bruna Hermann, Ana Luiza Ziulkoski, Vyctoria Goes, Karoline Schallenberger, Matheus Nunes Weber, Paula Rodrigues de Almeida, Alessandra Pavan Lamarca da Silva, Ronaldo da Silva F Jr , Luiz G P de Almeida, Alexandra L Gerber , Ana Paula de C Guimarães,Ana Tereza R de Vasconcelos
EPI_ISL_776756	Instituto Adolfo Lutz - Central	Instituto Adolfo Lutz, Interdiciplinary Procedures Center, Strategic Laboratory	Claudio Tavares Sacchi, Claudia Regina Gonçalves, Erica Valessa Ramos Gomes, Karoline Rodrigues Campos
EPI_ISL_792680, EPI_ISL_792683	Pathogen Genomics Center, National Institute of Infectious Diseases	Pathogen Genomics Center, National Institute of Infectious Diseases	Tsuyoshi Sekizuka, Kentaro Itokawa, Rina Tanaka, Masanori Hashino, Makoto Kuroda
EPI_ISL_833137	Laboratorio de Ecologia de Doencas Transmissiveis na Amazonia, Instituto Leonidas e Maria Deane - Fiocruz Amazonia	Laboratorio de Ecologia de Doencas Transmissiveis na Amazonia, Instituto Leonidas e Maria Deane - Fiocruz Amazonia	Valdinete Nascimento, Victor Souza, André Corado, Fernanda Nascimento, George Silva, Ágatha Costa, Debora Duarte, Karina Pessoa, Matilde Mejía, Luciana Gonçalves, Maria Júlia Brandão, Michele Jesus, Felipe Naveca on behalf of the Fiocruz COVID-19 Genomic Surveillance Network
EPI_ISL_833173	DB Diagnosticos do Brasil	Instituto Adolfo Lutz, Interdiciplinary Procedures Center, Strategic Laboratory	Claudio Tavares Sacchi, Claudia Regina Gonçalves, Erica Valessa Ramos Gomes, Karoline Rodrigues Campos
EPI_ISL_848593	Evandro Chagas Institute	Evandro Chagas Institute	Santos, M.C.; Silva, A.M.; Junior, W.D.C.; Barbagelata, L.S.; Ferreira, J.A.; Sousa, E.M.A.; da Silva, P.S.; Pinheiro, K.C.; L.C.; Sousa Junior, E.C.
EPI_ISL_906081	Hospital Beneficiencia Portuguesa	Instituto Adolfo Lutz, Interdiciplinary Procedures Center, Strategic Laboratory	Claudio Tavares Sacchi, Claudia Regina Gonçalves, Erica Valessa Ramos Gomes, Karoline Rodrigues Campos
EPI_ISL_926446	LACEN - Laboratório Central de Saúde Pública do Amazonas	Evandro Chagas Institute Virology	Santos, M.C.; Silva, A.M.; Junior, W.D.C.; Barbagelata, L.S.; Ferreira, J.A.; Sousa, E.M.A.; da Silva, P.S.; Pinheiro, K.C.; L.C.; Sousa Junior, E.C.
EPI_ISL_940615	LACEN-PI DR. Costa Alvarenga	Instituto Adolfo Lutz, Interdiciplinary Procedures Center, Strategic Laboratory	Claudio Tavares Sacchi, Claudia Regina Gonçalves, Erica Valessa Ramos Gomes, Karoline Rodrigues Campos

EPI_ISL_943987	LACEN do Estado de Tocantins	Instituto Adolfo Lutz, Interdiciplinary Procedures Center, Strategic Laboratory	Claudio Tavares Sacchi, Claudia Regina Gonçalves, Erica Valessa Ramos Gomes, Karoline Rodrigues Campos
EPI_ISL_956289, EPI_ISL_956292, EPI_ISL_956297	Instituto Nacional de Salud- Dirección de Redes de Laboratorios de Salud Pública	Instituto Nacional de Salud- Dirección de Investigación en Salud Pública	Katherine Laiton-Donato, Diego A. Álvarez-Díaz, Carlos Franco-Muñoz, Mauricio Pacheco-Montealegre, Hector Alejandro Ruiz-Moreno, Maria T. Herrera-Sepúlveda, Diego Andrés Prada, Jhonnatan Reales-González, Sheryll Corchuelo, Julian Naizaque, Gerardo Santamaría, Magdalena Wiesner, Martha Lucia Ospina Martinez, Marcela Mercado-Reyes
EPI_ISL_983865, EPI_ISL_984620	Central Laboratory of Public Health of Rio Grande do Sul (Lacen-RS)	State Center for Health Surveillance of the Health Department of the State of Rio Grande do Sul (CEVS/SES-RS)	Aline Campos, Cynthia Molina, Lara Crescente, Leticia Garay, Ludmila Fiorenzano Baethgen, Richard Salvato, Tatiana Gregianini
EPI_ISL_985303	LACEN do Estado de Goias	Instituto Adolfo Lutz, Interdiciplinary Procedures Center, Strategic Laboratory	Claudio Tavares Sacchi, Claudia Regina Gonçalves, Erica Valessa Ramos Gomes, Karoline Rodrigues Campos
